# Prolonged CD38 targeting with felzartamab in antibody-mediated kidney transplant rejection: a biomarker-guided open-label phase 2 extension

**DOI:** 10.1016/j.lanepe.2026.101768

**Published:** 2026-07-09

**Authors:** Katharina A. Mayer, Matthias Diebold, Eva V. Schrezenmeier, Philip F. Halloran, Susanne Haindl, Martina Schatzl, Aylin Akifova, Daniela M. Allmer, Sabine Schranz, Nicolas Kozakowski, Johannes Kläger, Kerstin Amann, Julia Beck, Ekkehard Schütz, Maarten Naesens, Alexandre Loupy, Marc Raynaud, Irene Görzer, Hannes Vietzen, Gordon Ingle, Donna L. Flesher, Uptal D. Patel, Fabian Halleck, Irene Graf, Bernd Jilma, Klemens Budde, Georg A. Böhmig

**Affiliations:** a Division of Nephrology and Dialysis, Department of Medicine III, Medical University of Vienna, Vienna, Austria; b Clinic for Transplantation Immunology and Nephrology, University Hospital Basel, University of Basel, Basel, Switzerland; c Department of Nephrology and Medical Intensive Care, Charité Universitätsmedizin Berlin, Berlin, Germany; d Alberta Transplant Applied Genomics Centre, Faculty of Medicine & Dentistry, University of Alberta, Edmonton, Alberta, Canada; e Department of Clinical Pharmacology, Medical University of Vienna, Vienna, Austria; f Department of Clinical Pathology, Medical University of Vienna, Vienna, Austria; g Department of Nephropathology, University of Erlangen-Nürnberg, Erlangen, Germany; h Chronix Biomedical GmbH, Göttingen, Germany; i Department of Microbiology, Immunology and Transplantation, KU Leuven, Leuven, Belgium; j Paris Institute for Transplantation and Organ Regeneration INSERM, Paris, France; k Center of Virology, Medical University of Vienna, Vienna, Austria; l Biogen, South San Francisco, CA, USA; m Division of Hematology and Hemostaseology, Department of Internal Medicine I, Medical University of Vienna, Vienna, Austria; n Insight Molecular Diagnostics, Nashville, TN, USA

**Keywords:** Antibody-mediated rejection, CD38, Clinical study, Natural killer cells, Therapeutic antibody

## Abstract

**Background:**

Antibody-mediated rejection (AMR) is a major cause of kidney transplant failure. The CD38 antibody felzartamab has been shown to reduce AMR activity, but recurrence after stopping treatment suggested a need for sustained therapy. We extended a placebo-controlled phase 2 trial (NCT05021484) to assess the feasibility and durability of prolonged, biomarker-guided treatment.

**Methods:**

Of the 21 patients who completed the primary study, eleven patients with recurrent or persistent AMR after treatment discontinuation received felzartamab for an additional 12 months (16 mg/kg IV): 6 months of fixed dosing followed by 6 months of donor-derived cell-free DNA (dd-cfDNA)–guided dosing. Endpoints included biopsy findings, dd-cfDNA, donor-specific antibodies (DSA), natural killer (NK) cell dynamics, urinary chemokines, kidney function, and safety.

**Findings:**

Felzartamab (median of two doses during the biomarker-guided phase) was associated with changes in rejection activity and stable kidney function. Median microvascular inflammation decreased from 2 (IQR 2–2) to 0 (0–2) at week 52, with 7 of 11 patients (64%) showing a score of 0; one developed low-grade intimal arteritis. Molecular AMR probability declined from 0.77 (0.58–0.87) to 0.12 (0.08–0.35). Overall, dd-cfDNA, NK cells and chemokines decreased, whereas DSA remained largely unchanged. Treatment was well tolerated, with mild-to-moderate infusion reactions and no treatment discontinuations.

**Interpretation:**

Re-dosing and prolonged CD38 targeting was associated with lower AMR activity in most patients despite heterogeneity, supporting AMR as a chronic process that may benefit from ongoing immunomodulation. dd-cfDNA-guided dosing was feasible, with variable dose requirements and effects. Larger and longer trials are required to determine optimal dosing and long-term benefit.

**Funding:**

Biogen (unrestricted grant). Insight (in kind dd-cfDNA measurements).

**Research in context**

**Evidence before this study**

We searched PubMed for studies published up to April 1st, 2026 (no restriction in the start date), using combinations of the search terms “kidney transplantation”, “antibody-mediated rejection”, “rejection treatment”, “CD38”, “plasma cells”, “natural killer cells”, and “donor-derived cell-free DNA”. Reference lists of relevant articles and reviews were also screened. No language restrictions were applied. We included clinical trials, cohort studies, case series, and translational studies evaluating the pathophysiology of antibody-mediated rejection (AMR) or therapeutic targeting of CD38-expressing immune cells in kidney transplantation. We excluded studies focusing on T cell-mediated rejection, studies unrelated to kidney transplantation, and preclinical studies without direct relevance to transplantation outcomes. The overall quality of the evidence was variable: most observational studies and case series were at moderate-to-high risk of bias due to confounding and selection bias, while available randomized studies were limited and generally at low-to-moderate risk of bias. However, emerging evidence indicates that late AMR is a major cause of kidney allograft failure, and effective therapeutic options remain limited. Standard interventions focused on antibody removal or B-cell depletion often fail to halt ongoing graft injury, particularly in late or chronic AMR, and high-quality randomised evidence remains scarce. Growing experimental and translational evidence implicates CD38-expressing immune cells—including plasma cells and natural killer (NK) cells—as mediators of persistent microvascular injury and antibody-dependent cellular cytotoxicity. Small case series and uncontrolled reports of off-label CD38 antibody use, mainly daratumumab, have suggested potential benefit but were limited by small sample size, short follow-up, and a lack of standardised endpoints. A recent randomised phase 2 trial of felzartamab suggested significant short-term improvements in histological and molecular markers of AMR and reductions in donor-derived cell-free DNA (dd-cfDNA), despite minimal effects on donor-specific antibody (DSA) levels. However, recurrence of rejection-associated biomarkers after treatment discontinuation raised concerns about the durability of response with finite therapy.

**Added value of this study**

This open-label extension study provides evidence that continued felzartamab therapy can sustain AMR control over an additional 12-month period in most patients. Prolonged CD38 targeting was associated with depletion of circulating NK cells and suppression of molecular and morphological rejection activity and of inflammatory urinary chemokines, while preserving indices of chronic injury and allograft function. However, treatment responses were heterogeneous, with a subset of patients demonstrating persistent or recurrent rejection activity despite ongoing therapy, indicating biological variability and suggesting that not all late AMR is equally dependent on CD38-expressing effector pathways. These effects occurred in the absence of meaningful reductions in circulating DSA, reinforcing the concept that downstream effector mechanisms, rather than antibody levels alone, are central drivers of tissue injury in many—but not all—cases of late AMR. Importantly, this study is the first to prospectively apply a dd-cfDNA–guided individualised maintenance strategy for AMR, demonstrating the feasibility of biomarker-informed retreatment to maintain disease control while limiting cumulative drug exposure in some patients and potentially preventing unnecessary biopsies. Extended felzartamab exposure was generally well tolerated, with no treatment discontinuations due to adverse events.

**Implications of all the available evidence**

Taken together, available evidence supports targeting CD38-expressing effector cells as an effective therapeutic strategy in late AMR. These findings reinforce a shift in disease conceptualisation—from a condition amenable to time-limited rescue therapy to a chronic alloimmune process that may benefit from sustained or adaptive immunomodulation. Incorporation of injury-based biomarkers such as dd-cfDNA into treatment algorithms may enable personalised maintenance approaches that achieve durable rejection control while minimising unnecessary immunosuppression. The present findings provide mechanistic insight and a strong rationale for larger, confirmatory trials evaluating biomarker-guided CD38-targeted therapies in AMR.

## Introduction

Despite advances in immunosuppressive therapy, antibody-mediated rejection (AMR) remains a leading cause of kidney allograft loss.^1^^,^^2^ AMR drives higher healthcare utilisation, including hospitalisations, outpatient visits, and costly therapies, resulting in higher direct medical costs.^3^ Conventional treatments, including apheresis, intravenous immunoglobulin, B cell-depleting antibodies, and complement inhibition, provide incomplete benefit, and robust randomised evidence is limited.^4^^,^^5^ There is an unmet need for targeted therapies that sustainably reduce alloimmune injury with minimal toxicity.

CD38, a transmembrane ectoenzyme expressed on plasma cells, plasmablasts, and natural killer (NK) cells, has emerged as a promising therapeutic target.^6^ By targeting CD38-expressing immune cells, CD38 monoclonal antibodies may attenuate antibody-mediated and NK cell–driven microvascular inflammation (MVI) and graft injury. Case reports, small series, and a recent randomized controlled trial have suggested therapeutic benefit in patients with AMR.^7^, ^8^, ^9^, ^10^ Felzartamab, an investigational CD38 antibody, was evaluated in a randomised, double-blind, placebo-controlled phase 2 trial.^10^ Treatment was associated with changes in histological and molecular features of AMR and with reductions in donor-derived cell-free DNA (dd-cfDNA), a marker of graft injury,^11^ without significantly lowering donor-specific antibody (DSA) levels.^10^^,^^12^ Concurrent NK cell depletion is consistent with a possible role of NK cells as effectors of graft injury, supporting targeting downstream immune mechanisms beyond antibody production. Felzartamab was well tolerated aside from mild to moderate first–dose reactions. The phase 2 trial demonstrated a signal of efficacy and acceptable tolerability supporting initiation of an ongoing phase 3 trial (ClinicalTrials.gov number: NCT06685757).

However, following treatment cessation at 6 months during the phase 2 study, most participants demonstrated recurrent molecular rejection and occasional histological rebound, suggesting that a 6-month treatment course alone may be insufficient for durable disease control.^10^^,^^12^ Optimal retreatment strategies are undefined. Biomarkers of graft injury and inflammation may nonetheless be informative in characterizing disease persistence following therapy withdrawal. In this context, dd-cfDNA, a marker of rejection-associated graft injury,^11^^,^^13^^,^^14^ represents a promising candidate to monitor ongoing or reemerging immune activity off-treatment, supported by recent case reports and findings from the felzartamab phase 2 trial.^8^^,^^10^

We hypothesised that felzartamab retreatment and continued dosing beyond 6 months, including a dd-cfDNA–guided personalised phase, would be feasible and associated with sustained suppression of AMR activity and attenuation of graft injury, even in patients with a history of persistent or recurrent rejection. To test this, the phase 2 trial was extended into an open-label 12-month study: 6 months of fixed-dose treatment followed by 6 months of personalised, dd-cfDNA-guided dosing, with comprehensive histologic, molecular, and non-invasive biomarker assessment.

## Methods

### Study design

We report the open-label extension phase of an investigator-initiated, randomised, double-blind, placebo-controlled phase 2 trial evaluating felzartamab in kidney transplant recipients with active or chronic-active AMR ≥180 days after transplantation (ClinicalTrials.gov identifier: NCT05021484; EUDRACT 2021-000545-40). The trial was conducted at two centres; Medical University of Vienna, Austria, and Charité Universitätsmedizin Berlin, Germany. The primary study design and the results of the randomised primary trial have been reported previously.^10^^,^^12^ After completion and unblinding of the primary trial, which demonstrated potential treatment efficacy of felzartamab in a randomized setting, and following protocol amendments, eligible patients who provided consent entered the extension study. They received open-label felzartamab if persistent (after previous placebo treatment) or recurrent (after previous felzartamab treatment) AMR activity was present. The 12-month extension consisted of a 6-month fixed-dose treatment phase (nine infusions of felzartamab, identical to the initial phase 2 protocol), followed by a 6-month dd-cfDNA–guided treatment phase (Supplementary Figures S1 and S2). After a high incidence of infusion-related reactions (IRR) was observed, the premedication schedule and infusion regimen were modified by protocol amendment. All patients provided written informed consent, and the study was conducted with external monitoring in accordance with the Declaration of Helsinki and Good Clinical Practice and CONSORT guidelines.

### Participants

Eleven adult patients (>18 years) previously enrolled in the primary randomized trial (22 randomised; 21 completed) entered the open-label extension (Supplementary Figure S1). Key inclusion criteria were completion of the randomised phase, a functioning allograft with an estimated glomerular filtration rate (eGFR) ≥15 mL/min/1.73 m^2^ (CKD-EPI), and persistent or recurrent active or chronic-active AMR on the 12-month follow-up biopsy or an indication biopsy after completion of the primary study. In addition, eligibility required evidence of pharmacodynamic activity (i.e., NK cell depletion or reduction in donor-specific antibodies) and/or a 6-month biopsy demonstrating a response to therapy during the first period of the primary trial (but AMR activity at the end of the trial); these criteria were met by all patients considered for inclusion. Full inclusion and exclusion criteria are provided in the Appendix (Supplementary Table S1), and the study flowchart and schematic overview are shown in the Appendix (Supplementary Figures S1 and S2). Patients received their first felzartamab dose in the extension phase a median of 4.8 months (IQR: 1.9–12.4 months) after the end-of-study visit of the primary study.

### Treatment

Felzartamab was administered intravenously at 16 mg/kg per infusion. The first nine infusions in the fixed schedule were given between day 0 and week 20. To reduce IRR incidence, patients received premedication prior to the first two infusions (Appendix p 2). After week 24, treatment transitioned to an individualized, on-demand regimen, guided by monthly measurements of absolute and relative dd-cfDNA levels. Patients received felzartamab at the next visit if any of the following thresholds were exceeded: relative dd-cfDNA ≥0.5%; absolute dd-cfDNA ≥50 copies/mL; or doubling of relative or absolute dd-cfDNA compared with week-24 baseline.

### Outcomes

Key outcomes are listed in the Appendix (Supplementary Table S2).

#### Biopsy endpoints

Follow-up biopsies were scheduled at weeks 24 and 52. Biopsies were graded using Banff 2022 criteria,^15^ morphologic activity and chronicity composite scores,^16^ and Molecular Microscope Diagnostic System (MMDx) analysis,^17^ as described in the Appendix (p 2).

#### Non-invasive endpoints

These included DSA number and mean fluorescence intensity (MFI), absolute and relative dd-cfDNA levels and their association with histology and transcriptome, peripheral blood CD56^dim^CD16^bright^ NK cell counts, urinary chemokines C–X–C motif chemokine ligands 9 and 10 (CXCL9 and CXCL10), Torque Teno virus (TTV) viremia, kidney function (eGFR), and protein-creatinine ratio. Laboratory assays are detailed in the Appendix (pp 2–3).

Details regarding iBox combined surrogate endpoint,^18^ safety monitoring and adverse event reporting are provided in the Appendix (p 3).

### Statistical analysis

For this exploratory extension phase, analyses were primarily descriptive. Continuous variables are reported as median (IQR) or mean (SD), and categorical variables as counts and percentages. Within-patient changes over time were evaluated descriptively; no formal hypotheses were prespecified for efficacy endpoints. Changes in key biopsy outcomes (MVI and a molecular classifiers reflecting AMR activity [AMR_Prob_]) across baseline, week 24, and week 52 were analysed using the Friedman test for repeated measures. Post-hoc pairwise comparisons were performed using Wilcoxon signed-rank tests with false discovery rate (FDR) correction for multiple testing. eGFR slopes were estimated using linear regression of individual longitudinal measurements. Adverse events (AE) and serious adverse events (SAE) were summarised descriptively. Analyses were performed using R version 4.0.2 (R Foundation for Statistical Computing; https://www.R-project.org). Packages used are listed in the Appendix (pp 3–4). Graphical representations were created using Biorender.com.

### Role of the funding source

The trial was financed by Biogen through an unrestricted grant awarded to the sponsor, Medical University of Vienna. The investigators and sponsor designed the study, collected and analysed the data, and prepared the report. The funder reviewed the manuscript but had no role in data collection or data analysis. Insight Molecular Diagnostics provided in kind dd-cfDNA measurements.

## Results

Twenty-two patients with AMR were initially enrolled in the randomised phase of the felzartamab phase 2 trial. After unblinding and database lock (March 2024), 11 patients with persistent or recurrent AMR activity entered the open-label extension (first visit: April 2024; final visit at week 52: February 2026) (Supplementary Figures S1 and S2). Baseline characteristics are summarised in Table 1 and in Supplementary Tables S3 and S4. Four patients were female and seven were male; two were Asian and nine White. Median age at extension entry was 54 years, and median eGFR 39 mL/min/1.73 m^2^. Ten patients received triple immunosuppression (tacrolimus or in one patient cyclosporine A, mycophenolic acid, steroids). Maintenance immunosuppression levels and dosing are shown in Supplementary Figure S3. Banff 2022 histologic phenotypes were chronic active AMR (n = 6), active AMR (n = 3), and probable AMR (n = 2); the latter included based on a high molecular AMR_Prob_ score. No T cell-mediated rejection (TCMR) or borderline rejection was observed. Molecular phenotyping showed a median AMR_Prob_ score of 0.77 (0.58–0.87), with a fully-developed AMR phenotype in eight patients; a molecular classifier reflecting TCMR (TCMR_Prob_) was negative. At inclusion, nine patients had detectable DSA; two were DSA-negative (with prior DSA positivity). Baseline rejection features were comparable between initial felzartamab and placebo groups (Supplementary Table S4).

**Table 1:** Summary of demographic and baseline clinical characteristics.

Variables	Total (N = 11)
Recorded at transplantation	
Female sex	4 (36)
Recipient age, years	40 (32–53)
Race^a^	
White	9 (82)
Asian	2 (18)
Living donor	4 (36)
Donor age, years	43 (30–49)
Prior kidney transplant	3 (27)
HLA (A, B, DR) mismatch	3 (2–4)
Cold ischaemia time, hr	10 (3–12)
CDC panel reactivity ≥10%	0 (0.0)
Missing data, no.	2 (18)
Preformed anti-HLA DSA^b^	3 (27)
Missing data	5 (46)
Recorded at inclusion in the extension study	
Previous felzartamab treatment	7 (64)
Age at inclusion, years	54 (44–59)
Time from transplantation to inclusion, years	10 (5–20)
eGFR, mL/min/1.73 m^2^	39 (28–57)
Protein/creatinine ratio, mg/g	539 (294–1334)
Triple immunosuppression	10 (91)
Tacrolimus-based immunosuppression	9 (82)
ACE inhibitor/ARB therapy	9 (82)
SGLT2 inhibitor therapy	2 (18)
DSA characteristics	
HLA class I DSA only	0 (0.0)
HLA class II DSA only	6 (55)
HLA class I and II DSA	3 (27)
Number of DSA	2 (1–3)
MFI of the immunodominant DSA	3025 (1881–5761)

Data are n (%) or median (IQR).

ACE = angiotensin converting enzyme; AMR = antibody-mediated rejection; CDC = complement-dependent cytotoxicity; DSA = donor-specific antibody; eGFR = estimated glomerular filtration rate; HLA = human leukocyte antigen; IQR = interquartile range; MFI = mean fluorescence intensity; NK cell = natural killer cell; SGLT2 = sodium glucose cotransporter-2.

a Race and ethnicity were reported by patients.

b Pre-transplant DSA data were available for 6 recipients (solid-phase HLA antibody screening on the wait list was implemented at the Vienna transplant unit in July 2009).

### Treatment exposure

Nine patients completed all scheduled doses during the 6-month fixed-treatment phase; two omitted one dose because of AE. During the subsequent 6-month biomarker-guided phase, patients received a median of two doses (range 1–6; Supplementary Figure S2). The median interval between doses in the dd-cfDNA-guided treatment phase was 12 weeks (IQR 4–16 weeks).

### Morphologic and molecular rejection activity

**Fig. 1:**
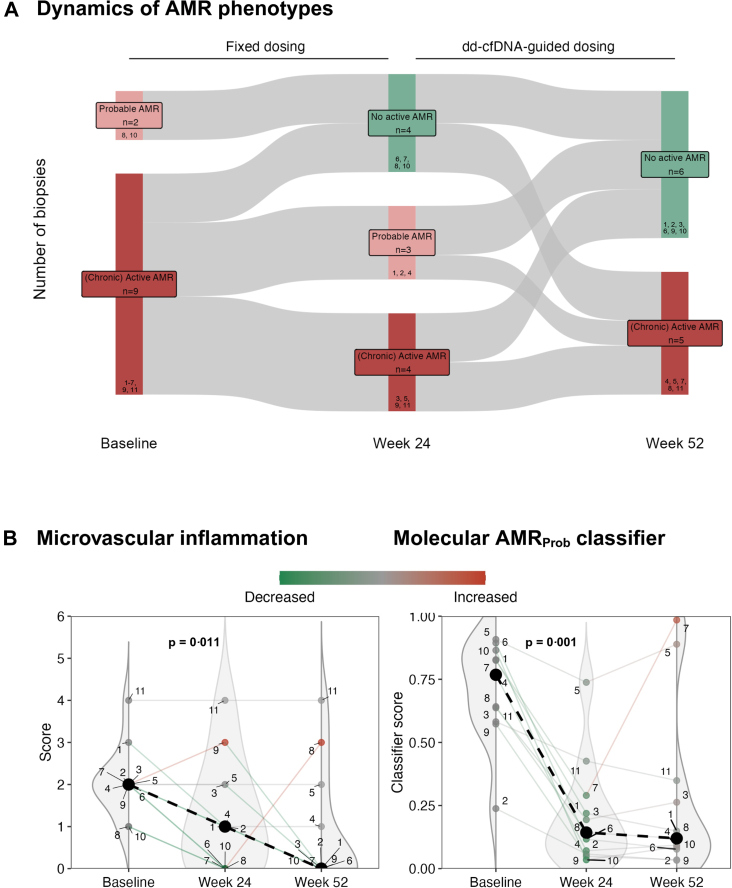
**Effect of felzartamab on antibody-mediated rejection activity.** Panel A shows a Sankey plot depicting changes in morphologic antibody-mediated rejection (AMR) phenotypes (active phenotypes: probable or [chronic] active AMR; phenotypes with no activity: chronic inactive AMR or no rejection) across renal allograft biopsies obtained at baseline, week 24 (fixed dosing), and week 52 (dd-cfDNA–guided dosing). Vertical stacks represent biopsy time points and the number of biopsies performed. Numbers identify individual patients. Grey bands illustrate transitions in biopsy phenotypes over time, with band width proportional to the number of cases. Panel B shows individual trajectories and median changes from baseline to weeks 24 and 52 in microvascular inflammation (MVI) and molecular AMR probability scores, respectively. Shaded areas represent group-wise density estimates. Solid black circles indicate median values, and dashed lines show changes in median values over time. Solid coloured circles represent individual patient values, and solid coloured lines indicate within-patient changes (green, decrease; red, increase). Numbers identify individual patients. Changes in scores across baseline, week 24, and week 52 were analysed using the Friedman test for repeated measures. Post-hoc Wilcoxon signed-rank tests with FDR correction indicated a numerical reduction in MVI between baseline and week 24 (p = 0.065) and between baseline and week 52 (p = 0.065), whereas no difference was observed between week 24 and week 52 (p = 0.50). AMR_Prob_ decreased significantly from baseline to week 24 (p = 0.010) and from baseline to week 52 (p = 0.010), with no difference between week 24 and week 52 (p = 0.824).

Changes in histologic and molecular rejection parameters are shown in Fig. 1, with individual AMR features and trajectories of rejection features and biomarkers in the Appendix (Supplementary Table S5, Supplementary Figure S4). By week 24, four patients (36%) had lost morphologic rejection activity, and three transitioned from (chronic) active AMR to probable AMR (all with g1 lesions). Four patients had persistent (chronic) active AMR (patients 3, 5, 9, and 11). At week 52, after dd-cfDNA–guided therapy, six patients (55%) had no rejection activity, whereas five had (chronic) active AMR per Banff 2022 criteria. Three of them showed a transition from no or probable AMR to an active AMR phenotype during the dd-cfDNA-guided phase of the study (patients 4, 7, and 8). Median MVI decreased significantly from 2 (IQR 2–2) at baseline to 1 (0–2) at week 24 and 0 (0–2) at week 52 (p = 0.011). Seven patients (64%) had an MVI score of 0 at week 52; one (patient 7) had an isolated *de novo* v lesion (score 1), together with C4d positivity, and was classified as active AMR. Two patients (5 and 11) showed no MVI reduction, and one (patient 8) developed recurrent MVI. The three patients without reduction in MVI scores at week 52 (compared to baseline) received 1, 2, and 5 biomarker-guided felzartamab doses, respectively.

Overall, AMR_Prob_ declined significantly from baseline (median 0.77 [IQR 0.58–0.87]) to week 24 (median 0.14 [0.06–0.29]) and remained low at week 52 (0.12 [0.08–0.35]; p = 0.001). Treatment responses, however, were heterogeneous. Two patients (5 and 11) showed only modest reductions after the first treatment phase. Seven patients had levels below the threshold of 0.20. Three patients showed re-increases at week 52: one of those with an MVI score of 0 (patient 3), one with persistent MVI (patient 5) and one with an isolated *de novo* v lesion (patient 7). The latter patient presented with *de novo* C4d staining in absence of MVI. TCMR_Prob_ remained negative throughout, concordant with histology (Supplementary Figure S5).

Numerically, resolution of histologic AMR activity was more frequent among patients who had received placebo in the primary randomized trial (Supplementary Figure S6). Among patients who had received felzartamab in the primary trial, two patients (7 and 8) showed resolution of rejection activity at week 24; both had also shown histologic resolution in the primary trial. The remaining five patients did not achieve complete histologic resolution of AMR activity at 24 weeks. Of these, three patients (1, 3, and 9) had previously shown complete resolution in the parent trial, whereas two (4 and 5) had not and continued to exhibit persistent AMR activity at week 52 despite re-treatment. Molecular AMR scores decreased over the 12-month treatment period in both placebo- and felzartamab-treated subsets (Supplementary Figure S7).

Histologic global activity indices and molecular all rejection transcript scores decreased over time, whereas morphological and molecular chronic injury was largely unchanged (Supplementary Figure S8).

### dd-cfDNA and urinary chemokines

**Fig. 2:**
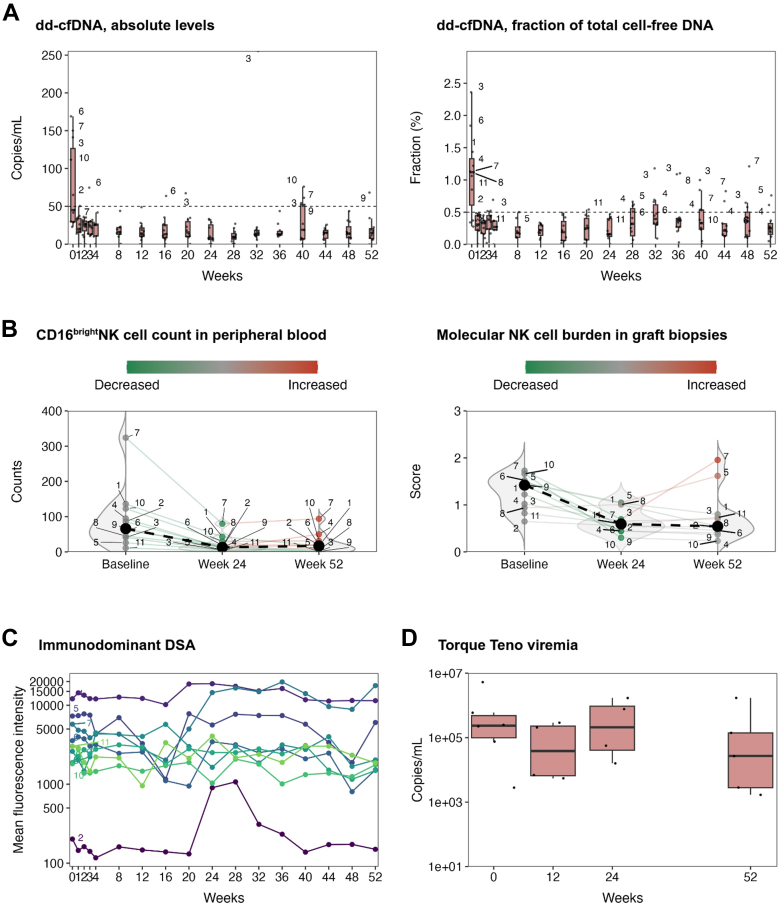
**Effect of felzartamab on donor-derived cell-free DNA, NK cells, antibody formation and overall immunity.** Panel A shows absolute levels and fractions of donor-derived cell-free DNA (dd-cfDNA). Dashed horizontal lines indicate predefined dd-cfDNA thresholds used to guide felzartamab treatment (50 copies/mL and 0.5%, respectively). In box plots, the horizontal line represents the median, boxes indicate the interquartile range (IQR), and whiskers represent 1.5 × IQR. Dots represent individual patient measurements. Panel B shows CD56^dim^CD16^bright^NK cell counts and a molecular pathogenesis-based transcript set reflecting NK cell burden (NKB) at baseline and at weeks 24 and 52. Shaded areas represent group-wise density estimates. Solid black circles indicate median values, and dashed lines show changes in median values over time. Solid coloured circles represent individual patient values, and coloured lines indicate within-patient changes (green, decrease; red, increase). Numbers identify patients. Panel C shows individual trajectories of mean fluorescence intensity of the immunodominant donor-specific antibodies (DSA). Data are shown for the nine patients with detectable DSA at extension study inclusion. Panel D shows Torque Teno virus (TTV) load as a marker of global immune competence during felzartamab treatment. In box plots, the horizontal line represents the median, boxes indicate the IQR, whiskers represent 1.5 × IQR, and dots represent individual patient measurements.

With some heterogeneity, absolute and relative dd-cfDNA (baseline median 45 copies/mL and 1.12%) decreased after the first dose and remained low during fixed dosing (Fig. 2). During the first 24 weeks, dd-cfDNA levels were largely stable, with only occasional marginal threshold exceedances in four patients, which resolved under continued fixed-dose treatment (Fig. 2). During the biomarker-guided treatment phase, triggered by dd-cfDNA increases, median absolute or relative levels immediately before felzartamab infusions (i.e., values that had doubled relative to the 24-week visit or exceeded absolute thresholds) versus 1 month after individual felzartamab infusions were 37.5 (IQR 14.5–59.3) versus 9.5 (6.0–16.8) copies/mL and 0.7 (0.52–0.92) versus 0.2 (0.2–0.3)%, respectively. At week 52, median levels were 15 copies/mL and 0.25%. Three of the five patients with persistent or recurrent (chronic) active AMR at week 52 (patients 7, 8, 11) had dd-cfDNA below diagnostic thresholds, whereas two (patient 4 and 5) had a relative level of 0.6% and 0.76%, marginally above threshold. During treatment we found numerical decrease in urinary CXCL9 and CXCL10, though with considerable inter-patient heterogeneity (Supplementary Figure S9). As shown in Supplementary Figure S10, urinary chemokine levels decreased from baseline in patients without rejection activity at week 52, whereas this was not observed in patients with persistent AMR activity. In contrast, both subsets showed decreases in dd-cfDNA.

### NK cells, antibody levels, and TTV viraemia

Peripheral blood CD56^dim^CD16^bright^ NK cell counts decreased during treatment, accompanied by lower intragraft NK cell transcript levels (Fig. 2). At week 52, patients with persistent or recurrent (chronic) active AMR showed no meaningful differences in the median reduction of NK cell counts or transcript levels compared with patients without rejection activity, although trajectories were heterogenous (Supplementary Figure S10). Immunodominant DSA MFI showed no consistent longitudinal change; no new DSA were recorded. At week 52, three patients with persistent DSA were C4d-positive: one without histologic or molecular AMR activity (patient 6), one with an MVI score of 1 but negative AMR_Prob_ (patient 4), and one with a v lesion associated with a re-increase of AMR_Prob_ (patient 7) (Appendix p 9). Total IgG decreased ∼10%, with larger reductions in IgM and IgA (20–30%) and across IgG subclasses (IgG4 > IgG3 > IgG1/IgG2, 10–40%) (Supplementary Figures S11 and S12). TTV viraemia, reflecting global immunity, remained stable (Fig. 2).

### Kidney function and proteinuria

**Fig. 3:**
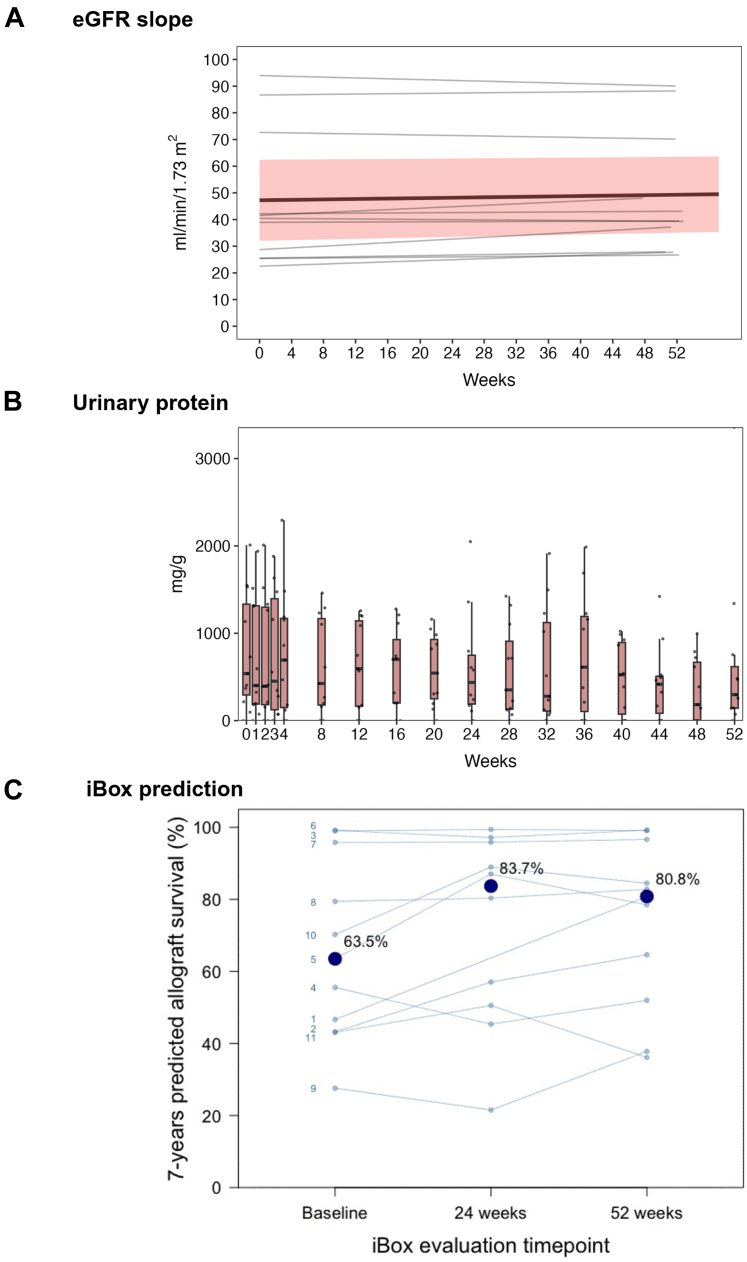
**Effect of felzartamab on estimated glomerular filtration rate (eGFR) and protein excretion.** Panel A shows individual eGFR trajectories (thin lines) and the mean slope (thick line). Shaded areas represent 95% confidence intervals. Panel B shows the course of urine protein-to-creatinine ratio. In box plots, the horizontal line represents the median, boxes indicate the IQR, whiskers represent 1.5 × IQR, and dots represent individual patient measurements. Panel C shows the medians of the iBox-based predicted probabilities of 7-year allograft failure, calculated at baseline, 24 weeks and 52 weeks. Large dots represents medians, and small blue dots represent individual evaluations (each light blue dot corresponds to one evaluation). Numbers identify patients.

Kidney function was stable over the 12-month treatment period. Median eGFR slope was 1.99 min/1.73 m^2^ per year (95% confidence interval: −0.64 to 4.63 min/1.73 m^2^ per year). Protein-to-creatinine ratio showed a numerical decrease from baseline (median 539 [IQR 294–1334] at baseline to 298 [144–619] at week 52) (Fig. 3). Outcomes were similar between prior randomisation groups (Supplementary Figure S13). Using the iBox scoring system, the median predicted 7-year death-censored graft survival was found to increase from 63.5% to 83.7% at week 24 and to 80.8% at week 52 (Fig. 3). The improvement in predicted probabilities of allograft failure was thereby primarily driven by functional parameters, namely eGFR and proteinuria (Supplementary Figure S14).

### Safety outcomes

Safety outcomes are summarised in Table 2 and in the Appendix (Supplementary Tables S6 and S7). Overall, 101 AE occurred: 49 mild, 49 moderate, and three severe. Twenty-eight AE were judged to be treatment-related. Two patients omitted one dose each because of AE, but no patients discontinued felzartamab because of AE. Nine patients experienced IRR (up to three per patient; eight mild, ten moderate), predominantly at the first dose (n = 8), or, in two patients, at the second or third dose. During the biomarker-guided phase, IRR recurred after prolonged treatment-free intervals (one after 2 months; three after 3 months; two after 4 months; one after 5 months; one after 6 months) (Supplementary Figure S2, Supplementary Table S7). Nine patients experienced infections: seven cases of nasopharyngitis, three of pneumonia, two of CMV viraemia, and one each of norovirus and sapovirus infection. One patient developed cutaneous basal cell and squamous cell carcinoma requiring surgery. Seven patients had serious adverse events (SAE), including infection-related SAE (three patients hospitalised for pneumonia, one hospitalised for gastroenteritis), carcinoma in situ of the cervix requiring hysterectomy, one myocardial infarction in a patient with known coronary artery disease, and two electrolyte disturbances. None were considered related to felzartamab. Laboratory safety parameters remained stable (Supplementary Figure S15).

**Table 2:** Adverse events.

Event	Patients (N = 11)	
	Patients with AE, n (%)	Number of AE
Patients with a TEAE	11 (100)	101
Mild	10 (91)	49
Moderate	10 (91)	49
Severe	3 (27)	3
Patients with a TRAE	9 (82)	28
Patients with TEAE of special interest		
Infusion-related reaction^a^	9 (82)	18
Serious AE	7 (64)	8
Pneumonia	3 (27)	3
Myocardial infarction	1 (9.1)	1
Cervix carcinoma	1 (9.1)	1
Hyponatremia	1 (9.1)	1
Hypocalcemia	1 (9.1)	1
Acute gastroenteritis	1 (9.1)	1

AE = adverse event. TEAE = treatment-emergent adverse event. TRAE = treatment-related adverse event.

a Infusion-related reactions were classified as mild (8 events) or moderate (10 events).

## Discussion

In this 12-month open-label extension of a phase 2 trial, we evaluated retreatment and prolonged dd-cfDNA–guided CD38 targeting therapy in kidney transplant recipients with AMR. In the preceding randomised phase, felzartamab reduced MVI and molecular AMR scores, likely through intragraft NK cell depletion. However, molecular and histologic rebound after treatment cessation indicated that AMR behaves as a chronic, self-sustaining effector process that may benefit from continued immunomodulation rather than short-term therapy alone^10^^,^^12^

The principal finding of this extension phase is that retreatment and prolonged CD38 targeting was associated with lower levels of histologic and molecular rejection activity in most patients, with kidney function stable in all participants. Disease control during the biomarker-guided phase was observed in many patients, with variable dosing patterns. Serial biopsies integrated with complementary non-invasive biomarkers confirmed overall treatment responsiveness but revealed pharmacodynamic heterogeneity, with some patients showing persistent or recurrent rejection features. These findings suggest biological heterogeneity in AMR, including cases where injury may be less dependent on CD38-expressing effector pathways and therefore less responsive to CD38-targeted therapy. However, variation in treatment intensity during the biomarker-guided phase may also have contributed to the heterogeneous responses.

A key observation was the close temporal coupling between felzartamab exposure and suppression of dd-cfDNA, supporting the potential of dd-cfDNA as a dynamic non-invasive biomarker for guiding therapy. Importantly, dd-cfDNA reflects overall graft injury—particularly injury associated with AMR and MVI—while avoiding sampling error and variability inherent to kidney biopsy, which assesses only a small portion of the graft.^11^ This is consistent with prior studies demonstrating a strong association between dd-cfDNA levels and molecular and histologic rejection activity^13^^,^^14^ To enhance diagnostic discrimination, we incorporated absolute dd-cfDNA levels alongside relative measurements using previously proposed thresholds to optimise specificity and sensitivity.^19^^,^^20^ However, the optimal thresholds for dd-cfDNA—whether absolute or relative—remain incompletely defined. The rationale for our dd-cfDNA–guided retreatment algorithm, including the use of doubling from the week-24 baseline as a trigger, was to increase sensitivity to dynamic changes in graft injury while accounting for inter-individual baseline differences. However, dd-cfDNA levels may exhibit biological and analytical variability over time, even under stable conditions. In addition, dd-cfDNA is considered a biomarker of the damage to be prevented rather than a pharmacodynamic or functional biomarker that anticipates the damage that should be avoided. Therefore, the applied thresholds may represent a relatively sensitive and potentially more “aggressive” approach, and the algorithm should be considered exploratory and hypothesis-generating. Although dd-cfDNA–guided treatment was associated with stabilisation of graft injury signals in most patients, discordant patterns—including persistent or recurrent MVI and, in one case, isolated *de novo* intimal arteritis despite suppressed dd-cfDNA—highlight the limitations of this approach. Taken together, these observations suggest that dd-cfDNA should be interpreted in conjunction with other clinical and biomarker data. Accordingly, our findings support integrated biomarker frameworks combining tissue and non-invasive markers to guide treatment and dosing strategies. Finally, in our study, interpretation of dd-cfDNA was restricted to the context of AMR, and the study did not systematically assess other causes of graft injury (e.g. polyomavirus-associated nephropathy), which may also influence dd-cfDNA levels in clinical practice.

Individualised dosing was feasible and associated with variable dosing patterns (median two doses over six months), suggesting that biomarker-guided treatment may reduce drug exposure compared with the fixed-dose regimen scheduled for the ongoing felzartamab phase 3 trial (ClinicalTrials.gov identifier: NCT06685757). However, the benefit appeared limited, as most participants ultimately required repeated re-dosing. An inherent limitation of biomarker-triggered retreatment is its reactive nature: treatment begins only after dd-cfDNA elevations signal alloimmune injury, when graft damage may already be underway, which might explain the re-emergency of histologic lesions in the 12-month biopsies. Fixed maintenance dosing may therefore provide more continuous suppression of effector pathways and potentially reduce cumulative injury. Additional practical considerations include recurrence of IRR after prolonged treatment-free intervals and the logistic burden of frequent testing. At the same time, the long-term safety profile of sustained therapy is unknown and prolonged treatment may carry substantial costs. Furthermore, although extending therapy beyond 6 months may improve disease control, longer-term studies are needed to define the optimal treatment duration and determine whether sustained therapy is required.

While biomarker-guided dosing using dd-cfDNA may be feasible, our findings remain preliminary and do not yet support a robust treatment recommendation. Accordingly, given the exploratory and uncontrolled design of our study, its role in guiding therapy requires validation in controlled studies including a head-to-head comparison between fixed-dose and bio-marker-guided treatment regimens. In addition, uncertainty regarding optimal dd-cfDNA thresholds and potential inter-assay variability may affect implementation and interpretation across clinical settings. In contrast, biomarkers reflecting immune effector activity, including NK-cell dynamics, may provide more mechanistically proximal and potentially more reliable signals of treatment response and could merit further exploration in guiding anti-CD38 rejection therapy.

Despite sustained suppression of molecular and morphologic rejection in many patients, immunodominant DSA MFI levels remained largely unchanged, consistent with findings from the preceding randomised trial.^10^ In contrast, felzartamab treatment was associated with depletion of peripheral and intragraft NK cells and numerically reduced levels of CXCL9 and CXCL10, supporting a possible contribution of NK-cell–mediated effector pathways in AMR. Notably, a subset of patients presenting with persistent or recurrent rejection activity showed little reduction—or even re-expansion—of NK cells by week 52, suggesting possible resistance at the target cell level. This pattern was unlikely to reflect insufficient drug exposure, as two of these patients had received three or more doses during the biomarker-guided phase. These observations highlight the need for further studies to clarify potential resistance mechanisms, including changes in CD38 expression on circulating and intragraft NK cells.

Kidney function and indices of chronic injury remained stable in all participants over the 12-month treatment period Although eGFR is a relatively late marker of graft dysfunction, its stabilisation is notable in late AMR, where one-year eGFR slopes of −4 to −8 mL/min are typically observed,^21^, ^22^, ^23^ including among placebo-treated patients in the double-blind phase of the trial.^10^ Kidney function remained stable even in patients with persistent AMR activity, suggesting that rejection activity and functional outcomes may not always progress in parallel. A similar pattern was reported in a recent phase 2 trial of IVIG in patients with chronic active AMR, where treatment did not reduce morphologic rejection activity or DSA levels but was nevertheless associated with stabilisation of kidney function and attenuation of chronic injury progression.^24^ Nonetheless, our observations regarding eGFR trajectories warrant cautious interpretation, as the cohort is small and uncontrolled, limiting conclusions about clinical benefit. Notably, at study entry, 9 of the 11 patients were receiving concomitant therapies known to influence graft function decline, including angiotensin-converting enzyme inhibitors or angiotensin receptor blockers, and in two cases additional treatment with sodium glucose cotransporter-2 inhibitor. Of note, there were no changes in prescribed medications during the study. These agents may have contributed to the observed stability in kidney function and proteinuria. Whether felzartamab itself exerts effects beyond immunological modulation—potentially influencing non-immunological pathways of graft injury—remains an open question.

MVI is a key predictor of graft outcomes^2^ and biopsy-confirmed histologic resolution—largely determined by MVI scores—has recently been accepted by regulatory authorities as a primary endpoint for an ongoing felzartamab phase 3 trial (ClinicalTrials.gov identifier: NCT06685757). Long-term risk may be better captured by integrated prognostic tools such as the iBox model, which was recently evaluated in the phase 2 parental trial.^25^ In line with this, iBox scores showed an increase in this cohort, although these findings should be interpreted cautiously given the limited sample size and lack of a control group, with predicted graft survival probabilities remaining stable. These findings align with prior transcriptomic analyses demonstrating reduced injury and maladaptive repair pathways following felzartamab treatment.^12^ Importantly, the applicability of composite prognostic models such as iBox in the context of emerging targeted therapies warrants careful consideration. These models were developed and validated in cohorts treated with conventional immunosuppression and may not fully capture treatment effects of novel agents that act through alternative mechanisms. In particular, therapies such as felzartamab may attenuate graft injury and improve outcomes without substantially modifying circulating DSA levels—one of the key components of the iBox score. As a result, the risk estimates generated by such models may not fully reflect the true therapeutic benefit in this setting. Future studies are therefore needed to recalibrate or refine prognostic tools to account for mechanism-specific effects of emerging therapies.

In the interpretation of efficacy outcomes, an important consideration is the potential for selection bias inherent to extension cohorts derived from a preceding interventional trial. Because inclusion required survival with a functioning allograft after the parent study, patients entering the extension phase may represent a “transplant survivor” population, potentially underrepresenting more aggressive disease courses that resulted in graft loss or marked functional decline. This may be particularly relevant for participants originally assigned to placebo, although numerically more responders were observed in this group during the main trial. However, given the small sample size, this observation was not powered for comparison and is not interpreted as evidence of differential efficacy. The extension study also selectively enrolled patients with persistent or recurrent MVI in the active treatment arm, potentially enriching for more refractory disease phenotypes. Patients who maintained disease control at the end of the parent study were not included. Furthermore, the cohort was highly selected, predominantly White, and characterised by clinical heterogeneity with respect to kidney function, rejection activity, and chronic injury burden, which may limit generalisability of the findings. Interpretation is further complicated by variable timing between completion of the parent trial and entry into the extension study, resulting in non-uniform baseline conditions across participants. Exploratory analyses further suggested that response in the extension phase may have been influenced by prior response in the parent trial. Among patients re-treated with felzartamab, complete histologic AMR resolution by week 24 occurred only in those who had also previously responded, whereas patients with a prior incomplete response showed more heterogeneous outcomes despite extended treatment up to week 52. Thus, larger studies are needed to further investigate interpatient variability and whether these differences reflect differing pathomechanisms.

The safety profile was consistent with the primary randomised study, with only two felzartamab doses omitted because of AE and no drug-related discontinuations. Given the total of 101 AE (including 7 SAE) observed over 12 months of treatment, the inherently high background rate of AE in transplant recipients must be considered. In this context, it is important to note that in the primary trial, a total of 89 AE (including 7 SAE) were recorded in the placebo group, compared with 119 AE (including 2 SAE) in the felzartamab-treated group. The overall burden and spectrum of adverse events—including infections, malignancies, and cardiovascular events—should therefore, in absence of a control group, be interpreted cautiously in this small cohort, particularly given the potential overlap with known effects of CD38-targeting therapies and concomitant maintenance immunosuppression. SAE occurred in seven patients (eight events in total); all events were judged unrelated to study drug. Given the small sample size and limited follow-up, these observations should be interpreted cautiously and do not exclude uncommon or delayed adverse effects.

Stability of TTV viraemia was observed, which in the context of previous literature may suggest no major impairment of immune competence,^26^ however, its interpretation as a marker of preserved global immunity is limited and should be viewed in the context of the observed infectious complications and reductions in immunoglobulin levels. The latter observation may be clinically relevant and could contribute to infection susceptibility, particularly with prolonged treatment exposure. Although CMV viraemia occurred in two patients and responded to antiviral therapy, no severe opportunistic infections were observed. As in the parent trial,^10^ IRR were common, mild to moderate in severity and typically occurring during the first two infusions or after prolonged treatment-free intervals (3 months or more), possibly due to partial reconstitution of CD38-expressing cells. This observation has important practical implications for adaptive dosing strategies, as recurrence of IRR after treatment interruptions may negatively affect tolerability, patient-reported outcomes, and the feasibility of long-term or intermittent treatment approaches.

In conclusion, AMR appears to be a chronic, self-sustaining process requiring prolonged immunomodulation. dd-cfDNA–guided felzartamab therapy was feasible and, with variable effects, was associated with sustained suppression of molecular and morphologic rejection and stable allograft function in most patients, though repeated redosing was often necessary. Extended initial dosing beyond six months may maintain AMR control. These findings support further investigation of prolonged CD38 targeting in larger, controlled trials.

## Contributors

KAM, KB, and GAB conceived and designed the study, with input from BJ and UDP. KAM, EVS, AA, SS, FH, BJ, KB, and GAB were responsible for trial management, execution, and site coordination. KAM, EVS, PFH, SH, MS, AA, DMA, SS, NK, JK, KA, JB, ES, IGoe, IGra, BJ, KB, and GAB contributed to data collection and patient recruitment. MD performed the statistical analyses with oversight from MN, AL, MR, KB, and GAB. KAM, MD, KB, and GAB had full access to and verified the data underlying the manuscript. KAM, MD, KB, and GAB drafted the manuscript, with critical input and revisions from EVS, PFH, SH, MS, AA, DMA, SS, NK, JK, KA, JB, ES, MN, AL, IGoe, HV, GI, DLF, UDP, FH, MR, IGra, and BJ. All authors had full access to the data and vouch for its accuracy and the fidelity of the trial to the protocol. All authors reviewed and approved the final version of the report and take responsibility for its accuracy and integrity. The decision to submit the manuscript for publication was made collectively by all authors.

## Data sharing statement

De-identified participant data underlying the results reported in this article (including text, tables, figures, and appendices), together with the study protocol, will be made available on reasonable request to the corresponding author. Data will be accessible beginning 9 months after publication of this article and ending 36 months thereafter, and will be available for 5 years following this period. Access will be granted following submission and approval of a methodologically sound research proposal and the signing of a data access agreement.

## Declaration of interests

KAM, SH, MS, DMA, SS, NK, JK, ES, AL, MR, IGoe, HV, and BJ report no competing interests. MD reports travel support from SOBI. EVS reports consulting fees from Astra Zeneca, Novartis, and Otsuka; travel support from Chiesi; and speaker fees from AstraZeneca, Novartis, and GSK. PFH reports shares in Transcriptome Sciences Inc., consulting fees and institutional research grants from Natera; and a license to Thermo Fisher. AA reports travel support from iMDx. KA reports advisory board participation and speaker compensation from Amicus, Novartis, and Vifor. JB reports being an inventor of the dd-cfDNA technology used (patents: US11,155,872B2; US10,570,443B2; US20230121271A1) and employee of Chronix Biomedical GmbH (stock options), a subsidiary of Insight Molecular Diagnostics Inc (iMDx). MN reports speaker fees from Biogen. GI, DLF, UDP are employees of Biogen and hold stock in Biogen. FH reports consulting fees from Hansa, MSD, Chiesi, Takeda, and Sanofi; participation on a Data Safety Monitoring Board or Advisory Board for the TOL-2 Study (TolerogenixX GmbH); and support for attending meetings or travel from Hansa. IGra reports travel support from AbbVie, Novartis, and Pfizer. KB reports research funds, travel support and/or honoraria from: Aicuris, Alexion, Astellas, AstraZeneca, Bayer, Biogen, Bristol-Myers Squibb, Biohope, Carealytics, CareDx, Chiesi, CSL Behring, Daiichii Sankyo, DTB GmbH, Eledon, Fresenius, Hansa, HiBio, iMDx, MSD, Natera, Neovii, Oncocyte, Oska, Otsuka, Paladin, Pfizer, Pirche, Sanofi, smart care solutions, Stada, Takeda, Veloxis, Vifor and Xenothera. GAB reports consulting fees from Argenx, Alexion, Biogen, CSL-Behring, Hi-Bio, SOBI, and Takeda; speaker fees and/or travel support from Astellas, Alexion, Biogen, CSL Behring, Hansa, Neovii, One Lambda, and Takeda; and institutional research payments from Biogen.
