# Supplementary material for: Prolonged CD38 targeting with felzartamab in antibody-mediated kidney transplant rejection: a biomarker-guided open-label phase 2 extension
Source: Lancet Reg Health Eur. 2026 Jul 9;68:101768. doi: 10.1016/j.lanepe.2026.101768 (PMC13379999; doi:10.1016/j.lanepe.2026.101768)
Supplement: Supplementary Figures and Tables [file mmc1.pdf]

Supplementary Appendix

Prolonged CD38 targeting with felzartamab achieves sustained suppression of antibody-mediated rejection in kidney transplant recipients: a biomarker-guided open-label phase 2 extension

Katharina A. Mayer, Matthias Diebold, Eva V. Schrezenmeier, Philip F. Halloran, Susanne Haindl, Martina Schatzl, Aylin Akifova, Daniela M. Allmer, Sabine Schranz, Nicolas Kozakowski, Johannes Kläger, Kerstin Amann, Julia Beck, Ekkehard Schütz, Maarten Naesens, Alexandre Loupy, Marc Raynaud, Irene Görzer, Hannes Vietzen, Gordon Ingle, Donna L. Flesher, Uptal D. Patel, Fabian Halleck, Irene Graf, Bernd Jilma, Klemens Budde, Georg A. Böhmig

TABLE OF CONTENTS

Supplementary Methods........ 2
- **Felzartamab administration and premedication** 2
- **Transplant biopsies**........ 2
- **Biobanking** 2
- **Antibody detection**........ 2
- **Donor-derived cell-free DNA (dd-cfDNA)**........ 2
- **Chemokine testing (peripheral blood and urine)** 2
- **NK cell monitoring** 3
- **iBox combined surrogate endpoint** 3
- **Safety monitoring and adverse event reporting**........ 3
- **List of R packages**........ 3

Supplementary Tables 5
- **Supplementary Table 1. Inclusion and exclusion criteria.** 5
- **Supplementary Table 2. Key outcomes in the intention-to-treat population.** 6
- **Supplementary Table 3. Baseline characteristics in relation to prior randomisation.** 7
- **Supplementary Table 4. Biopsy results at baseline in relation to prior randomisation.** 8
- **Supplementary Table 5. Lesion scores and molecular antibody-mediated rejection probability.*** 9
- **Supplementary Table 6. Treatment-related adverse events.**........ 10
- **Supplementary Table 7. Infusion-related reactions.**........ 11

Supplementary Figures........ 12
- **Supplementary Figure 1. Study profile.**........ 12
- **Supplementary Figure 2. Schematic overview of the study.** 13
- **Supplementary Figure 3. Immunosuppression levels and dosage.** 14
- **Supplementary Figure 4. Heat map visualisation of biomarker changes.** 15
- **Supplementary Figure 5. Molecular classifier reflecting T cell-mediated rejection.** 16
- **Supplementary Figure 6. Histologic AMR activity in relation to prior randomization.** 17
- **Supplementary Figure 7. Rejection activity in relation to prior randomisation.**........ 18
- **Supplementary Figure 8. Morphologic indices and molecular scores reflecting activity and chronicity.**19
- **Supplementary Figure 9. Effect of felzartamab on urinary chemokine levels.** 20
- **Supplementary Figure 10. Rejection biomarkers in relation to morphologic AMR activity at week 52.** 21
- **Supplementary Figure 11. IgG, IgM and IgA serum levels.** 22
- **Supplementary Figure 12. IgG subclass levels.**........ 23
- **Supplementary Figure 13. Renal function and proteinuria in relation to prior randomisation.**........ 24
- **Supplementary Figure 14. Drivers of changes in iBOX predictions over time.** Shown are the........ 25
- **Supplementary Figure 15. Safety laboratory.** 26

Trial Protocol........ 27

Supplementary Methods

Felzartamab administration and premedication

Felzartamab was diluted in 250 mL of 0·9% sodium chloride and administered intravenously over a treatment period of up to 12 months. During the fixed induction schedule, patients were scheduled to receive nine infusions on day 0, ans weeks 1, 2, 3, 4, 8, 12, 16, and 20. The first two infusions were initially administered slowly over approximately 90 minutes; following a protocol amendment in November 2024, these infusions were administered over 4 hours. Subsequent infusions were administered over 1 hour or less, with a minimum infusion time of 30 minutes. Two of the 11 patients received only eight doses, with one dose omitted in each case owing to adverse events. The fixed-dose regimen was scheduled as follows: nine doses (n=9) and eight doses (n=2). After week 20, additional felzartamab infusions were administered in response to increases in donor-derived cell-free DNA (dd-cfDNA). To prevent infusion-related reactions, patients received intravenous premedication before the first two felzartamab infusions (days 0 and 1). Intravenous premedication consisted of diphenhydramine (30 mg), paracetamol (1000 mg), and prednisolone (100 mg), each diluted in 100 mL and administered 30 minutes before felzartamab infusion. In August 2024, following a high incidence of infusion-related reactions, the premedication regimen was amended to include oral premedication, administered in addition to the intravenous regimen. This oral premedication comprised three doses of paracetamol (1000 mg each) and a single dose of famotidine (20 mg) and desloratadine (5 mg), administered within 24 hours before each of the first two felzartamab infusions.

Transplant biopsies

Biopsies were graded using Banff 2022 criteria,(1), including individual lesion scores and microvascular inflammation (MVI; glomerulitis [g] + peritubular capillaritis [ptc] sum score). To quantify histological activity and chronicity, we calculated continuous indices based on the semiquantitative Banff lesion scores recorded on kidney transplant biopsies, following the approach described by Vaulet et al. (2). Briefly, individual Banff lesion components reflecting acute inflammation (tubulitis [t], interstitial inflammation [i], intimal arteritis [v], g, and ptc) and chronic injury (chronic transplant glomerulopathy [cg], interstitial fibrosis [ci], tubular atrophy [ct], and vascular fibrous intimal thickening [cv]) were combined into composite scores using weighted sums. Activity Index=t+i+v+g+ptc+2×C4d_pos, where C4d positivity is coded as a binary variable (0 = negative, 1 = positive) to reflect its relative weight in antibody-mediated injury. The chronicity index was calculated as: 2×cg+ci+ct+cv (2). Molecular assessment used the Molecular Microscope Diagnostic System (MMDx) following established protocols (3). Molecular classifiers reflecting AMR activity (AMR_{Prob}), T cell-mediated rejection (TCMR) activity ($TCMR_{Prob}$) and overall rejection activity (Rej_{Prob}), rejection archetypes, the probability of histologic ci lesion score >1 (ci_{Prob}), and a pathogenesis-based transcript set reflecting NK cell burden (NKB), were generated using a reference set of 1,208 biopsies (3).

Biobanking

Specimens (serum, plasma, urine, peripheral blood cells) were collected and biobanked for retrospective evaluation, including HLA antibody and chemokine/cytokine detection, and flow cytometry. Aliquots were pseudonymised and frozen at −80°C or liquid nitrogen.

Antibody detection

HLA antibody testing was performed using LABscreen single-antigen flow-bead assays (One Lambda, Canoga Park, CA, USA) on a LABScan™ 200 flow analyzer (Luminex Corporation, Austin, TX, USA), as previously detailed (4). Serum samples were treated with ethylenediaminetetraacetic acid (10 mM) to prevent complement interference. Donor-specific antibodies (DSA) were assigned based on recipient and donor HLA typing and recipient serum reactivity to single-antigen beads. Results were reported as mean fluorescence intensity (MFI). For longitudinal analysis of DSA levels, bead assays were performed retrospectively in a centralised manner to preclude day-to-day variations in test results. To address occasional prozone phenomena despite EDTA pretreatment, all serum samples underwent systematic dilution. Peak MFI was then selected for each sample (a subset of tested sera exhibited peak MFI levels after 1:2 or 1:4 dilutions). Total serum IgG, IgA and IgM were measured using immunonephelometry with a BN™ II analyzer (Siemens Healthineers, Erlangen, Germany).

Donor-derived cell-free DNA (dd-cfDNA)

Peripheral blood for dd-cfDNA was collected into cell-stabilising tubes Streck Cell-Free DNA BCT tubes (La Vista, NE, USA). Relative and absolute levels of dd-cfDNA were quantified according to a previously described protocol using digital droplet PCR based on targeted SNP-detection following a previously described protocol (5, 6).

Chemokine testing (peripheral blood and urine)

For the measurement of CXCL9 and CXCL10, a Luminex-based scaled-down protocol was used as previously described (7). CXCL9 and CXCL10 were measured in urine. Undiluted samples were measured in duplicates using

Human ProcartaPlex Simplex Immunoassays (Thermo Fisher Scientific, Waltham, MA, USA). Assay results were analyzed on a LABScan™ 200 flow analyzer (Luminex Corporation). Urinary results were normalised to urinary creatinine excretion (pg[Chemokine]/mg[Creatinine]) to correct for varying urine dilutions. To account for the fact that chemokine levels can be elevated in any inflammatory milieu, such as BK- and CMV-viremia, (urinary tract) infections, or different types of rejection, data from single visits were excluded, if a CRP level ≥ 1mg/dL, a CMV-copy load ≥ 1000 copies per mL, a BK-copy load of greater than 100 copies per mL, or an ongoing infection-related adverse event was recorded.

Torque teno virus (TTV) viremia testing
Quantification of TTV DNA extracted from EDTA plasma was performed using TaqMan PCR following a previously described protocol (8). Results were recorded in copies/mL.

NK cell monitoring
Peripheral blood immune cell monitoring was performed using pre-defined assay tubes with standardised dry antibody panels using the DuraClone IM Phenotyping Basic panel (DuraClone; Beckman Coulter, Krefeld, Germany) (4). For Vienna patients, staining per the manufacturer's protocol was performed prospectively from freshly collected whole blood samples. For the majority of Berlin patients, staining per the manufacturer's protocol was performed in Berlin from freshly collected whole blood samples. Berlin samples were shipped to Vienna overnight at 4°C and analyzed on the next day. Central aquisition was performed in Vienna on a CytoFLEX LX flow cytometer (Beckman Coulter). Where prospective measurement of Berlin samples was not possible, mononuclear cells were isolated via density gradient centrifugation and stored in liquid nitrogen for retrospective analysis. Staining of mononuclear cells was performed at the Medical University of Vienna per the manufacturer's protocol. Samples were analysed on a CytoFLEX LX flow cytometer. The LIVE/DEAD TM Fixable Yellow Dead Cell Stain Kit (Invitrogen, Thermo Fisher, Waltham, MA) was used for excluding dead cells. FlowJo software (TreeStar, Version 10) was used to analyze distributions of Fc gamma receptor IIIA (CD16) expressing NK cells (defined as $CD45^{+}CD3^{-}CD56^{dim}CD16^{bright}$).

iBox combined surrogate endpoint
To predict death-censored allograft survival, we used the iBox score as an efficacy endpoint. The iBox is a validated prognostic system based on functional, histological, and immunological factors (9).

Safety monitoring and adverse event reporting
Safety assessments included interval history, physical examination, vital signs, laboratory safety panels, electrocardiograms, infection screening, and adverse event (AE) reporting. AE and serious adverse events (SAE) were coded using the Medical Dictionary for Regulatory Activities (MedDRA version 28.1). An independent Data Safety Monitoring Board participated in the decision process regarding progression to the extension study.

List of R packages
We used R version 4.4.1 (R Core Team 2024) and the following R packages: emmeans v. 1.11.2 (Lenth 2025), finalfit v. 1.0.8 (Harrison, Drake, and Pius 2024), flextable v. 0.9.9 (Gohel and Skintzos 2025), ggeffects v. 2.3.0 (Lüdecke 2018), gghalves v. 0.1.4 (Tiedemann 2022), ggpubr v. 0.6.1 (Kassambara 2025), ggrepel v. 0.9.6 (Slowikowski 2024), ggsankey v. 0.0.99999 (D. Sjoberg 2024), gtsummary v. 2.3.0 (D. D. Sjoberg et al. 2021), here v. 1.0.1 (Müller 2020), Hmisc v. 5.2.3 (Harrell Jr 2025), labelled v. 2.14.1 (Larmarange 2025), lme4 v. 1.1.37 (Bates et al. 2015), lmerTest v. 3.1.3 (Kuznetsova, Brockhoff, and Christensen 2017), officer v. 0.6.10 (Gohel, Moog, and Heckmann 2025), pheatmap v. 1.0.13 (Kolde 2025), rmcorr v. 0.7.0 (Bakdash and Marusich 2024), scales v. 1.4.0 (Wickham, Pedersen, and Seidel 2025), tidyverse v. 2.0.0 (Wickham et al. 2019).

Package citations:

Bakdash, Jonathan Z., and Laura R. Marusich. 2024. *rmcorr: Repeated Measures Correlation*. https://CRAN.R-project.org/package=rmcorr.
Bates, Douglas, Martin Mächler, Ben Bolker, and Steve Walker. 2015. "Fitting Linear Mixed-Effects Models Using lme4." *Journal of Statistical Software* 67 (1): 1–48. https://doi.org/10.18637/jss.v067.i01.
Gohel, David, Stefan Moog, and Mark Heckmann. 2025. *officer: Manipulation of Microsoft Word and PowerPoint Documents*. https://CRAN.R-project.org/package=officer.
Gohel, David, and Panagiotis Skintzos. 2025. *flextable: Functions for Tabular Reporting*. https://CRAN.R-project.org/package=flextable.
Harrell Jr, Frank E. 2025. *Hmisc: Harrell Miscellaneous*. https://CRAN.R-project.org/package=Hmisc.
Harrison, Ewen, Tom Drake, and Riinu Pius. 2024. *finalfit: Quickly Create Elegant Regression Results Tables and Plots When Modelling*. https://CRAN.R-project.org/package=finalfit.
Kassambara, Alboukadel. 2025. *ggpubr: "ggplot2" Based Publication Ready Plots*. https://CRAN.R-project.org/package=ggpubr.

Kolde, Raivo. 2025. *pheatmap: Pretty Heatmaps*. https://CRAN.R-project.org/package=pheatmap.
Kuznetsova, Alexandra, Per B. Brockhoff, and Rune H. B. Christensen. 2017. "lmerTest Package: Tests in Linear Mixed Effects Models." *Journal of Statistical Software* 82 (13): 1–26. https://doi.org/10.18637/jss.v082.i13.
Larmarange, Joseph. 2025. *labelled: Manipulating Labelled Data*. https://CRAN.R-project.org/package=labelled.
Lenth, Russell V. 2025. *emmeans: Estimated Marginal Means, Aka Least-Squares Means*. https://CRAN.R-project.org/package=emmeans.
Lüdecke, Daniel. 2018. "ggeffects: Tidy Data Frames of Marginal Effects from Regression Models." *Journal of Open Source Software* 3 (26): 772. https://doi.org/10.21105/joss.00772.
Müller, Kirill. 2020. *here: A Simpler Way to Find Your Files*. https://CRAN.R-project.org/package=here.
R Core Team. 2024. *R: A Language and Environment for Statistical Computing*. Vienna, Austria: R Foundation for Statistical Computing. https://www.R-project.org/.
Sjoberg, Daniel D., Karissa Whiting, Michael Curry, Jessica A. Lavery, and Joseph Larmarange. 2021. "Reproducible Summary Tables with the Gtsummary Package." *The R Journal* 13: 570–80. https://doi.org/10.32614/RJ-2021-053.
Sjoberg, David. 2024. *ggsankey: Sankey, Alluvial and Sankey Bump Plots*. https://github.com/davidsjoberg/ggsankey.
Slowikowski, Kamil. 2024. *ggrepel: Automatically Position Non-Overlapping Text Labels with "ggplot2"*. https://CRAN.R-project.org/package=ggrepel.
Tiedemann, Frederik. 2022. *gghalves: Compose Half-Half Plots Using Your Favourite Geoms*. https://CRAN.R-project.org/package=gghalves.
Wickham, Hadley, Mara Averick, Jennifer Bryan, Winston Chang, Lucy D'Agostino McGowan, Romain François, Garrett Grolemund, et al. 2019. "Welcome to the tidyverse." *Journal of Open Source Software* 4 (43): 1686. https://doi.org/10.21105/joss.01686.
Wickham, Hadley, Thomas Lin Pedersen, and Dana Seidel. 2025. *scales: Scale Functions for Visualization*. https://CRAN.R-project.org/package=scales.

References

1. Naesens M, Roufosse C, Haas M, Lefaucheur C, Mannon RB, Adam BA, et al. The Banff 2022 Kidney Meeting Report: Reappraisal of microvascular inflammation and the role of biopsy-based transcript diagnostics. Am J Transplant. 2024;24(3):338-49.
2. Vaulet T, Koshy P, Wellekens K, Aubert O, Bottomley C, Callemeyn J, et al. Continuous indices to assess the phenotypic spectrum of kidney transplant rejection. Nat Commun. 2025;16(1):10417.
3. Halloran PF, Madill-Thomsen KS, Reeve J. The Molecular Phenotype of Kidney Transplants: Insights From the MMDx Project. Transplantation. 2024;108(1):45-71.
4. Mayer KA, Schrezenmeier E, Diebold M, Halloran PF, Schatzl M, Schranz S, et al. A Randomized Phase 2 Trial of Felzartamab in Antibody-Mediated Rejection. N Engl J Med. 2024;391(2):122-32.
5. Beck J, Bierau S, Balzer S, Andag R, Kanzow P, Schmitz J, et al. Digital droplet PCR for rapid quantification of donor DNA in the circulation of transplant recipients as a potential universal biomarker of graft injury. Clin Chem. 2013;59(12):1732-41.
6. Oellerich M, Shipkova M, Asendorf T, Walson PD, Schauerte V, Mettenmeyer N, et al. Absolute quantification of donor-derived cell-free DNA as a marker of rejection and graft injury in kidney transplantation: Results from a prospective observational study. Am J Transplant. 2019;19(11):3087-99.
7. Mühlbacher J, Doberer K, Kozakowski N, Regele H, Camovic S, Haindl S, et al. Non-invasive Chemokine Detection: Improved Prediction of Antibody-Mediated Rejection in Donor-Specific Antibody-Positive Renal Allograft Recipients. Front Med (Lausanne). 2020;7:114.
8. Maggi F, Pifferi M, Fornai C, Andreoli E, Tempestini E, Vatteroni M, et al. TT virus in the nasal secretions of children with acute respiratory diseases: relations to viremia and disease severity. J Virol. 2003;77(4):2418-25.
9. Loupy A, Aubert O, Orandi BJ, Naesens M, Bouatou Y, Raynaud M, et al. Prediction system for risk of allograft loss in patients receiving kidney transplants: international derivation and validation study. BMJ. 2019;366:l4923.

Supplementary Tables

Supplementary Table 1. Inclusion and exclusion criteria.

**Inclusion criteria**
• Voluntary, written, informed consent<br>• Functioning allograft with an eGFR ≥15 ml/min/1.73 m^2<br>• Active or chronic active AMR in the 12-month biopsy of the randomised controlled trial phase (for patients in the felzartamab arm, provided that there was a signal regarding pharmacodynamics [NK cell depletion or donor-specific antibody reduction] and/or a 6-month biopsy showing a response to the therapy) or evidence of active AMR in an indication biopsy conducted after the completion of the initial trial phase.
**Exclusion criteria**
• Participation in another clinical trial<br>• Female subject is pregnant or lactating or not on adequate contraceptive therapy<br>• Index biopsy results:<br>*T-cell-mediated rejection classified Banff grade ≥I*<br>*De novo* or recurrent severe thrombotic microangiopathy<br>Polyoma virus nephropathy<br>*De novo* or recurrent glomerulonephritis<br>• Total bilirubin >2× ULN, alanine transaminase and aspartate aminotransferase >2·5×ULN<br>• Haemoglobin <8 g/dL<br>• Thrombocytopenia: Platelets <100 G/L<br>• Leukopenia: Leukocytes <3 G/L<br>• Neutropenia: Neutrophils < 1.5 G/L<br>• Active viral, bacterial or fungal infection precluding intensified immunosuppression<br>• Active malignant disease precluding intensified immunosuppressive therapy<br>• Latent or active tuberculosis<br>• Administration of a live vaccine within 6 weeks of screening<br>• History of alcohol or illicit substance abuse<br>• Serious medical or psychiatric illness likely to interfere with participation in the study

AMR=antibody-mediated rejection. eGFR=estimated glomerular filtration rate. NK cell=natural killer cell. ULN=upper limit of normal.

Supplementary Table 2. Key outcomes in the intention-to-treat population.

Efficacy outcomes

Weeks 24 and 52

Morphology (Banff 2022)

- Resolution of AMR activity (chronic [inactive] AMR or no rejection after treatment); at week 24 and at week 52
- Level of microvascular inflammation (MVI; score 0–6) (glomerulitis [g] plus peritubular capillaritis [ptc]); at week 24 and at week 52
- Resolution of MVI to a 0 score (versus a score of 1–6); at week 24 and at week 52
- Molecular classifier for AMR (range of score: 0.0–1.0); at week 24 and at week 52
- AMR category

MMDx analysis

- AMR-related classifier (AMR_{Prob})
- Molecular classifiers related to 'all rejection' (Rej_{Prob}; range of score: 0.0–1.0) and TCMR ($TCMR_{Prob}$; range of score: 0.0–1.0)
- Molecular transcript sets reflecting acute (IRRAT) and chronic injury (ciprob)
- Molecular rejection archetypes
- Molecular pathogenesis-based transcript set reflecting NK cell burden (NKB)

Day 0, weeks 12, 24 and 52

- Peripheral blood NK cells
- TTV load

Day 0, weeks 4, 8, 12, 16, 20, 24, 32, 40, and 52

- Total Ig classes (IgG, IgA, and IgM)
- IgG subclasses (IgG1, 2, 3, and 4)

Every visit

- dd-cfDNA
- Immunodominant DSA-MFI
- CXCL9 and CXCL10 levels in urine
- Slope of eGFR (Chronic Kidney Disease Epidemiology Collaboration [CKD-EPI] equation, 2021)
- Protein/creatinine ratio in spot urine

Safety outcomes*

Every visit

- Safety laboratory

Day 0 through week 52

- AE, overall
  Relationship
  - Treatment-related AE
  - Treatment-emergent AE

  Infusion Severity
  - Mild AE
  - Moderate AE
  - Severe AE
- Infusion-related reactions, overall
- Serious AE

*AE were summarised by System Organ Class (SOC) and Preferred Term (PT). AE=adverse event. AMR=antibody-mediated rejection. CXCL= C-X-C motif chemokine ligand. dd-cfDNA= Donor-derived cell-free DNA. DSA=donor-specific antibody. eGFR=estimated glomerular filtration rate. MFI=mean fluorescence intensity. MVI=microvascular inflammation. NK cell=natural killer cell. TTV=Torque Teno virus.

Supplementary Table 3. Baseline characteristics in relation to prior randomisation.

Variables	Placebo (n= 4)	Felzartamab (n=7)
**Recorded at transplantation**		
Female sex	2 (50·0)	2 (28·6)
Recipient age, years	48 (38–56)	36 (30–48)
Race – no. (%)*		
White	4 (100·0)	5 (71·4)
Asian	0 (0·0)	2 (28·6)
Living donor	1 (25·0)	3 (42·9)
Donor age, years	32 (27–38)	48 (40–52)
Prior kidney transplant	2 (50·0)	1 (14·3)
HLA (A, B, DR) mismatch	3 (3–3)	3 (2–4)
Cold ischaemia time, hr	10 (8 –14)	10 (3–11)
CDC panel reactivity ≥10%	0 (0·0)	0 (0·0)
Missing data, no.	1 (25·0)	1 (14·3)
Preformed anti-HLA DSA†	1 (25·0)	2 (28·6)
Missing data	2 (50·0)	3 (42·9)
**Recorded at inclusion in the extension study**		
Age at inclusion, years	58 (53–65)	49 (40–56)
Time from transplantation to inclusion, years	14 (6–21)	10 (5–16)
eGFR, mL/min/1·73 m^2	34 (26–53)	39 (33–57)
Protein/creatinine ratio, mg/g	952 (302–1536)	539 (312–932)
Triple immunosuppression	3 (75·0)	7 (100·0)
Tacrolimus-based immunosuppression	2 (50·0)	7 (100·0)
DSA characteristics		
Number of DSA	2 (1–2)	2 (1–3)
MFI of the immunodominant DSA	2422 (1414–3160)	5761 (2610–7309)

*Race and ethnicity were reported by patients. †Pre-transplant DSA data were available for 6 recipients (solid-phase HLA antibody screening on the wait list was implemented at the Vienna transplant unit in July 2009). Data are n (%) or median (IQR). AMR=antibody-mediated rejection; CDC=complement-dependent cytotoxicity; DSA=donor-specific antibody; eGFR=estimated glomerular filtration rate; HLA=human leukocyte antigen; IQR=interquartile range; MFI=mean fluorescence intensity; NK cell=natural killer cell.

Supplementary Table 4. Biopsy results at baseline in relation to prior randomisation.

Variable	All patients (n=11)	Placebo (n= 4)	Felzartamab (n=7)
**Morphologic results**			
Banff 2022 phenotypes			
C4d-negative active AMR	3 (27)	1 (25)	2 (29)
C4d-negative chronic active AMR	2 (18)	1 (25)	1 (14)
C4d-positive chronic active AMR	4 (36)	1 (25)	3 (43)
Probable active AMR	1 (9·1)	1 (25)	0 (0)
Probable chronic active AMR	1 (9·1)	0 (0)	1 (14)
Single lesion scores			
Glomerulitis (g)	1 (0–1)	1 (0–2)	1 (0–1)
Peritubular capillaritis (ptc)	1 (1–2)	2 (1–2)	1 (1–2)
MVI; g + ptc	2 (2–2)	2 (2–3)	2 (2–2)
Intimal arteritis (v)	0 (0–0)	0 (0–0)	0 (0–0)
Glomerular double contours (cg)	1 (0–2)	1 (0–3)	1 (0–2)
C4d in peritubular capillaries	0 (0–1)	0 (0–1)	0 (0–1)
Interstitial fibrosis (ci)	1 (1–2)	1 (1–1)	2 (1–2)
Tubular atrophy (ct)	1 (1–2)	1 (1–2)	1 (1–2)
Vascular fibrous intimal thickening (cv)	1 (1–2)	1 (1–2)	1 (1–2)
**Molecular results**			
Most probable molecular archetype			
Early-stage AMR	2 (18)	0	2 (29)
Fully-developed AMR	8 (73)	3 (75)	5 (71)
Late-stage AMR	1 (9·1)	1 (25)	0 (0)
Molecular rejection-associated scores			
AMR probability (AMR_{Prob})	0·77 (0·58–0·87)	0·72 (0·41–0·88)	0·77 (0·64–0·83)
TCMR probability ($TCMR_{Prob}$)	0·01 (0·01–0·02)	0·01 (0·01–0·04)	0·01 (0·01–0·02)
Rejection probability (Rej_{Prob})	0·65 (0·45–0·86)	0·59 (0·38–0·86)	0·65 (0·55–0·86)
ciprob	0·56 (0·28–0·77)	0·58 (0·34–0·68)	0·36 (0·28–0·87)
NK cell transcript burden (NKB)	1·42 (0·94–1·52)	1·17 (0·74–1·59)	1·42 (1·02–1·47)

Data are n (%) or median (IQR). AMR=antibody-mediated rejection. MVI=microvascular inflammation. NK cell=natural killer cell. TCMR=T cell-mediated rejection.

Supplementary Table 5. Lesion scores and molecular antibody-mediated rejection probability.*

Patient ID	Glomerulitis (g)			Peritubular capillaritis (ptc)			Intimal arteritis (v)			C4d score (c4d)			Glomerular double contours (cg)			AMR_{Prob}		
	Bx1	Bx2	Bx3	Bx1	Bx2	Bx3	Bx1	Bx2	Bx3	Bx1	Bx2	Bx3	Bx1	Bx2	Bx3	Bx1	Bx2	Bx3
1	1	1	0	2	0	0	0	0	0	0	0	0	1	0	-[†]	0·83	0·22	0·15
2	1	1	0	1	0	0	0	0	0	0	0	0	3	2	3	0·24	0·06	0·03
3	1	1	0	1	1	0	0	0	0	0	0	0	0	0	0	0·64	0·19	0·26
4	2	1	1	0	0	0	0	0	0	2	0	2	2	3	1	0·77	0·07	0·09
5	1	0	1	1	2	1	0	0	0	1	2	0	1	1	0	0·91	0·74	0·89
6	0	0	0	2	0	0	0	0	0	0	2	3	0	0	0	0·89	0·12	0·08
7	0	0	0	2	0	0	0	0	1	0	3	3	0	0	1	0·83	0·29	0·99
8	0	0	1	1	0	2	0	0	0	0	0	1	1	1	3	0·64	0·14	0·12
9	1	2	0	1	1	0	0	0	0	1	1	0	2	2	3	0·57	0·05	0·04
10	0	0	0	1	0	0	0	0	0	0	0	0	0	0	0	0·87	0·04	0·09
11	2	2	2	2	2	2	0	0	0	2	0	0	2	2	2	0·58	0·43	0·35

*Shown are results of baseline (Bx1), 24-week (Bx2) and 52-week (Bx3) biopies. [†]Because of severe collapse of capillary loops in parts of the specimen, reliable scoring of glomerular capillary double contours was not possible. AMR_{Prob}=antibody-mediated rejection probability. Bx=biopsy.

Supplementary Table 6. Treatment-related adverse events.

Adverse events*	N = 11
**General disorders and administration site conditions**	**9 (82)**
Infusion-related reaction	9 (82)
Fatigue	2 (18)
Oedema peripheral	2 (18)
Chest pain	1 (9·1)
**Infections and infestations**	**9 (82)**
Nasopharyngitis	7 (64)
Pneumonia	3 (27)
Cytomegalovirus viremia	2 (18)
COVID19	1 (9·1)
Gastroenteritis	1 (9·1)
Infection†	1 (9·1)
Influenza virus infection	1 (9·1)
Norovirus Infection	1 (9·1)
Urinary tract infection	1 (9·1)
**Gastrointestinal disorders**	**4 (36)**
Diarrhoea	3 (27)
Abdominal pain	2 (18)
Sapovirus Gastroenteritis	1 (9·1)
Gastrointestinal haemorrhage	1 (9·1)
Nausea	1 (9·1)
Vomiting	1 (9·1)
**Metabolism and nutrition disorders**	**3 (27)**
Hypocalcemia	2 (18)
Hyponatremia	2 (18)
Decreased appetite	1 (9·1)
Hypercalcaemia	1 (9·1)
Iron deficiency	1 (9·1)
**Musculoskeletal and connective tissue disorders**	**3 (27)**
Myalgia	2 (18)
Bone pain	1 (9·1)
**Respiratory, thoracic and mediastinal disorders**	**3 (27)**
Epistaxis	1 (9·1)
Rhinitis	1 (9·1)
Allergic rhinitis	1 (9·1)
**Skin and subcutaneous tissue disorders**	**3 (27)**
Pruritus	2 (18)
Basal cell carcinoma	1 (9·1)
Skin cancer	1 (9·1)
Tattoo skin reaction	1 (9·1)
**Injury poisoning and procedural complications**	**2 (18)**
Animal bite	2 (18)
Ankle fracture	1 (9·1)
Ligament sprain	1 (9·1)
**Nervous system disorders**	**2 (18)**
Headache	1 (9·1)
Sciatica	1 (9·1)
**Renal and urinary disorders**	**2 (18)**
Acute kidney injury	2 (18)
**Reproductive system and breast disorders**	**2 (18)**
Intermenstrual bleeding	1 (9·1)
Cervical dysplasia	1 (9·1)
Vulvovaginal dryness	1 (9·1)
**Vascular disorders**	2 (18)
Hypertension	2 (18)
**Blood and lymphatic system disorders**	**1 (9·1)**
Nephrogenic anemia	1 (9·1)
**Cardiac disorders**	1 (9·1)
Atrial fibrillation	1 (9·1)
Dyspnoea exertional	1 (9·1)
Myocardial infarction	1 (9·1)
**Endocrine disorders**	**1 (9·1)**
Thyroid mass	1 (9·1)
**Neoplasms benign malignant and unspecified**	**1 (9·1)**
Cervix carcinoma	1 (9·1)
**Surgical and medical procedures**	**1 (9·1)**
Tooth extraction	1 (9·1)

Data are n (%). *Adverse events were summarised by System Organ Class (SOC; bold text) and Preferred Term (PT; non-bold text). †One adverse event, a transient elevation of C-reactive protein of unclear cause, was documented as an infection (primary term) without further specification.

Supplementary Table 7. Infusion-related reactions.

Patient ID	Visit No.	Severity*	Symptoms	Felzartamab Infusion	Treatment
1	Visit 1	Mild	Sore throat	No action	None
	Visit 13	Moderate	Clogged nose, sore throat, bone pain, face exanthema	No action	Metamizol PO (500 mg), once
2	Visit 1	Moderate	Dyspnea, abdominalgia	No action	Metamizol PO 500 mg, 2x/day, 4 days
	Visit 2	Moderate	Abdominalgia	No action	Metamizol PO (500 mg), once
	Visit 13	Moderate	Abdominalgia	No action	Metamizol PO 500 mg, 2x/day, 4 days
	Visit 16	Moderate	Clogged nose, dyspnea, diarrhea, abdominalgia	No action	Metamizol PO 1000 mg, 3x/day, 2 days
3	Visit 1	Mild	Coughing, clogged nose	No action	None
	Visit 14	Moderate	Flush, cough, clogged nose	No action	Diphenhydramine 30 mg IV, once; paracetamol 1000 mg IV, once
4	Visit 1	Mild	Clogged nose, chest tightness	No action	None
	Visit 12	Moderate	Nausea, flush, dry cough and vomiting	No action	Ondansetron 4 mg PO, once
	Visit 15	Mild	Nausea, clogged nose, mild chest tightness, feeling	No action	None
5	Visit 3	Mild	Clogged nose	No action	None
7	Visit 1	Mild	Dyspnea, coughing	No action	None
	Visit 16	Moderate	Dyspnea, clogged nose	No action	None
8	Visit 1	Moderate	Dry cough, sore throat, nausea	No action	None
10	Visit 1	Mild	Vomiting	No action	None
	Visit 14	Moderate	Flush, hypertension thoracic pain, back pain	Infusion transiently stopped, and reinstituted	Prednisolone 150 mg IV, once Dimetinden 4 mg IV, once
11	Visit 1	Mild	Rhinitis, cough	No action	Nasal spray

*IRR severity was graded by the attending investigator. IRR=infusion-related reaction.

Supplementary Figures

Supplementary Figure 1. Study profile. Twenty-one adult kidney transplant recipients who had participated in the phase 2 randomised controlled trial of felzartamab were screened for eligibility for the extension trial, with 11 patients meeting the eligibility criteria. Two of those had no evidence of antibody-mediated rejection (AMR) activity at the end of the parent trial; however, re-screening biopsies revealed recurrent rejection activity. Included patients received felzartamab for 52 weeks, with donor-derived cell-free DNA-guided treatment after 20 weeks of prescheduled therapy. AMR=antibody-mediated rejection. eGFR=estimated glomerular filtration rat. IIT=intention-to-treat. Created with Biorender.com.

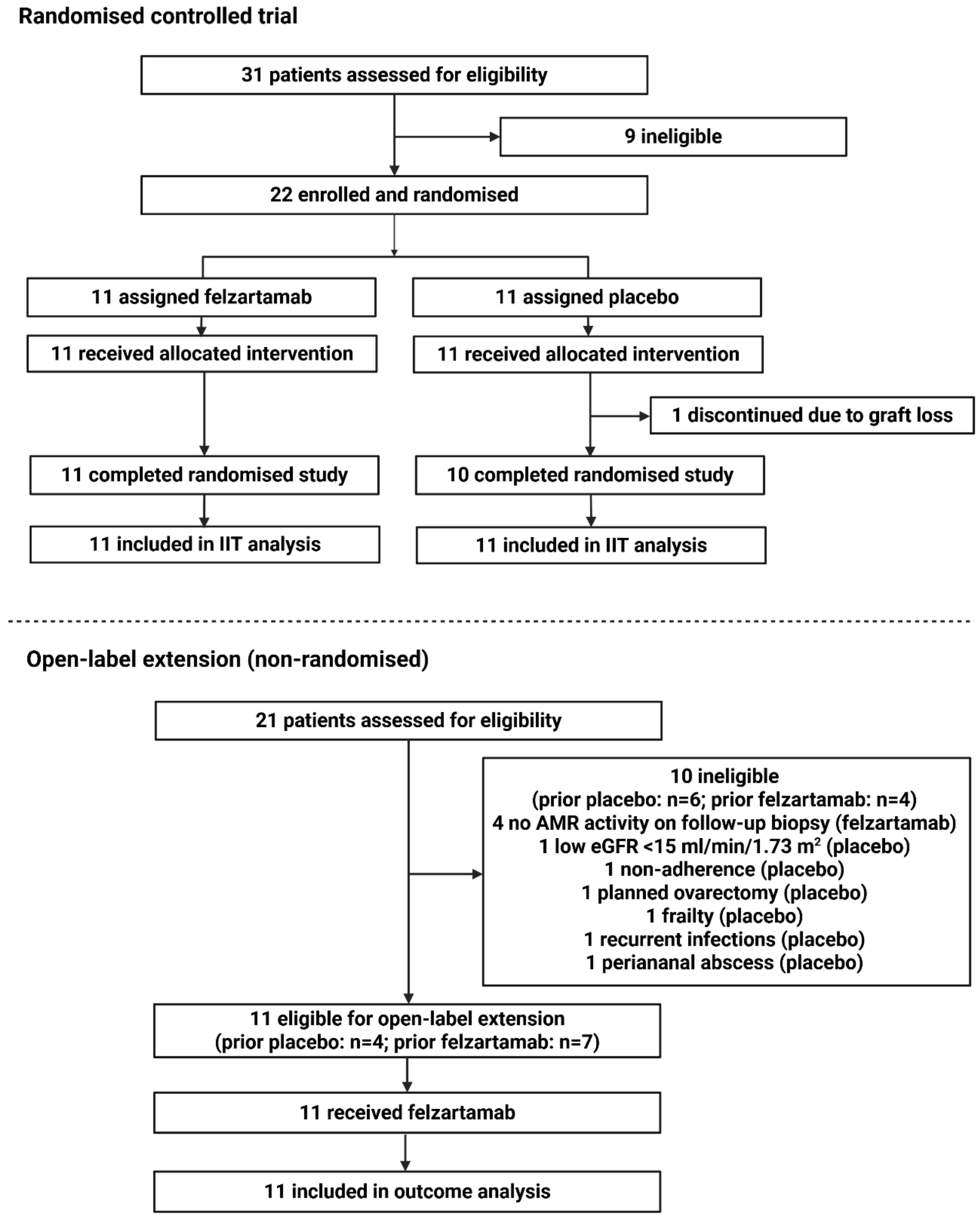

Supplementary Figure 2. Schematic overview of the study. Shown are infusion schedules in individual patients, in relation to trial visits (V1-17). During the fixed-dosing period, two infusions were omitted because of adverse events (patient 3 and 8). Infusions are shown as blue bars, and, if associated with infusion-related reactions as orange (mild) or red (moderate) bars. AMR=antibody-mediated rejection. dd-cfDNA=donor-derived cell-free DNA. DSA=donor-specific antibody. eGFR,=estimated glomerular filtration rate. NK cell=natural killer cell. TTV=Torque Teno virus. Created with Biorender.com.

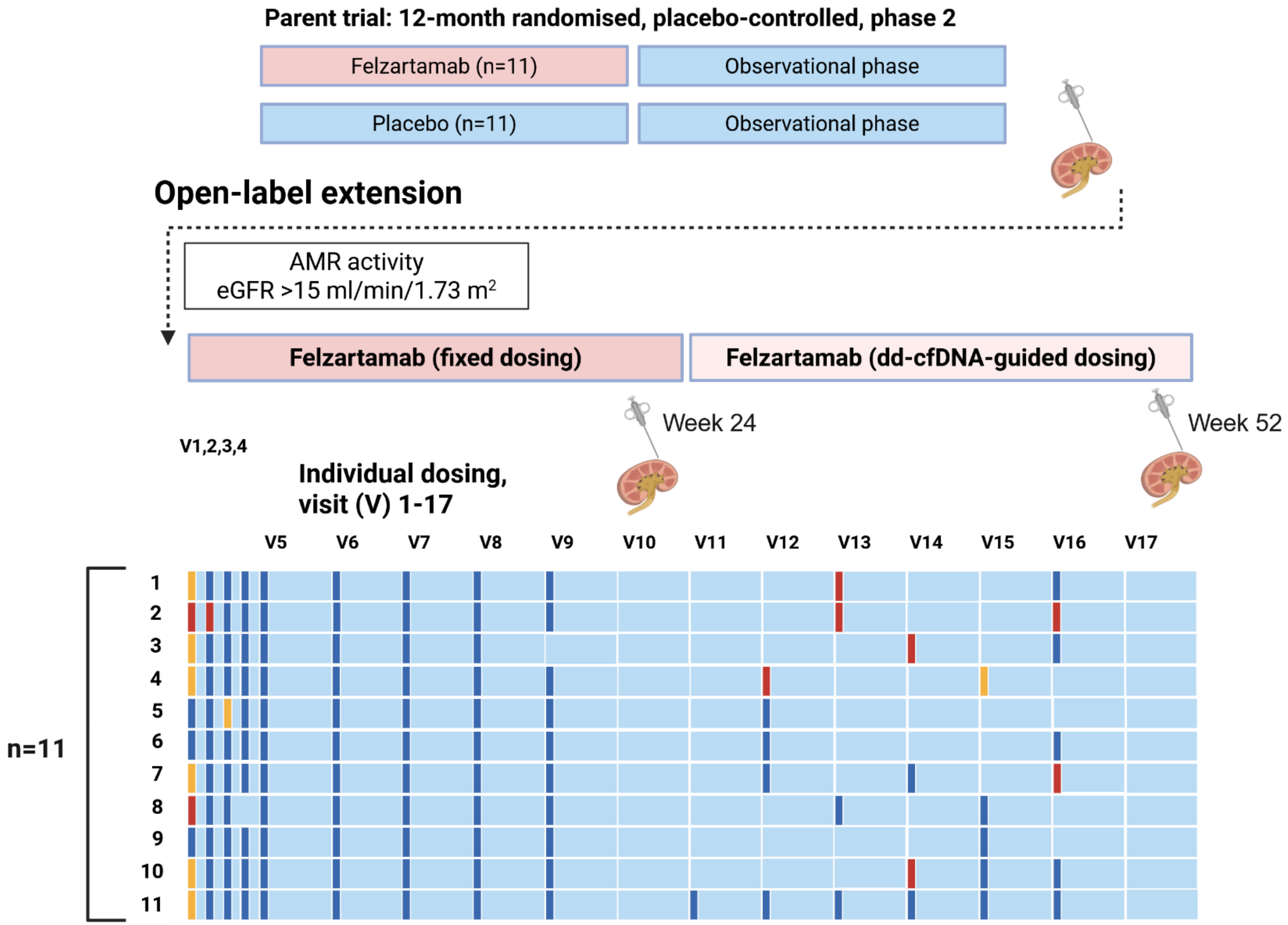

Supplementary Figure 3. Immunosuppression levels and dosage. Panel A shows trough levels of tacrolimus (n=9) and cyclosporin A (n=1). One patient was on MMF and steroids, without a calcineurin inhibitor. Panel B shows dosages of mycophenolate mofetil (n=9) and enteric-coated mycophenolic acid (n=2).

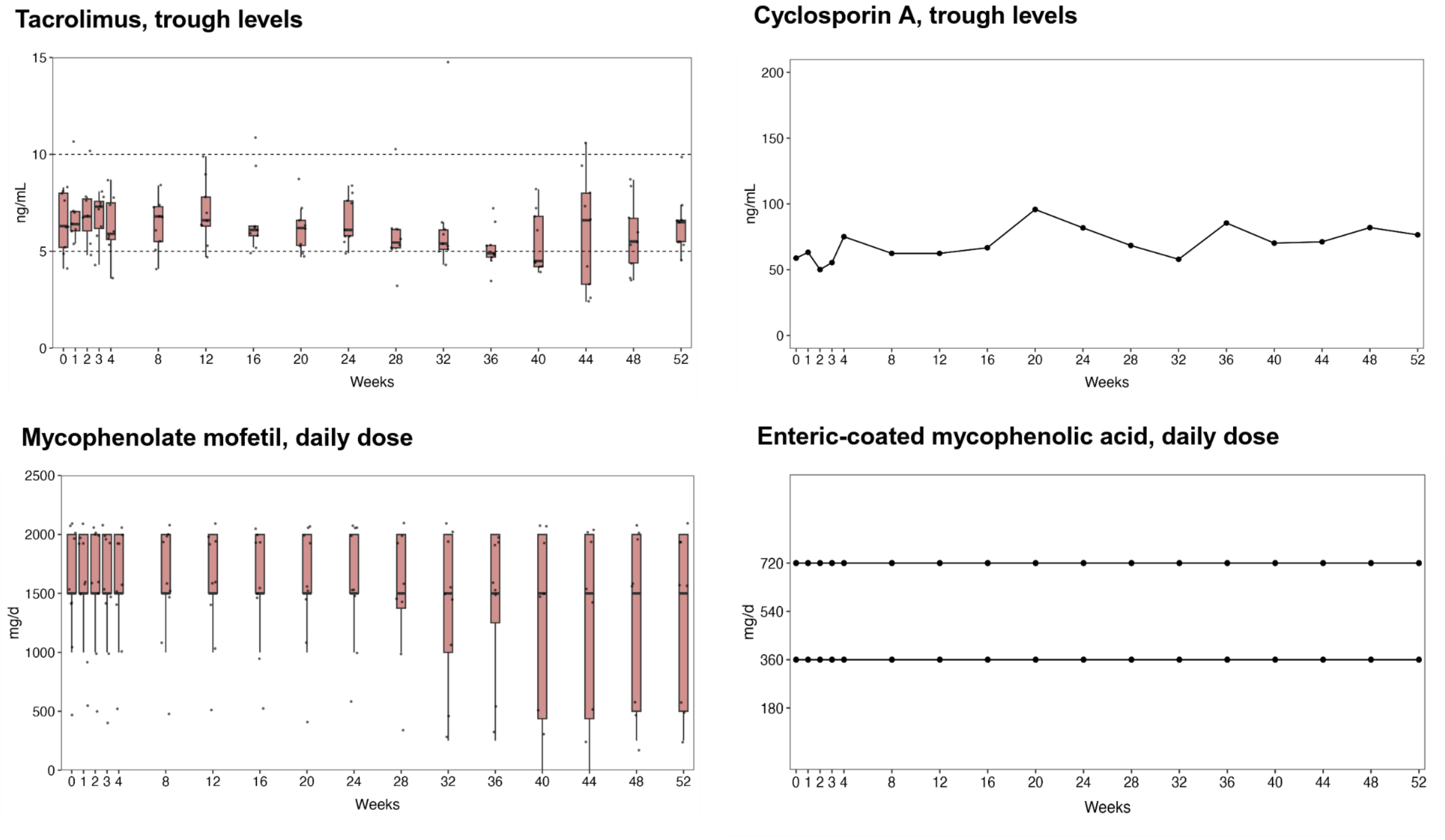

Supplementary Figure 4. Heat map visualisation of biomarker changes. Percent changes in biopsy-based biomarkers (microvascular inflammation [MVI] score, molecular AMR_{Prob} score, molecular pathogenesis-based transcript score for natural killer [NK] cell burden [NKB]) and peripheral blood biomarkers (cell-free DNA [dd-cfDNA] concentration, immunodominant donor-specific antibody [DSA] mean fluorescence intensity, and NK cell count) are shown for individual trial participants (patients 1–11). For each participant, percent changes at the two follow-up biopsies relative to baseline are shown, with varying intensities of blue indicating decreases and red indicating increases.

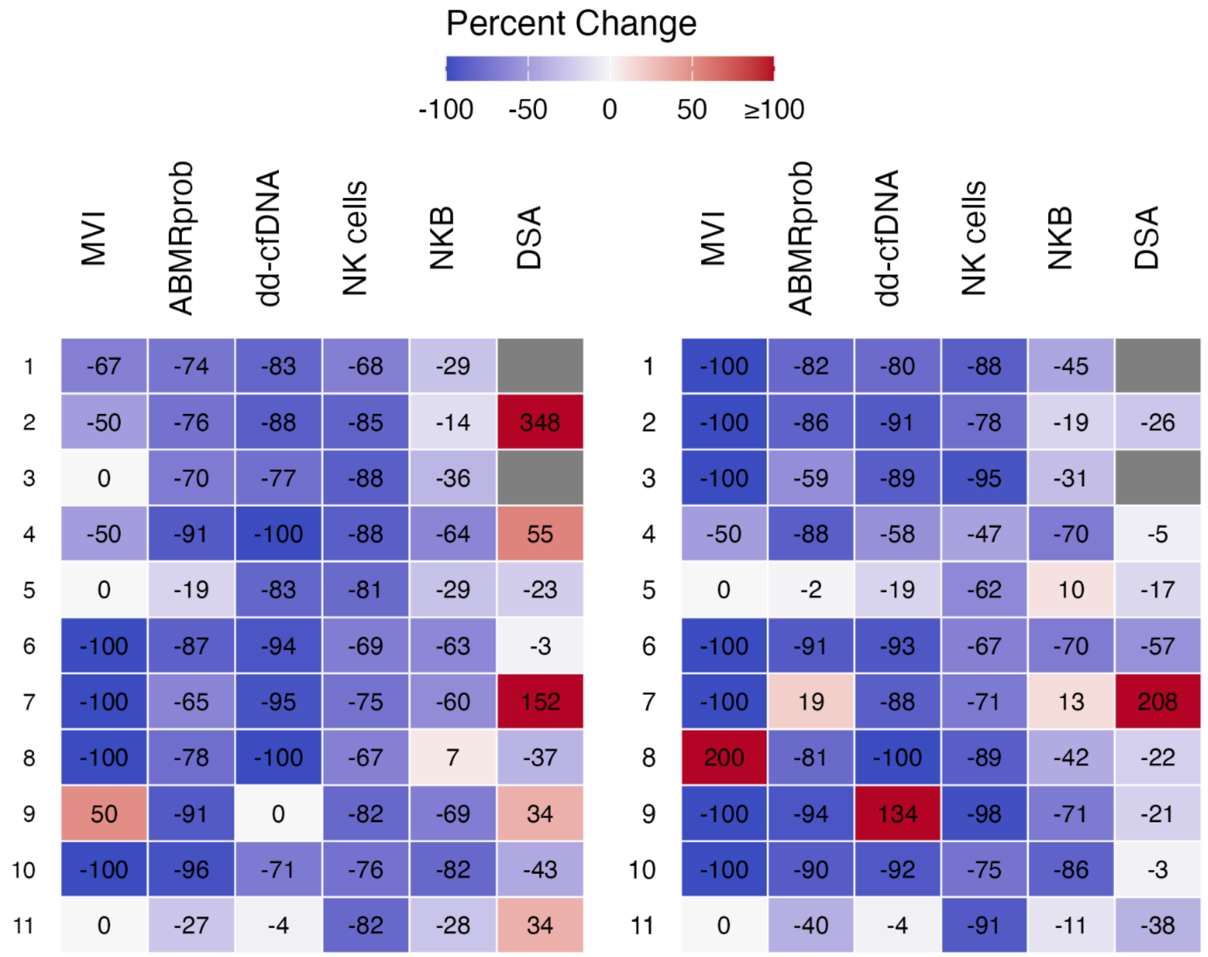

Supplementary Figure 5. Molecular classifier reflecting T cell-mediated rejection. The panel shows the course of a molecular T cell-mediated rejection probability score shown for baseline biopsies and follow-up biopsies at weeks 24 and 52. Shaded areas represent group-wise density estimates. Solid black circles indicate median values, and dashed lines depict changes in median scores over time. Solid coloured circles represent individual patient values, and solid coloured lines indicate within-patient changes; green indicates decrease and red indicates increase in scores.

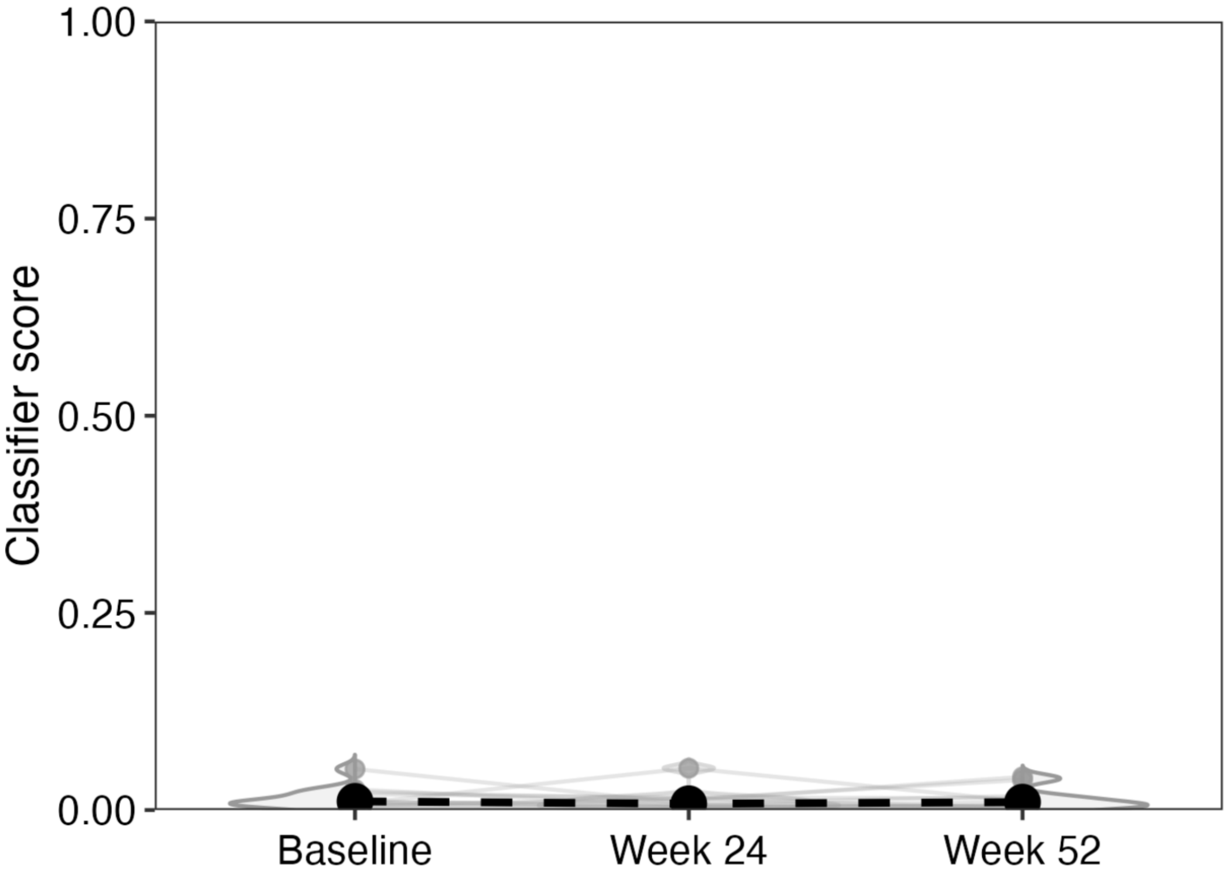

Supplementary Figure 6. Histologic AMR activity in relation to prior randomization. Shown are Sankey plots depicting changes in morphologic antibody-mediated rejection (AMR) phenotypes (active phenotypes: probable or [chronic] active AMR; phenotypes with no activity: chronic inactive AMR or no rejection) across renal allograft biopsies obtained at baseline, week 24 (fixed dosing), and week 52 (dd-cfDNA–guided dosing). Vertical stacks represent biopsy time points and the number of biopsies performed. Numbers identify individual patients. Grey bands illustrate transitions in biopsy phenotypes over time, with band width proportional to the number of cases.

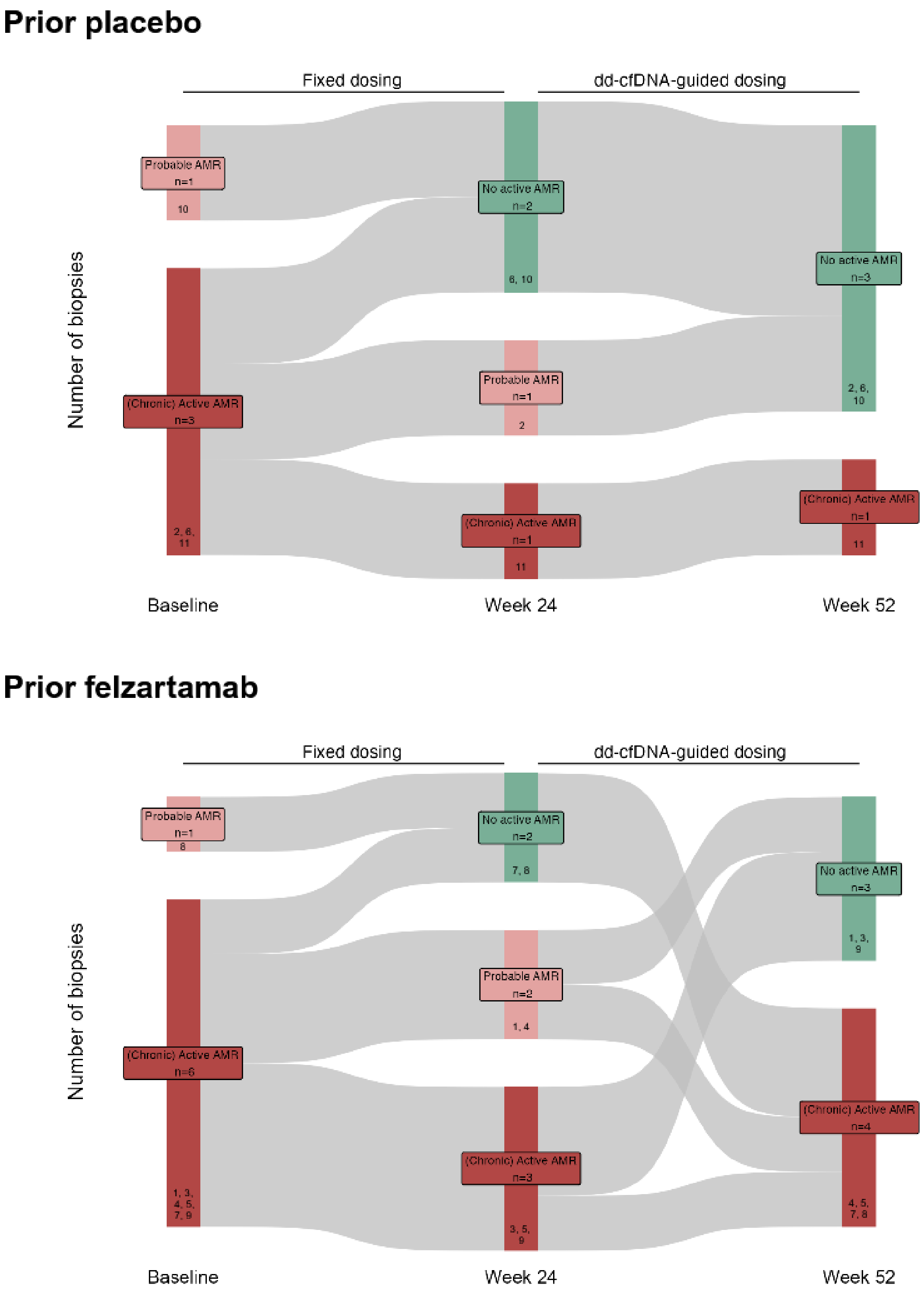

Supplementary Figure 7. Rejection activity in relation to prior randomisation. Shown are individual trajectories and median changes from baseline to weeks 24 and 52 in microvascular inflammation (MVI) and molecular antibody-mediated rejection (AMR) probability scores, respectively, in felzartamab versus placebo pre-treated participants. Shaded areas represent group-wise density estimates. Solid black circles indicate median values, and dashed lines depict changes in median scores over time. Solid coloured circles represent individual patient values, and solid coloured lines indicate within-patient changes green indicates decrease and red indicates increase in scores. Numbers identify selected patients of interest.

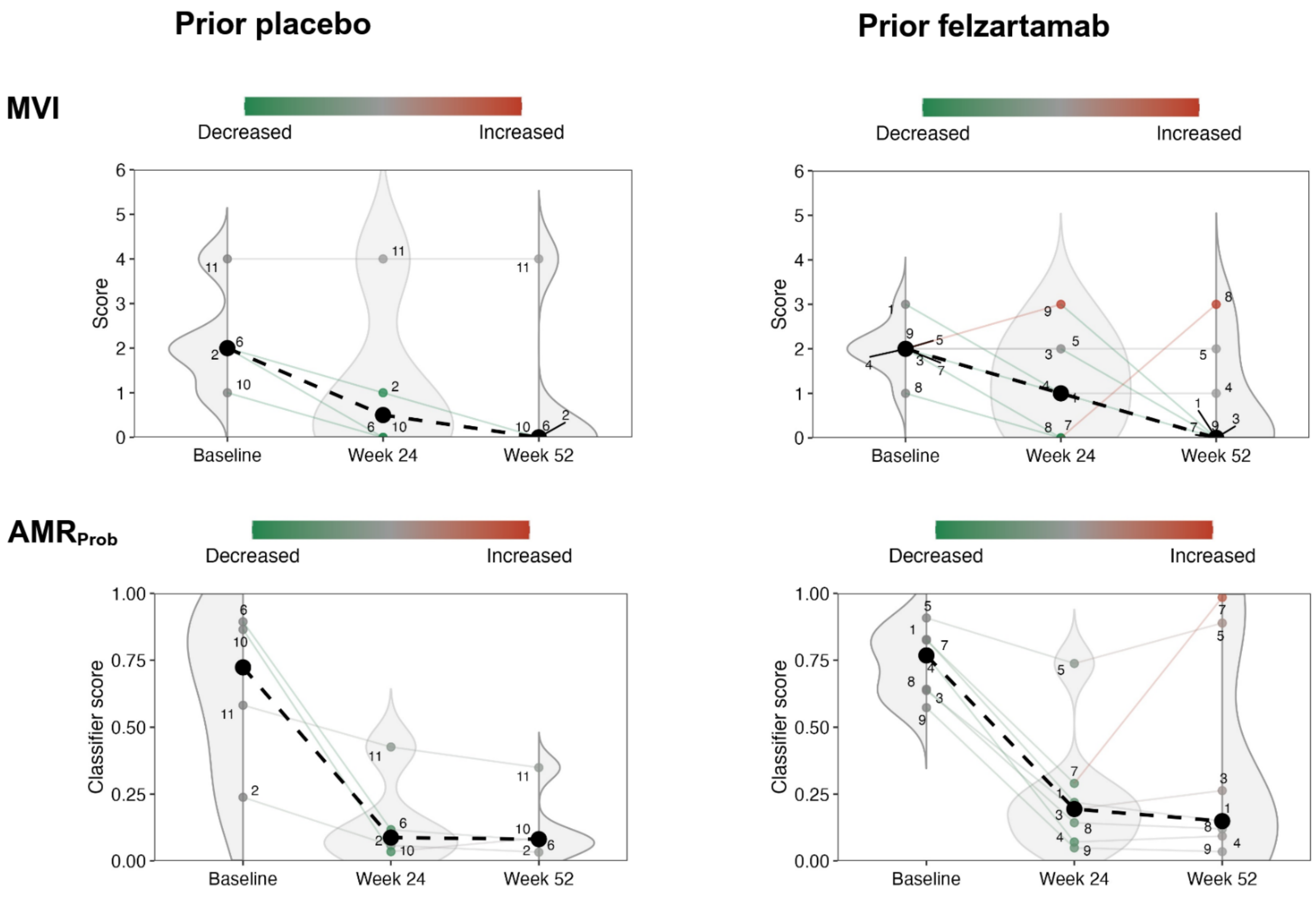

Supplementary Figure 8. Morphologic indices and molecular scores reflecting activity and chronicity. Shaded areas represent group-wise density estimates. Solid black circles indicate median values, and dashed lines depict changes in median scores over time. Solid coloured circles represent individual patient values, and solid coloured lines indicate within-patient changes; green indicates decrease and red indicates increase in scores. Numbers identify selected patients of interest.

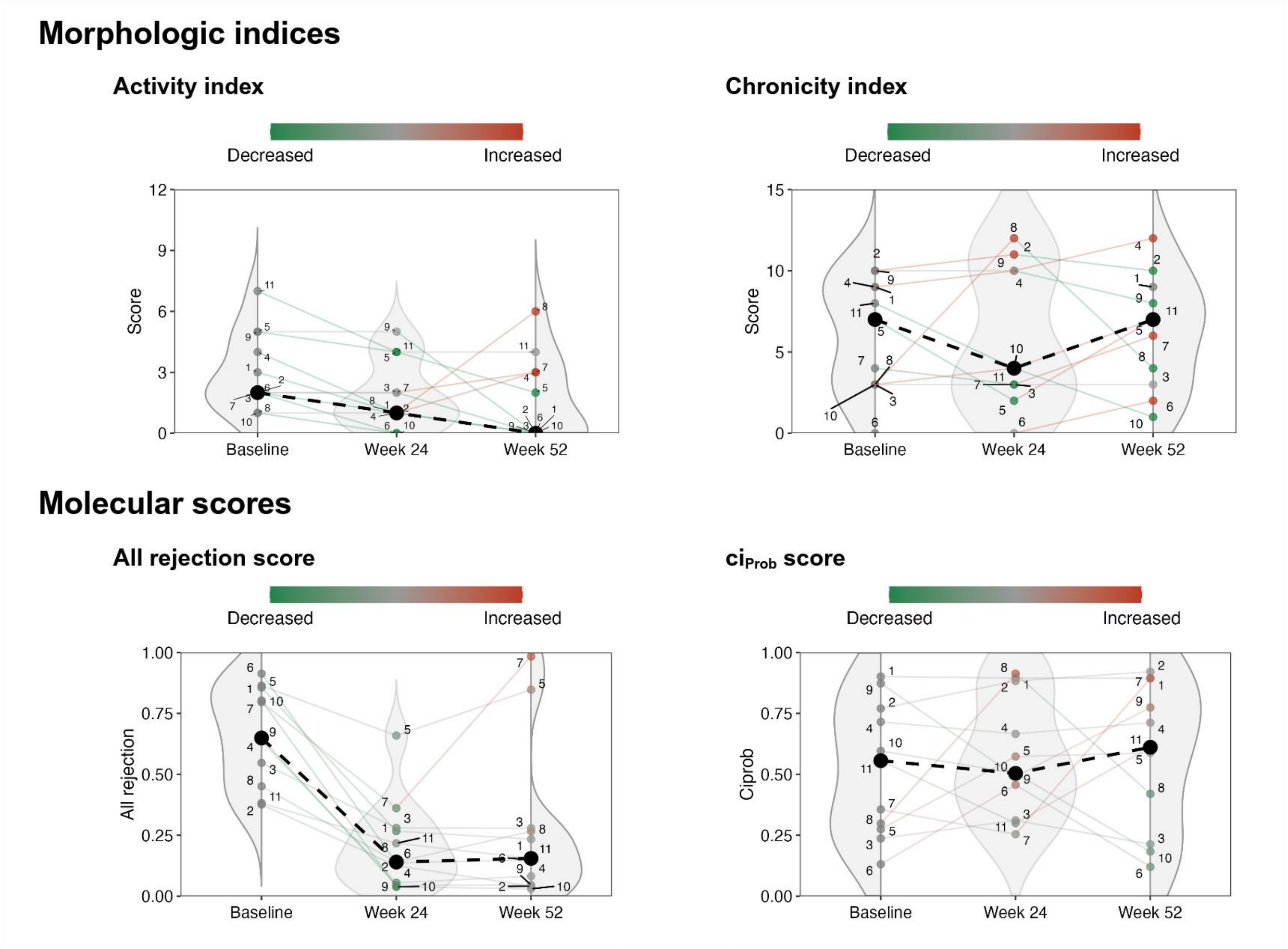

Supplementary Figure 9. Effect of felzartamab on urinary chemokine levels. Shown are urinary C–X–C motif chemokine ligands 9 and 10 (CXCL9 and CXCL10) normalised to urinary creatinine excretion at baseline and at scheduled follow-up visits up to 52 weeks. The horizontal line in each box represent the median, the tops and bottoms of the boxes represent the upper and lower limits of the interquartile range, and the I bars represent 1.5 times the interquartile range. Individual dots represent measurements from individual patients.

CXCL9

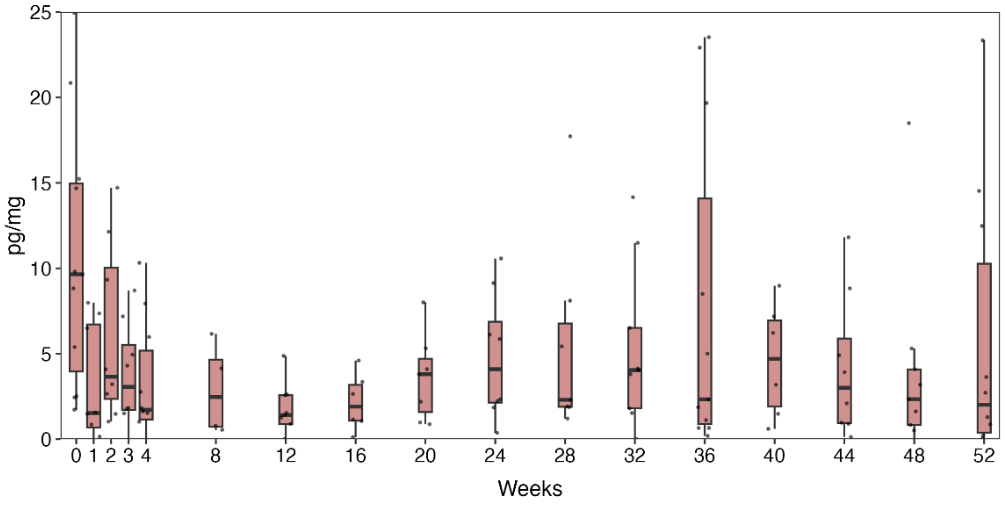

CXCL10

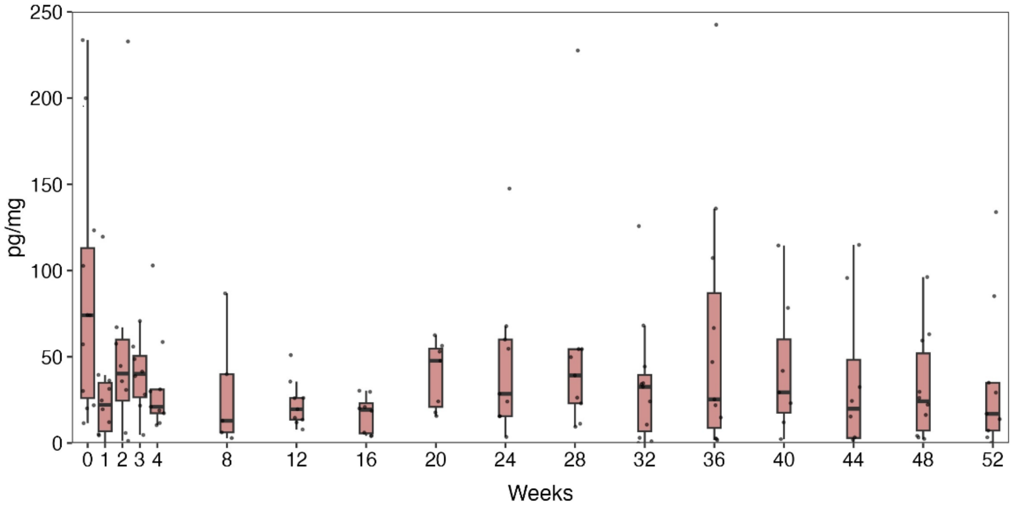

Supplementary Figure 10. Rejection biomarkers in relation to morphologic AMR activity at week 52. Absolute an relative donor-derived cell-free DNA, urinary C–X–C motif chemokine ligands 9 and 10 (CXCL9 and CXCL10) normalised to urinary creatinine excretion, $CD56^{dim}CD16^{bright}$NK cell counts and a molecular pathogenesis-based transcript set reflecting NK cell burden (NKB) are shown in relation to AMR activity (according to the Banff 2022 scheme) in 52-week biopsies. Individual dots represent measurements from individual patients. Horizontal lines represent the median.

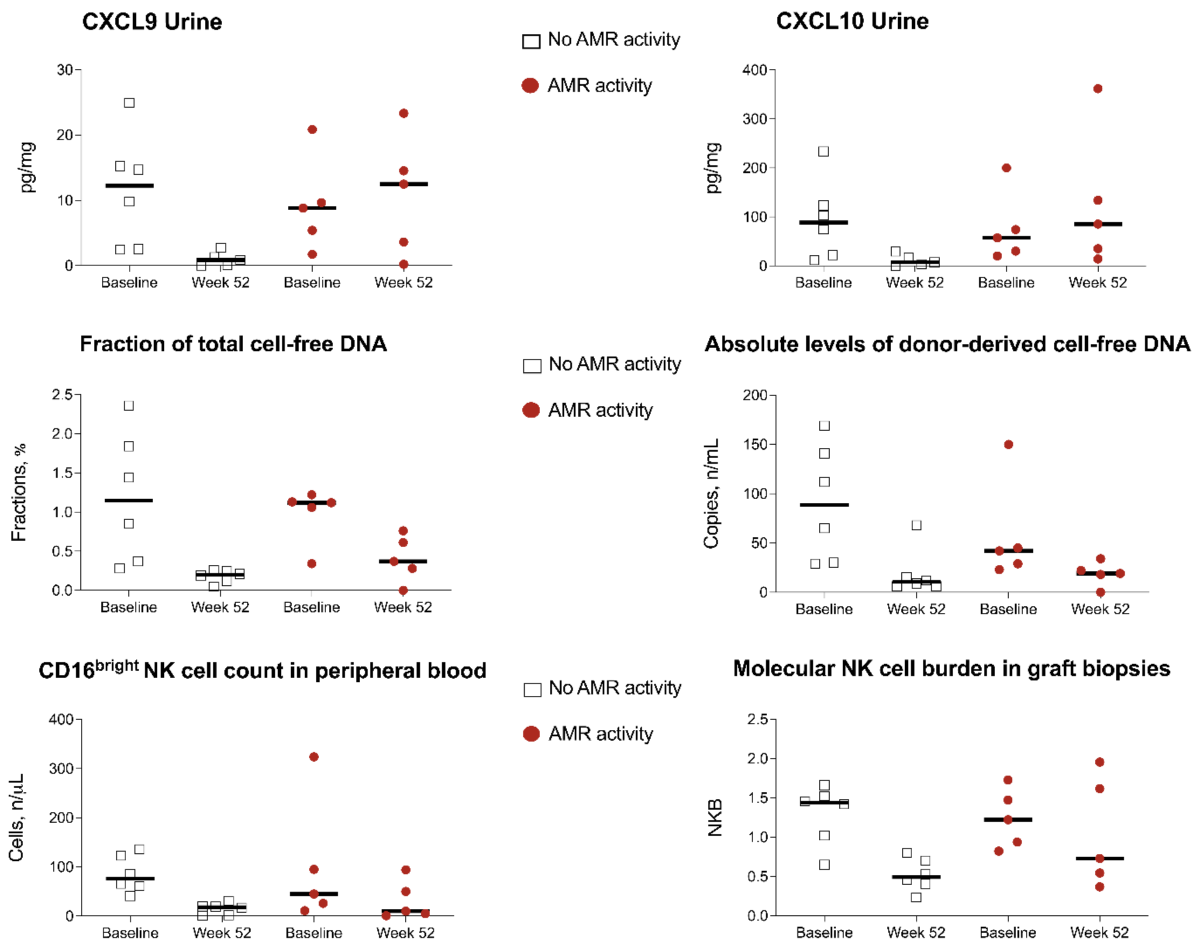

Supplementary Figure 11. IgG, IgM and IgA serum levels. The line plots show medians and interquartile range (IQR). Median absolute baseline levels (mg/dL): IgG, 763 (IQR 588-1030); IgM, 69 (IQR 38-95); IgA, 134 (IQR 100-208).

IgG

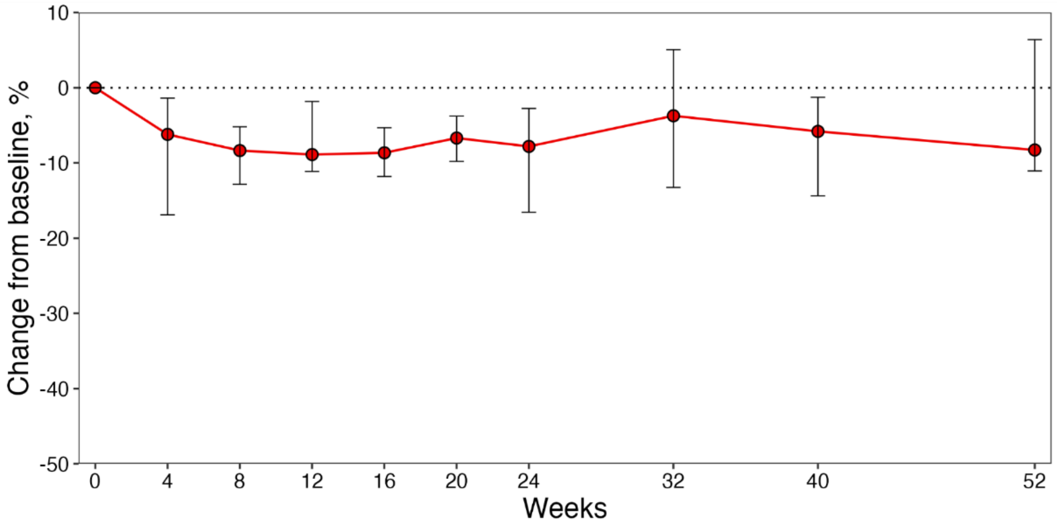

IgM

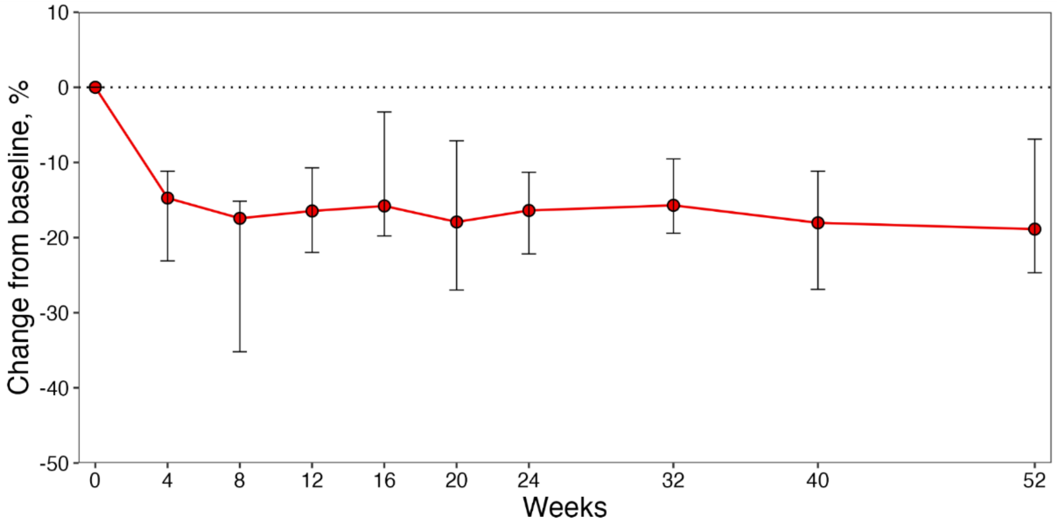

IgA

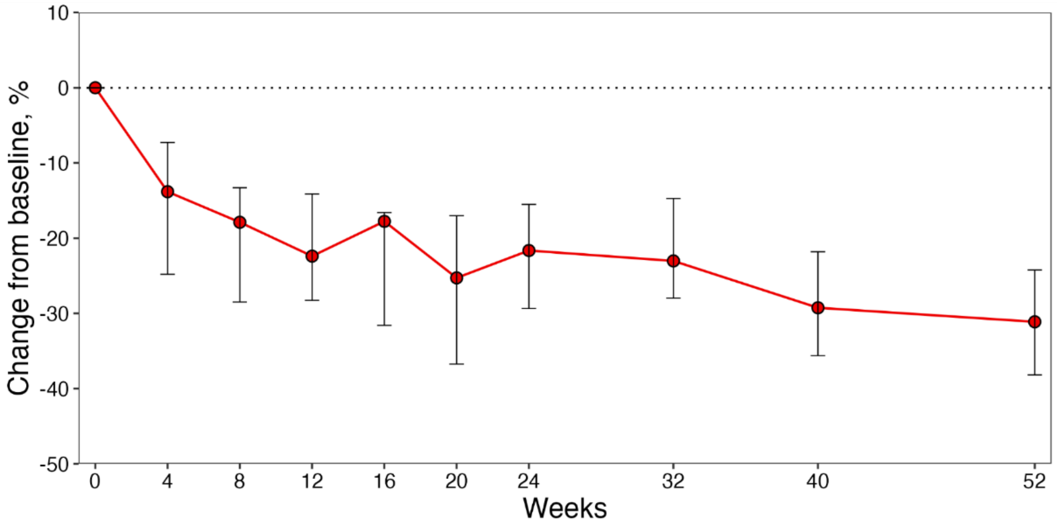

Supplementary Figure 12. IgG subclass levels. The line plots show medians and interquartile range (IQR). Absolute baseline levels (mg/dL): IgG1, 412 (IQR 361-682); IgG2, 193 (IQR 153-276); IgG3, 41 (IQR 19-69); IgG4: 11 (IQR 3.-23).

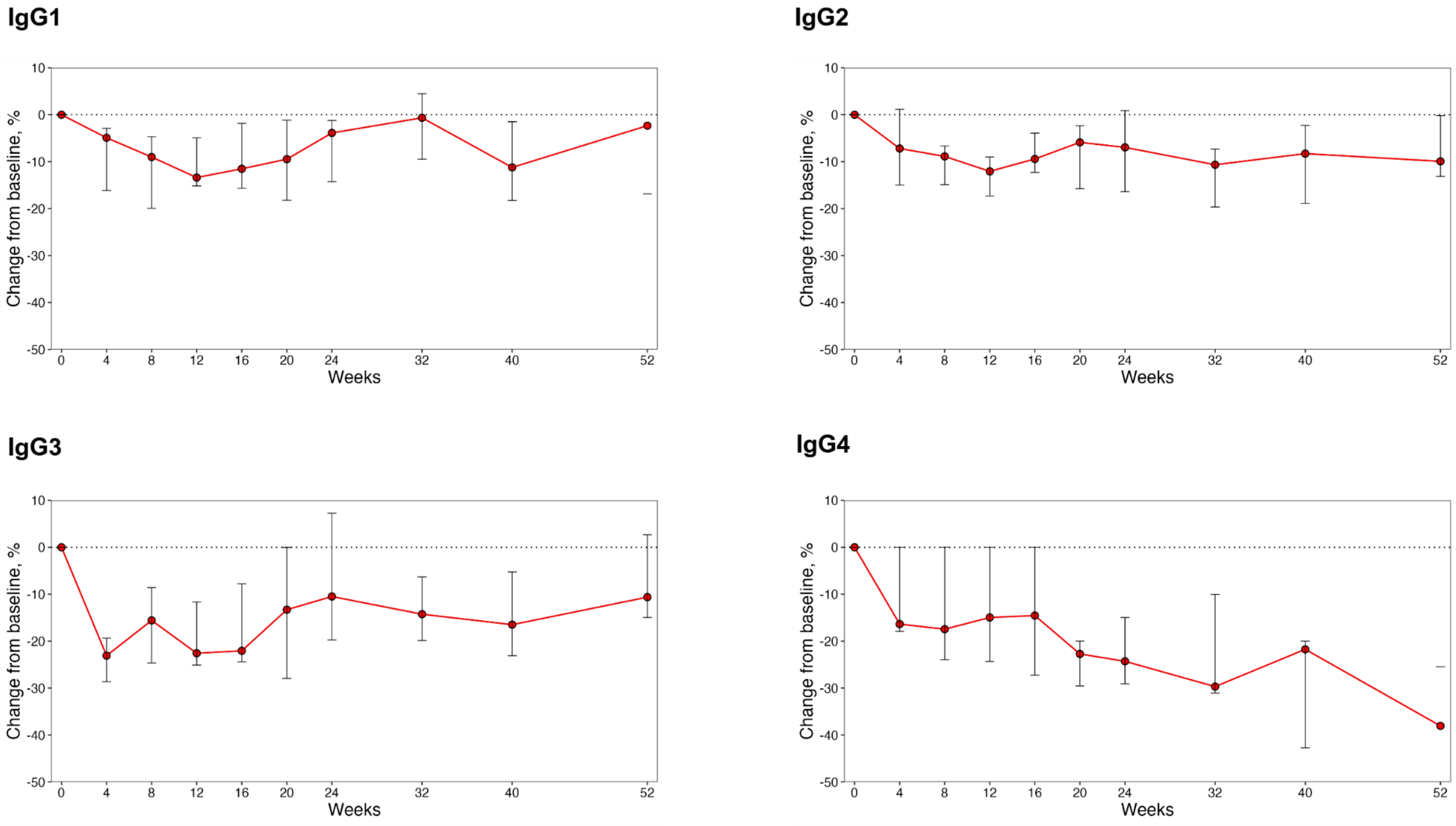

Supplementary Figure 13. Renal function and proteinuria in relation to prior randomisation. Shown are individual (thin lines) and mean estimated glomerular filtration rate (eGFR) slopes (thick lines) in relation to prior randomisation (placebo: n=4; felzartamab: n=7). Shaded areas represent 95% confidence intervals. Box plots illustrate the course of the spot urine protein-to-creatinine ratio. The horizontal line within each box represents the median; the upper and lower edges of the box indicate the interquartile range, and the whiskers represent 1·5 times the interquartile range. Individual dots represent measurements from individual patients.

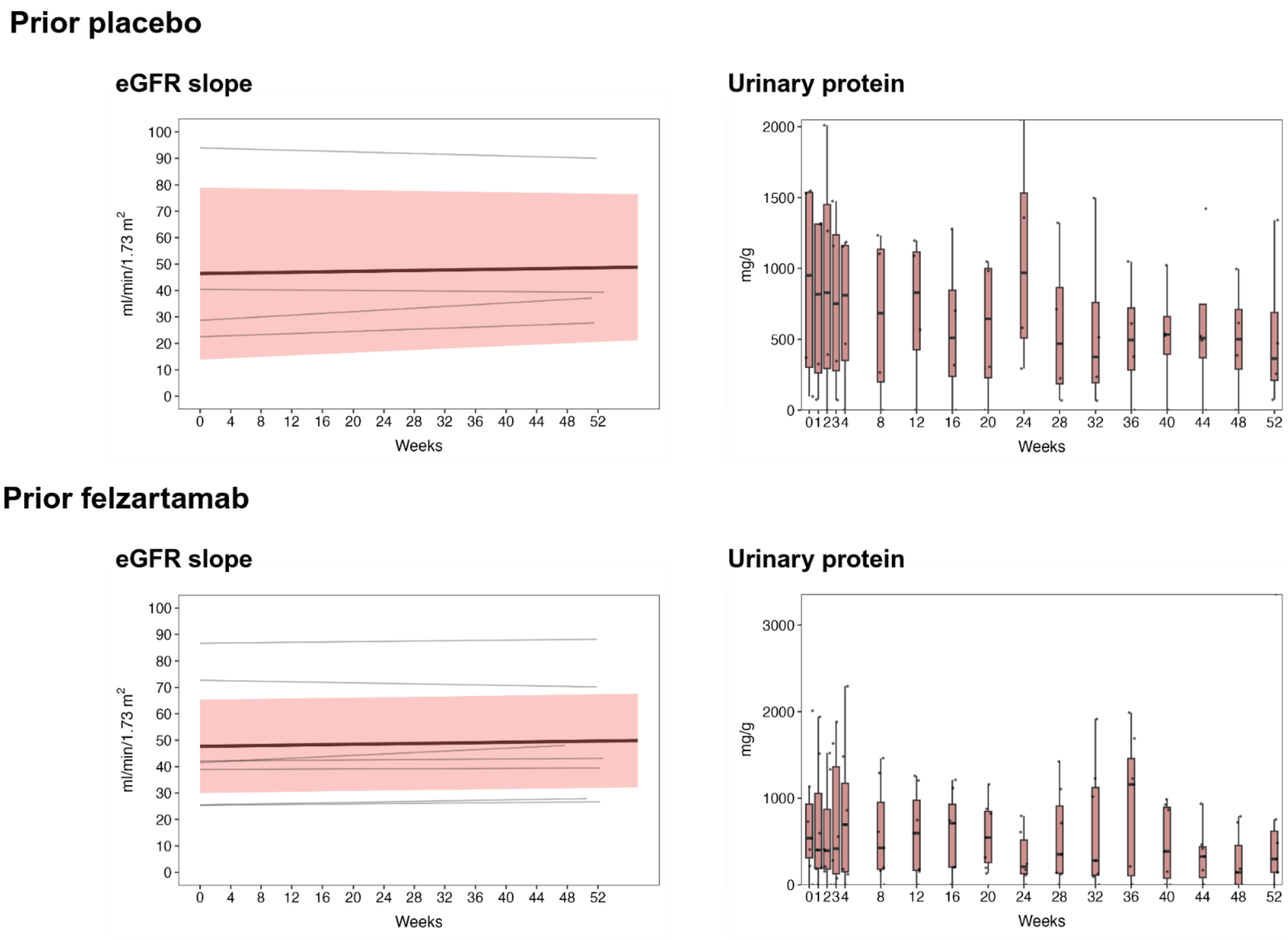

Supplementary Figure 14. Drivers of changes in iBOX predictions over time. Shown are the results of a decomposition analysis of the iBOX linear predictor from baseline to week 52. In the Cox proportional hazards model, the change in predicted risk was partitioned into variable-specific contributions, calculated as the product of each model coefficient and the corresponding change in the covariate between the two time points This approach provides an intuitive and transparent quantification of how much each parameter contributes to the overall evolution of the predicted risk. reflecting the drivers of change rather than the overall importance of variables in the model.

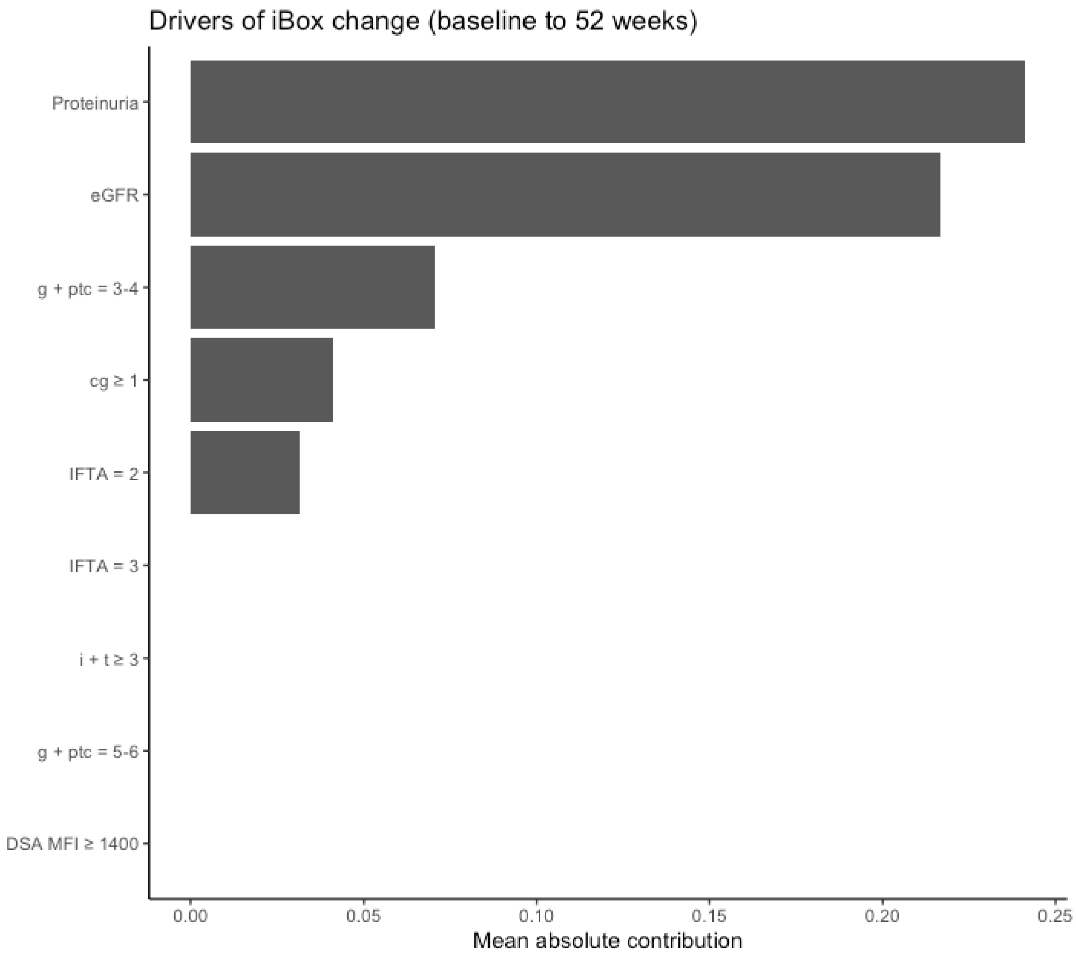

Supplementary Figure 15. Safety laboratory. Shown are levels of hematologic and liver parameters and C-reactive protein. ALT, alanine aminotransferase; AST, aspartate aminotransferase.

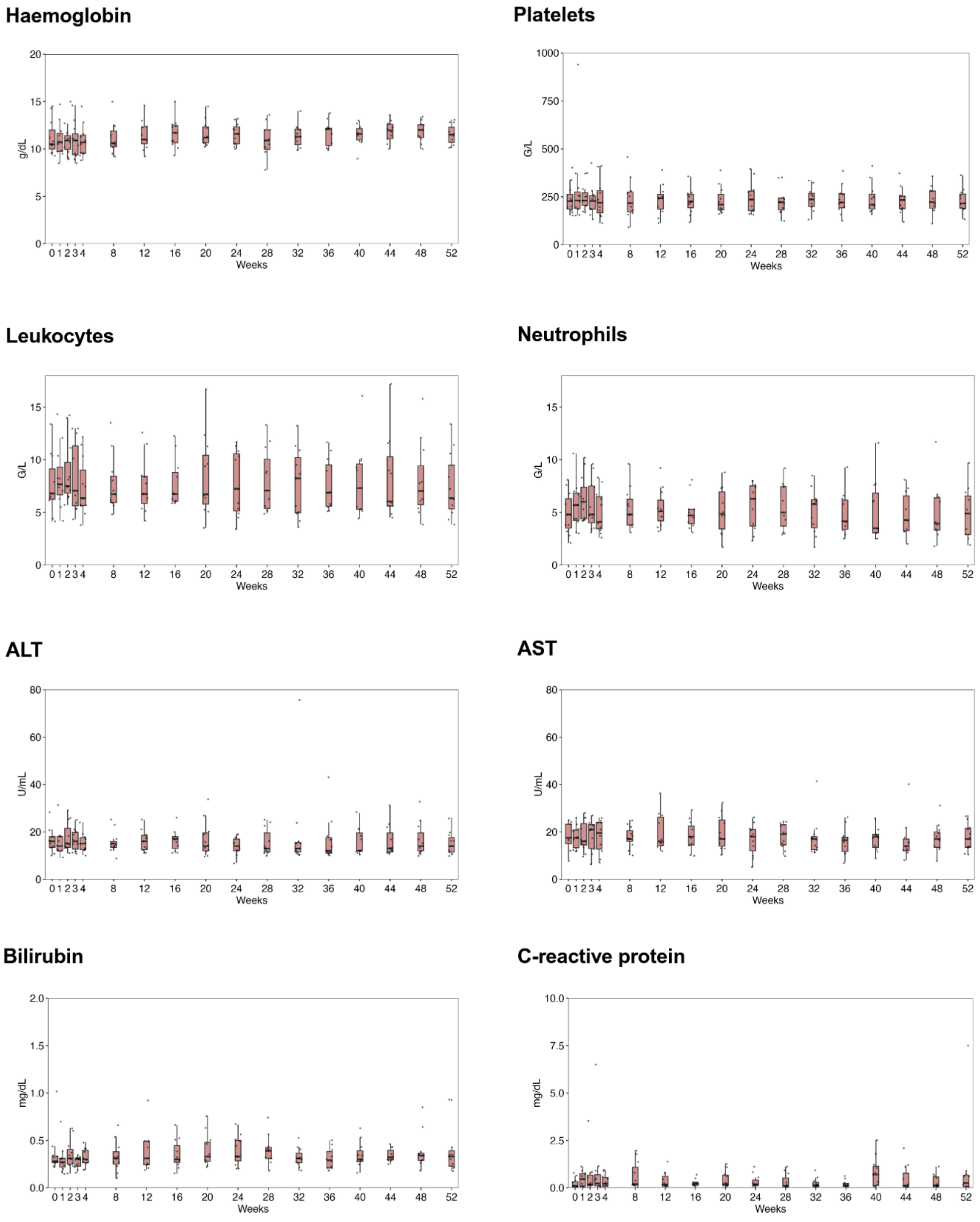

Trial Protocol

Safety, Tolerability and Efficacy of Monoclonal CD38 Antibody Felzartamab in Late Antibody-Mediated Renal Allograft Rejection – A Phase 2 Pilot Trial

EudraCT-No 2021-000545-40

The supplemental material contains the following items:

1. Original protocol - Version 2.0
2. Final protocol - Version 7.0
3. Summary of change

Study protocol

Version 2.0

Projekttitel (Deutsch)

Sicherheit, Verträglichkeit und Effektivität des monoklonalen CD38 Antikörpers Felzartamab bei später Antikörper-vermittelter Nierentransplantatabstoßung – Eine Phase 2 Pilotstudie

Project title (English)

Safety, Tolerability and Efficacy of Monoclonal CD38 Antibody Felzartamab in Late Antibody-Mediated Renal Allograft Rejection – A Phase 2 Pilot Trial

Vienna, April 30, 2021

**Trial Sponsor**		**Medical University of Vienna (MUW)**<br>Spitalgasse 23, 1090 Vienna, Austria
**Principal investigator**	**MUW**	**Prof. Dr. Georg A. Böhmig**<br>Medical University of Vienna<br>Department of Medicine III<br>Währinger Guertel 18-20<br>1090 Vienna, Austria<br>Phone: +43 1 40400 43630<br>Fax: + 43 1 40400 39302<br>Email: georg.boehmig@meduniwien.ac.at
**Co-Principal Investigator**		**Prof. Dr. Bernd Jilma**<br>Medical University of Vienna<br>Department of Clinical Pharmacology<br>Währinger Guertel 18-20<br>1090 Vienna, Austria<br>Phone: +43 1 40400 29810<br>Fax: + 43 1 40400 29980<br>Email: bernd.jilma@meduniwien.ac.at
**Investigators, MUW (alphabetical order)**		**Anita Borski**<br>Medical University of Vienna<br>Department of Medicine III<br>Währinger Guertel 18-20<br>1090 Vienna, Austria<br>Phone: +43 1 40400 43630<br>Fax: + 43 1 40400 39302<br>Email: anita.borski@meduniwien.ac.at<br>or: anitadumler@googlemailcom<br>**Dr. Konstantin Doberer**<br>Medical University of Vienna<br>Department of Medicine III<br>Währinger Guertel 18-20<br>1090 Vienna, Austria<br>Phone: +43 1 40400 55260<br>Fax: + 43 1 40400 39302<br>Email: konstantin.doberer@meduniwien.ac.at<br>**Dr. Farsad Eskandary**<br>Medical University of Vienna<br>Department of Medicine III<br>Währinger Guertel 18-20<br>1090 Vienna, Austria<br>Phone: +43 1 40400 43630<br>Fax: + 43 1 40400 39302<br>Email: farsad.eskandary@meduniwien.ac.at<br>**Dr. Christa Firbas**<br>Medical University of Vienna<br>Department of Clinical Pharmacology<br>Währinger Guertel 18-20<br>1090 Vienna, Austria<br>Phone: +43 1 40400 29810<br>Fax: + 43 1 40400 29980<br>Email: christa.firbas@meduniwien.ac.at

		**Dr. Alexander Kainz**<br>Medical University of Vienna<br>Department of Medicine III<br>Währinger Guertel 18-20<br>1090 Vienna, Austria<br>Phone: +43 1 40400 43910<br>Fax: + 43 1 40400 43920<br>**Dr. Katharina Mayer**<br>Medical University of Vienna<br>Department of Medicine III<br>Währinger Guertel 18-20<br>1090 Vienna, Austria<br>Phone: +43 1 40400 72520<br>Fax: + 43 1 40400 39302<br>Email: katharina.mayer@meduniwien.ac.at<br>**Prof. Dr. Heinz Regele**<br>Medical University of Vienna<br>Clinical Institute of Pathology<br>Währinger Guertel 18-20<br>1090 Vienna, Austria<br>Phone: +43 1 40400 49730<br>Fax: + 43 1 40400 51930<br>Email: heinz.regele@meduniwien.ac.at<br>**PD Dr. Christian Schörgenhofer**<br>Medical University of Vienna<br>Department of Clinical Pharmacology<br>Währinger Guertel 18-20<br>1090 Vienna, Austria<br>Phone: +43 1 4040029810<br>Fax: +43 1 40400 29980<br>Email:<br>christian.schoergenhofer@meduniwien.ac.at<br>**PD Dr. Markus Wahrmann**<br>Medical University of Vienna<br>Department of Medicine III<br>Währinger Guertel 18-20<br>1090 Vienna, Austria<br>Phone: +43 1 40400 6207<br>Fax: + 43 1 40400 39302<br>Email: markus.wahrmann@meduniwien.ac.at
**Investigators - Second participating center**	**Charité Berlin**	**Dr. Michael Dürr**<br>Charité Berlin<br>Department of Nephrology<br>Charitéplatz 1<br>10117 Berlin, Germany<br>Phone: +49 30 450 614 244<br>Fax: +49 30 450 514 002<br>Email: michael.duerr@charite.de<br>**Prof. Klemens Budde**<br>Charité Berlin<br>Department of Nephrology

		Chariteplatz 1<br>10117 Berlin, Germany<br>Phone: +49 30 450514002<br>Fax: 49 30 450514002<br>e-mail: klemens.budde@charite.de
**Co-Investigator (for 'Molecular Microscope', MMDx)**	**University of Alberta**	**Prof. Dr. Philip Halloran**<br>Alberta Transplant Applied Genomics Centre<br>Faculty of Medicine & Dentistry<br>#250 Heritage Medical Research Centre<br>University of Alberta<br>Edmonton, Alberta, Canada<br>T6G 2S2<br>Phone: (780) 492-6160<br>Fax: (780) 407-7450<br>Email: Halloran-admin@med.ualberta.ca

Summary of the project (Deutsch)

Projekttitel

Sicherheit, Verträglichkeit und Effektivität des monoklonalen CD38 Antikörpers Felzartamab bei später Antikörper-vermittelter Nierentransplantatabstoßung – Eine Phase 2 Pilotstudie

Wissenschaftlicher Hintergrund. Trotz kontinuierlicher Verbesserungen in der Betreuung nierentransplantierter Patienten/Patientinnen hat sich in den letzten Jahren gezeigt, dass das Überleben von Nierentransplantaten in der frühen Phase nach Transplantation zwar verbessert werden konnte, nicht aber das Transplantatüberleben im Langzeitverlauf. Dies konnte auch nicht durch die Implementierung neuer basisimmunsuppressiver Strategien erreicht werden. Eine wesentliche Erkenntnis der letzten Jahre ist, dass immunologische Prozesse (Abstoßungen) eine wesentliche kausale Bedeutung bei chronischen Transplantatverlusten darstellen. Insbesondere Antikörper gegen Spender-Alloantigene (HLA Antigene), möglicherweise in Kombination mit einer Antikörper-vermittelten Aktivierung zellulärer Mechanismen (z.B. Aktivierung von natürlichen Killerzellen) sind ein wesentlicher Trigger für den chronischen Transplantatschaden. Die Antikörper-vermittelte Abstoßung („antibody-mediated rejection“, ABMR) ist heute als eigene Entität gut etabliert, wobei die diagnostischen Kriterien, eine essenzielle Basis für gezielte therapeutische Interventionen, weitgehend definiert sind: Inflammation und morphologische Schäden in der Mikrozirkulation, (nicht obligate) Ablagerung des Komplementspaltprodukts C4d entlang des Transplantat-Endothels, und Nachweis von Antikörpern gegen Spenderantigene („donor-specific antibodies“, DSA). Mit der Definition spezifischer ABMR-Kriterien hat sich nun erwiesen, dass chronische ABMR eine der führenden Ursachen für einen Transplantatschaden, Dysfunktion und letztlich Verlust darstellt. Im Gegensatz zur frühen aktiven ABMR, wo verschiedene anti-humorale therapeutische Strategien, teilweise auch in kontrollierten Studien erprobt wurden, sind therapeutische Optionen für die späte chronische ABMR kaum definiert. Die klinischen Erfahrungen beschränken sich hier weitgehend auf anekdotische Berichte und kleine Fallserien. Erst rezent wurden immunmodulatorische Therapiestrategien, wie terminale

Komplement-Blockade (Eculizumab), oder Proteasom-Inhibition mit dem Ziel einer Plasmazell-Depletion (Bortezomib), in randomisierten Protokollen evaluiert. Allerdings haben diese Studien keinen eindeutigen Effekt solcher Therapien auf den Verlauf chronischer Abstoßungsprozesse ergeben. Es bleibt anzumerken, dass ein weiterer Ansatz, die Antagonisierung der Interleukin-6 (IL-6)/IL-6 Achse derzeit intensiv untersucht wird. Eine aktuelle Pilotstudie unserer Arbeitsgruppe wies dabei auf mögliche günstige Effekte in Hinblick auf Alloantikörper-Produktion und Nierenfunktionsverlauf hin. Allerdings wird diese Therapie, die aktuell bzgl. klinischer Endpunkte und vor allem auch deren Sicherheit und Verträglichkeit (Signal einer erhöhten Infektionsrate und gastrointestinaler Komplikationen in der Pilotstudie) in einer multizentrischen Phase 3 Studie eingehend untersucht wird, für die Routine der Abstoßungstherapie für längere Zeit nicht verfügbar sein. Letztlich steht damit nach wie vor keine effiziente Therapie für die Behandlung der späten chronischen ABMR zur Verfügung, und die optimale Therapie für späte chronische ABMR bleibt weiterhin unklar.

Ein attraktives therapeutisches Target ist CD38, ein 45 kDa Typ II Transmembran-Glycoprotein, welches auf zahlreichen Immunzellpopulationen exprimiert wird, in höchster Dichte auf (normalen und malignen) Plasmazellen und Natürlichen Killerzellen (NK-Zellen). Therapeutische monoklonale CD38 Antikörper haben bereits Eingang in die klinische Routine, z.B. in der Behandlung des Multiplen Myeloms, gefunden, wobei hier die Antikörper Daratumumab und Isatuximab für diese Indikation behördlich in den USA und Europa zugelassen sind. Für die Transplantationsmedizin ist die Möglichkeit einer effektiven Plasmazell-Depletion über CD38 von großem Interesse, da dadurch, wie in rezenten Fallberichten gezeigt werden konnte, eine nachhaltige anti-HLA Antikörper-Reduktion erreicht werden kann. Zudem hat sich gezeigt, dass NK Zellen (mit hoher CD38 Expression) eine tragende Rolle als Effektoren einer Fc Rezeptor-mediierten Transplantatschädigung spielen, was zu einem unmittelbaren Therapieeffekt einer CD38 Bindung beitragen könnte. Tatsächlich konnten wir rezent in einem Fall einer ABMR bei einem nierentransplantierten Patienten, der zudem die Diagnose einer Plasmazellerkrankung hatte (Smoldering Myelom), eine komplette morphologische und klinische Reversion der Abstoßung (und auch des Myeloms), eine effektive Depletion von Plasmazellen und damit Alloantikörpern, sowie eine fast komplette Depletion von NK Zellen im transplantierten Gewebe und peripheren Blut zeigen.

Letztlich bleibt aber der tatsächliche Nutzen durch eine solche Therapie mangels systematischer Daten (momentan sind nur wenige Fallberichte verfügbar) unbewiesen. Wenn auch bei Patienten mit Multiplem Myelom ein günstiges Sicherheitsprofil verschiedener CD38 Antikörper gezeigt werden konnte, gibt es für immunsupprimierte transplantierte Patienten noch keine ausreichenden Daten zu Sicherheit und Verträglichkeit. Dies betrifft nicht nur Erstdosisreaktionen (v.a. Daratumumab) sondern auch aufgrund einer zusätzlichen Immunmodulation ein potenziell erhöhtes Infektionsrisiko.

Ein interessanter Kandidat für eine CD38-gerichtete Antikörpertherapie ist der rekombinante monoklonale voll-humane CD38 Antikörper Felzartamab (MOR202; MOR03087; humanes IgG1λ; MorphoSys AG, Planegg, Deutschland). Dieser Antikörper ist in präklinischen Modellen in seiner Pharmakokinetik und Wirkweise gut untersucht. Felzartamab induziert dabei eine Zytolyse CD38 hochexprimierender Zellen (in Tiermodellen Myelomzellen), über Antikörper-mediierte zelluläre Toxizität (ADCC) und Phagozytose, nicht aber (wie z.B. bei Daratumumab) Komplementaktivierung. In einer (first-in-human) multizentrischen Phase 1-2a Studie konnte eine hohe Effizienz und Sicherheit in der Therapie des relapsierenden oder refraktären Multiplen Myeloms gezeigt werden. Eine Besonderheit war dabei ein im Vergleich zu anderen klinisch eingesetzten CD38 Antikörpern ein geringeres Risiko für Infusions-assoziierte Reaktionen. Bzgl. anderer Adverse Events (AE) war das Profil anderen CD38 Antikörpern vergleichbar.

Felzartamab soll nun im Rahmen dieser Phase 2 Studie bei nierentransplantierten Patienten mit aktiver bzw. chronisch-aktiver ABMR eingehend untersucht werden.

Zielsetzung. Im Rahmen dieser (akademischen) prospektiven, randomisierten, doppelblinden, Placebo-kontrollierten, Pilotstudie (Phase 2) soll die Sicherheit, Verträglichkeit, Effektivität, Pharmakokinetik und Pharmakodynamik einer Therapie mit dem monoklonalen CD38 Antikörper Felzartamab bei Nierentransplantierten mit später aktiver bzw. chronisch-aktiver ABMR untersucht werden. Die Studie soll in erster Linie Aufschluss über die Sicherheit und Verträglichkeit dieser neuen Therapie bei immunsupprimierten Patienten geben, zudem aber (sekundäre Endpunkte) erste Ergebnisse zur Effektivität in der Therapie später aktiver ABMR ermöglichen (v.a. Abschätzung der Effektgröße und Variabilität als Basis für die Planung einer

nachfolgenden großen „proof of concept“ Studie). Dies beinhaltet in erster Linie eine Analyse des Effekts einer gezielten anti-CD38 Therapie auf Inflammation in der Mikrozirkulation, Abstoßungs-assoziierter Genexpressionsmuster, sowie Progression chronischer Schäden in frühen und späten Transplantat-Biopsien, auf Biomarker aktiver ABMR (DSA-Nachweis und -Verlauf, Serummarker für Inflammation und Endothelaktivierung/schaden, Verteilung von Leukozyten(sub)populationen), sowie Nierenfunktion, Proteinurie und prädiktiver Scores (als Surrogat für Langzeit-Transplantatüberleben).

Studiendesign. Die Studie wird an der Medizinischen Universität Wien, sowie an einem zweiten Zentrum (Charité Berlin) durchgeführt (Inklusion von je 5-15 Patienten; insgesamt 20 Patienten). Dabei handelt es sich um eine prospektive, randomisierte doppelblinde Placebo-kontrollierte Pilotstudie der Phase 2. Es erfolgt eine 1:1 Randomisierung in zwei Gruppen, eine Felzartamab- und eine Placebo-Gruppe, wobei in 6 Zyklen insgesamt 9 Infusionen (Dosis: je 16 mg/kg) über einen Zeitraum von 24 Wochen verabreicht werden. Die Rekrutierungsphase soll 12-18 Monate betragen, daraus resultierend eine Studiendauer von 24-30 Monaten.

Primärer Endpunkt sind die Sicherheit und Verträglichkeit einer Therapie mit Felzartamab. Sekundäre Endpunkte sind die Pharmakokinetik (Konzentrationsverlauf von Felzartamab), Immunogenität (Nachweis von Antikörpern gegen Felzartamab) und Phamakodynamik (Plasmazell- und NK Zell-Reduktion im peripheren Blut) dieses Antikörpers, sowie eine präliminäre Analyse des Effekts auf den Abstoßungsprozess in zwei Protokollbiopsien, nach 24 und nach 52 Wochen: (i) Inflammation in der Mikrozirkulation, (ii) Progression chronischer Transplantatläsionen (Transplantatglomerulopathie, interstitielle Fibrose/tubuläre Atrophie), und (iii) molekulare Signaturen Antikörper-mediierter akuter und chronischer Transplantatschädigung. Des Weiteren wird der Effekt auf Abstoßungs-assoziierte Biomarker, wie DSA im Verlauf (parallel Serum-Immunglobulin-Spiegel und Impftiter), Zytokinmuster und Marker eines Endothelschadens sowie die Verteilung von Leukozyten-Subpopulationen und eine Genexpressionsanalyse auf RNA-Ebene in peripheren Blutzellen - komplementär zur Genexpressionsanalyse aus dem Nierenbiopsiematerial – durchgeführt. Der Grad der Immunsuppression wird über die Bestimmung der Torque Teno Virus (TTV)-Last und eine Analyse von Impftitern abgeschätzt werden. Klinische Endpunkte als Maß für Abstoßungsprogression sind

der Verlauf der eGFR (eGFR Slope), Proteinausscheidung (Protein/Kreatinin-Ratio), Transplantat- und Patientenüberleben, sowie das Auftreten akuter Abstoßungsepisoden. Mit erfolgter Inklusion von 10 bzw. 20 Patienten/Patientinnen (die zweite Interimanalyse erfolgt somit 1 Jahr vor Studienende) erfolgen Interimanalysen, deren Ergebnisse durch ein unabhängiges „Data and Safety Monitoring Board“ (DSMB) bewertet werden. Exakte (statistische) Kriterien für eine vorzeitige Studienbeendigung sind für diese Pilotstudie nicht definiert. Nach Bewertung der Sicherheit und Datenqualität in den zwei Interimanalysen wird die Studie jedoch vorzeitig beendet, wenn (i) in der Felzartamab-Gruppe schwere unerwünschte Ereignisse auftreten, die nach Einschätzung des DSMB auf ein inakzeptables Nutzen-Risiko-Profil hinweisen, und (ii) die Zahl der Drop-outs so hoch ist, dass ein adäquater Abschluss der Studie nicht gewährleistet werden kann.

Patienten. In diese Pilotstudie werden 20 erwachsene Patienten/Patientinnen inkludiert. Wesentliche Einschlusskriterien sind die Diagnose einer späten aktiven oder chronisch/aktiven ABMR (C4d-positiv oder –negativ), zumindest 180 Tage nach Transplantation (Befund einer Indikationsbiopsie = Indexbiopsie, die im Rahmen der klinischen Routine aufgrund einer DSA Detektion und/oder einer langsamen Verschlechterung der Transplantatfunktion und/oder einer steigenden Proteinurie durchgeführt wurde), eine eGFR ≥20 ml/min/1.73 m^2 (Ausschluss weit fortgeschrittener irreversibler schwerer chronischer Schäden des Transplantats), der Nachweis von HLA Klasse I und/oder II-spezifischen DSA, sowie ein Alter >18 Jahre. Ausschlusskriterien sind eine aktive Teilnahme an einer anderen interventionellen Studie, Schwangerschaft und Stillperiode, ABO-inkompatible Transplantation, akute Abstoßung innerhalb eines Monats vor Inklusion, akute Verschlechterung der Transplantatfunktion mit Verdacht auf eine akute Abstoßung, Applikation eines anderen CD38 Antikörpers (z.B. Daratumumab) vor Studieninklusion, Applikation eines anderen immunmodulatorischen Antikörpers (z.B. Rituximab, anti-IL-6 oder anti-IL-6R Antikörper) ≤3 Monate vor Studieninklusion, erhöhte Leberwerte (Gesamt-Bilirubin >2×Upper Limit of Normal [ULN], Transaminasen >2·5×ULN), ein Hämoglobin <8 g/dL, Thrombozytenzahl <100 G/L, Leukozytenzahl <3 G/L, Neutrophilenzahl <1.5 G/L, eine Hypogammaglobulinämie mit einem Serum IgG <400 mg/dL, eine aktive virale, bakterielle oder Pilz-Infektion, die den Einsatz einer intensivierten Immunsuppression verbietet, hier vor allem auch eine latente oder aktive Tuberkulose

(positiver QuantiFERON-TB-Gold test), eine aktive maligne Erkrankung, eine schwere medizinische bzw. psychiatrische Erkrankung, die eine Teilnahme an einer klinischen Studie unmöglich macht, sowie eine Impfung mit einem Lebendimpfstoff innerhalb 6 Wochen vor Studieninklusion. Weitere Ausschlusskriterien sind der Nachweis einer T-Zell-mediierten Abstoßung (≥Grad I gemäß Banff Klassifikation), einer Polyomavirus-Nephropathie, einer *de novo* oder rekurrenten Glomerulonephritis, sowie einer *de novo* oder rekurrenten thrombotischen Mikroangiopathie in der Indexbiopsie.

Methodik. Die Intervention besteht in der seriellen intravenösen Verabreichung von Felzartamab in einer Dosis von 16 mg/kg in 250 mL 0.9% NaCl, im ersten Monat (erster Zyklus) in 1-wöchigem Abstand, dann in 4-wöchigem Abstand über eine Periode von 5 Monaten (Zyklus 2-6; insgesamt 6 Monate Therapiedauer). Patienten der Placebo-Gruppe erhalten Infusionen mit 250 mL 0.9% NaCl. Vor den ersten beiden Verabreichungen erfolgt eine intravenöse Vortherapie mit 100 mg Prednisolon, 30 mg Diphenhydramin und 1000 mg Paracetamol (in der Placebogruppe wird stattdessen Placebo verabreicht). Bei allen Patienten wird nach Zentrumsstandard die Basisimmunsuppression dem Befund einer ABMR in der Indexbiopsie angepasst (Adjustierung von Tacrolimus bzw. Cyclosporin A mit einem Ziel-Talspiegel von 5-10 ng/mL bzw. 80-120 ng/mL; bei Steroid-Freiheit Wiederbeginn mit Prednisolon, 5 mg/Tag; bei Dualtherapie ohne einen Proliferationsinhibitor, Start einer Therapie mit Mycophenolat Mofetil (oder, alternativ, „enteric-coated" Mycophenolsäure (EC-MPA)) mit einer Zieldosis von bis zu 2000 (bzw. 1440 mg)/Tag. Biomarker aus peripherem Blut: Die Detektion von DSA erfolgt unter Einsatz von Bead Array Technologie. Dabei werden HLA-Antigen-beladene Mikropartikel (Single Antigen Kits für die Spezifizierung der Spender-Spezifität nach Abgleichen mit dem Spender-HLA-Typ) mit Patienten- (bzw. Kontroll) Seren inkubiert, anschließend mit einem Fluoreszenz-markierten zweiten Antikörper die Bindung von IgG detektiert. Der Nachweis von Markern einer Endothel- und B-Zell-Aktivierung (Chemokine, BAFF) erfolgt unter Verwendung von Luminex-Assays. Der Nachweis von TTV erfolgt mittels quantitativer PCR. Leukozyten-Subpopulationen werden mittels Durchflusszytometrie erfasst. Für eine Genexpressionsanalyse auf RNA-Ebene in peripheren Blutzellen wird Vollblut in PAXgene Röhrchen aufgenommen, anschließend die RNA isoliert und in cDNA umgeschrieben. Konzentrationsanalysen und anti-Felzartamab Antikörper: Die Serumkonzentration von Felzartamab und der Nachweis von möglichen Bildung von

anti-Falzaratamab Antikörper wird mittels Ligandbindungsassays bestimmt. Transplantat-Biopsien: Biopsien (nach 12 und 52 Wochen) werden mittels Ultraschall-Kontrolle gemäß Zentrumsstandard durchgeführt (Verwendung einer 16 G Nadel; 1-2 Biopsiezylinder für eine Asservierung von Material für Lichtmikroskopie inkl. Immunhistochemie (C4d), Elektronenmikroskopie, und Genexpressionsanalysen. Die Diagnose und Kategorisierung einer ABMR erfolgt gemäß Banff 2019 Update auf Basis einer eingehenden morphologischen und molekularen Aufarbeitung. Diese umfasst ein Scoring von Glomerulitis, peritubulärer Kapillaritis, Transplantat-Glomerulopathie, interstitieller Fibrose und Tubulusatrophie (IFTA); eine immunhistochemische Aufarbeitung für den Nachweis kapillärer C4d-Ablagerung (fokal versus diffus; Scoring nach der Banff Klassifikation) sowie Elektronenmikroskopie für den Nachweis einer Lamellierung peritubulärer und glomerulärer Basalmembranen. Der Nachweis einer ABMR in der Genexpressionsanalyse (Kriterium der Banff Klassifikation; ein ABMR Score ≥0.2 ist Voraussetzung für eine Studieninklusion) erfolgt mittels cDNA Microarrays am Alberta Transplant Applied Genomics Centre, ATAGC, in Edmonton, Kanada („molecular microscope", MMDx ™). Klinische Evaluation: Die Bestimmung der errechneten glomerulären Filtrationsrate (eGFR) erfolgt unter Verwendung der CKD-EPI eGFR, die Harnauscheidung von Protein wird mittels Protein/Kreatinin Ratio aus dem Spontanharn erfasst. Zudem wird nach 24 und nach 52 Wochen ein international validierter Prädiktions-Score (sog. iBOX Score unter Inklusion von Biopsie-assoziierten Parametern) berechnet, als Surrogat für eine Modifikation des Transplantat-Langzeitüberlebens. Statistische Analyse: Alle Analysen erfolgen nach dem Intention-to-Treat Prinzip. Kontinuierliche Daten werden mittels parametrischen oder nicht parametrischen Tests unabhängiger oder gepaarter Stichproben, kategorische mittels Fisher's Exact Tests berechnet. Die Berechnung der eGFR Slope erfolgt mittels einem linearen gemischten Modell mit dem Verlauf der eGFR (Tag 0 bis Woche 52, alle 4 Wochen) als abhängiger Variable. Die Auswertung inkludiert zudem Analysen der Pharmakokinetik (Halbwertszeit, Tmax, Cmax, Clearance und Verteilungsvolumen), unter Einsatz von Standard-Software.

Belastungen, mögliche Nebenwirkungen:

Im Rahmen der Studie entstehen Mehrbelastungen durch Verabreichung von Felzartamab bzw. Plazebo (9 Verabreichungen in 6 Zyklen). Nebenwirkungen, die

durch eine Therapie mit Felzartamab entstehen können (Daten aus einer Phase I-IIa Studie bei Multiplen Myelom) bestehen in erster Linie in Infusions-assoziierten Reaktionen zu Beginn der Therapie und hämatologischer Toxizität (Leukozytopenie, Anämie, Thrombozytopenie). Auch wenn in rezenten Phase 3 Studien für CD38 Antikörper keine substantielle Erhöhung des Infektionsrisikos im Vergleich zu Placebo oder anderen Myelom-Therapien gezeigt werden konnte, ist in Kombination mit immunsuppressiver Therapie bei Transplantierten von einem insgesamt erhöhten Infektionsrisiko auszugehen (entsprechend engmaschiges Infektions-Monitoring, wie in der Routine der Transplant-Nachbetreuung vorgesehen; aktive virale, bakterielle und Pilzinfektionen als Ausschlussgrund für einen Einschluss in die Studie. Eine weitere Belastung besteht, neben Routine-Laborkontrollen inkl. CNI-Spiegelbestimmung bei jeder Visite, in der Entnahme von zusätzlich 2 x 5 ml Blut bei der Mehrzahl aller Visiten (Pharmakokinetik und Immunogenität)) sowie zusätzlich 30 ml Blut am Tag 0, nach 12, 24 und nach 52 Wochen für die Gewinnung von Serum, Plasma und peripheren Blutzellen, zur Bestimmung von DSA, Leukozyten-(Sub)populationen, Chemokinen, TTV Virämie, sowie für Genexpressionsanalysen. Für Protokollbiopsien (zwei geplante Biopsien, nach 24 und nach 52 Wochen) gilt ein gut dokumentiertes geringes Komplikationsrisiko (selten: Hämatom, Hämaturie, außerordentlich selten Notwendigkeit einer chirurgischen Revision bis hin zu Nephrektomie, außerordentlich selten Blutdruckabfall bis hin zum Blutungsschock). Blutungsrisiken werden durch ein striktes Einhalten des Zentrums-Standards für Transplantat-Biopsie minimiert.

Risiken/Nutzenabwägung. Die chronische Abstoßung ist eine Hauptursache für einen Transplantatverlust im Langzeitverlauf. Bislang sind keine Therapien etabliert, die dem natürlichen Verlauf später Antikörper-vermittelter Abstoßung entgegenwirken. Der Organfunktionsverlauf für Patienten mit später ABMR ist ungünstig. Erste Ergebnisse der geplanten Studie haben das Potenzial, die Effizienz einer CD38 Antikörpertherapie, eines neuen therapeutischen Prinzips in der Therapie der chronischen Abstoßung, abzuschätzen. Sollten sich Hinweise ergeben, dass durch diese Therapie das Fortschreiten akuter und chronischer irreversibler Läsionen aufgehalten bzw. verzögert werden kann, dann hätte dies bedeutende Implikationen für immunsuppressive Therapiestrategien in der Organtransplantation. In Hinblick auf die bedeutende Rolle humoraler Abstoßungsprozesse als wesentlicher Mediator

chronischer Läsionen könnte der erfolgreiche Einsatz innovativer Therapien günstige Effekte auf das Langzeit-Transplantatüberleben haben. Demgegenüber ist eine Teilnahme an der Studie mit Belastungen und Risiken assoziiert (siehe oben). Für die Placebo-Gruppe im ersten Teil der Studie ergibt sich kein Nachteil, da diese, wie bisher nach Standard behandelt werden: dies beinhaltet eine Zugabe eines Steroids und/oder eines Proliferationshemmers (Mycophenolsäure), sofern nicht ohnehin Teil der Basistherapie, nicht aber eine spezifische anti-humorale Therapie, wie sie bei akuter Dysfunktion aufgrund einer frühen aktiven ABMR eingesetzt wird (z.B. Apherese). In Hinblick auf den möglichen Nutzen durch eine eventuelle Etablierung eines neuen immunsuppressiven Therapieansatzes für Transplantierte sowie den möglichen unmittelbaren Nutzen für die Patienten in der Interventionsgruppe, ist das Risiko durch Interventionen und Behandlungen im Rahmen der Studie vertretbar.

Clinical study protocol

Version 2.0

Safety, Tolerability and Efficacy of Monoclonal CD38 Antibody Felzartamab in Late Antibody-Mediated Renal Allograft Rejection - A Phase 2 Pilot Trial

Trial Sponsor

Medical University of Vienna (MUW)
Spitalgasse 23, 1090 Vienna, Austria

Principal Investigator

Prof. Dr. Georg A. Böhmig
Medical University of Vienna
Department of Medicine III
Währinger Guertel 18-20
1090 Vienna, Austria
Phone: +43 1 40400 43630
Fax: + 43 1 40400 39302
Email: georg.boehmig@meduniwien.ac.at

Vienna, April 30, 2021

Table of Contents

1. LIST OF ABBREVIATIONS 17
2. BACKGROUND AND RATIONALE 18
2.1. CD38 antibody felzartamab (MOR202)....20
2.2. Pharmacokinetics and Immunogenicity of felzartamab in humans....21
2.3. Clinical efficacy and safety 22
3. HYPOTHESIS AND OBJECTIVE 22
4. STUDY DESIGN 23
4.1. Trial overview 23
4.2. Inclusion/exclusion criteria....24
4.3. Random allocation....25
4.4. Blinding and unblinding 26
5. INTERVENTIONS 26
5.1. Treatment with felzartamab/placebo....26
5.2. Premedication 27
5.3. The following medications are prohibited during the study:....28
5.4. The following concomitant medications are permitted during the study: 28
5.5. Baseline immunosuppression....28
6. OUTCOME MEASURES 28
7. METHODOLOGY 31
7.1. HLA antibody detection 31
7.2. Immunoglobulin levels 32
7.3. Pharmacokinetics and Immunogenicity 32
7.4. Transplant biopsies 32
7.5. Kidney function....33
7.6. Immunologic biomarkers 34
7.7. Leukocyte subpopulations 34
7.8. Gene expression analysis 35
7.9. Torque Teno virus (TTV) quantification 35
7.10. Course of vaccination titers....35
7.11. Collection of biological material (outside routine monitoring)....36
7.12. Responsible Laboratories that are involved into the study 36
8. SAMPLE SIZE AND STATISTICAL ANALYSIS....37
9. INTERIM ANALYSES....40
10. ADVERSE EVENTS 40

10.1. Felzartamab 40
10.2. Adverse drug reactions - specific considerations 42
10.2.1. Felzartamab 42
10.2.2. Premedication 45
10.3. Covid-19 46
10.4. Pregnancies and Women of Child Bearing Potential 46
11. SAFETY ASSESSMENT AND REPORTING 48
11.1. Safety Assessment 48
11.2. Definition of adverse events 49
11.2.1. Serious Adverse Events (SAEs) 51
11.2.2. SAEs related to study-mandated procedures 52
11.2.3. Suspected unexpected serious adverse reactions (SUSARs) 52
11.2.4. Pregnancy 52
11.2.5. Adverse events of special interest 53
11.3. Severity of adverse events 53
11.4. Relationship to study drug 54
11.5. Reporting procedures for any AEs 55
11.6. Reporting procedures for SAEs and AESIs 56
11.7. Reporting procedures for SUSARs 57
11.8. The Development Safety Update Report (DSUR) 57
12. STUDY TERMINATION, WITHDRAWAL, REPLACEMENT 57
12.1. Criteria for withdrawal 58
12.2. Follow-up of patients withdrawn from the study 59
12.3. Premature termination of the study 59
13. ETHICAL ISSUES 59
14. REGULATORY REQUIREMENTS 60
15. PERIODIC MONITORING 60
16. AUDIT AND INSPECTIONS 60
17. INVESTIGATOR TRAINING 60
18. RECORD RETENTION 61
19. STUDY REGISTRATION 61
20. MODIFICATIONS OF THE PROTOCOL 61
20.1. Protocol Amendments 61
20.2. Protocol Violations and Deviations 61
21. REFERENCES 62

1. LIST OF ABBREVIATIONS

ABMR = antibody-mediated rejection ADCC = antibody-dependent cellular cytotoxicity, ADCP = antibody-dependent cellular phagocytosis, AE = adverse event, AESI = adverse event of special interest, ALT = Alanin-Aminotransferase, AST = Aspartat-Aminotransferase, BM = basement membrane, CDC = complement-dependent cytotoxicity, cg = transplant glomerulopathy, CMV = cytomegalovirus, CNI = calcineurin inhibitor, CRF = Case-Report-Form, CyA = cyclosporine A, DSA = donor-specific antibody, DSMB = data and safety monitoring board, eGFR = estimated glomerular filtration rate, EOS = end-of-study, GCP = Good Clinical Practice, GLP = Good Laboratory Practice, HLA = human leukocyte antigen, HSV, herpes simplex virus, HZV, herpes zoster virus, IFTA = interstitial fibrosis/tubular atrophy, IgG = Immunoglobulin G, IL-6 = Interleukin-6, IL-6R = Interleukin 6 receptor, IV = intravenous, IVIG = intravenous immunoglobulin, KTX = kidney transplantation, MFI = mean fluorescence intensity, MLPTC, multilayering of peritubular capillary basement membranes, MM, multiple myeloma, MMDx = molecular microscope; MMF = mycophenolate mofetil, MPA = mycophenolic acid, mTOR = mammalian target of rapamycin, MUW = Medical University Vienna, PC = plasma cell, PD = pharmacodynamics, PK = pharmacokinetics, PTC = peritubular capillaries, SAE = serious adverse event, SAFB = single-antigen flow-beads, SD = standard deviation, TTV = Torque Teno virus, ULN = upper limit of normal

2. BACKGROUND AND RATIONALE

Antibody-mediated rejection (ABMR) is one of the leading causes of kidney allograft dysfunction and failure [1]. This type of rejection, commonly triggered by *de novo* anti-HLA donor-specific antibodies (DSA), is a prevalent finding in late indication biopsies (up to 30-40% of indication biopsies in some kidney transplant cohorts). Its diagnosis, which is based on a combination of serological, morphologic and molecular criteria, is associated with a progressive decline in glomerular filtration rate (GFR), increased proteinuria, and graft failure [1]. While a continuous diagnostic refinement has helped define the role of this rejection type as a major trigger of chronic allograft injury, treatment of late and/or chronic ABMR still represents a major challenge. Currently, there is no treatment that has been proven to modify its course. [2-4]. Our knowledge is mainly based on the results of uncontrolled studies, where a variety of different treatment strategies were evaluated, ranging from modifications in maintenance immunosuppression [5-7] to different immunomodulatory measures, including high dose intravenous immunoglobulin (IVIG) with or without CD20 antibody rituximab [8-11], the proteasome inhibitor bortezomib [12], or complement inhibitors [13]. Recent randomized controlled trials have failed to demonstrate efficiency of proteasome inhibition (BORTEJECT trial) to affect the integrity of alloantibody-producing plasma cells (PC) [14], combined use of CD20 antibody rituximab and intravenous immunoglobulin (TRITON trial) for modulation of B cell immunity [15], and anti-C5 antibody eculizumab [16]. One interesting concept may be the interference with the IL-6/IL-6R axis to modulate the activation and development of B cells and antibody production [17]. In a very recent randomized controlled phase 2 pilot trial (Vienna, Berlin) we found a modulation of DSA levels, and under prolonged treatment a modulation of molecular rejection scores in transplant biopsies, associated with a significant effect on eGFR decline [18]. However, this trial revealed important safety signals, in particular the occurrence of infection-related serious adverse events (AE) and two cases of diverticulitis, suggesting the need of a careful evaluation in larger longer-duration phase 3 trial (which is currently underway; IMAGINE; clinicaltrials.gov identifier: NCT03744910) [18].

An increased understanding of the pathophysiology of ABMR may be a clue for the design of innovative therapeutic strategies [3]. Donor-specific antibodies produced by long-lived PC bind to the endothelial surface within the microvasculature of the graft, where they trigger tissue inflammation and injury, via complement-dependent and

complement-independent mechanisms. Recent studies have suggested a dominant role of natural killer (NK), e.g. activated upon engagement of activating Fc gamma receptor IIIA with endothelium-bound IgG [19,20].

One promising immunotherapeutic target may be CD38, a 43.7 kDa type II transmembrane protein primarily expressed on immune and hematopoietic cells, with particularly high expression levels on antibody-producing PC and natural killer (NK) cells [21]. CD38 is known to exhibit ecto-enzymatic activity as nicotinamide adenine dinucleotide (NAD+)-glycohydrolase / ADP-ribosyl cyclase. *In vitro* data suggest a potential role as an adhesion receptor supporting the interaction of lymphocytes with endothelial cells by interaction with CD31 (PECAM-1) and as a cell-activating receptor that upon ligation triggers among others proliferation and cytokine production [21]. Monoclonal antibodies against this surface protein are well established to be highly effective in the treatment of multiple myeloma (MM) [22]. Mechanisms of action include complement-dependent cytotoxicity (CDC), antibody-dependent cellular cytotoxicity (ADCC)/phagocytosis (ADCP), and/or apoptotic signalling [23-25]. Considering the critical role of alloantibody-producing PC in ABMR, efficacy of this therapeutic principle can also be expected for transplant conditions where DSA are a cause of injury. Currently, however, there is only scarce experience with daratumumab in this context.

We speculate that CD38 antibody treatment may effectively deplete also DSA-producing PC and thus, by reducing the load of deleterious alloreactivity, halt the progression of rejection. Beyond an effect on DSA production, targeting CD38 may interfere also with a key pathomechanism in ABMR, that is, antibody-dependent NK cell activation.

The concept of targeting CD38 to counteract ABMR is supported by a recently published case of ABMR (associated with smoldering MM; Medical University of Vienna), where a 9-month course of CD38 antibody daratumumab has led to a complete reversal of severe chronic active ABMR, with complete morphological and molecular resolution (two follow-up biopsies) of rejection activity [26]. Treatment led to a marked reduction of DSA (associated with a depletion and modulation of bone marrow-derived alloantibody-producing PC) and, in parallel, a profound reduction in peripheral blood NK cell counts and intra-graft NK cell infiltrates. This intriguing case may provide a valuable basis for the design of a systematic trial to investigate the concept of targeting CD38 in late ABMR [26].

Support for efficacy of CD38 antibody treatment also comes from two additional very recent observations. Jordan et al. (https://atcmeetingabstracts.com/abstract/daratumumab-for-treatment-of-antibody-mediated-rejection-in-a-kidney-transplant-recipient/) reported on a case of refractory ABMR, which was completely reversed by a treatment course with daratumumab. Moreover, in a recent experimental study (sensitized rhesus macaques, transplant model), targeting CD38 was shown to significantly reduce donor-specific antibodies and to prolong renal allograft survival [27]. In the same study, the authors reported on a significant decrease in DSA in two clinical cases (combined heart and kidney allograft recipient with refractory ABMR and a highly sensitized heart transplant candidate), leading to clinical improvement of rejection and accelerated heart graft access [27].

On the basis of the above detailed preliminary data, we hypothesize that targeting CD38, and thus interference with the integrity and function of NK cells and DSA-producing PC, is able to counteract tissue inflammation and injury in ongoing ABMR, in particular, inflammation in the microcirculation and, as a consequence, alloantibody-triggered chronic graft injury.

2.1. CD38 antibody felzartamab (MOR202)

Felzartamab (MOR202; also referred to as MOR03087); MorphoSys AG, Planegg, Germany) is a recombinant fully human monoclonal CD38 antibody (IgGλ) derived from a proprietary antibody phage library (produced in human PER.C6® cells by recombinant expression technology). This antibody was initially developed for the treatment of MM, but is currently also being evaluated in autoimmune disease (membranous nephropathy). Details on felzartamab are provided in the attached Investigator Brochure. In vitro binding studies have shown a binding affinity for CD38 in the low nanomolar range. The main mode of action for MOR202-induced lysis of MM cells is ADCC and ADCP, but not CDC, which is believed to be a major contributor to infusion-related reactions. In the last decade, felzartamab has been evaluated thoroughly in preclinical (e.g. xenograft orthotopic MM mouse model) and in a recent clinical trial (phase I-IIa trial in MM patients) [28]. Felzartamab has not yet been approved by the FDA or EMA for any condition. For patients diagnosed with refractory and relapsed MM, this antibody showed an efficacy and safety profile comparable to other CD38 antibodies, such as daratumumab and isatuximab [28]. A major advantage may be a, compared to other CD38 antibodies, lower risk of infusion-related reactions [28].

The felzartamab development program includes several non-clinical [29-31] and clinical studies conducted in subjects with MM (first-in-human phase I-II; ClinicalTrials.gov Identifier: NCT01421186 [28]) and, outside the hematology field, in antiPLA2R-positive membranous nephropathy (trial ongoing; phase Ib/IIa; ClinicalTrials.gov Identifier: NCT04145440). To date, no studies with felzartamab have been conducted in organ transplant recipients.

2.2. Pharmacokinetics and Immunogenicity of felzartamab in humans

(Experimental data on pharmacokinetics are provided in the Investigator Brochure). Clinical data on pharmacokinetics of felzartamab were evaluated in patients with relapsing or refractory MM [28]. In this study, the authors observed an inverse correlation of drug clearance with serum albumin concentrations (indirect measure of neonatal Fc receptor (FcRn) activity), resulting in a moderate effect on felzartamab concentrations. Moreover, the volume of distribution was inversely correlated with the dose of the antibody. This finding was explained by target-mediated drug disposition effects, which were most prominent at lower doses. Combined MM treatments with pomalidomide or lenalidomide had no effect on the pharmacokinetics of MOR20, and felzartamab did not influence the pharmacokinetics of these drugs. Final population pharmacokinetic parameter estimates are shown in Table 1.

Table 1. Pharmacokinetic parameter estimates (phase 1-2a trial in MM patients)

Parameters	Estimates	%rel. standard error
CL (L/day)	0·413	8·728
V1 (L)	3·92	2·413
Q (L/day)	1·265	5·61
V2 (L)	2·197	10·84
Vmax (mg/day)	29·6	10·81
Km (mg/L)	1·437 FIX	-
KD (/day)	0·005988	28·88
Albumin effect on CL and Vmax	-1·802	9·513
Dose effect on V1	-0·2118	8·25
CL (L/day)	0·413	8·728

CL=clearance; IIV=inter-individual variability; KD=rate constant for decline of Vmax over time; Km=drug concentration at which the reaction rate is half the Vmax; Q=inter-

compartmental clearance; V1=central volume of distribution; V2=peripheral volume of distribution; Vmax=maximum reaction rate.

Immunogenicity in humans. So far, anti-drug antibody samples from 85 MM subjects continuously treated with felzartamab for up to 29.5 month were analyzed with no ADAs detected. These data suggest that felzartamab offers only a very low risk for the induction of anti-drug antibodies (ADA).

2.3. Clinical efficacy and safety

Details on the safety profile: see section 9. Efficacy and safety data for felzartamab as monotherapy or in combination with other treatments are available from a first-in-human phase I-IIa trial conducted in refractory or relapsing MM [28]. Trial results indicate that felzartamab can be safely administered in combination with other treatments, such as pomalidomide and dexamethasone. An important finding was a low rate of infusion-related reactions, which was by far lower than that observed for other CD38 antibodies (daratumumab, isatuximab). For MM treatment, trial results suggested a dose of 16 mg/kg and a regimen of four 1-weekly (first 28-day cycle) followed by 2-weekly infusions in subsequent cycles.

3. HYPOTHESIS AND OBJECTIVE

Clinical data to date support that targeting CD38 is safe and well tolerated, and we hypothesize that felzartamab will have an acceptable safety profile in kidney transplant recipients on baseline immunosuppression. Moreover, we hypothesize that repeated administration of felzartamab is able to counteract tissue inflammation and injury in ongoing ABMR, in particular, inflammation in the microcirculation, HLA antigen-specific B cell alloresponses and, as a consequence, alloantibody/NK cell-triggered chronic graft injury.

The objective of our planned randomized controlled pilot trial (inclusion of 2 transplant centers) will be to evaluate the safety, tolerability and efficacy of CD38 monoclonal antibody felzartamab in a prospective cohort of 20 kidney transplant recipients diagnosed with late active or chronic active ABMR. Our planned trial will evaluate preliminary efficacy of targeting CD38 in late active ABMR, and can be expected to provide a valuable basis for the potential design of a pivotal trial powered for the detection of clinical outcome differences.

4. STUDY DESIGN

This planned prospective bi-center study (University of Vienna, Charité Universitätsmedizin Berlin, Sponsor: Medical University of Vienna, Vienna, Austria) is an investigator-driven pilot trial designed to assess the safety, tolerability, pharmacokinetics, immunogenicity, pharmacodynamics and efficacy (preliminary assessment) of the fully human CD38 monoclonal antibody felzartamab in kidney transplant recipients with late active or chronic-active ABMR. The sponsor of this non-commercial trial is the Medical University of Vienna. Apart from study design, the sponsor will carry out the trial (in collaboration with research partners), being responsible for all its scientific, ethical, regulatory and legal aspects. The funder (MorphoSys AG, Planegg, Germany) has set funding conditions and will provide external funding. The trial will be designed as a randomized, controlled, double-blind phase 2 pilot trial. The primary endpoint will be safety and tolerability.

4.1. Trial overview

A flowchart of the trial is shown in Figure 1.

Figure 1

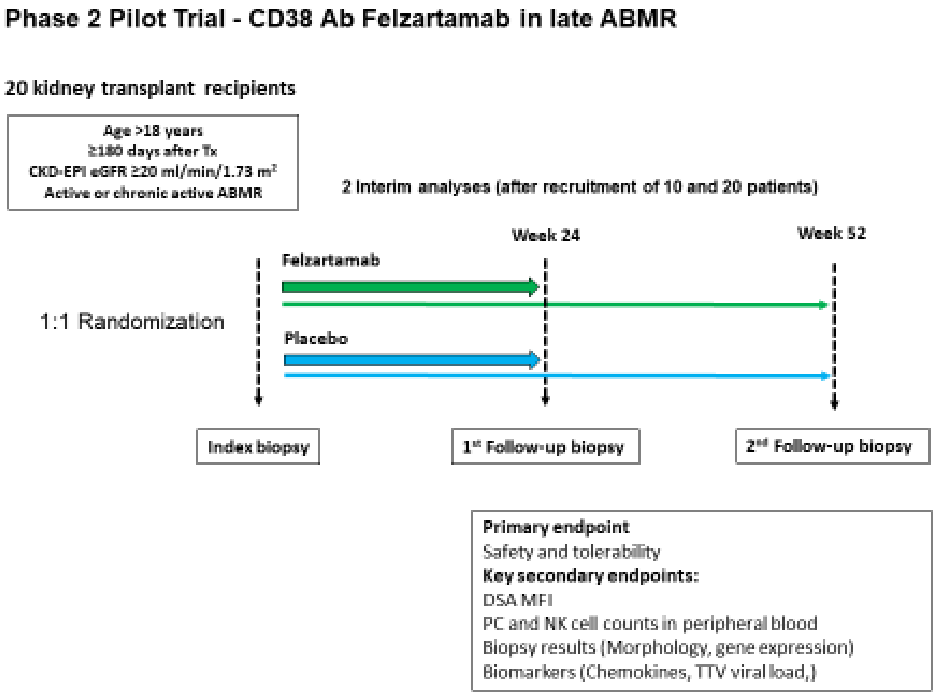

Participants will be randomized to receive either felzartamab (16 mg/kg, intravenous [IV] administration) or placebo (1:1 randomization) for a period of 6 months (administration of felzartamab/placebo at day 0, 7, 14, 21 (cycle 1), and thereafter in 4-weekly intervals at weeks 4, 8, 12, 16, and 20 (cycles 2-6). After six (week 24) and twelve months (week 52), study participants will be subjected to follow-up allograft biopsies. Primary goals of the trial are to assess the safety, pharmacokinetics and pharmacodynamics (peripheral blood PC and NK cell depletion) of a 6-month course of treatment over a period of 12 months. The trial will in addition provide first data on efficacy (progression/activity of rejection, blood biomarkers) and potential associations of treatment with parameters reflecting clinical progression of allograft dysfunction (course of renal function [32] or iBOX score [33]). We expect completion of patient recruitment after 12-18 months and a total study duration of 24-30 months (12 months follow-up period).

4.2. Inclusion/exclusion criteria

Inclusion and exclusion criteria are listed in Table 2. We plan to include 20 kidney transplant recipients with circulating anti-HLA DSA and biopsy features of late (≥180 days post-transplant) active ABMR (ABMR according to the Banff 2019 scheme) in an indication biopsy (index biopsy; performed within the clinical routine for a positive post-transplant DSA result and slow deterioration of allograft function and/or proteinuria). Other key inclusion criteria are an age >18 years, a functioning graft at ≥180 days post-transplantation and an estimated GFR (eGFR according to the CKD-EPI equation) ≥20 ml/min/1.73 m^2. Exclusion criteria are detailed in Table 2.

Table 2. Inclusion and exclusion criteria.

**INCLUSION CRITERIA**
Voluntary written informed consent
Age >18 years (maximum: 80 years)
Functioning living or deceased donor allograft after ≥180 days post-transplantation
eGFR ≥20 ml/min/1.73 m^2 (CKD-EPI formula)
HLA class I and/or II antigen-specific antibodies (preformed and/or *de novo* DSA).
Active or chronic/active ABMR (±C4d in PTC) according to the Banff 2019 classification
Molecular ABMR score (MMDx) ≥0.2
**EXCLUSION CRITERIA**
Patients actively participating in another clinical trial
Age ≤18 years
Female subject is pregnant or lactating or not on adequate contraceptive therapy
ABO-incompatible transplant
Index biopsy results:
T-cell-mediated rejection classified Banff grade ≥I
De novo or recurrent severe thrombotic microangiopathy
Polyoma virus nephropathy
De novo or recurrent glomerulonephritis
Acute rejection treatment ≤3 month before screening
Previous treatment with other CD38 monoclonal antibodies (e.g. daratumumab)
Previous treatment with other immunomodulatory monoclonal/polyclonal antibodies (e.g. CD20 Ab rituximab, IL-6/IL-6R Ab) ≤3 months before study treatment
Total bilirubin >2×the upper limit of normal [ULN], alanine transaminase and aspartate aminotransferase >2·5×ULN
Haemoglobin <8 g/dL
Thrombocytopenia: Platelets <100 G/L
Leukopenia: Leukocytes <3 G/L
Neutropenia: Neutrophils < 1.5 G/L
Hypogammaglobulinemia: Serum IgG <400 mg/dL
Active viral, bacterial or fungal infection precluding intensified immunosuppression
Active malignant disease precluding intensified immunosuppressive therapy
Latent or active tuberculosis (positive QuantiFERON-TB-Gold test)
Administration of a live vaccine within 6 weeks of screening
History of alcohol or illicit substance abuse
Serious medical or psychiatric illness likely to interfere with participation in the study

4.3. Random allocation

Subjects eligible for study inclusion will be randomized in a 1:1 ratio to one of the two study arms (felzartamab versus placebo) by computer assignment using a web-based randomization platform (www.meduniwien.ac.at/randomizer). Patients will be randomized after completion of the screening phase and after informed consent has been obtained, at the day of study initiation. Randomization will be stratified by study site and according to ABMR categories (active ABMR versus chronic/active ABMR) to ensure a balance of patients with these two histological types between the two arms. For each patient, a study ID will be assigned. The trial will be carried out in a double-

blind fashion. Individual roles of investigators, study nurses and clinical pharmacists will be defined within the online tool: study participants, care providers, and those assessing outcomes will be unaware of the randomization sequence, being blinded to group allocation until the completion of the study. The allocation sequence will be generated and medication/placebo will be prepared by independent study pharmacists (Vienna: Department of Clinical Pharmacology). Study physicians and nurses will be provided with blinded intravenous and oral medication. For included patients, treatment code envelopes will be securely stored on site, and in case of emergency, the respective code envelope can be opened.

4.4. Blinding and unblinding

The study is designed as a double-blinded trial, in order to minimize bias. Investigational drug (felzartamab) and placebo (normal 0.9% saline) are prepared by non-blinded pharmacists and formulated to have identical appearance. Neither study participants nor medical staff interacting with patients or data know the assigned treatment (felzartamab versus placebo). The randomization sequence will be unblinded by a dedicated clinical pharmacist (according to his/her role defined in the randomization tool) after the last patient has completed the trial. Premature unblinding (treatment code envelopes) may be necessary in cases of medical emergencies or serious medical conditions, where participants cannot be treated adequately unless the medical staff knows the allocated treatment, or reports of suspected unexpected serious adverse events. Unblinding can, if necessary, be requested by the data and safety monitoring board (DSMB).

5. INTERVENTIONS

5.1. Treatment with felzartamab/placebo

Based on the results of a PK modelling for an ongoing phase Ib/IIa trial in autoimmune disease (membranous nephropathy; ClinicalTrials.gov Identifier: NCT04145440; see investigator brochure), patients will be dosed with felzartamab for a period of 6 months, administered as an intravenous infusion. As transplant patients are on multi-compound immunosuppressive baseline therapy, and therefore at increased risk of infections, we plan a reduction in doing intervals after the first cycle. 9 doses of Felzartamab will be administered as an intravenous infusion at 16 mg/kg over 6 treatment cycles at 28-

days each. Dosing occurs every week in cycle 1 (C1) and every four weeks in cycles 2-6. The first two infusions of felzartamab will be slow (approximately 90 min), and, if no infusion reactions occur, infusion times may be shortened to 1 hour or shorter (minimum 30 min) in subsequent infusions. In case of infusion reactions, the infusion is stopped temporarily or entirely depending on the severity. In some cases, premedication may be extended to subsequent infusions. The infusion rate for resuming felzartamab application is slower.

Felzartamab (MOR202; MOR03087) will be supplied and provided by MorphoSys [One vial contains 325 mg felzartamab (lyophilizate); nominal concentration/composition after reconstitution with 4.8 mL water for injection: 65 mg/mL felzartamab in 10 mM L-histidine, 260 mM sucrose, 0.1% polysorbate 20, pH 6.0.]. The reconstituted solution (65 mg/mL) should be used immediately for further dilution (storage prior to dilution for up to 8 hours at 2°C to 25°C). Felzartamab will be administered after dilution with 250 mL 0.9% sodium chloride solution (final concetration should be between 1 and 20 mg/mL). Prepared infusions may be stored at room temperature for up to 16 hours (concentration 1-10 mg/mL) or 8 hours (10-20 mg/mL), respectively (this includes also the duration of infusion). The prepared infusions will be protected from light. Prior to administration, felzartamab/placebo infusion must reach room temperature by storing unrefrigerated for 30 to 60 minutes before use. The placebo medication will be administered with 250 mL normal saline for infusion (commercially available in country) and will be provided by the Investigator.

Storage and Accountability. Felzartamab (lyophilisate) will be stored at 2°C to 8°C, with protection from light. Placebo will be stored at controlled room temperature and should not be frozen. Study product disposition and accountability will be documented on the subject level, whereby all study product dispositions will be listed (drug accountability log). In a stock record, overall bulk study product supplies and accountability will be recorded.

5.2. Premedication

To prevent infusion-related reactions, patients allocated to the felzartamab arm will receive intravenous premedication prior to the first two felzartamab infusions (day 0 and day 14). Patients in the placebo arm will receive placebo (0.9% NaCl solution).

Premedication will be administered 30 min before the infusion of felzartamab, and will consist of Diphenhydramine (30 mg), Paracetamol (1000 mg), and Prednisolon (100 mg), respectively (each in 100 mL Volume). In the placebo arm, patients will receive 3x100 mL NaCl 0.9%.

5.3. The following medications are prohibited during the study:

Rituximab, eculizumab, proteasome inhibitors, IVIG, plasma exchange or immunoadsorption, other investigational drugs/treatments including commercially available CD38 or anti-IL-6/sIL-6R monoclonal antibody drugs such as daratumumab (Darzalex®) or tocilizumab (RoActemra®).

5.4. The following concomitant medications are permitted during the study:

Calcineurin inhibitors (CNI, tacrolimus or cyclosporine A), mammalian target of rapamycin (mTOR) inhibitor (everolimus or rapamycin), mycophenolate mofetil (MMF)/mycophenolate sodium; long-term treatment with low dose corticosteroids (prednisolone 5mg/day).

5.5. Baseline immunosuppression

Upon diagnosis of late ABMR, all recipients on therapy with a calcineurin inhibitor [tacrolimus or cyclosporine A (CyA)] or a mTOR inhibitor (everolimus or rapamycin), without azathioprine or mycophenolic acid (MPA), will receive mycophenolate mofetil (or, alternatively, enteric-coated mycophenolic acid (EC-MPA), initially at a dose of 2 x 500 mg (or 2 x 360 mg, respectively) per day; stepwise increase to 2 x 1000 mg (or 2 x 720 mg) per day if tolerated) to avoid under-immunosuppression. Tacrolimus will be adjusted to achieve target trough levels between 5 and 10 ng/mL, CyA to 80-120 ng/mL. Recipients weaned off steroids will receive low dose prednisolone (5 mg/day).

6. OUTCOME MEASURES

Study endpoints are listed in Table 3.

Table 3

**PRIMARY OUTCOME**
**Safety and tolerability** of felzartamab in renal allograft recipients with ABMR on baseline immunosuppression
**SECONDARY OUTCOMES**
**DSA/immunoglobulin levels (week 0, 12, 24, and 52)**<br>Mean fluorescence intensity (MFI) of immunodominant DSA<br>Changes in levels of the immunodominant DSA calculated from dilution experiments<br>Number of detected DSA<br>Total Ig (IgG, IgA, IgM) and IgG subclasses (IgG1, IgG2, IgG3, IgG4)<br>Course of vaccination titers<br>**Effect on leukocyte subsets in peripheral blood (week 0, 1, 4, 8, 12, 24, and 52)**<br>Circulating PC, NK cells, T and B cell subpopulations, expression of CD38 using non-crossreactive CD38 Ab clone HIT2<br>**Results of follow-up protocol biopsies (week 24 and 52)**<br>***Morphological results***:<br>ABMR category (active vs. chronic active ABMR; C4d+ vs. C4d- ABMR)<br>Extent of glomerular/pertibulular capillary microcirculation inflammation (g+ptc score)<br>Transplant glomerulopathy (cg) and interstitial fibrosis/tubular atrophy scores<br>Intragraft complement activation<br>Patterns of intragraft cellular infiltrates (NK cells, PC, T cells, B cells)<br>***Gene expression analysis*** (Molecular Microscope® Diagnostic System, MMDx)<br>Molecular classifiers/scores related to ABMR and T cell-mediated rejection<br>Molecular classifiers/scores related to rejection in general<br>Molecular classifiers/scores related to acute and chronic renal injury<br>Archetypal analysis of rejection-related categories<br>Pathogenesis-based transcripts (PBT) scores (cytotoxic T cell infiltration, γ-interferon effects, NK cell burden, epithelial cell damage)<br>**Effect on immunologic biomarkers (week 0, 12, 24, and 52**)<br>CXCL9 and CXCL10 levels in blood and urine (Luminex-based detection)<br>BAFF levels in blood (ELISA/Luminex)<br>**Effect on measures of overall immunosuppression (week 0, 12, 24, and 52)**<br>Torque Teno virus (TTV) levels in plasma (quantitative PCR)<br>**Effect on clinical outcome parameters and surrogate endpoints:**<br>eGFR slope over a period of 52 weeks (4-weekly measurements)<br>iBox clinical prediction score at, 24 and 52 weeks<br>Protein excretion (protein/creatinine ratio) over 52 weeks (4-weekly measurements)<br>12-month (death-censored and overall) graft and patient survival

A schedule of events is provided in Table 4.

Table 4

**Visit**	**Screen**	**1**	**2**	**3**	**4**	**5**	**6**	**7**	**8**	**9**	**10**	**11**	**12**	**13**	**14**	**15**	**16**	**17**
**Week**	**up to -4**	**0**	**1**	**2**	**3**	**4**	**8**	**12**	**16**	**20**	**24**	**28**	**32**	**36**	**40**	**44**	**48**	**52**
Informed consent	x																	
Physical examination, medical history	x	x									x							x
Concomitant medications	x	x	x	x	x	x	x	x	x	x	x	x	x	x	x	x	x	x
Pregnancy test, if applicable	x					x	x	x	x	x	x	x	x	x	x	x	x	x
Quantiferon test	x																	
Virology	x																	
ECG	x										x							x
**Felzartamab/placebo**		x	x	x	x	x	x	x	x	x								
**Safety**																		
Temperature/heart rate/blood pressure		x	x	x	x	x	x	x	x	x	x	x	x	x	x	x	x	x
AE monitoring		x	x	x	x	x	x	x	x	x	x	x	x	x	x	x	x	x
Safety Lab		x	x	x	x	x	x	x	x	x	x	x	x	x	x	x	x	x
Cov-19 Test/nasal swab		x	x	x	x	x	x	x	x	x	x	x	x	x	x	x	x	x
Ig(sub)classes		x				x	x	x	x	x	x		x		x			x
CMV/HSV/HZV PCR		x				x	x	x	x	x	x		x		x			x
Vaccination titers		x						x			x							x
TTV copy number		x						x			x							x
**PK and Immunogenicity**																		
Felzartamab serum concentration		x^2	x^2	x^2	x^1	x^1	x^1	x^1	x^1	x^1	x	x	x	x	x	x	x	x
Anti-felzartamab antibodies		x^1	x			x^1		x^1			x			x				x
**Preliminary efficacy assessment**																		
Follow-up biopsies (incl. MMDx)											x							x
DSA detection		x						x			x							x
Chemokines		x						x			x							x
BAFF levels		x	x	x		x		x			x							x
Gene expression analysis		x						x			x							x
Cell-free donor-derived DNA		x						x			x							x
Leukocyte subpopulations		x	x	x		x		x			x							x
eGFR (CKD-EPI equation)		x	x	x	x	x	x	x	x	x	x	x	x	x	x	x	x	x
Protein/creatinine ratio		x	x	x	x	x	x	x	x	x	x	x	x	x	x	x	x	x
iBOX score		x						x			x							x
**Immunosuppression**																		
CNI/mTOR inhibitor trough level		x	x	x	x	x	x	x	x	x	x	x	x	x	x	x	x	x
**Biobanking** (Plasma, plasma in Streck tubes, serum, blood RNA, urine)		x						x			x							x

Legend:

**Procedure**	**Details**
**Pregnancy Test**	▪ ß- humane Choriongonadotropin, Urine-test
**Quantiferon Test**	▪ QuantiFERON-TB-Gold test, Quiagen
**Virology**	▪ HIV Ab 1+2, HbsAg, HbsAb, HbcAb, HCVAb
**CMV, HSV, HZV PCR**	▪ Real-time PCR for quantification of viral load
**Vaccination titers**	▪ HBs Ab, mumps, measles and rubella Ab; Covid-19 titers
**Torque Teno virus (TTV) quantification**	▪ TTV PCR, Plasma samples
**Covid-19**	▪ Antigen test, if positive, confirmation by PCR assay
**Safety lab**	haemoglobin, erythrocyte count, MCV, white blood cell count and differential (neutrophils, bands, lymphocytes, monocytes, eosinophils and basophils), platelets, PT, PTT, sodium, potassium, calcium, phosphate, magnesium, urea, creatinine, total protein, C-reactive protein, creatinine kinase, venous blood gas analysis, albumin, ALT (SGPT), AST (SGOT), alkaline phosphatise, γGT, LDH, total bilirubin, LDL, HDL, cholesterol, triglycerides, uric acid, glucose, total amylase, TSH
**PK and Immunogenicity**	x^1: pre-dose only; x^2: pre-dose and 30 min after end of infusion
**Gene Expression**	▪ PAXgene Blood RNA tubes
**Chemokines, BAFF**	▪ CXCL9, CXCL10 and BAFF using multiplex beads on a Luminex 200 reader, or ELISA technique
**Cell-free donor-derived DNA**	▪ Assessment of the proportion of cf-dd DNA in plasma samples by next-generation sequencing
**Leukocyte subpopulations**	▪ DuraClone®; flow cytometry based immune phenotyping
**iBox risk prediction score**	▪ Integrative system to predict long term kidney allograft failure

Analysis of safety and tolerability will be the primary endpoint. Major secondary endpoints include the course of DSA (and in parallel total Ig and IgG subclass levels), the dynamics of peripheral blood counts of PC, NK cells, and T and B cell subpopulations (Duraclone multicolor flow cytometry; panels: phenotyping/basic, T cell subsets, Treg panel, B cell panel, PC panel), as well as biomarkers of rejection (Chemokine (C-X-C motif) ligand (CXCL)9 and CXCL10 in blood and urine), B cell immunity (BAFF) and overall immunosuppression (Torque Teno viral load [34]). Moreover, 6- and 12-month renal allograft biopsies will be assessed for morphological (Banff criteria of rejection and chronic injury; immunohistochemistry for detection of complement activation/deposition and characterization of cellular infiltrates including NK cells) and molecular rejection criteria (molecular ABMR score; microarray analysis using the Molecular Microscope® Diagnostic System, MMDx [35]), including pathogenesis-based transcripts (PBT) scores (cytotoxic T cell infiltration, γ-interferon effects, natural killer cell burden, epithelial cell damage). Clinical endpoints will be proteinuria as well as the slope of eGFR and the iBox clinical prediction score, both validated surrogate endpoints that accurately predict long-term allograft survival [32,33].

7. METHODOLOGY

7.1. HLA antibody detection

For assessment of the course of HLA antibody levels, serum samples will be evaluated after completion of the study according to published protocols [18]. For antibody detection, LABscreen single-antigen flow-bead assays (One Lambda, Canoga Park, CA) will be applied. Serum samples will be incubated with ethylenediaminetetraacetic acid (EDTA, 10 mM) to prevent complement interference [36]. Data acquisition will be performed via a LABScan™ 200 flow analyzer (Luminex Corporation, Austin, TX, USA). For longitudinal analysis of DSA/HLA antibody levels, bead assays will be performed retrospectively (centralized analysis) to avoid influences of day-by-day variations in test results (test batches including samples from 4-6 patients each). Donor-specificity will be defined according to serological and/or low- or high-resolution donor/recipient HLA typing (HLA-A, -B, -Cw, -DR, -DQ, -DP upon availability) provided either by the local HLA lab or the Eurotransplant database. Test results will be documented as mean fluorescence intensity (MFI) of the immunodominant DSA. An MFI threshold >1,000 will be considered as positive. To estimate the impact of

felzartamab treatment on DSA levels, we will document the percent change in MFI. In an effort to quantify changes in DSA levels more accurately, we plan to additionally perform dilution experiments following an earlier described protocol [26]. In brief, nonlinear standard curves based on raw DSA MFI levels (immunodominant DSA) will be obtained by serial dilution of individual patient sera collected prior to start of treatment (all samples were incubated with EDTA) and at week 24. According to computed standard curves, the fold change of antibody levels will then be calculated from DSA MFI levels detected in the same experiment for undiluted week-12, -24 and week-52 samples.

7.2. Immunoglobulin levels

Total IgG, IgM, IgA and IgG subclasses will be assessed in serum applying immunonephelometry on a BN™ II analyzer (Siemens Healthineers, Erlangen, Germany).

7.3. Pharmacokinetics and Immunogenicity

For the determination of felzartamab serum concentrations (PK) and the potential development of anti-falzartamab antibodies (ADA), serum sample will be collected throughout the course of the study (i.e. 2 x 1 mL serum per parameter and collection time point labelled as A- and B-samples). The assessment will be performed with drug specific validated ligand binding assays (ELISA and MSD based). The actual dates and times of each PK and ADA blood sampling will be recorded in the CRF.

7.4. Transplant biopsies

Follow-up biopsies will be performed at weeks 24 and 52 (end-of-study visit), after exclusion of a coagulation disorder or platelet counts below 80%. Anticoagulants or inhibitors of thrombocyte aggregation will be transiently paused. The biopsy will be performed under local anaesthesia (lidocain) using ultrasound-guided percutaneous techniques (1-2 cores per biopsy, 16 gauge needle). After biopsy, patients will be monitored closely for 5 to 8 hours for any complications (serial blood pressure measurements, monitoring for hematuria, haemoglobin check 4 hours after biopsy). Histomorphology will be evaluated on paraffin-embedded sections applying standard methodology. For immunohistochemical C4d staining, we will use a polyclonal anti-C4d antibody (BI-RC4D, Biomedica; Vienna, Austria), and, following the rules of the

Banff scheme [37], minimal immunohistochemical staining (C4d Banff score ≥1) along peritubular capillaries will be considered positive. Biopsies will also be evaluated by electron microscopy for detection of multilayering of peritubular capillary basement membranes (MLPTC). Morphological results will be read locally (Medical University of Vienna, Charité Universitätsmedizin Berlin) in a blinded fashion. At the end of the trial, biopsies will be scanned and re-evaluated by a central pathologist (Heinz Regele, Medical University of Vienna). In addition, all biopsies will also be analyzed using microarrays as also proposed by the Banff scheme, using the internationally validated Molecular Microscope® Diagnostic System MMDx platform [35,38], For each biopsy, a 3 mm portion of one core will be placed immediately in RNAlater, stored at -20°C and shipped at ambient temperature or dry ice to the Alberta Transplant Applied Genomics Centre (ATAGC, University of Alberta, Edmonton, AB, Canada) for gene array analysis. Thoroughly validated molecular scores based on machine-learning derived lesion-based classifiers related to rejection [ABMR, T cell-mediated rejection (TCMR), all Rejection], inflammation (global disturbance score) or chronic injury (atrophy/fibrosis score) will be generated using a reference set of 1529 biopsies. Moreover, gene expression patterns will be evaluated using unbiased archetype analysis [39]. For classification of ABMR according to the Banff 2019 scheme, all biopsy results will be analyzed in the context of the molecular results. ABMR categories and morphological single lesions will be defined and scored following the 2019 update of the Banff classification [37]. ABMR will be defined on the basis of both morphological (histomorphology, immunohistochemistry, electron microscopy) and thoroughly validated molecular criteria: (i) evidence of acute or chronic tissue injury, (ii) evidence of current/recent antibody interaction with the vascular endothelium, and (iii) serological evidence of DSA.

7.5. Kidney function

eGFR will be assessed using the Chronic Kidney Disease Epidemiology Collaboration (CKD-EPI) equation (mL/min/1.73m^2). Protein excretion will be documented as protein/creatinine ratio in spot urine (mg/g).

7.6. Immunologic biomarkers

For chemokine detection we will use Luminex-based protocol as described earlier [40]. For quantification of *chemokine (C-X-C motif) ligand (CXCL)9 and CXCL10*, serum samples will be adjusted to 10 mM EDTA to prevent complement interference. Undiluted samples will be measured in duplicates using multiplexed Human ProcartaPlex Simplex Immunoassays (Thermo Fisher Scientific, Waltham, MA, USA) according to the manufacturer's instructions. Immunoassays will be performed on a Luminex 200 instrument (Luminex Corp., Austin, Tx, USA). Urinary results will be normalized to creatinine excretion and presented as pg (chemokine)/mg (creatinine). Levels of dd-cf DNA in recipient plasma samples reflecting the extent of ongoing allograft injury will be detected using standard technology, based on the detection of a defined set of single nucleotide polymorphisms (SNPs) detected by NGS (next-generation sequencing) on an Illumina MiSeq sequencer (Illumina Inc, San Diego, CA, USA) [41]. BAFF levels will be detected using Luminex (or alternative ELISA technology) using commercial kits.

7.7. Leukocyte subpopulations

The underlying mechanisms of chronic antibody mediated rejection, especially the role of peripheral T- and B-cell subsets are not fully clarified. Thus, the prospective monitoring of immune phenotype under therapy with felzartamab is a promising approach to further elucidate the impact on immune-regulatory pathways when CD38 is targeted. Moreover, assessment of PC and NK cell counts allows for the monitoring of the pharamacodynamic effects of the CD38 antibody. For monitoring of leukocyte (sub)populations we will use reproducible immune monitoring (IM) panels for phenotyping. Recently, the international "The ONE study" consortium has designed a standardized panel (DuraClone®) for flow cytometry based immune phenotyping that demonstrated robust results [42,43]. In the DuraClone IM kits pre-defined assay tubes contain a layer with the dried-down antibody panel ready to use. Up to 10 different monoclonal antibodies per tube allows the identification of leukocyte (e.g. T cell, B cell, NK cell subsets) subpopulations present in whole blood samples. For flow cytometric assessment of CD38 expression, assays will be modified to include staining with non-crossreactive CD38 mAb.

7.8. Gene expression analysis

For gene expression analysis, 5 mL of blood will be collected in PAXgene Blood RNA tubes and stored at -80°C until retrospective analysis. These tubes are designed for stabilization of RNA in blood during long-term storage at ultra-low temperature. Gene expression pattern analyses (microarray analysis) will be performed from peripheral blood to evaluate the impact of felzartamab on antibody-producing cells, analyzing genes annotated as part of the B-cell receptor signalling pathway [44].

7.9. Torque Teno virus (TTV) quantification

For TTV analysis, DNA will be extracted from plasma using the NucliSENS easyMAG platform (bioMeriéux, France) and eluted in 50 µL of elution buffer. TTV DNA will be quantitated by TaqMan real time polymerase chain reaction (PCR), according to earlier described protocols [34,45,46]. The quantitative PCR reactions will be performed in a volume of 25 µL using 2 × TaqMan Universal PCR Master Mix, containing 5 µL of extracted DNA, 400 nM of each primer, and 80 nM of the probe. Thermal cycling will be started for 3 minutes at 50°C, followed by 10 minutes at 95°C, and then by 45 cycles at 95°C for 15 seconds, at 55°C for 30 seconds, and at 72°C for 30 seconds, using the CFX96 Real-time System (Bio-Rad, Hercules, CA). Results will be recorded as copies/mL.

7.10. Course of vaccination titers

Serum IgG titers specific for mumps, measles and rubella (MMR) will be analyzed by ELISA technique. Covid-19 antibody titers will be detected using commercial kits.

7.11. Collection of biological material (outside routine monitoring)

Upon screening, we will perform a Quantiferon test and asses HIV/hepatitis serology (1x15 mL). Plasma (10 mL blood; chemokines, BAFF levels, TTV load), serum (10 mL blood; HLA antibody studies), whole blood (10 mL blood; flow cytometry, RNA for gene expression analysis) and urine (10 mL) will be collected before study initiation (day 0), after 3, 6 and after 12 months (in total 4x35 mL peripheral blood). BAFF and leukocyte populations will in addition be evaluated at weeks 1, 2, and 4 (3x10 mL blood). For detection of Ig(sub)classes and CMV/HSV/HZV PCR, serum and plasma will be collected every 4 weeks (10 mL blood, total of 10 visits = 10x10 mL peripheral blood). Finally, for measurement of felzartamab concentrations and ADA, serum will be obtained at every study visit (5 mL peripheral blood per visit; first three felzartamab infusions: additional 5 mL after infusion; total of 17 visits = 14 x 5 mL + 3 x 10 mL peripheral blood). Biological material for retrospective evaluation will be aliquoted and stored for testing at the Department of Clinical Pharmacology.

7.12. Responsible Laboratories that are involved into the study

<u>VIETAC LAb</u>
Medical University of Vienna
Department of Medicine III
Waehringer Guertel 18-20
1090 Vienna, Austria

<u>Department of Laboratory Medicine</u>
Medical University of Vienna
Department of Medicine III
Waehringer Guertel 18-20
1090 Vienna, Austria

<u>Institute of Pathology; Medical University of Vienna</u>
Clinical Institute of Pathology
Waehringer Guertel 18-20
1090 Vienna, Austria

<u>Molecular Microscope', MMDx</u>
Alberta Transplant Applied Genomics Centre
Faculty of Medicine & Dentistry
#250 Heritage Medical Research Centre
University of Alberta
Edmonton, Alberta, Canada

<u>Nephrologisches Forschungslabor Charité</u>
Charité Berlin
Department of Nephrology
Chariteplatz 1
10117 Berlin, Germany

<u>HLA Labor, Charité</u>
Charité Berlin
HLA Laboratory
Augustenburger Platz 1
13353 Berlin, Germany

<u>SGS France</u>
90 Avenue des Hauts de la Chaume BP. 28
86281 Saint-Benoît Cedex
France

8. SAMPLE SIZE AND STATISTICAL ANALYSIS

Rationale for sample size

For this pilot study, no exact sample size estimation based on an efficacy test can be performed, because the effect size is unknown (there is no sufficient prior information to base a sample size on). An important aspect of this pilot trial is to evaluate whether there are any safety issues.

In a myeloma study [28] with n=53 patients who have received felzartamab with or without a steroid, the following AE were reported:

Table 5

**AE rates in the MM trial with with observed frequencies of > 10%**	**AE rate (%)**	**AE rates in the MM trial with with observed frequencies of <10%**	**AE rate (%)**
Infusion reaction	32.1	Pneumonia	5.7
Anemia	39.6	Obstipation	7.5
Leucopenia	37.7	CRP elevation	7.5
Fatigue	32.1	Rash	3.8
Thrombocytopenia	18.9	Cramps	9.4
Tachycardia	15.1	Muscle pain	9.4
Headache	22.6		
Diarrhea	22.6		
Nausea	20.8		
Fever	15.1		

Table 6 gives the probability to observe at least 1 AE of a certain type depending on the actual sample size and the probability for an AE.

Table 6

	**N=5**	**N=10**	**N=20**
**Actual Probabiliy of Event, π=1%**			
**Chance of Observing ≥1 Event, P**	0.049	0.096	0.182
**Actual Probabiliy of Event, π=5%**			
**Chance of Observing ≥1 Event, P**	0.226	0.401	0.642
**Actual Probabiliy of Event, π=10%**			
**Chance of Observing ≥1 Event, P**	0.410	0.651	0.878
**Actual Probabiliy of Event, π=15%**			
**Chance of Observing ≥1 Event, P**	0.556	0.803	0.961

Furthermore when the sample size is 10, a two-sided 95% confidence interval for a single proportion will range from 0 to 0.072 when the sample proportion is 0,01. For proportion of 0.05, 0.1 and 0.15 see Table 6.

A preliminary assessment of efficacy outcomes (~~primary and~~ secondary endpoints) will provide first systematic results (including the extent of variation) on the effect of felzartamab on important molecular, morphological, immunological and clinical endpoints (measurement of the capacity of beneficial change). Our study results can be expected to provide a valuable foundation of the design of future trials. Analyses will be conducted according to the intention-to-treat principle.

For qualitative variables (e.g. occurrence of AE in the two treatment groups), absolute (n=) and relative frequencies will be calculated per treatment group. Data will be visualized by bar plots. Such nominal data will be compared using the Fisher's exact test for descriptive purposes. Transplant and patient survival or AE (SAE)-free survival will be evaluated using Kaplan Meier analysis and the log rank test will be applied for group comparisons. For quantitative data (e.g. group comparisons for DSA levels, g+ptc score, IF/TA score, molecular rejection scores, eGFR and protein/creatinine ratio after 3 months), the number of valid observations (n =), mean, standard deviation, standard error, median, minimum and maximum will be calculated for each treatment group and each time point separately. Data will be visualized by boxplots and

histograms. For descriptive purposes only such continuous data will be analysed using parametric or non-parametric (independent/dependent data) tests. These include the Mann Whitney U test or the t-test, as appropriate. For paired data (e.g. difference in IF/TA score between month 0 and month 12), paired t test or Wilcoxon test will be used as appropriate.

Analysis of pharmacokinetics of felzartamab will include a description of the time evolution of antibody/drug concentration. Elimination half-life, Tmax, Cmax, clearance and volume of distribution will be computed using standard software. For some study endpoints, data on variability and center (median) that can be expected also for the present study, are available from a recent interventional randomized controlled trial performed at our unit, which included a similar cohort of kidney transplant recipients diagnosed with late ABMR (BORTEJECT, NCT01873157) [14]. The respective data obtained in study groups are listed in the table below:

Table 7. Median & variability of key endpoints – Results of BORTEJECT

Endpoints	All patients	n	Placebo (n=23)	Bortezomib (n=21)
**HLA DSA after 1 yr**				
Immunodominant DSA	3553 (1012-10365; 163-16284)	39	4360 (1080-10226; 778-16284)	2054 (918-10968; 163-14461)
Sum of DSA MFI	4605 (1119-12009; 273-33855)	39	5410 (1354-13612; 778-28828)	3479 (929-12009; 273-33855)
**Follow-up biopsy (24 mo)**				
g+ptc score	2 (1-5; 0-6)	38	2 (1-5; 0-6)	4 (0-5; 0-6)
IF/TA score	3 (2-5; 0-6)	38	3 (2-4; 0-6)	3 (2-5; 0-6)
Molecular ABMR score	0.61 (0.30-0.89; 0.05-0.99)	37	0.58 (0.24-0.89; 0.08-0.99)	0.77 (0.41-0.90; 0.05-0.97)
**Kidney parameters after 1 yr**				
eGFR, ml/min/1.73 m2	44 (23-72; 15-95)	43	53 (23-81; 15-95)	42 (25-69; 15-89)
Protein/creatinine ratio, mg/g	201 (86-1249; 0-4863)	44	194 (82-959; 0-4863)	208 (100-1427; 0-2972)

GFR trajectories will be analysed using a linear mixed model with eGFR values from 0 to 52 weeks (week 0, 4, 8, 12, 16, 20, 24, 28, 32, 36, 40, 44, 48, 52) as dependent variable. Time and treatment as well as their interaction will be used as fixed effects. Furthermore, patient-specific random effects for intercept and slope will be specified.

If p-values are presented, this is done for exploratory purposes only as this is a pilot study. Therefore only unadjusted p-values are presented and no correction for multiplicity will be applied. For statistical analysis, IBM SPSS Statistics version 24 (IBM

Corporation, Armonk, NY, USA) and SAS version 9.4. (The SAS Institute Inc., Cary, North Carolina, USA) will be applied.

9. INTERIM ANALYSES

The trial will be monitored by an independent data and safety monitoring board (DSMB) to assess the safety and data quality. To timely detect major differences between groups in terms of safety aspects, the board members will be instructed to perform interim analyses after 10 and 20 patients have been randomized and entered the trial (depending on the duration of recruitment period, for interim analyses a complete patient follow-up may not be available). The DSMB will analyze recorded AEs and safety lab results in relation to the randomization sequence, and may consider stopping the trial if the overall pattern of related serious adverse events (SAEs) or changes in safety lab results strongly support a major safety signal. Exact statistical definitions of criteria for premature study termination are not defined.

10. ADVERSE EVENTS

10.1. Felzartamab

Until now, no data obtained in transplant patients on dual or triple immunosuppression are available, so that safety assessment remains a major objective of this pilot trial. Safety data were obtained in a clinical phase 1-2a trial, in a cohort of 91 patients with refractory or relapsing MM [28]. Treatment-emergent adverse events under felzartamab monotherapy or felzartamab combined with dexamethasone are provided in Table 5. The results of combined treatment with pomalidomide and lenalidomide, which may not be relevant for our planned trial, are not included in this Table (full lists of AE are provided in reference 28).

Table 8. Treatment-emergent adverse events (phase 1-2a trial/MM; IB/Table 22)

Incidence[a] (%) All grades					Incidence[a] (%) grade ≥ 3			
**Preferred Term**	**MOR202+ MOR202/ DEX N=53**	**MOR202/ POM/ DEX N=21**	**MOR20 2/ LEN/ DEX N=17**	**Overall N=91**	**MOR202+ MOR202/ DEX N=53**	**MOR202/ POM/ DEX N=21**	**MOR20 2/ LEN/ DEX N=17**	**Overall N=91**
**Blood and lymphatic system disorders**								
Leukopenia[b]	37.7%	85.7%	88.2%	58.2%	13.2%	57.1%	47.1%	29.7%
Neutropenia[d]	24.5%	90.5%	70.6%	48.4%	11.3%	81.0%	58.8%	36.3%
Lymphopenia[c]	30.2%	66.7%	76.5%	47.3%	26.4%	52.4%	64.7%	39.6%
Anemia[f]	39.6%	47.6%	35.3%	40.7%	9.4%	9.5%	17.6%	11.0%
Thrombocytopenia[e]	18.9%	66.7%	64.7%	38.5%	9.4%	23.8%	17.6%	14.3%
**Cardiac disorders**								
Tachycardia	15.1%	4.8%	5.9%	11.0%	0%	0%	0%	0%
**Gastrointestinal disorders**								
Diarrhea	22.6%	52.4%	58.8%	36.3%	0%	9.5%	11.8%	4.4%
Constipation	7.5%	23.8%	64.7%	22.0%	0%	0%	0%	0%
Nausea	20.8%	9.5%	35.3%	20.9%	0%	0%	0%	0%
**General disorders and administration site conditions**								
Fatigue	32.1%	57.1%	35.3%	38.5%	0%	0%	5.9%	1.1%
Pyrexia	15.1%	28.6%	11.8%	17.6%	0%	0%	0%	0%
Oedema peripheral	9.4%	9.5%	23.5%	12.1%	0%	0%	0%	0%
**Investigations**								
C-reactive protein increase[d]	7.5%	14.3%	17.6%	11.0%	0%	4.8%	0%	1.1%
**Infections and infestations**								
Nasopharyngitis	30.2%	19.0%	35.3%	28.6%	0%	0%	0%	0%
Upper respiratory tract infection	20.8%	23.8%	35.3%	24.2%	0%	4.8%	0%	1.1%
Respiratory tract infection	7.5%	42.9%	23.5%	18.7%	0%	19.0%	0%	4.4%
Pneumonia	5.7%	33.3%	17.6%	14.3%	3.8%	28.6%	5.9%	9.9%
Bronchitis	15.1%	4.8%	5.9%	11.0%	1.9%	0%	0%	1.1%
**Injury, poisoning and procedural complications**								
Infusion related reactions[g]	32.1%	4.8%	5.9%	20.9%	1.9%	0.0%	0.0%	1.1%
**Metabolism and nutrition disorders**								
Hypokalemia	15.1%	28.6%	35.3%	22.0%	0%	9.5%	0.0%	2.2%
**Musculoskeletal and connective tissue disorders**								
Muscle spasms	9.4%	23.8%	52.9%	20.9%	0%	0%	0%	0%
Back pain	13.2%	33.3%	23.5%	19.8%	0.0%	0.0%	5.9%	1.1%
Myalgia	9.4%	19.0%	11.8%	12.1%	0%	0%	0%	0%
Pain in extremity	13.2%	4.8%	11.8%	11.0%	0%	0%	0%	0%
**Nervous system disorders**								
Headache	22.6%	9.5%	5.9%	16.5%	0%	0%	0%	0%
Dizziness	11.3%	4.8%	29.4%	13.2%	0%	0%	0%	0%
**Psychiatric disorders**								
Insomnia	15.1%	4.8%	5.9%	11.0%	0%	0%	0%	0%
**Respiratory, thoracic and mediastinal disorders**								
Cough	15.1%	23.8%	29.4%	19.8%	0%	0%	0%	0%
Dyspnoea	15.1%	4.8%	5.9%	11.0%	3.8%	0%	0%	2.2%
**Skin and subcutaneous tissue disorders**								
Rash	3.8%	23.8%	29.4%	13.2%	0%	0%	0%	0%
**Vascular disorders**								
Hypertension	13.2%	23.8%	11.8%	15.4%	9.4%	19.0%	11.8%	12.1%

N: number of patients; [a]Incidence: percentage of patients; [b]Includes White blood cell count decreased / Leukopenia; [c]Includes Lymphocyte count decreased / Natural killer cell count decreased / CD4 lymphocytes decreased / B-lymphocyte count decreased / Lymphopenia; [d]Includes Neutrophil count decreased / Neutropenia; [e]Includes Platelet count decreased / Thrombocytopenia; [f]Includes Red blood cell count decreased / Hemoglobin decreased / Hematocrit decreased / Mean cell volume decreased /Anemia. [g]One infusion-related reaction was related to comedication and was considered to be not related to felzartamab.

The most common grade 3 or higher treatment-emergent AE were hematologic and included lymphopenia (in 36 [39.6%] of 91 patients; whole study, including combinations with pomalidomide and lenalidomide), neutropenia (in 33 [36.3%]

patients), leukopenia (in 27 [29.7%] patients), thrombocytopenia (in 13 [14.3%] patients) and anemia (in 10 [11.0%] patients) which were reported at higher incidences in patients treated with felzartamab plus an immunomodulatory drug than in patients treated with felzartamab with or without dexamethasone. The most common grade ≥3 non-hematologic TEAEs were hypertension (in 11 [12.1%] patients), pneumonia (in 9 [9.9%] patients), respiratory tract infection (in 4 [4.4%] patients), and diarrhea (in 4 [4.4%] patients).Infusion-related reactions were reported in 18 (19.8%) of 91 patients (in one case, infusion-related reaction was related to a concomitant medication / not felzartamab-related) ; infusion-related reactions graded ≥3 in one patient [1.1%]. Infusion-related reactions were less frequent (7%) among patients who received felzartamab in combination with dexamethasone (with or without combinations with lenalidomide or pomalidomide). This might be attributed to a preventive effect of dexamethasone.

10.2. Adverse drug reactions - specific considerations

10.2.1. Felzartamab

Opportunistic Infections and Serious Infections

In the phase 1-2a trial of felzartamab performed in MM patients the rate of serious infection-related AE was considerably low [28]. In addition, large phase 3 trials evaluating daratumumab in MM rates of serious infections did not or only slightly increase under antibody treatment [47,48]. Currently, however, no systematic data are available for the use of CD38 mAb as a rejection treatment in organ transplant recipients on multi-compound baseline immunosuppression, and intensified immunosuppression, in addition to standard dual or triple baseline immunosuppression, can be expected to be associated with an increased infection risk. Hence, a careful patient follow-up will include a close monitoring for infectious complications (bacterial, viral and fungal infections).

Considering a potentially increased risk of viral infections, patients under felzartamab will be regularly monitored for cytomegalovirus (CMV), herpes simplex virus (HSV) and herpes zoster virus (HZV) viremia. Moreover, patients will be monitored for IgG levels and substituted with intravenous immunoglobulin in case of severe hypogammaglobulinemia (IgG below 400 mg/dL). Physicians should exercise caution

when considering the use of felzartamab in patients with a history of recurring infection or with underlying conditions (eg, diabetes) which may predispose patients to infections (see also inclusion/exclusion criteria). Felzartamab should not be administered in patients with active infection. The effects of felzartamab on neutrophil counts, and possibly signs and symptoms of infection should be considered when evaluating a patient for a potential infection. Patients must be instructed to contact their physician immediately when any symptoms suggesting infection appear, in order to assure rapid evaluation and appropriate treatment. If a patient develops a serious infection, administration of felzartamab is to be interrupted until the infection is controlled. The clinician should consider the benefit-risk before resuming treatment with felzartamab.

Hematologic Abnormalities

Decreases in leukocyte (neutrophil and lymphocyte) and platelet counts have been observed following treatment with felzartamab as monotherapy and in combination with dexamethasone and/or immunomodulatory agents [28]. For patients with concomitant medications associated with hematologic toxicity, the reduction or interruption of the suspected medication is recommended prior to modifying felzartamab. Dosing guidance for neutropenia and thrombocytopenia are described in Tables 9 and 10.

Table 9. Neutropenia risk mitigation

ANC (cells/mm^3)	Action
> 1000	Maintain dose (in case of mild neutropenia, reduction of the daily dose of MMF/EC-MPA)
500 – 1000	Interrupt felzartamab dosing.<br>When ANC increases to > 1000, resume treatment
< 500	Discontinue felzartamab; start filgrastim biosimilar.
ANC = absolute neutrophil count	

Table 10. Thrombocytopenia risk mitigation

Platelet count (cells/mm^3)	Action
> 100,000<br>50,000 – 100,000	Maintain dose.<br>Interrupt felzartamab dosing.

	When platelet count increases to > 100,000, resume treatment
< 50,000	Discontinue felzartamab.

Elevated Liver Enzymes and Hepatic Events

Elevations in ALT and AST have been observed during treatment with the study medications. Dosing recommendations for events of elevated liver enzymes are described in **Table 11**.

Table 11. Elevated liver enzymes – risk mitigation

Lab Value	Action
> 1 to 3x ULN	Dose modify concomitant transplant immunosuppressive drugs if appropriate<br>For persistent increases in this range, interrupt felzartamab until ALT/AST have normalized<br>Restart with treatment as clinically appropriate
> 3 to 5x ULN<br>(confirmed by repeat testing)	Interrupt felzartamab dosing until < 3x ULN and follow recommendations above for >1 to 3x ULN<br>For persistent increases > 3x ULN, discontinue felzartamab
> 5x ULN	Discontinue felzartamab

Infusion-related reactions

In the felzartamab MM trial [28], overall, infusion-related reactions to felzartamab were reported in 18 (20%) of 91 patients (grade ≥3: 1.1%). Importantly, all occurred during the first treatment cycle. As detailed above (see also IB and Table 5), a higher proportion of patients in the steroid-free felzartamab monotherapy groups (14 [40%] of 35 patients; ten [32%] of 31 in the felzartamab q2w group and four [100%] of four in the felzartamab q1w group) had infusion-related reactions than those treated in the felzartamab combination groups (four [7%] of 56; two [11%] of 18 in the felzartamab with dexamethasone group, one [5%] of 21 in the felzartamab with dexamethasone plus pomalidomide group, and one [6%] of 17 in the felzartamab with dexamethasone plus lenalidomide group). Notably, in the felzartamab MM trial, rates of infusion-related reactions were reported at lower frequencies than those reported for two other CD38, daratumumab and isatuximab. Moreover, lower rates of such events under combined regimens with dexamethasone may point to a beneficial effect of steroid-based immunosuppression in transplant patients. However, available results reinforce that during the first cycle of felzartamab adequate pre-treatment including steroids is necessary to minimize the risk of infusion-related reactions.

Healthcare professionals administering felzartamab should be trained in the appropriate administrative procedures, be able to recognize the symptoms associated with potential anaphylactic or hypersensitivity reactions, and have the appropriate medication available for immediate use in case of anaphylaxis or hypersensitivity reaction during or after administration of felzartamab. Healthcare professionals should also instruct patients to seek medical attention if they experience symptoms of a hypersensitivity reaction outside of the clinic.

If a patient has symptoms of anaphylaxis or serious hypersensitivity, or requires an interruption of the study drug because of symptoms of anaphylaxis or hypersensitivity, administration of felzartamab must be discontinued permanently. The patient should be treated according to the standard of care for management of the hypersensitivity reaction. A blood sample for the presence of anti-felzartamab antibodies should be obtained. Felzartamab should not be administered to subjects who have had any previous allergic reactions to monoclonal antibodies.

10.2.2. Premedication

Diphenhydramin-hydrochlorid

The administered single dose of dibondrin is low (30 mg) and is far below the maximum permitted daily dose. For diphenhydramin, the following side effects have been described: Diphenhydramin can frequently cause tiredness (≥1/10). Occasionally (≥1/1.000, <1/100) circulatory problems, dizziness, drowsiness, headaches, concentration and coordination disorders, muscle weakness, visual disturbances, increase in intraocular pressure, thickening of the bronchial secretion, a feeling of tightness in the chest, gastrointestinal complaints, impaired bladder function, dryness of the mouth, nose and throat. Rare side effects (≥1/10.000, <1/1.000) are palpitations, allergic skin reactions, skin irritations and light sensitivity of the skin. Changes in the blood count (neutropenia, leukopenia, thrombocytopenia,
Hemolytic anemia) are very rare (<1/10.000).

Paracetamol

The single administration of paracetamol in the planned dosage (1000 mg) rarely leads to side effects. Increases in liver parameters and malaise are rarely observed (1: 1000-1: 10000). Very rare side effects are (below 1: 10000): changes in blood counts

(thrombocytopenia, leukocytopenia, neutropenia, agranulocytosis), allergic reactions (skin reactions, bronchospasm, anaphylactic shock).

Prednisolon

Short-term administration is considered to be unproblematic and usually causes no or only minor side effects. With the planned two administrations of intravenous prednisolone as part of premedication (100 mg each, at an interval of 14 days), the typical side effects known for long-term therapy, such as increased susceptibility to infection, changes in blood cell counts, endocrine disease, sodium retention, weight gain, osteoporosis, muscle atrophy, skin changes, eye diseases, diseases of the gastrointestinal tract (e.g. gastric ulcer, increase in liver parameters) or impaired wound healing, are therefore *not* to be expected. Short-term intravenous administration can occasionally lead to an increase in blood glucose, increases in blood pressure or restlessness or sleep disorders. Allergic reactions are extremely rare.

10.3. Covid-19

In the context of the trial, the study team will adhere to all measures currently prescribed by the authorities with regard to the COVID-19 pandemic, and will ensure that no resources required to combat the pandemic are tied up, that sufficient personnel will be available, and that the participants are not exposed to any additional risk of infection through their participation in the study. As mentioned in Section 10.1, Covid-19 antigen or PCR tests will be carried out at every visit (or before administration of study medication / placebo). In case of a positive test result, the study medication will not be administered, and patient care will follow current recommendations and guidelines.

10.4. Pregnancies and Women of Child Bearing Potential

There are no adequate well-controlled studies in pregnant or lactating women. All subjects of child bearing potential being treated with felzartamab (and their partners) must be informed of this risk, and use highly effective birth control, as defined in the study protocol. Under no circumstances shall felzartamab injection be administered to women known to be pregnant or lactating. All pregnancies must be reported to MorphoSys within 24 hours and in accordance with SAE reporting procedures.

a) Women of childbearing potential and men with partners that are WOCBP must be using a highly effective acceptable method of contraception to avoid pregnancy throughout the study and for 6 months after the last dose of investigation product, in such a manner that the risk of pregnancy is minimized.

   For this study purpose, only following highly effective birth control methods are allowed (based on the CTFG-Working group on Contraception):

   - combined (estrogen and progestogen containing) hormonal contraception associated with inhibition of ovulation
     a) oral
     b) intravaginal
     c) transdermal
   - progestogen-only hormonal contraception associated with inhibition of ovulation:
     a) oral
     b) injectable
     c) implantable
   - intrauterine device (IUD)
   - intrauterine hormone-releasing system (IUS)
   - bilateral tubal occlusion
   - sexual abstinence

b) WOCBP must have a negative serum or urine pregnancy test (minimum sensitivity 25 IU/L or equivalent units of HCG) within 24 hours prior to the start of investigational product.

c) Women must not be breastfeeding.

d) Patients must be willing to use condoms as adequate barrier to minimize the risk for STDs transmission to their sex partner

e) Men must be willing to refrain from sperm donation during study participation and for 6 months after dosing has been concluded.

All subjects of child bearing potential being treated with felzartamab (and their partners) must be informed of this risk, and use highly effective birth control, as defined above. All pregnancies must be reported to MorphoSys within 24 hours and in accordance with SAE reporting procedures.

11. SAFETY ASSESSMENT AND REPORTING

All patients will be evaluated for safety and adverse events will be documented according to the procedures of the investigational site. The investigator will determine the relationship of the events to the administration of the investigational drug felzartamab. All serious adverse events (SAEs) and adverse event of special interest (AESIs) must be reported as described in this section. All nonserious adverse events must be collected as well and documented according to the procedures of the investigational site.

11.1. Safety Assessment

Following predefined safety assessments will be performed:

WOCBP (Women Of Childbearing Potential)

If the patient is a WOCBP, pregnancy tests (blood or urine; minimum sensitivity 25 IU/L of HCG) must be performed within 72 hours before the first infusion of felzartamab. Women of childbearing potential and men with partners that are WOCBP must be using a highly effective acceptable method of contraception to avoid pregnancy (s. section 9.2.). If the patient becomes pregnant, she must be discontinued from felzartamab treatment.

Standard of care

Laboratory tests (safety lab), assessment of vital signs [heart rate, blood pressure, body temperature, (in case of clinical symptoms monitoring of SpO2 and respiratory rate before and in 30 min intervals during and after infusion of the antibody) will be performed as part of routine standard of care as directed by the investigator. Laboratory specimens should be obtained before felzartamab infusion. Additional tests may be ordered if deemed necessary for monitoring the patient’s safety.

Renal transplant function

Renal function will be evaluated at every study visit. Estimated renal function (eGFR) will be determined by the CKD-EPI-formula. Additional surrogate parameter of

transplant function will be assessed via protein excretion at predefined time points (see Table 3).

IgG monitoring

Patients will be monitored for IgG levels in serial intervals (every 4 weeks under felzartamab, every 8 weeks thereafter). In the case of severe hypogammaglobulinemia (IgG <400 mg/dL), treatment with IVIG will be permitted.

Covid-19-Screening

Before study inclusion and at every trial visit (and before administration of study medication/placebo), study participants will undergo Covid-19 antigen or PCR testing to exclude a SARS Cov-2 infection.

Monitoring for viremia

Patients will undergo CMV, HSV and HZV PCR monitoring in serial intervals (every 4 weeks under felzartamab, every 8 weeks thereafter).

Acute Rejection Episode

At every study visit renal transplant function will be evaluated in regard for acute rejection episode. Any clinical suspected rejection will be confirmed or excluded by renal transplant biopsy histology. Rejection will be classified by the actual BANFF classification from 2019. Incidence of biopsy-proven acute rejection will be reported as SAE.

C0 levels of immunosuppressant

At every visit, trough levels of immunosuppressant (CNI) will be measured. Medication of immunosuppressant will be adapted according to the defined target levels of corresponding immunosuppressive drugs. Every change of medication will be documented in the source data. Change of more than 50% of daily dosing under DCV+SOF will be reported as AE.

11.2. Definition of adverse events

An AE is any untoward or unfavorable medical occurrence in a clinical research study participant, including any abnormal sign (e.g. abnormal physical exam or laboratory

finding), symptom, or disease, temporally associated with the participants' involvement in the research, whether or not considered related to participation in the research.

Adverse events <u>include</u>:

- Exacerbation of a pre-existing disease.
- Increase in frequency or intensity of a pre-existing episodic disease or medical condition.
- Disease or medical condition detected or diagnosed after study drug administration even though it may have been present prior to the start of the study.
- Continuous persistent disease or symptoms present at baseline that worsen following the start of the study.
- Lack of efficacy in the acute treatment of a life-threatening disease.
- Events considered by the investigator to be related to study-mandated procedures.
- Abnormal assessments, e.g., ECG and physical examination findings, must be reported as AEs if they represent a clinically significant finding that was not present at baseline or worsened during the course of the study.
- Laboratory test abnormalities must be reported as AEs if they represent a clinically significant finding, symptomatic or not, which was not present at baseline or worsened during the course of the study or led to dose reduction, interruption or permanent discontinuation of study drug.

Adverse events <u>do not include</u>:

- Pre-planned interventions or occurrence of endpoints specified in the study protocol are not considered AE´s, if not defined otherwise (e.g. as a result of overdose)
- Medical or surgical procedure, e.g. surgery, endoscopy, tooth extraction, transfusion. However, the event leading to the procedure is an AE. If this event is serious, the procedure will be described in the SAE narrative.
- Pre-existing disease or medical condition that does not worsen.
- Situations in which an adverse change did not occur, e.g., hospitalizations for cosmetic elective surgery or for social and/or convenience reasons.

- Overdose of either study drug or concomitant medication without any signs or symptoms. However, overdose must be mentioned in the Study Drug Log and reported on an AE form.

11.2.1. Serious Adverse Events (SAEs)

A Serious Adverse Event (SAE) will be defined by the International Conference on Harmonization (ICH) guidelines and WHO GCP guidelines as any AE fulfilling at least one of the following criteria:

- Results in deaths.
- Life-threatening – defined as an event in which the subject was, in the judgment of the investigator, at risk of death at the time of the event; it does not refer to an event that hypothetically might have caused death had it been more severe.
- Requiring subject's hospitalization or prolongation of existing hospitalization – inpatient hospitalization refers to any inpatient admission
- Resulting in persistent or significant disability or incapacity (i.e., a substantial disruption of a person's ability to conduct normal life functions).
- Congenital anomaly or birth defect.
- Is medically significant or requires intervention to prevent at least one of the outcomes listed above.

Life-threatening refers to an event in which the subject was at risk of death at the time of the event. It does not refer to an event that hypothetically might have caused death if it were more severe. Important medical events that may not immediately result in death, be life-threatening, or require hospitalization may be considered as SAEs when, based upon appropriate medical judgment, they may jeopardize the subject and may require medical or surgical intervention to prevent one of the outcomes listed in the definitions above.

Hospitalization – Prolongation of existing hospitalization

Hospitalization is defined as an overnight stay in a hospital unit and/or emergency room. An additional overnight stay defines a prolongation of existing hospitalization.

The following is not considered an SAE and will be reported as an AE only:

- Treatment on an emergency or outpatient basis for an event not fulfilling the definition of seriousness given above and not resulting in hospitalization.

<u>The following reasons for hospitalizations are not considered AEs, and therefore not SAEs:</u>

- Hospitalizations for cosmetic elective surgery, social and/or convenience reasons.
- Standard monitoring of a pre-existing disease or medical condition that did not worsen, e.g., hospitalization for coronary angiography in a subject with stable angina pectoris.
- Elective treatment of a pre-existing disease or medical condition that did not worsen, e.g., hospitalization for chemotherapy for cancer, elective hip replacement for arthritis.

11.2.2. SAEs related to study-mandated procedures

Such SAEs are defined as SAEs that appear to have a reasonable possibility of causal relationship (i.e., a relationship cannot be ruled out) to study-mandated procedures (excluding administration of study drug) such as discontinuation of subject's previous treatment during a washout period, or complication of a mandated invasive procedure (e.g., blood sampling, heart catheterization), or car accident on the way to the hospital for a study visit, etc.

11.2.3. Suspected unexpected serious adverse reactions (SUSARs)

SUSARs are all serious adverse reactions with suspect causal relationship to the study drug that is unexpected (not previously described in the Investigator's brochure) and serious.

11.2.4. Pregnancy

Pregnancy itself is not an AE/SAE; the outcome of a pregnancy can be a SAE. Any pregnancy that occurs during study participation will be reported to the investigator/sponsor. To ensure subject safety, each pregnancy will be reported to the investigator/sponsor immediately. The pregnancy will be followed up to determine outcome (including premature termination) and status of mother and child. Pregnancy complications and elective terminations for medical reasons will be reported as an AE or SAE. Spontaneous abortions will be reported as an SAE. Any SAE occurring in

association with a pregnancy brought to the investigator's attention after the subject has completed the study and considered by the investigator as possibly related to the investigational product, will be promptly reported to the principal investigator/sponsor. In addition, the investigator will attempt to collect pregnancy information on any female partners of male study subjects who become pregnant while the subject is enrolled in the study. Pregnancy information will be reported to the investigator/sponsor as described above.

11.2.5. Adverse events of special interest

Adverse events of special interest (AESI) in this clinical trial should be reported along with their respective symptoms (e.g. hives, chill, and fever for infusion-related reaction) in the same way as described for SAEs (see Section 10.6).

AESIs in this trial are:

- Infusion-related reactions to IMP ≥ grade 3
- Cytokine release syndrome
- Allergic reaction to IMP
- Infections ≥ grade 3
- Neutropenia ≥ grade 3 (< 1.0 G/L)
- Thrombocytopenia ≥ grade 3 (< 50 G/L)
- Major bleeds (defined as critical organ bleed or hemoglobin decrease of more than 20 g/L within 24 h)
- Hepatitis B reactivation

11.3. Severity of adverse events

The severity of clinical AEs will be graded on a three-point scale: mild, moderate, severe, and reported on specific AE pages of the CRF. If the severity of an AE worsens during study drug administration, only the worst intensity should be reported on the AE page. If the AE lessens in intensity, no change in the severity is required. If an AE occurs during a washout or placebo run-in phase and afterwards worsens during the treatment phase, a new AE page will be filled in with the intensity observed during study drug administration.

Mild: Event may be noticeable to subject; does not influence daily activities; the AE resolves spontaneously or may require minimal therapeutic intervention;

Moderate: Event may make subject uncomfortable; performance of daily activities may be influenced; intervention may be needed; the AE produces no sequelae.

Severe: Event may cause noticeable discomfort; usually interferes with daily activities; subject may not be able to continue in the study; the AE produces sequelae, which require prolonged therapeutic intervention.

A mild, moderate or severe AE may or may not be serious. These terms are used to describe the intensity of a specific event (as in mild, moderate, or severe myocardial infarction). However, a severe event may be of relatively minor medical significance (such as severe headache) and is not necessarily serious. For example, nausea lasting several hours may be rated as severe, but may not be clinically serious. Fever of 39°C that is not considered severe may become serious if it prolongs hospital discharge by a day. Seriousness rather than severity serves as a guide for defining regulatory reporting obligations.

11.4. Relationship to study drug

For all AEs, the investigator will assess the causal relationship between the study drug and the AE using his/her clinical expertise and judgment according to the following algorithm that best fits the circumstances of the AE:

11.4.1. Unrelated

- May or may not follow a reasonable temporal sequence from administration of the study product
- Is biologically implausible and does not follow known response pattern to the suspect study drug (if response pattern is previously known).
- Can be explained by the known characteristics of the subject’s clinical state or other modes of therapy administered to the subject.
- Unlikely
  -May or may not follow a reasonable temporal sequence from administration of the study product
  -Is biologically not very plausible

-May be explained by the known characteristics of the subject's clinical state or other modes of therapy administered to the subject.

11.4.2. Related

Possible related

- Follows a reasonable temporal sequence form administration of the study drug.
- May follow a known response pattern to the study drug (if response pattern is previously known).
- Could not be reasonably explained by the known characteristics of the subject's clinical state or other modes of therapy administered to the subject, if applicable.
- Probable
  -Follows a reasonable temporal sequence form administration of the study drug.
  -Follows a known response pattern to the study drug (if response pattern is previously known).
  -other causes for the event are unlikely

Definitely related

- Follows a reasonable temporal sequence form administration of the study drug.
- Follows a known response pattern to the study drug (if response pattern is previously known).
- No other reasonable cause is present.

11.5. Reporting procedures for any AEs

A special section is designated to adverse events in the case report form. The following details must thereby be entered:

- Type of adverse event
- Start (date and time)
- End (date and time)
- Severity (mild, moderate, severe)
- Serious (no / yes)
- Unexpected (no / yes)
- Outcome (resolved, ongoing, ongoing – improved, ongoing – worsening)
- Relation to study drug (unrelated, related)

Adverse events are to be documented in the case report form in accordance with the above mentioned criteria.

11.6. Reporting procedures for SAEs and AESIs

In the event of serious, the investigator has to use all supportive measures for best patient treatment. A written report is also to be prepared and made available to the clinical investigator immediately. SAEs and AESIs should be reported within 24 h. The following details should at least be available:

- Patient number; ID (study identification number)
- Patient: age, sex, ethical origin
- Name of investigator and investigating site
- Period of administration
- The suspected investigational medical product (IMP)
- The adverse event assessed as serious
- Concomitant disease and medication
- Short description of the event:
    - Description
    - Onset and if applicable, end
    - Therapeutic intervention
    - Causal relationship
    - Hospitalization of prolongation of hospitalization
    - Death, life-threatening, persistent or significant disability or incapacity

If applicable, the initial report should be followed by the Follow up report, indicating the outcome of the SAE/AESI.

All SAEs/AESIs will be recorded on a CIOMS Form and submitted to:

SAE/AESI Reporting	Contact Information
MorphoSys Safety Vendor: UBC	MorphosysPV@ubc.com<br>Back-up fax: +44 20 3936 2286

11.7. Reporting procedures for SUSARs

It must be remembered that the regulatory authorities, and in case of SUSARs which could possibly concern the safety of the study participants, also the Institutional Review Board / Independent Ethics Committee (IRB / IEC) are to be informed. Such reports shall be made by the study management and the following details should be at least available:

- Patient number; ID (study identification number)
- Patient: age, sex, ethical origin
- Name of investigator and investigating site
- Period of administration
- The suspected investigational medical product (IMP)
- The adverse event assessed as serious and unexpected, and for which there is a reasonable suspected causal relationship to the IMP
- Concomitant disease and medication
- Short description of the event:
    - Description
    - Onset and if applicable, end
    - Therapeutic intervention
    - Causal relationship
    - Hospitalization of prolongation of hospitalization
    - Death, life-threatening, persistent or significant disability or incapacity

Electronic reporting should be the expected method for reporting of SUSARs to the competent authority.

11.8. The Development Safety Update Report (DSUR)

The DSUR will be provided by the principal investigator at least once a year.

This report will also be presented annually to the Independent Ethics (IEC) and to the competent authorities by the sponsor.

12. STUDY TERMINATION, WITHDRAWAL, REPLACEMENT

12.1. Criteria for withdrawal

Subjects may prematurely discontinue from the study at any time. Premature discontinuation from the study is to be understood when the subject did not undergo EOS examination and / or all pivotal assessments during the study.

Subjects MUST discontinue investigational product (and non-investigational product at the discretion of the investigator) and study participation will be terminated for any of the following reasons:

a) Withdrawal of informed consent (subject's decision to withdraw for any reason)
b) Any clinical adverse event (AE), laboratory abnormality or intercurrent illness which, in the opinion of the investigator, indicates that continued participation in the study is not in the best interest of the subject
c) New onset of pregnancy
d) Instruct WOCBP to contact the investigator or study staff immediately if they suspect they might be pregnant (e.g. missed or late menstrual period) at any time during study participation. Institutional policy and local regulations should determine the frequency of on-study pregnancy tests for WOCBP enrolled in the study.
e) The investigator must immediately notify MorphoSys if a study subject becomes pregnant. The mechanism for reporting pregnancy is described above.
f) Termination of the study by the sponsor.
g) Loss of ability to freely provide consent through imprisonment or involuntary incarceration for treatment of either a psychiatric or physical (eg, infectious disease) illness
h) Inability to comply with protocol
i) By discretion of the investigator
j) Evidence of confirmed hepatic decompensation (Child-Pugh Class B or C, Score > 6)
k) ALT ≥ 5 x baseline OR ≥ 10x ULN AND either total bilirubin > 2x ULN or INR > 2x ULN
l) Platelets count <25,000/mL
m) Any Grade 4 AE or clinical laboratory finding considered study drug related

The only exception to this requirement is when a subject withdraws consent for all study procedures or loses the ability to consent freely (i.e., is imprisoned or involuntarily incarcerated for the treatment of either a psychiatric or physical illness). If a subject was withdrawn before completing the study, the reason for withdrawal must be entered on the appropriate case report form (CRF) page. In all cases, the reason why subjects are withdrawn will be recorded in detail in the CRF and in the subject's medical records. Should the study be discontinued prematurely, all study materials (complete, partially completed and empty CRFs) will be retained.

12.2. Follow-up of patients withdrawn from the study

In case of premature discontinuation after study drug intake, the investigations scheduled for the EOS visit will be performed 28 days after study drug discontinuation. The subjects will be advised that participation in these investigations is voluntary. Furthermore, they may request that from the time point of withdrawal no more data will be recorded and that all biological samples collected in the course of the study will be destroyed.

12.3. Premature termination of the study

The sponsor has the right to close this study at any time. The IEC and the competent regulatory authority must be informed within 15 days of early termination.
The trial or single dose steps will be terminated prematurely in the following cases:

- If the overall pattern of related SAEs or changes in safety lab results strongly supports a major safety signal, then the DSMB may consider stopping the study.
- If the number of dropouts is so high that proper completion of the trial cannot realistically be expected.

13. ETHICAL ISSUES

The study will be conducted in accordance with the principles of the Declaration of Helsinki 2008. Ethical committee approval will be obtained for all aspects of the study. All study participants will be asked to sign the informed consent to participate in the study (patient insurance included).

14. REGULATORY REQUIREMENTS

We will adhere to all the trial-related requirements, Good Clinical Practice (GCP) requirements (ICH GCP), Good Laboratory Practice (GLP) and the applicable regulatory requirements.

15. PERIODIC MONITORING

The designated monitor (Clinical Trials Coordination Centres – Vienna/Berlin) will contact and visit the investigator regularly and will be allowed to have access to all source documents needed to verify the entries in the CRFs and other protocol-related documents provided that subject confidentiality is maintained in agreement with local regulations. It will be the monitor's responsibility to inspect the CRFs at regular intervals according to the monitoring plan throughout the study, to verify the adherence to the protocol and the completeness, consistency and accuracy of the data being entered on them. The monitoring standards require full verification for the presence of informed consent, adherence to the inclusion/exclusion criteria, documentation of SAEs/AESIs and the recording of the main efficacy, safety, and tolerability endpoints. The monitor will be working according to SOPs and will provide a monitoring report after each visit. The investigator will resolve discrepancies of data. Source data will be checked by the monitor (which means 100% SDV).

16. AUDIT AND INSPECTIONS

Upon request, the investigator will make all study-related source data and records available to a qualified quality assurance auditor mandated by the sponsor or to competent authority inspectors. The main purposes of an audit or inspection are to confirm that the rights and welfare of the subjects have been adequately protected, and that all data relevant for assessment of safety and efficacy of the investigational product have appropriately been reported to the sponsor in accordance with applicable legislation.

17. INVESTIGATOR TRAINING

All investigators and their study personnel will receive training regarding the study procedures and GCP/regulations specific to the conduct of clinical trials. This training

will be documented and will take place prior to enrollment and throughout the study as necessary.

18. RECORD RETENTION

Essential documents will be retained as required by the applicable regulatory requirements. An agreement with MorphoSys may be adhered to in addition.

19. STUDY REGISTRATION

The study is planned to be approved by the Austrian regulatory authority (Federal Office for Safety in Health Care, Austrian Agency for Health and Food Safety) and German regulatory authority (The Paul-Ehrlich-Institut, PEI). We plan to register the study to the European Clinical Trials Database (EUDRACT) and a public clinical trial database (http://clinicaltrial.gov). The study protocol will be submitted for publication to *Trials* (http://www.trialsjournal.com/).

20. MODIFICATIONS OF THE PROTOCOL

20.1. Protocol Amendments

Protocol amendments, except where necessary to eliminate an immediate hazard to patients, will be made with the prior approval of MorphoSys. Each applicable Regulatory Authority/IRB/EC/IEC will review and approve amendments prior to their implementation. Regulatory Authority/IRB/EC/IEC approval need not be obtained prior to removal of an immediate hazard to patients.

20.2. Protocol Violations and Deviations

Protocol waivers will not be permitted except where necessary to eliminate an immediate hazard to patients. The Principal Investigator or designee must document and explain in the subject's source documentation any deviation from the approved protocol. A deviation from the protocol is an unintended and/or unanticipated departure from the procedures and/or processes approved by the Sponsor and the IEC and agreed to by the Principal Investigator. Protocol violations and deviations will be documented by the clinical monitor throughout the course of monitoring visits. Principal Investigator will be notified of violations and/or deviations in writing by the monitor. The

IEC will be notified of all protocol violations and deviations according to IEC reporting requirements.

21. REFERENCES

1. Loupy, A, Lefaucheur, C: Antibody-Mediated Rejection of Solid-Organ Allografts. *N Engl J Med,* 379**:** 1150-1160, 2018
2. Budde, K, Dürr, M: Any Progress in the Treatment of Antibody-Mediated Rejection? *J Am Soc Nephrol,* 29**:** 350-352, 2018
3. Böhmig, GA, Eskandary, F, Doberer, K, Halloran, PF: The therapeutic challenge of late antibody-mediated kidney allograft rejection. *Transpl Int,* 32**:** 775-788, 2019
4. Schinstock, CA, Mannon, RB, Budde, K, Chong, AS, Haas, M, Knechtle, S, et al.: Recommended Treatment for Antibody-mediated Rejection After Kidney Transplantation: The 2019 Expert Consensus From the Transplantion Society Working Group. *Transplantation,* 104**:** 911-922, 2020
5. Theruvath, TP, Saidman, SL, Mauiyyedi, S, Delmonico, FL, Williams, WW, Tolkoff-Rubin, N, et al.: Control of antidonor antibody production with tacrolimus and mycophenolate mofetil in renal allograft recipients with chronic rejection. *Transplantation,* 72**:** 77-83, 2001
6. Lederer, SR, Friedrich, N, Banas, B, Welser, G, Albert, ED, Sitter, T: Effects of mycophenolate mofetil on donor-specific antibody formation in renal transplantation. *Clin Transplant,* 19**:** 168-174, 2005
7. Schwarz, C, Mayerhoffer, S, Berlakovich, GA, Steininger, R, Soliman, T, Watschinger, B, et al.: Long-term outcome of belatacept therapy in de novo kidney transplant recipients - a case-match analysis. *Transplant International,* 28**:** 820-827, 2015
8. Fehr, T, Rusi, B, Fischer, A, Hopfer, H, Wüthrich, RP, Gaspert, A: Rituximab and intravenous immunoglobulin treatment of chronic antibody-mediated kidney allograft rejection. *Transplantation,* 87**:** 1837-1841, 2009
9. Billing, H, Rieger, S, Süsal, C, Waldherr, R, Opelz, G, Wühl, E, et al.: IVIG and rituximab for treatment of chronic antibody-mediated rejection: a prospective study in paediatric renal transplantation with a 2-year follow-up. *Transpl Int,* 25**:** 1165-1173, 2012

10. Cooper, JE, Gralla, J, Klem, P, Chan, L, Wiseman, AC: High dose intravenous immunoglobulin therapy for donor-specific antibodies in kidney transplant recipients with acute and chronic graft dysfunction. *Transplantation,* 97**:** 1253-1259, 2014

11. Bachelet, T, Nodimar, C, Taupin, JL, Lepreux, S, Moreau, K, Morel, D, et al.: Intravenous immunoglobulins and rituximab therapy for severe transplant glomerulopathy in chronic antibody-mediated rejection: a pilot study. *Clin Transplant,* 29**:** 439-446, 2015

12. Walsh, RC, Alloway, RR, Girnita, AL, Woodle, ES: Proteasome inhibitor-based therapy for antibody-mediated rejection. *Kidney Int,* 81**:** 1067-1074, 2012

13. Eskandary, F, Jilma, B, Mühlbacher, J, Wahrmann, M, Regele, H, Kozakowski, N, et al.: Anti-C1s monoclonal antibody BIVV009 in late antibody-mediated kidney allograft rejection-results from a first-in-patient phase 1 trial. *Am J Transplant,* 18**:** 916-926, 2017

14. Eskandary, F, Regele, H, Baumann, L, Bong, G, Kozakowski, N, Wahrmann, M, et al.: A randomized trial of bortezomib in late antibody-mediated rejection (BORTEJECT). *J Am Soc Nephrol,* 29**:** 591-605, 2018

15. Moreso, F, Crespo, M, Ruiz, JC, Torres, A, Gutierrez-Dalmau, A, Osuna, A, et al.: Treatment of chronic antibody mediated rejection with intravenous immunoglobulins and rituximab: A multicenter, prospective, randomized, double-blind clinical trial. *Am J Transplant,* 18**:** 927-935, 2018

16. Kulkarni, S, Kirkiles-Smith, NC, Deng, YH, Formica, RN, Moeckel, G, Broecker, V, et al.: Eculizumab therapy for chronic antibody-mediated injury in kidney transplant recipients: a pilot randomized controlled trial. *Am J Transplant,* 17**:** 682-691, 2017

17. Jordan, SC, Ammerman, N, Choi, J, Kumar, S, Huang, E, Toyoda, M, et al.: Interleukin 6: An Important Mediator of Allograft Injury. *Transplantation*, 2020

18. Doberer, K, Duerr, M, Halloran, PF, Eskandary, F, Budde, K, H., R, et al.: A Randomized Trial of Anti-Interleukin-6 Antibody Clazakizumab in Late Antibody-Mediated Kidney Transplant Rejection. *J Am Soc Nephrol*, in press

19. Parkes, MD, Halloran, PF, Hidalgo, LG: Evidence for CD16a-mediated NK cell stimulation in antibody-mediated kidney transplant rejection. *Transplantation,* 101**:** e102-e111. doi: 110.1097/TP.0000000000001586., 2017

20. Yazdani, S, Callemeyn, J, Gazut, S, Lerut, E, de Loor, H, Wevers, M, et al.: Natural killer cell infiltration is discriminative for antibody-mediated rejection and predicts outcome after kidney transplantation. *Kidney International,* 95**:** 188-198, 2019

21. Horenstein, AL, Faini, AC, Morandi, F, Bracci, C, Lanza, F, Giuliani, N, et al.: The Circular Life of Human CD38: From Basic Science to Clinics and Back. *Molecules,* 25, 2020

22. van de Donk, NWCJ, Richardson, PG, Malavasi, F: CD38 antibodies in multiple myeloma: back to the future. *Blood,* 131**:** 13-29, 2018

23. de Weers, M, Tai, Y-T, van der Veer, MS, Bakker, JM, Vink, T, Jacobs, DCH, et al.: Daratumumab, a Novel Therapeutic Human CD38 Monoclonal Antibody, Induces Killing of Multiple Myeloma and Other Hematological Tumors. *The Journal of Immunology,* 186**:** 1840-1848, 2011

24. Overdijk, MB, Verploegen, S, Bogels, M, van Egmond, M, Lammerts van Bueren, JJ, Mutis, T, et al.: Antibody-mediated phagocytosis contributes to the anti-tumor activity of the therapeutic antibody daratumumab in lymphoma and multiple myeloma. *MAbs,* 7**:** 311-321, 2015

25. Overdijk, MB, Jansen, JH, Nederend, M, Lammerts van Bueren, JJ, Groen, RW, Parren, PW, et al.: The Therapeutic CD38 Monoclonal Antibody Daratumumab Induces Programmed Cell Death via Fcgamma Receptor-Mediated Cross-Linking. *J Immunol,* 197**:** 807-813, 2016

26. Doberer, K, Kläger, J, Gualdoni, GA, Mayer, KA, Eskandary, F, Farkash, EA, et al.: CD38 Antibody Daratumumab for the Treatment of Chronic Active Antibody-mediated Kidney Allograft Rejection. *Transplantation,* Publish Ahead of Print, 2020

27. Kwun, J, Matignon, M, Manook, M, Guendouz, S, Audard, V, Kheav, D, et al.: Daratumumab in Sensitized Kidney Transplantation: Potentials and Limitations of Experimental and Clinical Use. *Journal of the American Society of Nephrology,* 30**:** 1206-1219, 2019

28. Raab, MS, Engelhardt, M, Blank, A, Goldschmidt, H, Agis, H, Blau, IW, et al.: MOR202, a novel anti-CD38 monoclonal antibody, in patients with relapsed or refractory multiple myeloma: a first-in-human, multicentre, phase 1-2a trial. *Lancet Haematol,* 7**:** e381-e394, 2020

29. Teiluf, K, Seidl, C, Blechert, B, Gaertner, FC, Gilbertz, KP, Fernandez, V, et al.: alpha-Radioimmunotherapy with (2)(1)(3)Bi-anti-CD38 immunoconjugates is effective in a mouse model of human multiple myeloma. *Oncotarget,* 6**:** 4692-4703, 2015

30. Busch, L, Mougiakakos, D, Buttner-Herold, M, Muller, MJ, Volmer, DA, Bach, C, et al.: Lenalidomide enhances MOR202-dependent macrophage-mediated effector functions via the vitamin D pathway. *Leukemia,* 32**:** 2445-2458, 2018

31. Flamann, C, Busch, L, Mackensen, A, Bruns, H: Combination of lenalidomide and vitamin D enhances MOR202-mediated cytotoxicity of macrophages: It takes three to tango. *Oncotarget,* 10**:** 10-12, 2019
32. Irish, W, Nickerson, P, Astor, BC, Chong, E, Wiebe, C, Moreso, F, et al.: Change in Estimated GFR and Risk of Allograft Failure in Patients Diagnosed With Late Active Antibody-mediated Rejection Following Kidney Transplantation. *Transplantation,* Publish Ahead of Print, 2020
33. Loupy, A, Aubert, O, Orandi, BJ, Naesens, M, Bouatou, Y, Raynaud, M, et al.: Prediction system for risk of allograft loss in patients receiving kidney transplants: international derivation and validation study. *BMJ,* 366**:** l4923, 2019
34. Doberer, K, Schiemann, M, Strassl, R, Haupenthal, F, Dermuth, F, Gorzer, I, et al.: Torque teno virus for risk stratification of graft rejection and infection in kidney transplant recipients-A prospective observational trial. *Am J Transplant,* in press, 2020
35. Halloran, PF, Famulski, KS, Reeve, J: Molecular assessment of disease states in kidney transplant biopsy samples. *Nat Rev Nephrol,* 12**:** 534-548, 2016
36. Schwaiger, E, Wahrmann, M, Bond, G, Eskandary, F, Böhmig, GA: Complement component C3 activation: the leading cause of the prozone phenomenon affecting HLA antibody detection on single-antigen beads. *Transplantation,* 97**:** 1279-1285, 2014
37. Loupy, A, Haas, M, Roufosse, C, Naesens, M, Adam, B, Afrouzian, M, et al.: The Banff 2019 Kidney Meeting Report (I): Updates on and clarification of criteria for T cell– and antibody-mediated rejection. *American Journal of Transplantation***:** ajt.15898, 2020
38. Halloran, PF, Reeve, J, Akalin, E, Aubert, O, Böhmig, GA, Brennan, D, et al.: Real time central assessment of kidney transplant indication biopsies by microarrays: the INTERCOMEX study. *Am J Transplant,* 17**:** 2851-2862, 2017
39. Reeve, J, Böhmig, GA, Eskandary, F, Einecke, G, Lefaucheur, C, Loupy, A, et al.: Precision molecular phenotyping of kidney transplant biopsies using archetypal analysis. *JCI Insight,* 2**:** e94197, 2017
40. Mühlbacher, J, Doberer, K, Kozakowski, N, Regele, H, Camovic, S, Haindl, S, et al.: Non-invasive Chemokine Detection: Improved Prediction of Antibody-Mediated Rejection in Donor-Specific Antibody-Positive Renal Allograft Recipients. *Front Med (Lausanne),* 7**:** 114, 2020

41. Bloom, RD, Bromberg, JS, Poggio, ED, Bunnapradist, S, Langone, AJ, Sood, P, et al.: Cell-Free DNA and Active Rejection in Kidney Allografts. *J Am Soc Nephrol,* 28**:** 2221-2232, 2017

42. Geissler, EK: The ONE Study compares cell therapy products in organ transplantation: introduction to a review series on suppressive monocyte-derived cells. *Transplant Res,* 1**:** 11, 2012

43. Streitz, M, Miloud, T, Kapinsky, M, Reed, MR, Magari, R, Geissler, EK, et al.: Standardization of whole blood immune phenotype monitoring for clinical trials: panels and methods from the ONE study. *Transplant Res,* 2**:** 17, 2013

44. Banham, GD, Flint, SM, Torpey, N, Lyons, PA, Shanahan, DN, Gibson, A, et al.: Belimumab in kidney transplantation: an experimental medicine, randomised, placebo-controlled phase 2 trial. *Lancet,* 391**:** 2619-2630, 2018

45. Maggi, F, Pifferi, M, Fornai, C, Andreoli, E, Tempestini, E, Vatteroni, M, et al.: TT virus in the nasal secretions of children with acute respiratory diseases: relations to viremia and disease severity. *J Virol,* 77**:** 2418-2425, 2003

46. Schiemann, M, Puchhammer-Stockl, E, Eskandary, F, Kohlbeck, P, Rasoul-Rockenschaub, S, Heilos, A, et al.: Torque Teno virus load-inverse association with antibody-mediated rejection after kidney transplantation. *Transplantation,* 101**:** 360-367, 2017

47. Mateos, MV, Dimopoulos, MA, Cavo, M, Suzuki, K, Jakubowiak, A, Knop, S, et al.: Daratumumab plus Bortezomib, Melphalan, and Prednisone for Untreated Myeloma. *N Engl J Med,* 378**:** 518-528, 2018

48. Moreau, P, Attal, M, Hulin, C, Arnulf, B, Belhadj, K, Benboubker, L, et al.: Bortezomib, thalidomide, and dexamethasone with or without daratumumab before and after autologous stem-cell transplantation for newly diagnosed multiple myeloma (CASSIOPEIA): a randomised, open-label, phase 3 study. *Lancet,* 394**:** 29-38, 2019

Study protocol

Version 7.0

Projekttitel (Deutsch)

Sicherheit, Verträglichkeit und Effektivität des monoklonalen CD38 Antikörpers Felzartamab bei später Antikörper-vermittelter Nierentransplantatabstoßung – Eine Phase 2 Pilotstudie

Project title (English)

Safety, Tolerability and Efficacy of Monoclonal CD38 Antibody Felzartamab in Late Antibody-Mediated Renal Allograft Rejection – A Phase 2 Pilot Trial

Vienna, 06.08.2024

**Trial Sponsor**		**Medical University of Vienna (MUW)**<br>Spitalgasse 23, 1090 Vienna, Austria
**Principal investigator**	**MUW**	**Prof. Dr. Georg A. Böhmig**<br>Medical University of Vienna<br>Department of Medicine III<br>Währinger Guertel 18-20<br>1090 Vienna, Austria<br>Phone: +43 1 40400 43630<br>Fax: + 43 1 40400 39302<br>Email: georg.boehmig@meduniwien.ac.at
**Co-Principal Investigator**		**Prof. Dr. Bernd Jilma**<br>Medical University of Vienna<br>Department of Clinical Pharmacology<br>Währinger Guertel 18-20<br>1090 Vienna, Austria<br>Phone: +43 1 40400 29810<br>Fax: + 43 1 40400 29980<br>Email: bernd.jilma@meduniwien.ac.at
**Investigators, MUW (alphabetical order)**		**Anita Borski**<br>Medical University of Vienna<br>Department of Medicine III<br>Währinger Guertel 18-20<br>1090 Vienna, Austria<br>Phone: +43 1 40400 43630<br>Fax: + 43 1 40400 39302<br>Email: anita.borski@meduniwien.ac.at<br>or: anitadumler@googlemailcom<br>**Dr. Konstantin Doberer**<br>Medical University of Vienna<br>Department of Medicine III<br>Währinger Guertel 18-20<br>1090 Vienna, Austria<br>Phone: +43 1 40400 55260<br>Fax: + 43 1 40400 39302<br>Email: konstantin.doberer@meduniwien.ac.at<br>**Dr. Farsad Eskandary**<br>Medical University of Vienna<br>Department of Medicine III<br>Währinger Guertel 18-20<br>1090 Vienna, Austria<br>Phone: +43 1 40400 43630<br>Fax: + 43 1 40400 39302<br>Email: farsad.eskandary@meduniwien.ac.at<br>**Dr. Christa Firbas**<br>Medical University of Vienna<br>Department of Clinical Pharmacology<br>Währinger Guertel 18-20<br>1090 Vienna, Austria<br>Phone: +43 1 40400 29810<br>Fax: + 43 1 40400 29980<br>Email: christa.firbas@meduniwien.ac.at

		**Dr. Alexander Kainz**<br>Medical University of Vienna<br>Department of Medicine III<br>Währinger Guertel 18-20<br>1090 Vienna, Austria<br>Phone: +43 1 40400 43910<br>Fax: + 43 1 40400 43920<br>**Dr. Katharina Mayer**<br>Medical University of Vienna<br>Department of Medicine III<br>Währinger Guertel 18-20<br>1090 Vienna, Austria<br>Phone: +43 1 40400 72520<br>Fax: + 43 1 40400 39302<br>Email: katharina.mayer@meduniwien.ac.at<br>**Prof. Dr. Heinz Regele**<br>Medical University of Vienna<br>Clinical Institute of Pathology<br>Währinger Guertel 18-20<br>1090 Vienna, Austria<br>Phone: +43 1 40400 49730<br>Fax: + 43 1 40400 51930<br>Email: heinz.regele@meduniwien.ac.at<br>**Martina Schatzl**<br>Medical University of Vienna<br>Department of Medicine III<br>Währinger Gürtel 18-20<br>1090 Vienna, Austria<br>Phone: +43 1 40400 43630<br>Fax: + 43 1 40400 39302<br>Email: m.rtinaschatzl@gmail.com<br>**PD Dr. Christian Schörgenhofer**<br>Medical University of Vienna<br>Department of Clinical Pharmacology<br>Währinger Guertel 18-20<br>1090 Vienna, Austria<br>Phone: +43 1 4040029810<br>Fax: +43 1 40400 29980<br>Email:<br>christian.schoergenhofer@meduniwien.ac.at<br>**PD Dr. Markus Wahrmann**<br>Medical University of Vienna<br>Department of Medicine III<br>Währinger Guertel 18-20<br>1090 Vienna, Austria<br>Phone: +43 1 40400 6207<br>Fax: + 43 1 40400 39302<br>Email: markus.wahrmann@meduniwien.ac.at
**Investigators - Second participating center**	**Charité Berlin**	**PD Dr. Eva Schrezenmeier**<br>Charité Berlin<br>Department of Nephrology

		Charitéplatz 1<br>10117 Berlin, Germany<br>Phone: +49 30 450 614 244<br>Fax: +49 30 450 514 002<br>Email: eva-vanessa.schrezenmeier@charite.de<br>**Prof. Klemens Budde**<br>Charité Berlin<br>Department of Nephrology<br>Charitéplatz 1<br>10117 Berlin, Germany<br>Phone: +49 30 450514002<br>Fax: 49 30 450514002<br>e-mail: klemens.budde@charite.de
**Co-Investigator (for 'Molecular Microscope', MMDx)**	**University of Alberta**	**Prof. Dr. Philip Halloran**<br>Alberta Transplant Applied Genomics Centre<br>Faculty of Medicine & Dentistry<br>#250 Heritage Medical Research Centre<br>University of Alberta<br>Edmonton, Alberta, Canada<br>T6G 2S2<br>Phone: (780) 492-6160<br>Fax: (780) 407-7450<br>Email: Halloran-admin@med.ualberta.ca

Summary of the project (Deutsch)

Projekttitel

Sicherheit, Verträglichkeit und Effektivität des monoklonalen CD38 Antikörpers Felzartamab bei später Antikörper-vermittelter Nierentransplantatabstoßung – Eine Phase 2 Pilotstudie

Wissenschaftlicher Hintergrund. Trotz kontinuierlicher Verbesserungen in der Betreuung nierentransplantierter Patienten/Patientinnen hat sich in den letzten Jahren gezeigt, dass das Überleben von Nierentransplantaten in der frühen Phase nach Transplantation zwar verbessert werden konnte, nicht aber das Transplantatüberleben im Langzeitverlauf. Dies konnte auch nicht durch die Implementierung neuer basisimmunsuppressiver Strategien erreicht werden. Eine wesentliche Erkenntnis der letzten Jahre ist, dass immunologische Prozesse (Abstoßungen) eine wesentliche kausale Bedeutung bei chronischen Transplantatverlusten darstellen. Insbesondere Antikörper gegen Spender-Alloantigene (HLA Antigene), möglicherweise in Kombination mit einer Antikörper-vermittelten Aktivierung zellulärer Mechanismen (z.B. Aktivierung von natürlichen Killerzellen) sind ein wesentlicher Trigger für den chronischen Transplantatschaden. Die Antikörper-vermittelte Abstoßung („antibody-mediated rejection“, ABMR) ist heute als eigene Entität gut etabliert, wobei die diagnostischen Kriterien, eine essenzielle Basis für gezielte therapeutische Interventionen, weitgehend definiert sind: Inflammation und morphologische Schäden in der Mikrozirkulation, (nicht obligate) Ablagerung des Komplementspaltprodukts C4d entlang des Transplantat-Endothels, und Nachweis von Antikörpern gegen Spenderantigene („donor-specific antibodies“, DSA). Mit der Definition spezifischer ABMR-Kriterien hat sich nun erwiesen, dass chronische ABMR eine der führenden Ursachen für einen Transplantatschaden, Dysfunktion und letztlich Verlust darstellt. Im Gegensatz zur frühen aktiven ABMR, wo verschiedene anti-humorale therapeutische Strategien, teilweise auch in kontrollierten Studien erprobt wurden, sind therapeutische Optionen für die späte chronische ABMR kaum definiert. Die klinischen Erfahrungen beschränken sich hier weitgehend auf anekdotische Berichte und kleine Fallserien. Erst rezent wurden immunmodulatorische Therapiestrategien, wie terminale

Komplement-Blockade (Eculizumab), oder Proteasom-Inhibition mit dem Ziel einer Plasmazell-Depletion (Bortezomib), in randomisierten Protokollen evaluiert. Allerdings haben diese Studien keinen eindeutigen Effekt solcher Therapien auf den Verlauf chronischer Abstoßungsprozesse ergeben. Es bleibt anzumerken, dass ein weiterer Ansatz, die Antagonisierung der Interleukin-6 (IL-6)/IL-6 Achse derzeit intensiv untersucht wird. Eine aktuelle Pilotstudie unserer Arbeitsgruppe wies dabei auf mögliche günstige Effekte in Hinblick auf Alloantikörper-Produktion und Nierenfunktionsverlauf hin. Allerdings wird diese Therapie, die aktuell bzgl. klinischer Endpunkte und vor allem auch deren Sicherheit und Verträglichkeit (Signal einer erhöhten Infektionsrate und gastrointestinaler Komplikationen in der Pilotstudie) in einer multizentrischen Phase 3 Studie eingehend untersucht wird, für die Routine der Abstoßungstherapie für längere Zeit nicht verfügbar sein. Letztlich steht damit nach wie vor keine effiziente Therapie für die Behandlung der späten chronischen ABMR zur Verfügung, und die optimale Therapie für späte chronische ABMR bleibt weiterhin unklar.

Ein attraktives therapeutisches Target ist CD38, ein 45 kDa Typ II Transmembran-Glycoprotein, welches auf zahlreichen Immunzellpopulationen exprimiert wird, in höchster Dichte auf (normalen und malignen) Plasmazellen und Natürlichen Killerzellen (NK-Zellen). Therapeutische monoklonale CD38 Antikörper haben bereits Eingang in die klinische Routine, z.B. in der Behandlung des Multiplen Myeloms, gefunden, wobei hier die Antikörper Daratumumab und Isatuximab für diese Indikation behördlich in den USA und Europa zugelassen sind. Für die Transplantationsmedizin ist die Möglichkeit einer effektiven Plasmazell-Depletion über CD38 von großem Interesse, da dadurch, wie in rezenten Fallberichten gezeigt werden konnte, eine nachhaltige anti-HLA Antikörper-Reduktion erreicht werden kann. Zudem hat sich gezeigt, dass NK Zellen (mit hoher CD38 Expression) eine tragende Rolle als Effektoren einer Fc Rezeptor-mediierten Transplantatschädigung spielen, was zu einem unmittelbaren Therapieeffekt einer CD38 Bindung beitragen könnte. Tatsächlich konnten wir rezent in einem Fall einer ABMR bei einem nierentransplantierten Patienten, der zudem die Diagnose einer Plasmazellerkrankung hatte (Smoldering Myelom), eine komplette morphologische und klinische Reversion der Abstoßung (und auch des Myeloms), eine effektive Depletion von Plasmazellen und damit Alloantikörpern, sowie eine fast komplette Depletion von NK Zellen im transplantierten Gewebe und peripheren Blut zeigen.

Letztlich bleibt aber der tatsächliche Nutzen durch eine solche Therapie mangels systematischer Daten (momentan sind nur wenige Fallberichte verfügbar) unbewiesen. Wenn auch bei Patienten mit Multiplem Myelom ein günstiges Sicherheitsprofil verschiedener CD38 Antikörper gezeigt werden konnte, gibt es für immunsupprimierte transplantierte Patienten noch keine ausreichenden Daten zu Sicherheit und Verträglichkeit. Dies betrifft nicht nur Erstdosisreaktionen (v.a. Daratumumab) sondern auch aufgrund einer zusätzlichen Immunmodulation ein potenziell erhöhtes Infektionsrisiko.

Ein interessanter Kandidat für eine CD38-gerichtete Antikörpertherapie ist der rekombinante monoklonale voll-humane CD38 Antikörper Felzartamab (MOR202; MOR03087; humanes IgG1λ; Human Immunology Biosciences, Inc. [HI-Bio], South San Francisco, CA, USA). Dieser Antikörper ist in präklinischen Modellen in seiner Pharmakokinetik und Wirkweise gut untersucht. Felzartamab induziert dabei eine Zytolyse CD38 hochexprimierender Zellen (in Tiermodellen Myelomzellen), über Antikörper-mediierte zelluläre Toxizität (ADCC) und Phagozytose, nicht aber (wie z.B. bei Daratumumab) Komplementaktivierung. In einer (first-in-human) multizentrischen Phase 1-2a Studie konnte eine hohe Effizienz und Sicherheit in der Therapie des relapsierenden oder refraktären Multiplen Myeloms gezeigt werden. Eine Besonderheit war dabei ein im Vergleich zu anderen klinisch eingesetzten CD38 Antikörpern ein geringeres Risiko für Infusions-assoziierte Reaktionen. Bzgl. anderer Adverse Events (AE) war das Profil anderen CD38 Antikörpern vergleichbar.

Felzartamab soll nun im Rahmen dieser Phase 2 Studie bei nierentransplantierten Patienten mit aktiver bzw. chronisch-aktiver ABMR eingehend untersucht werden.

Zielsetzung. Im Rahmen dieser (akademischen) prospektiven, randomisierten, doppelblinden, Placebo-kontrollierten, Pilotstudie (Phase 2) soll die Sicherheit, Verträglichkeit, Effektivität, Pharmakokinetik und Pharmakodynamik einer Therapie mit dem monoklonalen CD38 Antikörper Felzartamab bei Nierentransplantierten mit später aktiver bzw. chronisch-aktiver ABMR untersucht werden. Die Studie soll in erster Linie Aufschluss über die Sicherheit und Verträglichkeit dieser neuen Therapie bei immunsupprimierten Patienten geben, zudem aber (sekundäre Endpunkte) erste Ergebnisse zur Effektivität in der Therapie später aktiver ABMR ermöglichen (v.a. Abschätzung der Effektgröße und Variabilität als Basis für die Planung einer

nachfolgenden großen „proof of concept“ Studie). Dies beinhaltet in erster Linie eine Analyse des Effekts einer gezielten anti-CD38 Therapie auf Inflammation in der Mikrozirkulation, Abstoßungs-assoziierter Genexpressionsmuster, sowie Progression chronischer Schäden in frühen und späten Transplantat-Biopsien, auf Biomarker aktiver ABMR (DSA-Nachweis und -Verlauf, Serummarker für Inflammation und Endothelaktivierung/schaden, Verteilung von Leukozyten(sub)populationen), sowie Nierenfunktion, Proteinurie und prädiktiver Scores (als Surrogat für Langzeit-Transplantatüberleben).

Studiendesign. Die Studie wird an der Medizinischen Universität Wien, sowie an zwei weiteren Zentren (Charité Berlin; Grenoble Alpes University Hospital) durchgeführt (Inklusion von je 5-15 Patienten; insgesamt 22 Patienten). Dabei handelt es sich um eine prospektive, randomisierte doppelblinde Placebo-kontrollierte Pilotstudie der Phase 2. Es erfolgt eine 1:1 Randomisierung in zwei Gruppen, eine Felzartamab- und eine Placebo-Gruppe, wobei in 6 Zyklen insgesamt 9 Infusionen (Dosis: je 16 mg/kg) über einen Zeitraum von 24 Wochen verabreicht werden. Die Rekrutierungsphase soll 12-18 Monate betragen, daraus resultierend eine Studiendauer von 24-30 Monaten.

Primärer Endpunkt sind die Sicherheit und Verträglichkeit einer Therapie mit Felzartamab. Sekundäre Endpunkte sind die Pharmakokinetik (Konzentrationsverlauf von Felzartamab), Immunogenität (Nachweis von Antikörpern gegen Felzartamab) und Phamakodynamik (Plasmazell- und NK Zell-Reduktion im peripheren Blut) dieses Antikörpers, sowie eine präliminäre Analyse des Effekts auf den Abstoßungsprozess in zwei Protokollbiopsien, nach 24 und nach 52 Wochen: (i) Inflammation in der Mikrozirkulation, (ii) Progression chronischer Transplantatläsionen (Transplantatglomerulopathie, interstitielle Fibrose/tubuläre Atrophie), und (iii) molekulare Signaturen Antikörper-mediierter akuter und chronischer Transplantatschädigung. Des Weiteren wird der Effekt auf Abstoßungs-assoziierte Biomarker, wie DSA im Verlauf (parallel Serum-Immunglobulin-Spiegel und Impftiter), Zytokinmuster und Marker eines Endothelschadens sowie die Verteilung von Leukozyten-Subpopulationen und eine Genexpressionsanalyse auf RNA-Ebene in peripheren Blutzellen - komplementär zur Genexpressionsanalyse aus dem Nierenbiopsiematerial – durchgeführt. Der Grad der Immunsuppression wird über die Bestimmung der Torque Teno Virus (TTV)-Last und eine Analyse von Impftitern abgeschätzt werden. Klinische Endpunkte als Maß für Abstoßungsprogression sind

der Verlauf der eGFR (eGFR Slope), Proteinausscheidung (Protein/Kreatinin-Ratio), Transplantat- und Patientenüberleben, sowie das Auftreten akuter Abstoßungsepisoden. Mit erfolgter Inklusion von 10 bzw. 20 Patienten/Patientinnen (die zweite Interimanalyse erfolgt somit 1 Jahr vor Studienende) erfolgen Interimanalysen, deren Ergebnisse durch ein unabhängiges „Data and Safety Monitoring Board“ (DSMB) bewertet werden. Exakte (statistische) Kriterien für eine vorzeitige Studienbeendigung sind für diese Pilotstudie nicht definiert. Nach Bewertung der Sicherheit und Datenqualität in den zwei Interimanalysen wird die Studie jedoch vorzeitig beendet, wenn (i) in der Felzartamab-Gruppe schwere unerwünschte Ereignisse auftreten, die nach Einschätzung des DSMB auf ein inakzeptables Nutzen-Risiko-Profil hinweisen, und (ii) die Zahl der Drop-outs so hoch ist, dass ein adäquater Abschluss der Studie nicht gewährleistet werden kann.

Patienten. In diese Pilotstudie werden 22 erwachsene Patienten/Patientinnen inkludiert (unter Berücksichtigung einer antizipierten Dropout-Rate von 1-2 Fällen kann so eine Fallzahl von zumindest 20 Patienten erreicht werden). Wesentliche Einschlusskriterien sind die Diagnose einer späten aktiven oder chronisch/aktiven ABMR (C4d-positiv oder –negativ), zumindest 180 Tage nach Transplantation (Befund einer Indikationsbiopsie = Indexbiopsie, die im Rahmen der klinischen Routine aufgrund einer DSA Detektion und/oder einer langsamen Verschlechterung der Transplantatfunktion und/oder einer steigenden Proteinurie durchgeführt wurde), eine eGFR ≥20 ml/min/1.73 m^2 (Ausschluss weit fortgeschrittener irreversibler schwerer chronischer Schäden des Transplantats), der Nachweis von HLA Klasse I und/oder II-spezifischen DSA, sowie ein Alter >18 Jahre. Ausschlusskriterien sind eine aktive Teilnahme an einer anderen interventionellen Studie, Schwangerschaft und Stillperiode, ABO-inkompatible Transplantation, akute Abstoßung innerhalb eines Monats vor Inklusion, akute Verschlechterung der Transplantatfunktion mit Verdacht auf eine akute Abstoßung, Applikation eines anderen CD38 Antikörpers (z.B. Daratumumab) vor Studieninklusion, Applikation eines anderen immunmodulatorischen Antikörpers (z.B. Rituximab, anti-IL-6 oder anti-IL-6R Antikörper) ≤3 Monate vor Studieninklusion, erhöhte Leberwerte (Gesamt-Bilirubin >2×Upper Limit of Normal [ULN], Transaminasen >2·5×ULN), ein Hämoglobin <8 g/dL, Thrombozytenzahl <100 G/L, Leukozytenzahl <3 G/L, Neutrophilenzahl <1.5 G/L, eine Hypogammaglobulinämie mit einem Serum IgG <400 mg/dL, eine aktive virale,

bakterielle oder Pilz-Infektion, die den Einsatz einer intensivierten Immunsuppression verbietet, hier vor allem auch eine latente oder aktive Tuberkulose (positiver QuantiFERON-TB-Gold test), eine aktive maligne Erkrankung, eine schwere medizinische bzw. psychiatrische Erkrankung, die eine Teilnahme an einer klinischen Studie unmöglich macht, sowie eine Impfung mit einem Lebendimpfstoff innerhalb 6 Wochen vor Studieninklusion. Weitere Ausschlusskriterien sind der Nachweis einer T-Zell-mediierten Abstoßung (≥Grad I gemäß Banff Klassifikation), einer Polyomavirus-Nephropathie, einer *de novo* oder rekurrenten Glomerulonephritis, sowie einer *de novo* oder rekurrenten thrombotischen Mikroangiopathie in der Indexbiopsie.

Methodik. Die Intervention besteht in der seriellen intravenösen Verabreichung von Felzartamab in einer Dosis von 16 mg/kg in 250 mL 0.9% NaCl, im ersten Monat (erster Zyklus) in 1-wöchigem Abstand, dann in 4-wöchigem Abstand über eine Periode von 5 Monaten (Zyklus 2-6; insgesamt 6 Monate Therapiedauer). Patienten der Placebo-Gruppe erhalten Infusionen mit 250 mL 0.9% NaCl. Vor den ersten beiden Verabreichungen erfolgt eine intravenöse Vortherapie mit 100 mg Prednisolon, 30 mg Diphenhydramin und 1000 mg Paracetamol (in der Placebogruppe wird stattdessen Placebo verabreicht). Bei allen Patienten wird nach Zentrumsstandard die Basisimmunsuppression dem Befund einer ABMR in der Indexbiopsie angepasst (Adjustierung von Tacrolimus bzw. Cyclosporin A mit einem Ziel-Talspiegel von 5-10 ng/mL bzw. 80-120 ng/mL; bei Steroid-Freiheit Wiederbeginn mit Prednisolon, 5 mg/Tag; bei Dualtherapie ohne einen Proliferationsinhibitor, Start einer Therapie mit Mycophenolat Mofetil (oder, alternativ, „enteric-coated" Mycophenolsäure (EC-MPA)) mit einer Zieldosis von bis zu 2000 (bzw. 1440 mg)/Tag. Biomarker aus peripherem Blut: Die Detektion von DSA erfolgt unter Einsatz von Bead Array Technologie. Dabei werden HLA-Antigen-beladene Mikropartikel (Single Antigen Kits für die Spezifizierung der Spender-Spezifität nach Abgleichen mit dem Spender-HLA-Typ) mit Patienten- (bzw. Kontroll) Seren inkubiert, anschließend mit einem Fluoreszenz-markierten zweiten Antikörper die Bindung von IgG detektiert. Der Nachweis von Markern einer Endothel- und B-Zell-Aktivierung (Chemokine, BAFF) erfolgt unter Verwendung von Luminex-Assays. Der Nachweis von TTV erfolgt mittels quantitativer PCR. Leukozyten-Subpopulationen werden mittels Durchflusszytometrie erfasst. Für eine Genexpressionsanalyse auf RNA-Ebene in peripheren Blutzellen wird Vollblut in PAXgene Röhrchen aufgenommen, anschließend die RNA isoliert und in cDNA

umgeschrieben. Konzentrationsanalysen und anti-Felzartamab Antikörper: Die Serumkonzentration von Felzartamab und der Nachweis von möglichen Bildung von anti-Falzaratamab Antikörper wird mittels Ligandbindungsassays bestimmt. Transplantat-Biopsien: Biopsien (nach 12 und 52 Wochen) werden mittels Ultraschall-Kontrolle gemäß Zentrumsstandard durchgeführt (Verwendung einer 16 G Nadel; 1-2 Biopsiezylinder für eine Asservierung von Material für Lichtmikroskopie inkl. Immunhistochemie (C4d), Elektronenmikroskopie, und Genexpressionsanalysen. Die Diagnose und Kategorisierung einer ABMR erfolgt gemäß Banff 2019 Update auf Basis einer eingehenden morphologischen und molekularen Aufarbeitung. Diese umfasst ein Scoring von Glomerulitis, peritubulärer Kapillaritis, Transplantat-Glomerulopathie, interstitieller Fibrose und Tubulusatrophie (IFTA); eine immunhistochemische Aufarbeitung für den Nachweis kapillärer C4d-Ablagerung (fokal versus diffus; Scoring nach der Banff Klassifikation) sowie Elektronenmikroskopie für den Nachweis einer Lamellierung peritubulärer und glomerulärer Basalmembranen. Der Nachweis einer ABMR in der Genexpressionsanalyse (Kriterium der Banff Klassifikation; ein ABMR Score ≥0.2 ist Voraussetzung für eine Studieninklusion) erfolgt mittels cDNA Microarrays am Alberta Transplant Applied Genomics Centre, ATAGC, in Edmonton, Kanada („molecular microscope“, MMDx ™). Klinische Evaluation: Die Bestimmung der errechneten glomerulären Filtrationsrate (eGFR) erfolgt unter Verwendung der CKD-EPI eGFR, die Harnauscheidung von Protein wird mittels Protein/Kreatinin Ratio aus dem Spontanharn erfasst. Zudem wird nach 24 und nach 52 Wochen ein international validierter Prädiktions-Score (sog. iBOX Score unter Inklusion von Biopsie-assoziierten Parametern) berechnet, als Surrogat für eine Modifikation des Transplantat-Langzeitüberlebens. Statistische Analyse: Alle Analysen erfolgen nach dem Intention-to-Treat Prinzip. Kontinuierliche Daten werden mittels parametrischen oder nicht parametrischen Tests unabhängiger oder gepaarter Stichproben, kategorische mittels Fisher’s Exact Tests berechnet. Die Berechnung der eGFR Slope erfolgt mittels einem linearen gemischten Modell mit dem Verlauf der eGFR (Tag 0 bis Woche 52, alle 4 Wochen) als abhängiger Variable. Die Auswertung inkludiert zudem Analysen der Pharmakokinetik (Halbwertszeit, Tmax, Cmax, Clearance und Verteilungsvolumen), unter Einsatz von Standard-Software.

Belastungen, mögliche Nebenwirkungen:

Im Rahmen der Studie entstehen Mehrbelastungen durch Verabreichung von Felzartamab bzw. Plazebo (9 Verabreichungen in 6 Zyklen). Nebenwirkungen, die durch eine Therapie mit Felzartamab entstehen können (Daten aus einer Phase I-IIa Studie bei Multiplen Myelom) bestehen in erster Linie in Infusions-assoziierten Reaktionen zu Beginn der Therapie und hämatologischer Toxizität (Leukozytopenie, Anämie, Thrombozytopenie). Auch wenn in rezenten Phase 3 Studien für CD38 Antikörper keine substantielle Erhöhung des Infektionsrisikos im Vergleich zu Placebo oder anderen Myelom-Therapien gezeigt werden konnte, ist in Kombination mit immunsuppressiver Therapie bei Transplantierten von einem insgesamt erhöhten Infektionsrisiko auszugehen (entsprechend engmaschiges Infektions-Monitoring, wie in der Routine der Transplant-Nachbetreuung vorgesehen; aktive virale, bakterielle und Pilzinfektionen als Ausschlussgrund für einen Einschluss in die Studie. Eine weitere Belastung besteht, neben Routine-Laborkontrollen inkl. CNI-Spiegelbestimmung bei jeder Visite, in der Entnahme von zusätzlich 2 x 5 ml Blut bei der Mehrzahl aller Visiten (Pharmakokinetik und Immunogenität)) sowie zusätzlich 30 ml Blut am Tag 0, nach 12, 24 und nach 52 Wochen für die Gewinnung von Serum, Plasma und peripheren Blutzellen, zur Bestimmung von DSA, Leukozyten-(Sub)populationen, Chemokinen, TTV Virämie, sowie für Genexpressionsanalysen. Für Protokollbiopsien (zwei geplante Biopsien, nach 24 und nach 52 Wochen) gilt ein gut dokumentiertes geringes Komplikationsrisiko (selten: Hämatom, Hämaturie, außerordentlich selten Notwendigkeit einer chirurgischen Revision bis hin zu Nephrektomie, außerordentlich selten Blutdruckabfall bis hin zum Blutungsschock). Blutungsrisiken werden durch ein striktes Einhalten des Zentrums-Standards für Transplantat-Biopsie minimiert.

Risiken/Nutzenabwägung. Die chronische Abstoßung ist eine Hauptursache für einen Transplantatverlust im Langzeitverlauf. Bislang sind keine Therapien etabliert, die dem natürlichen Verlauf später Antikörper-vermittelter Abstoßung entgegenwirken. Der Organfunktionsverlauf für Patienten mit später ABMR ist ungünstig. Erste Ergebnisse der geplanten Studie haben das Potenzial, die Effizienz einer CD38 Antikörpertherapie, eines neuen therapeutischen Prinzips in der Therapie der chronischen Abstoßung, abzuschätzen. Sollten sich Hinweise ergeben, dass durch diese Therapie das Fortschreiten akuter und chronischer irreversibler Läsionen aufgehalten bzw. verzögert werden kann, dann hätte dies bedeutende Implikationen

für immunsuppressive Therapiestrategien in der Organtransplantation. In Hinblick auf die bedeutende Rolle humoraler Abstoßungsprozesse als wesentlicher Mediator chronischer Läsionen könnte der erfolgreiche Einsatz innovativer Therapien günstige Effekte auf das Langzeit-Transplantatüberleben haben. Demgegenüber ist eine Teilnahme an der Studie mit Belastungen und Risiken assoziiert (siehe oben). Für die Placebo-Gruppe im ersten Teil der Studie ergibt sich kein Nachteil, da diese, wie bisher nach Standard behandelt werden: dies beinhaltet eine Zugabe eines Steroids und/oder eines Proliferationshemmers (Mycophenolsäure), sofern nicht ohnehin Teil der Basistherapie, nicht aber eine spezifische anti-humorale Therapie, wie sie bei akuter Dysfunktion aufgrund einer frühen aktiven ABMR eingesetzt wird (z.B. Apherese). In Hinblick auf den möglichen Nutzen durch eine eventuelle Etablierung eines neuen immunsuppressiven Therapieansatzes für Transplantierte sowie den möglichen unmittelbaren Nutzen für die Patienten in der Interventionsgruppe, ist das Risiko durch Interventionen und Behandlungen im Rahmen der Studie vertretbar.

Optionen für eine Verlängerung der Studie

Option A: Es besteht für alle Studienteilnehmer nach Abschluss der randomisierten kontrollierten 1-jährigen Phase der Studie die Möglichkeit, an einer verlängerten Observationsphase bis 5 Jahre nach Studieninklusion teilzunehmen (Erfassung des Langzeitverlaufs von Transplantatfunktion und -überleben). Diese Extension umfasst jährliche Visiten ab Beendigung des randomisierten Teils der Studie.

Option B: Nach Abschluss der 1-jährigen randomisierten Placebo-kontrollierten Phase der Studie soll allen Patienten mit funktionierendem Transplantat und Fortbestehen einer aktiven Abstoßung die Option einer „open-label“ Felzartamab-Therapie angeboten werden. Voraussetzung für ein solches Vorgehen ist der im Rahmen der ersten Studienphase erbrachte Nachweis (i) eines Behandlungseffekts (Verringerung der Abstoßungsaktivität) in Relation zur Randomisierungssequenz und (ii) einer ausreichenden Verträglichkeit und Sicherheit einer Therapie mit Felzartamab. Die Ergebnisse werden dabei gemeinsam mit den Mitgliedern des DSMB bewertet. Das DSMB entscheidet dann nach Nutzen-Risiko Abwägung unabhängig vom Studienteam, ob den Patienten die Option einer „open label“ Felzartamab-Therapie angeboten werden kann. Die Therapie im Anschluss an die Behandlung soll in einem offenen Design, nach erneuter Einverständnis der Patienten und die Erfüllung aller definierten Ein-und Ausschlusskriterien vorausgesetzt, über weitere 6 Monate

erfolgen. Wesentliches Inklusionskriterium sind eine aktive oder chronisch aktive ABMR in der 12-Monatsbiopsie (für Patienten im Felzartamab-Arm vorausgesetzt, dass die 6-Monatsbiopsie ein Therapieansprechen angezeigt hat) oder in einer nach Abschluss der Studie durchgeführten Indikationsbiopsie. Für eine Fortführung der Therapie ist zudem eine eGFR >15 ml/min/1.73 m^2 obligat. Insgesamt erfolgen 9 Infusionen von Felzartamab erfolgen an Tag 0, sowie nach 1, 2, 3, 4, 8, 12, 16 und 20 Wochen, analog zur ersten Studienphase. Es erfolgen insgesamt zumindest 17 Visiten. Für ein Monitoring der Therapie erfolgen eine regelmäßige Erfassung von eGFR und Proteinurie (jede Visite) sowie (wie in der ersten Phase) ein Immunmonitoring mittels Bestimmung von DSA, dd-cfDNA, Chemokinen, TTV Virämie, Leukozyten-(Sub)populationen und Genexpressionsanalysen. Nach 24 und 52 Wochen erfolgt analog der ersten Studienphase eine Follow-up Biopsie (Histomorphologie und MMDX) zur Beurteilung der Wirksamkeit. Ebenso werden regelmäßig Harn und Blut für ein Immun-Monitoring gesammelt. Zur Analyse des Wirkmechanismus der Therapie soll nach zusätzlicher Einverständnis optional bei bis zu 8 Patienten im Rahmen zweier zusätzlicher Visiten eine Knochenmarks-aspiration zur Erforschung des Effekts von Felzartamab auf Antikörper-produzierende Zellen im Knochenmark erfolgen. Die mit Knochenmarksaspiration assoziierten Risiken beinhalten ein sehr geringes Risiko für Blutungen (sehr selten schwerwiegend mit Notwendigkeit einer Intervention), Infektionen im Bereich der Einstichstelle sowie Schmerzen während und nach dem Eingriff. Entsprechende Risiken werden durch ein striktes Einhalten des Zentrums-Standards minimiert.

<u>Individuelle Verlängerung der Felzartamab-Therapie während der zweiten 6-Monats-Phase von Option B:</u> Alle in Option B eingeschlossenen Patienten erhalten eine fixe 6-monatige Therapie mit Felzartamab. Aufgrund der beobachteten Rekurrenzrate der Antikörper-vermittelten Abstoßung nach Ende Therapie (Ergebnisse der ersten Studienphase) soll allen Patienten, die in Option B der Studienextension eingeschlossen wurden, die Möglichkeit einer individualisiert verlängerten Felzartamab-Therapie erhalten. Diese Verlängerung der Therapie erfolgt „on-demand“ in Abhängigkeit von der Dynamik eines bestimmten Biomarkers, der ein Fortbestehen bzw. ein Wiederauftreten des Astoßungsgeschehens nachweisen kann: die vom Spender in die Zirkulation (Plasma) freigesetzte zellfreie DNA („donor-derived cell-free DNA“; dd-cfDNA). Ergebnisse der ersten Studienphase haben einen signifikanten Abfall dieses Biomarkers nach Beginn der Felzartamab-Therapie und dabei eine gute

Assoziation mit der ABMR-Aktivität gezeigt. Nach Ende der Felzartamab-Therapie kam es – vor allem bei Patienten mit Rekurrenz der ABMR in der Histomorphologie – zu einem deutlichen Wiederanstieg des Biomarkers im peripheren Blut. Diesen Ergebnissen folgend ist eine individuelle „on-demand" Therapie geplant: Wenn der Biomarker stabil niedrig bleibt, erhalten die Patienten keine weitere Therapie während der 6-monatigen Beobachtungsphase. Wenn der Abstoßungs-Biomarker allerdings über gewisse vordefinierte Schwellenwerte ansteigt, werden nochmalige Felzartamab Infusionen verabreicht. Die Biomarker-Bestimmung erfolgt prospektiv in monatlichen Abständen. Das Ziel dieses maßgeschneiderten Vorgehens ist, dass nach 12 Monaten keiner der eingeschlossenen Patienten ein Wiederauftreten der Abstoßung in der 12-Monats-Biopsie zeigt.

Clinical study protocol

Version 7.0

Safety, Tolerability and Efficacy of Monoclonal CD38 Antibody Felzartamab in Late Antibody-Mediated Renal Allograft Rejection - A Phase 2 Pilot Trial

Trial Sponsor

Medical University of Vienna (MUW)
Spitalgasse 23, 1090 Vienna, Austria

Principal Investigator

Prof. Dr. Georg A. Böhmig
Medical University of Vienna
Department of Medicine III
Währinger Guertel 18-20
1090 Vienna, Austria
Phone: +43 1 40400 43630
Fax: + 43 1 40400 39302
Email: georg.boehmig@meduniwien.ac.at

Vienna, 06.08.2024

Table of Contents

1. LIST OF ABBREVIATIONS 19
2. BACKGROUND AND RATIONALE 20
2.1. CD38 antibody felzartamab (MOR202)....22
2.2. Pharmacokinetics and Immunogenicity of felzartamab in humans....23
2.3. Clinical efficacy and safety 24
3. HYPOTHESIS AND OBJECTIVE 24
4. STUDY DESIGN 25
4.1. Trial overview 25
4.2. Inclusion/exclusion criteria....26
4.3. Random allocation....27
4.4. Blinding and unblinding 28
5. INTERVENTIONS 28
5.1. Treatment with felzartamab/placebo....28
5.2. Premedication 29
5.3. The following medications are prohibited during the study:....30
5.4. The following concomitant medications are permitted during the study: 30
5.5. Baseline immunosuppression....30
6. OUTCOME MEASURES 30
7. METHODOLOGY 34
7.1. HLA antibody detection 34
7.2. Immunoglobulin levels 35
7.3. Pharmacokinetics and Immunogenicity 35
7.4. Transplant biopsies 35
7.5. Kidney function....36
7.6. Immunologic biomarkers 37
7.7. Leukocyte subpopulations 37
7.8. Gene expression analysis 38
7.9. Torque Teno virus (TTV) quantification 38
7.10. Course of vaccination titers....38
7.11. Collection of biological material (outside routine monitoring)....39
7.12. Responsible Laboratories that are involved into the study 39
8. SAMPLE SIZE AND STATISTICAL ANALYSIS....40
9. INTERIM ANALYSES....43
10. EXTENDED OBSERVATION AND TREATMENT OPTIONS....44
11. ADVERSE EVENTS 53

11.1. Felzartamab 53
11.2. Adverse drug reactions - specific considerations 55
11.2.1. Felzartamab 55
11.2.2. Premedication 59
11.3. Covid-19 61
11.4. Pregnancies and Women of Child Bearing Potential 61
12. SAFETY ASSESSMENT AND REPORTING 63
12.1. Safety Assessment 63
12.2. Definition of adverse events 65
12.2.1. Serious Adverse Events (SAEs) 66
12.2.2. SAEs related to study-mandated procedures 67
12.2.3. Suspected unexpected serious adverse reactions (SUSARs) 67
12.2.4. Pregnancy 67
12.2.5. Adverse events of special interest 68
12.3. Severity of adverse events 68
12.4. Relationship to study drug 69
12.5. Reporting procedures for any AEs 70
12.6. Reporting procedures for SAEs and AESIs 71
12.7. Reporting procedures for SUSARs 71
12.8. The Development Safety Update Report (DSUR) 72
13. STUDY TERMINATION, WITHDRAWAL, REPLACEMENT 72
13.1. Criteria for withdrawal 72
13.2. Follow-up of patients withdrawn from the study 74
13.3. Premature termination of the study 74
14. ETHICAL ISSUES 74
15. REGULATORY REQUIREMENTS 74
16. PERIODIC MONITORING 75
17. AUDIT AND INSPECTIONS 75
18. INVESTIGATOR TRAINING 76
19. RECORD RETENTION 76
20. STUDY REGISTRATION 76
21. MODIFICATIONS OF THE PROTOCOL 76
21.1. Protocol Amendments 76
21.2. Protocol Violations and Deviations 77
22. REFERENCES 78

1. LIST OF ABBREVIATIONS

ABMR = antibody-mediated rejection ADCC = antibody-dependent cellular cytotoxicity, ADCP = antibody-dependent cellular phagocytosis, AE = adverse event, AESI = adverse event of special interest, ALT = Alanin-Aminotransferase, AST = Aspartat-Aminotransferase, BM = basement membrane, CDC = complement-dependent cytotoxicity, cg = transplant glomerulopathy, CMV = cytomegalovirus, CNI = calcineurin inhibitor, CRF = Case-Report-Form, CyA = cyclosporine A, DSA = donor-specific antibody, DSMB = data and safety monitoring board, eGFR = estimated glomerular filtration rate, EOS = end-of-study, GCP = Good Clinical Practice, GLP = Good Laboratory Practice, HLA = human leukocyte antigen, HSV, herpes simplex virus, HZV, herpes zoster virus, IFTA = interstitial fibrosis/tubular atrophy, IgG = Immunoglobulin G, IL-6 = Interleukin-6, IL-6R = Interleukin 6 receptor, IV = intravenous, IVIG = intravenous immunoglobulin, KTX = kidney transplantation, MFI = mean fluorescence intensity, MLPTC = multilayering of peritubular capillary basement membranes, MM = multiple myeloma, MMDx = molecular microscope; MMF = mycophenolate mofetil, MPA = mycophenolic acid, mTOR = mammalian target of rapamycin, MUW = Medical University Vienna, PC = plasma cell, PD = pharmacodynamics, PK = pharmacokinetics, PTC = peritubular capillaries, SAE = serious adverse event, SAFB = single-antigen flow-beads, SD = standard deviation, TTV = Torque Teno virus, ULN = upper limit of normal

2. BACKGROUND AND RATIONALE

Antibody-mediated rejection (ABMR) is one of the leading causes of kidney allograft dysfunction and failure [1]. This type of rejection, commonly triggered by *de novo* anti-HLA donor-specific antibodies (DSA), is a prevalent finding in late indication biopsies (up to 30-40% of indication biopsies in some kidney transplant cohorts). Its diagnosis, which is based on a combination of serological, morphologic and molecular criteria, is associated with a progressive decline in glomerular filtration rate (GFR), increased proteinuria, and graft failure [1]. While a continuous diagnostic refinement has helped define the role of this rejection type as a major trigger of chronic allograft injury, treatment of late and/or chronic ABMR still represents a major challenge. Currently, there is no treatment that has been proven to modify its course. [2-4]. Our knowledge is mainly based on the results of uncontrolled studies, where a variety of different treatment strategies were evaluated, ranging from modifications in maintenance immunosuppression [5-7] to different immunomodulatory measures, including high dose intravenous immunoglobulin (IVIG) with or without CD20 antibody rituximab [8-11], the proteasome inhibitor bortezomib [12], or complement inhibitors [13]. Recent randomized controlled trials have failed to demonstrate efficiency of proteasome inhibition (BORTEJECT trial) to affect the integrity of alloantibody-producing plasma cells (PC) [14], combined use of CD20 antibody rituximab and intravenous immunoglobulin (TRITON trial) for modulation of B cell immunity [15], and anti-C5 antibody eculizumab [16]. One interesting concept may be the interference with the IL-6/IL-6R axis to modulate the activation and development of B cells and antibody production [17]. In a very recent randomized controlled phase 2 pilot trial (Vienna, Berlin) we found a modulation of DSA levels, and under prolonged treatment a modulation of molecular rejection scores in transplant biopsies, associated with a significant effect on eGFR decline [18]. However, this trial revealed important safety signals, in particular the occurrence of infection-related serious adverse events (AE) and two cases of diverticulitis, suggesting the need of a careful evaluation in larger longer-duration phase 3 trial (which is currently underway; IMAGINE; clinicaltrials.gov identifier: NCT03744910) [18].

An increased understanding of the pathophysiology of ABMR may be a clue for the design of innovative therapeutic strategies [3]. Donor-specific antibodies produced by long-lived PC bind to the endothelial surface within the microvasculature of the graft, where they trigger tissue inflammation and injury, via complement-dependent and

complement-independent mechanisms. Recent studies have suggested a dominant role of natural killer (NK), e.g. activated upon engagement of activating Fc gamma receptor IIIA with endothelium-bound IgG [19,20].

One promising immunotherapeutic target may be CD38, a 43.7 kDa type II transmembrane protein primarily expressed on immune and hematopoietic cells, with particularly high expression levels on antibody-producing PC and natural killer (NK) cells [21]. CD38 is known to exhibit ecto-enzymatic activity as nicotinamide adenine dinucleotide (NAD+)-glycohydrolase / ADP-ribosyl cyclase. *In vitro* data suggest a potential role as an adhesion receptor supporting the interaction of lymphocytes with endothelial cells by interaction with CD31 (PECAM-1) and as a cell-activating receptor that upon ligation triggers among others proliferation and cytokine production [21]. Monoclonal antibodies against this surface protein are well established to be highly effective in the treatment of multiple myeloma (MM) [22]. Mechanisms of action include complement-dependent cytotoxicity (CDC), antibody-dependent cellular cytotoxicity (ADCC)/phagocytosis (ADCP), and/or apoptotic signaling [23-25]. Considering the critical role of alloantibody-producing PC in ABMR, efficacy of this therapeutic principle can also be expected for transplant conditions where DSA are a cause of injury. Currently, however, there is only scarce experience with daratumumab in this context.

We speculate that CD38 antibody treatment may effectively deplete also DSA-producing PC and thus, by reducing the load of deleterious alloreactivity, halt the progression of rejection. Beyond an effect on DSA production, targeting CD38 may interfere also with a key pathomechanism in ABMR, that is, antibody-dependent NK cell activation.

The concept of targeting CD38 to counteract ABMR is supported by a recently published case of ABMR (associated with smoldering MM; Medical University of Vienna), where a 9-month course of CD38 antibody daratumumab has led to a complete reversal of severe chronic active ABMR, with complete morphological and molecular resolution (two follow-up biopsies) of rejection activity [26]. Treatment led to a marked reduction of DSA (associated with a depletion and modulation of bone marrow-derived alloantibody-producing PC) and, in parallel, a profound reduction in peripheral blood NK cell counts and intra-graft NK cell infiltrates. This intriguing case may provide a valuable basis for the design of a systematic trial to investigate the concept of targeting CD38 in late ABMR [26].

Support for efficacy of CD38 antibody treatment also comes from two additional very recent observations. Jordan et al. (https://atcmeetingabstracts.com/abstract/daratumumab-for-treatment-of-antibody-mediated-rejection-in-a-kidney-transplant-recipient/) reported on a case of refractory ABMR, which was completely reversed by a treatment course with daratumumab. Moreover, in a recent experimental study (sensitized rhesus macaques, transplant model), targeting CD38 was shown to significantly reduce donor-specific antibodies and to prolong renal allograft survival [27]. In the same study, the authors reported on a significant decrease in DSA in two clinical cases (combined heart and kidney allograft recipient with refractory ABMR and a highly sensitized heart transplant candidate), leading to clinical improvement of rejection and accelerated heart graft access [27].

On the basis of the above detailed preliminary data, we hypothesize that targeting CD38, and thus interference with the integrity and function of NK cells and DSA-producing PC, is able to counteract tissue inflammation and injury in ongoing ABMR, in particular, inflammation in the microcirculation and, as a consequence, alloantibody-triggered chronic graft injury.

2.1. CD38 antibody felzartamab (MOR202)

Felzartamab (MOR202; also referred to as MOR03087); Human Immunology Biosciences, Inc. [HI-Bio], South San Francisco, CA, USA) is a recombinant fully human monoclonal CD38 antibody (IgGλ) derived from a proprietary antibody phage library (produced in human PER.C6® cells by recombinant expression technology). This antibody was initially developed for the treatment of MM, but is currently also being evaluated in autoimmune disease (membranous nephropathy). Details on felzartamab are provided in the attached Investigator Brochure. In vitro binding studies have shown a binding affinity for CD38 in the low nanomolar range. The main mode of action for MOR202-induced lysis of MM cells is ADCC and ADCP, but not CDC, which is believed to be a major contributor to infusion-related reactions. In the last decade, felzartamab has been evaluated thoroughly in preclinical (e.g. xenograft orthotopic MM mouse model) and in a recent clinical trial (phase I-IIa trial in MM patients) [28]. Felzartamab has not yet been approved by the FDA or EMA for any condition. For patients diagnosed with refractory and relapsed MM, this antibody showed an efficacy and safety profile comparable to other CD38 antibodies, such as daratumumab and isatuximab [28]. A major advantage may be a, compared to other CD38 antibodies, lower risk of infusion-

related reactions [28]. The felzartamab development program includes several non-clinical [29-31] and clinical studies conducted in subjects with MM (first-in-human phase I-II; ClinicalTrials.gov Identifier: NCT01421186 [28]) and, outside the hematology field, in antiPLA2R-positive membranous nephropathy (trial ongoing; phase Ib/IIa; ClinicalTrials.gov Identifier: NCT04145440). To date, no studies with felzartamab have been conducted in organ transplant recipients.

2.2. Pharmacokinetics and Immunogenicity of felzartamab in humans

(Experimental data on pharmacokinetics are provided in the Investigator Brochure). Clinical data on pharmacokinetics of felzartamab were evaluated in patients with relapsing or refractory MM 28. In this study, the authors observed an inverse correlation of drug clearance with serum albumin concentrations (indirect measure of neonatal Fc receptor (FcRn) activity), resulting in a moderate effect on felzartamab concentrations. Moreover, the volume of distribution was inversely correlated with the dose of the antibody. This finding was explained by target-mediated drug disposition effects, which were most prominent at lower doses. Combined MM treatments with pomalidomide or lenalidomide had no effect on the pharmacokinetics of MOR20, and felzartamab did not influence the pharmacokinetics of these drugs. Final population pharmacokinetic parameter estimates are shown in Table 1.

Table 1. Pharmacokinetic parameter estimates (phase 1-2a trial in MM patients)

Parameters	Estimates	%rel. standard error
CL (L/day)	0·413	8·728
V1 (L)	3·92	2·413
Q (L/day)	1·265	5·61
V2 (L)	2·197	10·84
Vmax (mg/day)	29·6	10·81
Km (mg/L)	1·437 FIX	-
KD (/day)	0·005988	28·88
Albumin effect on CL and Vmax	-1·802	9·513
Dose effect on V1	-0·2118	8·25
CL (L/day)	0·413	8·728

CL=clearance; IIV=inter-individual variability; KD=rate constant for decline of Vmax over time; Km=drug concentration at which the reaction rate is half the Vmax; Q=inter-

compartmental clearance; V1=central volume of distribution; V2=peripheral volume of distribution; Vmax=maximum reaction rate.

Immunogenicity in humans. So far, anti-drug antibody samples from 85 MM subjects continuously treated with felzartamab for up to 29.5 month were analyzed with no ADAs detected. These data suggest that felzartamab offers only a very low risk for the induction of anti-drug antibodies (ADA).

2.3. Clinical efficacy and safety

Details on the safety profile: see section 9. Efficacy and safety data for felzartamab as monotherapy or in combination with other treatments are available from a first-in-human phase I-IIa trial conducted in refractory or relapsing MM [28]. Trial results indicate that felzartamab can be safely administered in combination with other treatments, such as pomalidomide and dexamethasone. An important finding was a low rate of infusion-related reactions, which was by far lower than that observed for other CD38 antibodies (daratumumab, isatuximab). For MM treatment, trial results suggested a dose of 16 mg/kg and a regimen of four 1-weekly (first 28-day cycle) followed by 2-weekly infusions in subsequent cycles.

3. HYPOTHESIS AND OBJECTIVE

Clinical data to date support that targeting CD38 is safe and well tolerated, and we hypothesize that felzartamab will have an acceptable safety profile in kidney transplant recipients on baseline immunosuppression. Moreover, we hypothesize that repeated administration of felzartamab is able to counteract tissue inflammation and injury in ongoing ABMR, in particular, inflammation in the microcirculation, HLA antigen-specific B cell alloresponses and, as a consequence, alloantibody/NK cell-triggered chronic graft injury.

The objective of our planned randomized controlled pilot trial (inclusion of 2 transplant centers) will be to evaluate the safety, tolerability and efficacy of CD38 monoclonal antibody felzartamab in a prospective cohort of 22 kidney transplant recipients diagnosed with late active or chronic active ABMR. Our planned trial will

evaluate preliminary efficacy of targeting CD38 in late active ABMR, and can be expected to provide a valuable basis for the potential design of a pivotal trial powered for the detection of clinical outcome differences.

4. STUDY DESIGN

This planned prospective bi-center study (University of Vienna, Charité Universitätsmedizin Berlin, Sponsor: Medical University of Vienna, Vienna, Austria) is an investigator-driven pilot trial designed to assess the safety, tolerability, pharmacokinetics, immunogenicity, pharmacodynamics and efficacy (preliminary assessment) of the fully human CD38 monoclonal antibody felzartamab in kidney transplant recipients with late active or chronic-active ABMR. The sponsor of this noncommercial trial is the Medical University of Vienna. Apart from study design, the sponsor will carry out the trial (in collaboration with research partners), being responsible for all its scientific, ethical, regulatory and legal aspects. The funder (HI-Bio, South San Francisco, CA, USA) has set funding conditions and will provide external funding. The trial will be designed as a randomized, controlled, double-blind phase 2 pilot trial. The primary endpoint will be safety and tolerability.

4.1. Trial overview

A flowchart of the trial is shown in Figure 1.

Figure 1

Participants will be randomized to receive either felzartamab (16 mg/kg, intravenous [IV] administration) or placebo (1:1 randomization) for a period of 6 months (administration of felzartamab/placebo at day 0, 7, 14, 21 (cycle 1), and thereafter in 4-weekly intervals at weeks 4, 8, 12, 16, and 20 (cycles 2-6). After six (week 24) and twelve months (week 52), study participants will be subjected to follow-up allograft biopsies. Primary goals of the trial are to assess the safety, pharmacokinetics and pharmacodynamics (peripheral blood PC and NK cell depletion) of a 6-month course of treatment over a period of 12 months. The trial will in addition provide first data on efficacy (progression/activity of rejection, blood biomarkers) and potential associations

of treatment with parameters reflecting clinical progression of allograft dysfunction (course of renal function [32] or iBOX score [33]). We expect completion of patient recruitment after 12-18 months and a total study duration of 24-30 months (12 months follow-up period).

4.2. Inclusion/exclusion criteria

Inclusion and exclusion criteria are listed in Table 2. We plan to include 22 kidney transplant recipients (considering an anticipated drop-out rate of 1-2 patients, this planned case number is expected to allow for at least 20 participants completing the trial). Inclusion criteria circulating anti-HLA DSA and biopsy features of late (≥180 days post-transplant) active ABMR (ABMR according to the Banff 2019 scheme) in an indication biopsy (index biopsy; performed within the clinical routine for a positive post-transplant DSA result and slow deterioration of allograft function and/or proteinuria). Other key inclusion criteria are an age >18 years, a functioning graft at ≥180 days post-transplantation and an estimated GFR (eGFR according to the CKD-EPI equation) ≥20 ml/min/1.73 m^2. Exclusion criteria are detailed in Table 2.

Table 2. Inclusion and exclusion criteria.

**INCLUSION CRITERIA**
1. Voluntary written informed consent
2. Age >18 years (maximum: 80 years)
3. Functioning living or deceased donor allograft after ≥180 days post-transplantation eGFR ≥20 ml/min/1.73 m^2 (CKD-EPI formula)
4. HLA class I and/or II antigen-specific antibodies (preformed and/or *de novo* DSA).
5. Active or chronic/active ABMR (±C4d in PTC) according to the Banff 2019 classification Molecular ABMR score (MMDx) ≥0.2
**EXCLUSION CRITERIA**
1. Patients actively participating in another clinical trial
2. Age ≤18 years
3. Female subject is pregnant or lactating or not on adequate contraceptive therapy
4. ABO-incompatible transplant
5. Index biopsy results:<br>T-cell-mediated rejection classified Banff grade ≥I<br>*De novo* or recurrent severe thrombotic microangiopathy<br>Polyoma virus nephropathy<br>*De novo* or recurrent glomerulonephritis
6. Acute rejection treatment ≤3 month before screening
7. Previous treatment with other CD38 monoclonal antibodies (e.g. daratumumab)
8. Previous treatment with other immunomodulatory monoclonal/polyclonal antibodies (e.g.
9. CD20 Ab rituximab, IL-6/IL-6R Ab) ≤3 months before study treatment
10. Total bilirubin >2×the upper limit of normal [ULN], alanine transaminase and aspartate aminotransferase >2·5×ULN
11. Haemoglobin <8 g/dL
12. Thrombocytopenia: Platelets <100 G/L
13. Leukopenia: Leukocytes <3 G/L
14. Neutropenia: Neutrophils < 1.5 G/L
15. Hypogammaglobulinemia: Serum IgG <400 mg/dL
16. Active viral, bacterial or fungal infection precluding intensified immunosuppression
17. Active malignant disease precluding intensified immunosuppressive therapy
18. Latent or active tuberculosis (positive QuantiFERON-TB-Gold test)
19. Administration of a live vaccine within 6 weeks of screening
20. History of alcohol or illicit substance abuse
21. Serious medical or psychiatric illness likely to interfere with participation in the study

4.3. Random allocation

Subjects eligible for study inclusion will be randomized in a 1:1 ratio to one of the two study arms (felzartamab versus placebo) by computer assignment using a web-based randomization platform (www.meduniwien.ac.at/randomizer). Patients will be randomized after completion of the screening phase and after informed consent has been obtained, at the day of study initiation. Randomization will be stratified by study site and according to ABMR categories (active ABMR versus chronic/active ABMR) to ensure a balance of patients with these two histological types between the two arms. For each patient, a study ID will be assigned. The trial will be carried out in a double-

blind fashion. Individual roles of investigators, study nurses and clinical pharmacists will be defined within the online tool: study participants, care providers, and those assessing outcomes will be unaware of the randomization sequence, being blinded to group allocation until the completion of the study. The allocation sequence will be generated and medication/placebo will be prepared by independent study pharmacists (Vienna: Department of Clinical Pharmacology). Study physicians and nurses will be provided with blinded intravenous and oral medication. For included patients, treatment code envelopes will be securely stored on site, and in case of emergency, the respective code envelope can be opened.

4.4. Blinding and unblinding

The study is designed as a double-blinded trial, in order to minimize bias. Investigational drug (felzartamab) and placebo (normal 0.9% saline) are prepared by non-blinded pharmacists and formulated to have identical appearance. Neither study participants nor medical staff interacting with patients or data know the assigned treatment (felzartamab versus placebo). The randomization sequence will be unblinded by a dedicated clinical pharmacist (according to his/her role defined in the randomization tool) after the last patient has completed the trial. Premature unblinding (treatment code envelopes) may be necessary in cases of medical emergencies or serious medical conditions, where participants cannot be treated adequately unless the medical staff knows the allocated treatment, or reports of suspected unexpected serious adverse events. Unblinding can, if necessary, be requested by the data and safety monitoring board (DSMB).

5. INTERVENTIONS

5.1. Treatment with felzartamab/placebo

Based on the results of a PK modelling for an ongoing phase Ib/IIa trial in autoimmune disease (membranous nephropathy; ClinicalTrials.gov Identifier: NCT04145440; see investigator brochure), patients will be dosed with felzartamab for a period of 6 months, administered as an intravenous infusion. As transplant patients are on multi-compound immunosuppressive baseline therapy, and therefore at increased risk of infections, we plan a reduction in doing intervals after the first cycle. 9 doses of Felzartamab will be administered as an intravenous infusion at 16 mg/kg over 6 treatment cycles at 28-

days each. Dosing occurs every week in cycle 1 (C1) and every four weeks in cycle 2-6. The first two infusions of felzartamab will be slow (approximately 90 min), and, if no infusion reactions occur, infusion times may be shortened to 1 hour or shorter (minimum 30 min) in subsequent infusions. In case of infusion reactions, the infusion is stopped temporarily or entirely depending on the severity. In some cases, premedication may be extended to subsequent infusions. The infusion rate for resuming felzartamab application is slower.

Felzartamab (MOR202; MOR03087) will be supplied and provided by Hi-Bio [One vial contains 325 mg felzartamab (lyophilizate); nominal concentration/composition after reconstitution with 4.8 mL water for injection: 65 mg/mL felzartamab in 10 mM L-histidine, 260 mM sucrose, 0.1% polysorbate 20, pH 6.0.]. The reconstituted solution (65 mg/mL) should be used immediately for further dilution (storage prior to dilution for up to 8 hours at 2°C to 25°C). Felzartamab will be administered after dilution with 250 mL 0.9% sodium chloride solution (final concentration should be between 1 and 20 mg/mL). Prepared infusions may be stored at room temperature for up to 16 hours (concentration 1-10 mg/mL) or 8 hours (10-20 mg/mL), respectively (this includes also the duration of infusion). The prepared infusions will be protected from light. Prior to administration, felzartamab/placebo infusion must reach room temperature by storing unrefrigerated for 30 to 60 minutes before use. The placebo medication will be administered with 250 mL normal saline for infusion (commercially available in country) and will be provided by the Investigator.

Storage and Accountability. Felzartamab (lyophilisate) will be stored at 2°C to 8°C, with protection from light. Placebo will be stored at controlled room temperature and should not be frozen. Study product disposition and accountability will be documented on the subject level, whereby all study product dispositions will be listed (drug accountability log). In a stock record, overall bulk study product supplies and accountability will be recorded.

5.2. Premedication

To prevent infusion-related reactions, patients allocated to the felzartamab arm will receive intravenous premedication prior to the first two felzartamab infusions (day 0 and day 14). Patients in the placebo arm will receive placebo (0.9% NaCl solution).

Premedication will be administered 30 min before the infusion of felzartamab, and will consist of Diphenhydramine (30 mg), Paracetamol (1000 mg), and Prednisolone (100 mg), respectively (each in 100 mL Volume). In the placebo arm, patients will receive 3x100 mL NaCl 0.9%.

5.3. The following medications are prohibited during the study:

Rituximab, eculizumab, proteasome inhibitors, IVIG, plasma exchange or immunoadsorption, other investigational drugs/treatments including commercially available CD38 or anti-IL-6/sIL-6R monoclonal antibody drugs such as daratumumab (Darzalex®) or tocilizumab (RoActemra®).

5.4. The following concomitant medications are permitted during the study:

Calcineurin inhibitors (CNI, tacrolimus or cyclosporine A), mammalian target of rapamycin (mTOR) inhibitor (everolimus or rapamycin), mycophenolate mofetil (MMF)/mycophenolate sodium; long-term treatment with low dose corticosteroids (prednisolone 5mg/day).

5.5. Baseline immunosuppression

Upon diagnosis of late ABMR, all recipients on therapy with a calcineurin inhibitor [tacrolimus or cyclosporine A (CyA)] or a mTOR inhibitor (everolimus or rapamycin), without azathioprine or mycophenolic acid (MPA), will receive mycophenolate mofetil (or, alternatively, enteric-coated mycophenolic acid (EC-MPA), initially at a dose of 2 x 500 mg (or 2 x 360 mg, respectively) per day; stepwise increase to 2 x 1000 mg (or 2 x 720 mg) per day if tolerated) to avoid under-immunosuppression. Tacrolimus will be adjusted to achieve target trough levels between 5 and 10 ng/mL, CyA to 80-120 ng/mL. Recipients weaned off steroids will receive low dose prednisolone (5 mg/day).

6. OUTCOME MEASURES

Study endpoints are listed in Table 3.

Table 3

**PRIMARY OUTCOME**
**Safety and tolerability** of felzartamab in renal allograft recipients with ABMR on baseline immunosuppression
**SECONDARY OUTCOMES**
**DSA/immunoglobulin levels (week 0, 12, 24, and 52)**<br>Mean fluorescence intensity (MFI) of immunodominant DSA<br>Changes in levels of the immunodominant DSA calculated from dilution experiments<br>Number of detected DSA<br>Total Ig (IgG, IgA, IgM) and IgG subclasses (IgG1, IgG2, IgG3, IgG4)<br>Course of vaccination titers<br>**Effect on leukocyte subsets in peripheral blood (week 0, 1, 4, 8, 12, 24, and 52)**<br>Circulating PC, NK cells, T and B cell subpopulations, expression of CD38 using non-crossreactive CD38 Ab clone HIT2<br>**Results of follow-up protocol biopsies (week 24 and 52)**<br>***Morphological results***:<br>ABMR category (active vs. chronic active ABMR; C4d+ vs. C4d- ABMR)<br>Extent of glomerular/pertibulular capillary microcirculation inflammation (g+ptc score)<br>Transplant glomerulopathy (cg) and interstitial fibrosis/tubular atrophy scores<br>Intragraft complement activation<br>Patterns of intragraft cellular infiltrates (NK cells, PC, T cells, B cells)<br>***Gene expression analysis*** (Molecular Microscope® Diagnostic System, MMDx)<br>Molecular classifiers/scores related to ABMR and T cell-mediated rejection<br>Molecular classifiers/scores related to rejection in general<br>Molecular classifiers/scores related to acute and chronic renal injury<br>Archetypal analysis of rejection-related categories<br>Pathogenesis-based transcripts (PBT) scores (cytotoxic T cell infiltration, γ-interferon effects, NK cell burden, epithelial cell damage)<br>**Effect on immunologic biomarkers (week 0, 12, 24, and 52**)<br>CXCL9 and CXCL10 levels in blood and urine (Luminex-based detection)<br>BAFF levels in blood (ELISA/Luminex)<br>**Effect on measures of overall immunosuppression (week 0, 12, 24, and 52)**<br>Torque Teno virus (TTV) levels in plasma (quantitative PCR)<br>**Effect on clinical outcome parameters and surrogate endpoints:**<br>eGFR slope over a period of 52 weeks (4-weekly measurements)<br>iBox clinical prediction score at, 24 and 52 weeks<br>Protein excretion (protein/creatinine ratio) over 52 weeks (4-weekly measurements)<br>12-month (death-censored and overall) graft and patient survival

Schedule of events is provided in Table 4.

Table 4 Schedule of Events

**Visit**	**Screen**	**1**	**2**	**3**	**4**	**5**	**6**	**7**	**8**	**9**	**10**	**11**	**12**	**13**	**14**	**15**	**16**	**17**
**Week**	**up to -4**	**0**	**1**	**2**	**3**	**4**	**8**	**12**	**16**	**20**	**24**	**28**	**32**	**36**	**40**	**44**	**48**	**52**
Informed consent	x																	
Physical examination, medical history	x	x									x							x
Concomitant medications	x	x	x	x	x	x	x	x	x	x	x	x	x	x	x	x	x	x
Pregnancy test, if applicable	x					x	x	x	x	x	x	x	x	x	x	x	x	x
Quantiferon test	x																	
Virology	x																	
ECG	x										x							x
**Felzartamab/placebo**		x	x	x	x	x	x	x	x	x								
**Safety**																		
Temperature/heart rate/blood pressure		x	x	x	x	x	x	x	x	x	x	x	x	x	x	x	x	x
AE monitoring		x	x	x	x	x	x	x	x	x	x	x	x	x	x	x	x	x
Safety Lab		x	x	x	x	x	x	x	x	x	x	x	x	x	x	x	x	x
Cov-19 Test/nasal swab		x	x	x	x	x	x	x	x	x	x	x	x	x	x	x	x	x
Ig(sub)classes		x				x	x	x	x	x	x		x		x			x
CMV/HSV/HZV PCR		x				x	x	x	x	x	x		x		x			x
Vaccination titers		x						x			x							x
TTV copy number		x						x			x							x
**PK and Immunogenicity**																		
Felzartamab serum concentration		x[2]	x[2]	x[2]	x[1]	x[1]	x[1]	x[1]	x[1]	x[1]	x	x	x	x	x	x	x	x
Anti-felzartamab antibodies		x[1]	x			x[1]		x[1]			x			x				x
**Preliminary efficacy assessment**																		
Follow-up biopsies (incl. MMDx)											x							x
DSA detection		x						x			x							x
Chemokines		x						x			x							x
BAFF levels		x	x	x		x		x			x							x
Gene expression analysis		x						x			x							x
Cell-free donor-derived DNA		x						x			x							x
Leukocyte subpopulations		x	x	x		x		x			x							x
eGFR (CKD-EPI equation)		x	x	x	x	x	x	x	x	x	x	x	x	x	x	x	x	x
Protein/creatinine ratio		x	x	x	x	x	x	x	x	x	x	x	x	x	x	x	x	x
iBOX score		x						x			x							x
**Immunosuppression**																		
CNI/mTOR inhibitor trough level		x	x	x	x	x	x	x	x	x	x	x	x	x	x	x	x	x
**Biobanking** (Plasma, plasma in Streck tubes, serum, blood RNA, urine)		x						x			x							x

Legend:

Procedure	Details
**Pregnancy Test**	▪ ß- humane Choriongonadotropin, Urine-test
**Quantiferon Test**	▪ QuantiFERON-TB-Gold test, Quiagen
**Virology**	▪ HIV Ab 1+2, HbsAg, HbsAb, HbcAb, HCVAb
**CMV, HSV, HZV PCR**	▪ Real-time PCR for quantification of viral load
**Vaccination titers**	▪ HBs Ab, mumps, measles and rubella Ab; Covid-19 titers
**Torque Teno virus (TTV) quantification**	▪ TTV PCR, Plasma samples
**Covid-19**	▪ Antigen test, if positive, confirmation by PCR assay
**Safety lab**	haemoglobin, erythrocyte count, MCV, white blood cell count and differential (neutrophils, bands, lymphocytes, monocytes, eosinophils and basophils), platelets, PT, PTT, sodium, potassium, calcium, phosphate, magnesium, urea, creatinine, total protein, C-reactive protein, creatinine kinase, venous blood gas analysis, albumin, ALT (SGPT), AST (SGOT), alkaline phosphatise, γGT, LDH, total bilirubin, LDL, HDL, cholesterol, triglycerides, uric acid, glucose, total amylase, TSH
**PK and Immunogenicity**	x^1: pre-dose only; x^2: pre-dose and 30 min after end of infusion
**Gene Expression**	▪ PAXgene Blood RNA tubes
**Chemokines, BAFF**	▪ CXCL9, CXCL10 and BAFF using multiplex beads on a Luminex 200 reader, or ELISA technique
**Cell-free donor-derived DNA**	▪ Assessment of the proportion of cf-dd DNA in plasma samples by next-generation sequencing
**Leukocyte subpopulations**	▪ DuraClone®; flow cytometry based immune phenotyping
**iBox risk prediction score**	▪ Integrative system to predict long term kidney allograft failure

Analysis of safety and tolerability will be the primary endpoint. Major secondary endpoints include the course of DSA (and in parallel total Ig and IgG subclass levels), the dynamics of peripheral blood counts of PC, NK cells, and T and B cell subpopulations (Duraclone multicolor flow cytometry; panels: phenotyping/basic, T cell subsets, Treg panel, B cell panel, PC panel), as well as biomarkers of rejection (Chemokine (C-X-C motif) ligand (CXCL)9 and CXCL10 in blood and urine), B cell immunity (BAFF) and overall immunosuppression (Torque Teno viral load [34]). Moreover, 6- and 12-month renal allograft biopsies will be assessed for morphological (Banff criteria of rejection and chronic injury; immunohistochemistry for detection of complement activation/deposition and characterization of cellular infiltrates including NK cells) and molecular rejection criteria (molecular ABMR score; microarray analysis using the Molecular Microscope® Diagnostic System, MMDx [35]), including pathogenesis-based transcripts (PBT) scores (cytotoxic T cell infiltration, γ-interferon effects, natural killer cell burden, epithelial cell damage). Clinical endpoints will be proteinuria as well as the slope of eGFR and the iBox clinical prediction score, both validated surrogate endpoints that accurately predict long-term allograft survival [32,33].

7. METHODOLOGY

7.1. HLA antibody detection

For assessment of the course of HLA antibody levels, serum samples will be evaluated after completion of the study according to published protocols [18]. For antibody detection, LABscreen single-antigen flow-bead assays (One Lambda, Canoga Park, CA) will be applied. Serum samples will be incubated with ethylenediaminetetraacetic acid (EDTA, 10 mM) to prevent complement interference [36]. Data acquisition will be performed via a LABScan™ 200 flow analyzer (Luminex Corporation, Austin, TX, USA). For longitudinal analysis of DSA/HLA antibody levels, bead assays will be performed retrospectively (centralized analysis) to avoid influences of day-by-day variations in test results (test batches including samples from 4-6 patients each). Donor-specificity will be defined according to serological and/or low- or high-resolution donor/recipient HLA typing (HLA-A, -B, -Cw, -DR, -DQ, -DP upon availability) provided either by the local HLA lab or the Eurotransplant database. Test results will be documented as mean fluorescence intensity (MFI) of the immunodominant DSA. An

MFI threshold >1,000 will be considered as positive. To estimate the impact of felzartamab treatment on DSA levels, we will document the percent change in MFI. In an effort to quantify changes in DSA levels more accurately, we plan to additionally perform dilution experiments following an earlier described protocol [26]. In brief, nonlinear standard curves based on raw DSA MFI levels (immunodominant DSA) will be obtained by serial dilution of individual patient sera collected prior to start of treatment (all samples were incubated with EDTA) and at week 24. According to computed standard curves, the fold change of antibody levels will then be calculated from DSA MFI levels detected in the same experiment for undiluted week-12, -24 and week-52 samples.

7.2. Immunoglobulin levels

Total IgG, IgM, IgA and IgG subclasses will be assessed in serum applying immunonephelometry on a BN™ II analyzer (Siemens Healthineers, Erlangen, Germany).

7.3. Pharmacokinetics and Immunogenicity

For the determination of felzartamab serum concentrations (PK) and the potential development of anti-falzartamab antibodies (ADA), serum sample will be collected throughout the course of the study (i.e. 2 x 1 mL serum per parameter and collection time point labelled as A- and B-samples). The assessment will be performed with drug specific validated ligand binding assays (ELISA and MSD based). The actual dates and times of each PK and ADA blood sampling will be recorded in the CRF.

7.4. Transplant biopsies

Follow-up biopsies will be performed at weeks 24 and 52 (end-of-study visit), after exclusion of a coagulation disorder or platelet counts below 80%. Anticoagulants or inhibitors of thrombocyte aggregation will be transiently paused. The biopsy will be performed under local anaesthesia (lidocain) using ultrasound-guided percutaneous techniques (1-2 cores per biopsy, 16 gauge needle). After biopsy, patients will be monitored closely for 5 to 8 hours for any complications (serial blood pressure measurements, monitoring for hematuria, haemoglobin check 4 hours after biopsy). Histomorphology will be evaluated on paraffin-embedded sections applying standard methodology. For immunohistochemical C4d staining, we will use a polyclonal anti-

C4d antibody (BI-RC4D, Biomedica; Vienna, Austria), and, following the rules of the Banff scheme [37], minimal immunohistochemical staining (C4d Banff score ≥1) along peritubular capillaries will be considered positive. Biopsies will also be evaluated by electron microscopy for detection of multilayering of peritubular capillary basement membranes (MLPTC). Morphological results will be read locally (Medical University of Vienna, Charité Universitätsmedizin Berlin; Grenoble Alpes University Hospital) in a blinded fashion. At the end of the trial, biopsies will be scanned and re-evaluated by a central pathologist (Heinz Regele, Medical University of Vienna). In addition, all biopsies will also be analyzed using microarrays as also proposed by the Banff scheme, using the internationally validated Molecular Microscope® Diagnostic System MMDx platform [35,38], For each biopsy, a 3 mm portion of one core will be placed immediately in RNAlater, stored at -20°C and shipped at ambient temperature or dry ice to the Alberta Transplant Applied Genomics Centre (ATAGC, University of Alberta, Edmonton, AB, Canada) for gene array analysis. Thoroughly validated molecular scores based on machine-learning derived lesion-based classifiers related to rejection [ABMR, T cell-mediated rejection (TCMR), all Rejection], inflammation (global disturbance score) or chronic injury (atrophy/fibrosis score) will be generated using a reference set of 1529 biopsies. Moreover, gene expression patterns will be evaluated using unbiased archetype analysis [39]. For classification of ABMR according to the Banff 2019 scheme, all biopsy results will be analyzed in the context of the molecular results. ABMR categories and morphological single lesions will be defined and scored following the 2019 update of the Banff classification [37]. ABMR will be defined on the basis of both morphological (histomorphology, immunohistochemistry, electron microscopy) and thoroughly validated molecular criteria: (i) evidence of acute or chronic tissue injury, (ii) evidence of current/recent antibody interaction with the vascular endothelium, and (iii) serological evidence of DSA.

7.5. Kidney function

eGFR will be assessed using the Chronic Kidney Disease Epidemiology Collaboration (CKD-EPI) equation (mL/min/1.73m^2). Protein excretion will be documented as protein/creatinine ratio in spot urine (mg/g).

7.6. Immunologic biomarkers

For chemokine detection we will use Luminex-based protocol as described earlier [40]. For quantification of *chemokine (C-X-C motif) ligand (CXCL)9 and CXCL10*, serum samples will be adjusted to 10 mM EDTA to prevent complement interference. Undiluted samples will be measured in duplicates using multiplexed Human ProcartaPlex Simplex Immunoassays (Thermo Fisher Scientific, Waltham, MA, USA) according to the manufacturer's instructions. Immunoassays will be performed on a Luminex 200 instrument (Luminex Corp., Austin, Tx, USA). Urinary results will be normalized to creatinine excretion and presented as pg (chemokine)/mg (creatinine). Levels of dd-cf DNA in recipient plasma samples reflecting the extent of ongoing allograft injury will be detected using standard technology, based on the detection of a defined set of single nucleotide polymorphisms (SNPs) detected by NGS (next-generation sequencing) on an Illumina MiSeq sequencer (Illumina Inc, San Diego, CA, USA) [41]. BAFF levels will be detected using Luminex (or alternative ELISA technology) using commercial kits.

7.7. Leukocyte subpopulations

The underlying mechanisms of chronic antibody mediated rejection, especially the role of peripheral T- and B-cell subsets are not fully clarified. Thus, the prospective monitoring of immune phenotype under therapy with felzartamab is a promising approach to further elucidate the impact on immune-regulatory pathways when CD38 is targeted. Moreover, assessment of PC and NK cell counts allows for the monitoring of the pharamacodynamic effects of the CD38 antibody. For monitoring of leukocyte (sub)populations we will use reproducible immune monitoring (IM) panels for phenotyping. Recently, the international "The ONE study" consortium has designed a standardized panel (DuraClone®) for flow cytometry based immune phenotyping that demonstrated robust results [42,43]. In the DuraClone IM kits pre-defined assay tubes contain a layer with the dried-down antibody panel ready to use. Up to 10 different monoclonal antibodies per tube allows the identification of leukocyte (e.g. T cell, B cell, NK cell subsets) subpopulations present in whole blood samples. For flow cytometric assessment of CD38 expression, assays will be modified to include staining with non-crossreactive CD38 mAb.

7.8. Gene expression analysis

For gene expression analysis, 5 mL of blood will be collected in PAXgene Blood RNA tubes and stored at -80°C until retrospective analysis. These tubes are designed for stabilization of RNA in blood during long-term storage at ultra-low temperature. Gene expression pattern analyses (microarray analysis) will be performed from peripheral blood to evaluate the impact of felzartamab on antibody-producing cells, analyzing genes annotated as part of the B-cell receptor signalling pathway [44].

7.9. Torque Teno virus (TTV) quantification

For TTV analysis, DNA will be extracted from plasma using the NucliSENS easyMAG platform (bioMeriéux, France) and eluted in 50 µL of elution buffer. TTV DNA will be quantitated by TaqMan real time polymerase chain reaction (PCR), according to earlier described protocols [34,45,46]. The quantitative PCR reactions will be performed in a volume of 25 µL using 2 × TaqMan Universal PCR Master Mix, containing 5 µL of extracted DNA, 400 nM of each primer, and 80 nM of the probe. Thermal cycling will be started for 3 minutes at 50°C, followed by 10 minutes at 95°C, and then by 45 cycles at 95°C for 15 seconds, at 55°C for 30 seconds, and at 72°C for 30 seconds, using the CFX96 Real-time System (Bio-Rad, Hercules, CA). Results will be recorded as copies/mL.

7.10. Course of vaccination titers

Serum IgG titers specific for mumps, measles and rubella (MMR) will be analyzed by ELISA technique. Covid-19 antibody titers will be detected using commercial kits.

7.11. Collection of biological material (outside routine monitoring)

Upon screening, we will perform a Quantiferon test and assess HIV/hepatitis serology (1x15 mL). Plasma (10 mL blood; chemokines, BAFF levels, TTV load), serum (10 mL blood; HLA antibody studies), whole blood (10 mL blood; flow cytometry, RNA for gene expression analysis) and urine (10 mL) will be collected before study initiation (day 0), after 3, 6 and after 12 months (in total 4x35 mL peripheral blood). BAFF and leukocyte populations will in addition be evaluated at weeks 1, 2, and 4 (3x10 mL blood). For detection of Ig(sub)classes and CMV/HSV/HZV PCR, serum and plasma will be collected every 4 weeks (10 mL blood, total of 10 visits = 10x10 mL peripheral blood). Finally, for measurement of felzartamab concentrations and ADA, serum will be obtained at every study visit (5 mL peripheral blood per visit; first three felzartamab infusions: additional 5 mL after infusion; total of 17 visits = 14 x 5 mL + 3 x 10 mL peripheral blood). Biological material for retrospective evaluation will be aliquoted and stored for testing at the Department of Clinical Pharmacology.

7.12. Responsible Laboratories that are involved into the study

VIETAC LAb
Medical University of Vienna
Department of Medicine III
Waehringer Guertel 18-20
1090 Vienna, Austria

Department of Laboratory Medicine
Medical University of Vienna
Department of Medicine III
Waehringer Guertel 18-20
1090 Vienna, Austria

Institute of Pathology; Medical University of Vienna
Clinical Institute of Pathology
Waehringer Guertel 18-20
1090 Vienna, Austria

Molecular Microscope', MMDx
Alberta Transplant Applied Genomics Centre
Faculty of Medicine & Dentistry
#250 Heritage Medical Research Centre
University of Alberta
Edmonton, Alberta, Canada

Nephrologisches Forschungslabor Charité
Charité Berlin
Department of Nephrology
Charitéplatz 1
10117 Berlin, Germany

HLA Labor, Charité
Charité Berlin
HLA Laboratory
Augustenburger Platz 1
13353 Berlin, Germany

SGS France
90 Avenue des Hauts de la Chaume BP. 28
86281 Saint-Benoît Cedex
France

8. SAMPLE SIZE AND STATISTICAL ANALYSIS

Rationale for sample size

For this pilot study, no exact sample size estimation based on an efficacy test can be performed, because the effect size is unknown (there is no sufficient prior information to base a sample size on). An important aspect of this pilot trial is to evaluate whether there are any safety issues.

In a myeloma study [28] with n=53 patients who have received felzartamab with or without a steroid, the following AE were reported:

Table 5

**AE rates in the MM trial with with observed frequencies of > 10%**	**AE rate (%)**	**AE rates in the MM trial with with observed frequencies of <10%**	**AE rate (%)**
Infusion reaction	32.1	Pneumonia	5.7
Anemia	39.6	Obstipation	7.5
Leucopenia	37.7	CRP elevation	7.5
Fatigue	32.1	Rash	3.8
Thrombocytopenia	18.9	Cramps	9.4
Tachycardia	15.1	Muscle pain	9.4
Headache	22.6		
Diarrhea	22.6		
Nausea	20.8		
Fever	15.1		

Table 6 gives the probability to observe at least 1 AE of a certain type depending on the actual sample size and the probability for an AE.

Table 6

	N=5	N=10	N=20
**Actual Probabiliy of Event, π=1%**			
Chance of Observing ≥1 Event, P	0.049	0.096	0.182
**Actual Probabiliy of Event, π=5%**			
Chance of Observing ≥1 Event, P	0.226	0.401	0.642
**Actual Probabiliy of Event, π=10%**			
Chance of Observing ≥1 Event, P	0.410	0.651	0.878
**Actual Probabiliy of Event, π=15%**			
Chance of Observing ≥1 Event, P	0.556	0.803	0.961

Furthermore when the sample size is 10, a two-sided 95% confidence interval for a single proportion will range from 0 to 0.072 when the sample proportion is 0,01. For proportion of 0.05, 0.1 and 0.15 see Table 6.

A preliminary assessment of efficacy outcomes (secondary endpoints) will provide first systematic results (including the extent of variation) on the effect of felzartamab on important molecular, morphological, immunological and clinical endpoints (measurement of the capacity of beneficial change). Our study results can be expected to provide a valuable foundation of the design of future trials. Analyses will be conducted according to the intention-to-treat principle.

For qualitative variables (e.g. occurrence of AE in the two treatment groups), absolute (n=) and relative frequencies will be calculated per treatment group. Data will be visualized by bar plots. Such nominal data will be compared using the Fisher's exact test for descriptive purposes. Transplant and patient survival or AE (SAE)-free survival will be evaluated using Kaplan Meier analysis and the log rank test will be applied for group comparisons. For quantitative data (e.g. group comparisons for DSA levels, g+ptc score, IF/TA score, molecular rejection scores, eGFR and protein/creatinine ratio after 3 months), the number of valid observations (n =), mean, standard deviation, standard error, median, minimum and maximum will be calculated for each treatment

group and each time point separately. Data will be visualized by boxplots and histograms. For descriptive purposes only such continuous data will be analysed using parametric or non-parametric (independent/dependent data) tests. These include the Mann Whitney U test or the t-test, as appropriate. For paired data (e.g. difference in IF/TA score between month 0 and month 12), paired t test or Wilcoxon test will be used as appropriate.

Analysis of pharmacokinetics of felzartamab will include a description of the time evolution of antibody/drug concentration. For some study endpoints, data on variability and center (median) that can be expected also for the present study, are available from a recent interventional randomized controlled trial performed at our unit, which included a similar cohort of kidney transplant recipients diagnosed with late ABMR (BORTEJECT, NCT01873157) [14]. The respective data obtained in study groups are listed in the table below:

Table 7. Median & variability of key endpoints – Results of BORTEJECT

Endpoints	All patients	n	Placebo (n=23)	Bortezomib (n=21)
**HLA DSA after 1 yr**				
Immunodominant DSA	3553 (1012-10365; 163-16284)	39	4360 (1080-10226; 778-16284)	2054 (918-10968; 163-14461)
Sum of DSA MFI	4605 (1119-12009; 273-33855)	39	5410 (1354-13612; 778-28828)	3479 (929-12009; 273-33855)
**Follow-up biopsy (24 mo)**				
g+ptc score	2 (1-5; 0-6)	38	2 (1-5; 0-6)	4 (0-5; 0-6)
IF/TA score	3 (2-5; 0-6)	38	3 (2-4; 0-6)	3 (2-5; 0-6)
Molecular ABMR score	0.61 (0.30-0.89; 0.05-0.99)	37	0.58 (0.24-0.89; 0.08-0.99)	0.77 (0.41-0.90; 0.05-0.97)
**Kidney parameters after 1 yr**				
eGFR, ml/min/1.73 m2	44 (23-72; 15-95)	43	53 (23-81; 15-95)	42 (25-69; 15-89)
Protein/creatinine ratio, mg/g	201 (86-1249; 0-4863)	44	194 (82-959; 0-4863)	208 (100-1427; 0-2972)

GFR trajectories will be analyzed using a linear mixed model with eGFR values from 0 to 52 weeks (week 0, 4, 8, 12, 16, 20, 24, 28, 32, 36, 40, 44, 48, 52) as dependent variable. Time and treatment as well as their interaction will be used as fixed effects. Furthermore, patient-specific random effects for intercept and slope will be specified.

If p-values are presented, this is done for exploratory purposes only as this is a pilot study. Therefore only unadjusted p-values are presented and no correction for multiplicity will be applied. For statistical analysis, IBM SPSS Statistics version 24 (IBM

Corporation, Armonk, NY, USA) and SAS version 9.4. (The SAS Institute Inc., Cary, North Carolina, USA) will be applied.

9. INTERIM ANALYSES

The trial will be monitored by an independent data and safety monitoring board (DSMB) to assess the safety and data quality. To timely detect major differences between groups in terms of safety aspects, the board members will be instructed to perform interim analyses after 10 and 20 patients have been randomized and entered the trial (depending on the duration of recruitment period, for interim analyses a complete patient follow-up may not be available). The DSMB will analyze recorded AEs and safety lab results in relation to the randomization sequence, and may consider stopping the trial if the overall pattern of related serious adverse events (SAEs) or changes in safety lab results strongly support a major safety signal. Exact statistical definitions of criteria for premature study termination are not defined.

10. EXTENDED OBSERVATION AND TREATMENT OPTIONS

Following the completion, data base lock and unblinding of the randomized controlled phase of the trial, the study can optionally be extended to a 5-year observational period (Option A) and/or, in the event of sufficient safety and potential efficacy of felzartamab, to a subsequent 1-year uncontrolled open-label treatment phase (Option B).

Results of the first study period[49]

Twenty-two patients were included in the first study period. Twenty-one patients (felzartamab: n=11; placebo: n=10) completed the trial and had 24-week and 52-week follow-up biopsies. One patient in the placebo group experienced graft loss at week 14 due to persistent chronic active ABMR and was not included in the efficacy analysis. Nine of 11 patients (82%) in the felzartamab arm versus 2 of 10 patients (20%) in the placebo arm exhibited resolution of ABMR in the follow-up biopsy at week 24 (defined as either chronic [inactive] ABMR or no ABMR). Felzartamab led to a significant reduction in median microvascular inflammation scores at week 24 and 7/11 patients (64%) in the felzartamab group achieved a microvascular inflammation score of zero (versus 1 of 10 patients [10%] in the placebo group). Biopsies at week 52 (6 months after completion of the treatment) indicated recurrence of ABMR activity in 3 of the 9 felzartamab responders[49].

Biomarkers in peripheral blood indicating ongoing allograft injury: donor-derived cell-free DNA (dd-cfDNA)

At baseline, median dd-cfDNA levels were not statistically different between groups (felzartamab: 1.19% [IQR: 0.71 – 2.29%] vs. placebo: 0.63% [IQR: 0.4 – 1.35%]). In patients allocated to felzartamab treatment, dd-cfDNA levels decreased considerably while patients were on active treatment. At week 24, the median fraction of donor-derived cell-free DNA was 0.31% (IQR: 0.21 to 0.49) in the felzartamab group vs. 0.82% (IQR: 0.34 to 2.90%) in the placebo group. At week 52, median levels of dd-cfDNA of felzartamab-treated patients increased toward baseline levels. Changes in absolute dd-cfDNA copy numbers of felzartamab-treated patients followed similar

patterns[49]. Importantly, felzartamab-treated patients with recurrence of ABMR activity at week 52 had higher biomarker levels compared to patients who had no recurrence of ABMR activity (felzartamab-treated patient with no ABMR activity at week 52: 0.58% [IQR: 0.24 – 0.98%] vs. felzartamab-treated patients with ABMR activity at week 52: 0.91% [0.51 – 1.91%]).

Observation period (Option A)

The observational extension within the trial will provide information on the treatment's long-term performance and its impact on transplant function, graft survival, and patient outcomes over a period of 5 years. After obtaining informed consent, all 22 study participants will be given the opportunity to participate in a prospective 5-year observation phase. Annual visits will be conducted after the completion of the blinded part of the randomized trial phase. This will allow for the documentation of the clinical course of all patients for the duration of 5 years. Four study visits are scheduled at yearly intervals, specifically at 24, 36, 48, and 60 months after trial inclusion. Optionally, these visits can be integrated into routine clinical care and the study visits can be scheduled to align with regular routine appointments, thereby minimizing any additional burden on the patients and optimizing the use of existing healthcare resources. At scheduled time points, eGFR, protein/creatinine ratio, routine laboratory parameters (blood picture and blood chemistry) and immunosuppressant levels (e.g. CNI trough level) as well as DSA specificity/levels, circulating dd-cfDNA fractions/levels, chemokine and BAFF levels, TTV viremia, leukocyte(sub)populations and gene expression analysis from peripheral blood and urine will be evaluated. In addition, medical events, such as graft loss, death and hospitalizations as well as immunosuppressive medication will be recorded (see schedule of events – Table 8).

Table 8. Schedule of events - Observational extension / Option A

Visit	Visit A1	Visit A2	Visit A3	Visit A4
Month	24	36	48	60
Graft loss (date, primary cause)	X	X	X	X
Death (date, primary cause)	X	X	X	X
Hospitalizations (primary reason)	X	X	X	X
Indication biopsies (results)	X	X	X	X
Immunosuppressive medication	X	X	X	X
Blood count, chemistry	X	X	X	X
Immunosuppressant level	X	X	X	X
eGFR (ml/min/1.73 m2)	X	X	X	X
Protein/creatinine ratio (mg/g)	X	X	X	X
Urine biomarkers	X	X	X	X
DSA (MFI, specificity)	X	X	X	X
dd-cfDNA (%, absolute levels)	X	X	X	X
Chemokines	X	X	X	X
BAFF levels	X	X	X	X
Gene expression analysis	X	X	X	X
iBOX score	X	X	X	X
Biobanking (Plasma, plasma in Streck tubes, serum, blood RNA, urine)	X	X	X	X

Open label treatment option (Option B)

After the completion of the 1-year randomized controlled phase of the trial and data base lock, the study will be unblinded, and the randomization sequence will be assessed in terms of safety data and major efficacy endpoints, including follow-up biopsy results and levels of donor-specific antibodies (DSA). Based on the safety and efficacy results, patients with functioning transplants and ongoing active rejection (active or chronic active ABMR) will be given the opportunity to receive "open-label" treatment with felzartamab (eligibility criteria: see Table 9). The conditions for proceeding with this option are twofold:

(i) There must be clear evidence of a treatment effect on the biopsy level, analyzed in relation to the randomization sequence.

(ii) There should be a demonstration of sufficient tolerability and safety throughout the entire duration of the study.

Before considering the option of extended treatment for participants, the results of the initial phase of the trial will undergo a thorough evaluation by the Data and Safety Monitoring Board (DSMB). The DSMB will independently decide whether study participants are eligible to enter the open-label phase. For those who meet the eligibility criteria (Table 9), and after providing informed consent, patients will undergo 6 months of felzartamab treatment. To participate in Option B, consent for Option A is essential.

Table 9. Inclusion and exclusion criteria for open label phase

**INCLUSION CRITERIA**
1. Voluntary written informed consent
2. Finalization of the randomized controlled trial phase (placebo or felzartamab arm)
3. Functioning allograft with an eGFR ≥15 ml/min/1.73 m^2 (CKD-EPI formula)
4. Active or chronic active ABMR in the 12-month biopsy for patients in both the placebo and felzartamab arms (for the latter, provided that there is a signal regarding pharmacodynamics (NK cell depletion or DSA reduction) and/or a 6-month biopsy showing a response to the therapy; reduction in the extent of MVI) or evidence of active ABMR in an indication biopsy conducted after the completion of the study.
**EXCLUSION CRITERIA**
1. Participation in another clinical trial
2. Female subject is pregnant or lactating or not on adequate contraceptive therapy
3. Index biopsy results:<br>T-cell-mediated rejection classified Banff grade ≥I<br>*De novo* or recurrent severe thrombotic microangiopathy<br>Polyoma virus nephropathy<br>*De novo* or recurrent glomerulonephritis
4. Total bilirubin >2×the upper limit of normal [ULN], alanine transaminase and aspartate aminotransferase >2·5×ULN
5. Haemoglobin <8 g/dL
6. Thrombocytopenia: Platelets <100 G/L
7. Leukopenia: Leukocytes <3 G/L
8. Neutropenia: Neutrophils < 1.5 G/L
9. Active viral, bacterial or fungal infection precluding intensified immunosuppression
10. Active malignant disease precluding intensified immunosuppressive therapy
11. Latent or active tuberculosis (positive QuantiFERON-TB-Gold test)
12. Administration of a live vaccine within 6 weeks of screening
13. History of alcohol or illicit substance abuse
14. Serious medical or psychiatric illness likely to interfere with participation in the study

Therapy regimen (analogous to the randomized controlled phase of the trial; see chapter 5.1.):

- 16 mg/kg Felzartamab IV per infusion (first two infusions administered slowly over approximately 90 min as described in chapter 5.1.)
- In case of infusion reaction: see 5.1.
- Premedication with diphenhydramine (30 mg), paracetamol (1000 mg), and prednisolone (100 mg) before the first two infusions (see 5.1.)

Infusions are, as in the first phase of the trial, planned at day 0, after 1, 2, 3, 4, 8, 12, 16, and 20 weeks After week 24, felzartamab treatment will be administered as individualized “on-demand” therapy, based on the dynamics of individual biomarker levels (dd-cfDNA).

<u>Rationale and criteria for biomarker-guided (dd-cfDNA-guided) prolonged felzartamab therapy</u>

After discontinuation of felzartamab in the first study period, histomorphologic recurrence of ABMR activity was observed in a sub-cohort of felzartamab-treated patients in the follow-up biopsy at week 52, as described above. Accordingly, some patients may benefit from a prolonged, individualized felzartamab treatment period beyond week 24 to maintain resolution of ABMR at week 52. Dd-cfDNA has been used as a biomarker for ABMR activity during the first study period of the trial. Dd-cfDNA levels were found to associate with the histomorphologic response to felzartamab treatment, as discussed above. In this second study period (trial extension), patients will be subjected to an individualized regimen of felzartamab beyond week 24. Treatment (single doses of felzartamab) will be administered in the case of elevated biomarker levels (dd-cfDNA). Thresholds for dd-cfDNA for the detection of allograft rejection have been extensively studied and were defined previously[50,51].

Elevated dd-cfDNA is defined as one or more of the following criteria:

- dd-cfDNA level above **relative** threshold (≥0.5%)
- dd-cfDNA level above **absolute** threshold (≥ 50 copies/ml)
- Doubling in dd-cfDNA **relative** levels as compared to week 24

- Doubling in dd-cfDNA **absolute** levels as compared to week 24

The ultimate goal of this individualized, biomarker-guided on-demand therapy is that all felzartamab-treated patients show complete resolution of ABMR activity at the end of the second study period (week 52).

Visits (see Table 10): Screening visit, followed by visits at Day -7 (optional) and until Week 52 (in total 19 visits including two optional visits)

Efficacy monitoring (analogous to the first phase of the trial):

- Renal function (eGFR) and proteinuria
- Graft loss and death
- DSA (Donor-Specific Antibodies)
- dd-cfDNA
- Chemokines, BAFF
- TTV viremia
- Leukocyte (Sub)populations
- Gene expression analysis from peripheral blood
- Urine biomarkers
- iBOX Score
- Follow-up biopsies (Histomorphology and MMDX) after 24 and 52 weeks

Pharmacokinetics and Immunogenicity monitoring:
For the determination of felzartamab serum concentrations (PK) and the potential development of anti-felzartamab antibodies (ADA), serum sample will be collected throughout the course of the study (i.e. 2 x 1 mL serum per parameter and collection time point labelled accordingly). The assessment will be performed with drug specific validated ligand binding assays (ELISA and MSD based). The actual dates and times of each PK and ADA blood sampling will be recorded in the CRF.

Safety assessment:

- According to the rules described in chapter 11.

Associated risks and potential adverse events:

- Adverse events that could be potentially associated with felzartamab or premedication: see chapter 11.2
- Renal allograft biopsies: see chapter 7
- Risks associated with bone marrow aspiration:
  The most common risks include pain or discomfort at the aspiration site, bruising or bleeding, and a small risk of infection. In rare cases, there may be damage to surrounding structures or nerves. Additionally, some patients may experience an allergic reaction to the local anesthetic used during the procedure.

Statistical analysis:

- Descriptive statistics (see chapter 8).
  Comprehensive description of the course of efficacy endpoints AEs/SAEs documented during the whole trial (including patients in in Option A or B) (event rate; AE types according to MedDRA)
- Statistics will also include a combined analysis of the course of efficacy endpoints obtained in phase 1 (felzartamab arm) and placebo patients subjected to felzartamab treatment in the extended phases (analysis of efficacy endpoints over 12 months)

Table 10. Schedule of events – open-label treatment with felzartamab

Visit	Screen	B1a	B1	B2	B3	B4	B5	B6	B7	B8	B9	B10a	B10	B11	B12	B13	B14	B15	B14	B17
**Week**	**up to -4**	**-1**	**0**	**1**	**2**	**3**	**4**	**8**	**12**	**16**	**20**	**23**	**24**	**28**	**32**	**36**	**40**	**44**	**48**	**52**
Informed consent	x																			
Physical examination	x		x										x							x
Concomitant medications	x		x	x	x	x	x	x	x	x	x		x	x	x	x	x	x	x	x
Pregnancy test, if applicable	x		x	x	x	x	x	x	x	x	x		x	x	x	x	x	x	x	x
Virology	x		x										x							x
ECG	x		x										x							x
**Felzartamab**			x	x	x	x	x	x	x	x	x			x[1]	x[1]	x[1]	x[1]	x[1]	x[1]	
**Safety**																				
Temperature/heart rate/blood pressure			x	x	x	x	x	x	x	x	x		x	x	x	x	x	x	x	x
AE monitoring			x	x	x	x	x	x	x	x	x		x	x	x	x	x	x	x	x
Safety Lab			x	x	x	x	x	x	x	x	x		x	x	x	x	x	x	x	x
Ig (sub) classes			x				x	x	x	x	x		x		x		x			x
CMV/HSV/HZV PCR			x				x	x	x	x	x		x		x		X			x
Vaccination titers			x						x				x							x
TTV copy number			x						x				x							x
**PK and Immunogenicity**																				
Felzartamab serum concentration		x[2]	x[2]	x[2]	x[3]	x[3]	x[3]	x[3]	x[3]	x[3]	x		x	x	x	x	x	x		
Anti-felzartamab antibodies		x[3]	x[3]			x[3]		x[3]			x			x				x		
**Efficacy assessment**																				
Follow-up biopsies													x							x
Bone marrow aspiration		x[4]										x[4]								
DSA detection			x						x				x							x
Chemokines			x	x	x	x	x	x	x	x	x		x	x	x	x	x	x	x	x
BAFF levels			x	x	x	x	x	x	x	x	x		x	x	x	x	x	x	x	x
Gene expression analysis			x						x				x							x
dd-cfDNA			x	x	x	x	x	x	x	x	x		x	x	x	x	x	x	x	x
Leukocyte subpop.			x	x	x	x	x	x	x	x	x		x	x	x	x	x	x	x	x
eGFR (CKD-EPI)			x	x	x	x	x	x	x	x	x		x	x	x	x	x	x	x	x
Protein/creatinine ratio			x	x	x	x	x	x	x	x	x		x	x	x	x	x	x	x	x
Urine biomarkers			x						x				x							x
iBOX Score			x						x				x							x

**CNI/mTOR levels**			x	x	x	x	x	x	x	x	x		x	x	x	x	x	x	x	x
**Biobanking** (Plasma, serum, blood RNA, urine)			x						x				x							x

[1] Felzartamab treatment is provided as a single dose (16 mg/kg) depending on biomarker levels (dd-cfDNA).
[2] PK and Immunogenicity: x[2]: pre-dose and 30 min after end of infusion
[3] PK and Immunogenicity: x[3]: pre-dose only
[4] As an optional procedure, up to 8 subjects who have provided additional informed consent will undergo bone marrow aspiration before starting felzartamab treatment. Subsequently, another follow-up bone marrow aspiration will be performed after 24 weeks of treatment. The primary objective of these bone marrow aspirates is to investigate the impact of felzartamab therapy on HLA antibody-producing plasma cells within the bone marrow. This optional sub-analysis aims to achieve a better understanding of the underlying mechanisms involved in the treatment's effectiveness. To achieve this, advanced techniques like single-cell sequencing and tracking of B-cell receptors/clonality will be employed, along with clustering assignment methodologies [https://doi.org/10.21203/rs.3.rs-2378630/v1].

Formularbeginn

Formularende

11. ADVERSE EVENTS

11.1. Felzartamab

Safety data from the first study period showed that felzartamab had an acceptable safety and side-effect profile[49]. AE were reported in all the patients in both treatment groups and were predominantly mild or moderate in severity (Table 11). A greater incidence of treatment-emergent AE was reported in the felzartamab group compared to the placebo group (119 vs. 81 events), as were treatment-related AE (felzartamab: 27 events vs. or placebo: 11 events). No patient discontinued treatment because of AE. There were no fatal AE. The frequency of serious AE, which were primarily infection-related, was lower in the felzartamab group than in the placebo group (placebo: 4 patients [36%]; felzartamab: 1 patient [9%]).
Mild or moderate infusion-related reactions occurred in 8 patients (73%) in the felzartamab group. There events were limited to the first infusion of felzartamab.

Table 11. Treatment-emergent adverse events

	Placebo (N=11)		Felzartamab (N=11)	
	Patients with AE – no. (%)	Number of AE	Patients with AE – no. (%)	Number of AE
**Patients with a TEAE – no. (%)**	11 (100)	81	11 (100)	119
**Mild**	9 (81.8)	37	11 (100)	61
**Moderate**	11 (100)	42	11 (100)	55
**Severe**	1 (9.1)	2	2 (18.2)	3
**Patients with a TRAE – no. (%)**	7 (63.6)	11	10 (90.9)	27
**Infusion-related reaction**	0 (0)	0	8 (72.7)	8
**Serious AE (SAE) – no. (%)**	4 (36.4)	7	1 (9.1)	2
**COVID-19 pneumonia**	2 (18.2)	2	0 (0)	0
**Urinary tract infection**	2 (18.2)	2	0 (0)	0
**Hyponatremia**	1 (9.1)	1	0 (0)	0
**RSV infection**	1 (9.1)	1	0 (0)	0
**Clostridium difficile diarrhea**	1 (9.1)	1	0 (0)	0
**Acute kidney injury**	0 (0)	0	1 (9.1)	1

**Viral keratoconjunctivitis**	0 (0)	0	1 (9.1)	1

~~Until now, no data obtained in transplant patients on dual or triple immunosuppression are available, so that safety assessment remains a major objective of this pilot trial.~~ Other safety data of felzartamab were obtained in a clinical phase 1-2a trial, in a cohort of 91 patients with refractory or relapsing MM [28]. Treatment-emergent adverse events under felzartamab monotherapy or felzartamab combined with dexamethasone are provided in Table 12. The results of combined treatment with pomalidomide and lenalidomide, which may not be relevant for our planned trial, are not included in this Table (full lists of AE are provided in reference 28).

Table 12. Treatment-emergent adverse events (phase 1-2a trial/MM; IB/Table 22)

**Incidence[a] (%) All grades**					Incidence[a] (%) grade ≥ 3			
**Preferred Term**	**MOR202+ MOR202/ DEX N=53**	**MOR202/ POM/ DEX N=21**	**MOR20 2/ LEN/ DEX N=17**	**Overall N=91**	**MOR202+ MOR202/ DEX N=53**	**MOR202/ POM/ DEX N=21**	**MOR20 2/ LEN/ DEX N=17**	**Overall N=91**
**Blood and lymphatic system disorders**								
Leukopenia[b]	37.7%	85.7%	88.2%	58.2%	13.2%	57.1%	47.1%	29.7%
Neutropenia[d]	24.5%	90.5%	70.6%	48.4%	11.3%	81.0%	58.8%	36.3%
Lymphopenia[c]	30.2%	66.7%	76.5%	47.3%	26.4%	52.4%	64.7%	39.6%
Anemia[f]	39.6%	47.6%	35.3%	40.7%	9.4%	9.5%	17.6%	11.0%
Thrombocytopenia[e]	18.9%	66.7%	64.7%	38.5%	9.4%	23.8%	17.6%	14.3%
**Cardiac disorders**								
Tachycardia	15.1%	4.8%	5.9%	11.0%	0%	0%	0%	0%
**Gastrointestinal disorders**								
Diarrhea	22.6%	52.4%	58.8%	36.3%	0%	9.5%	11.8%	4.4%
Constipation	7.5%	23.8%	64.7%	22.0%	0%	0%	0%	0%
Nausea	20.8%	9.5%	35.3%	20.9%	0%	0%	0%	0%
**General disorders and administration site conditions**								
Fatigue	32.1%	57.1%	35.3%	38.5%	0%	0%	5.9%	1.1%
Pyrexia	15.1%	28.6%	11.8%	17.6%	0%	0%	0%	0%
Oedema peripheral	9.4%	9.5%	23.5%	12.1%	0%	0%	0%	0%
**Investigations**								
C-reactive protein increase[d]	7.5%	14.3%	17.6%	11.0%	0%	4.8%	0%	1.1%
**Infections and infestations**								
Nasopharyngitis	30.2%	19.0%	35.3%	28.6%	0%	0%	0%	0%
Upper respiratory tract infection	20.8%	23.8%	35.3%	24.2%	0%	4.8%	0%	1.1%
Respiratory tract infection	7.5%	42.9%	23.5%	18.7%	0%	19.0%	0%	4.4%
Pneumonia	5.7%	33.3%	17.6%	14.3%	3.8%	28.6%	5.9%	9.9%
Bronchitis	15.1%	4.8%	5.9%	11.0%	1.9%	0%	0%	1.1%
**Injury, poisoning and procedural complications**								
Infusion related reactions[g]	32.1%	4.8%	5.9%	20.9%	1.9%	0.0%	0.0%	1.1%
**Metabolism and nutrition disorders**								
Hypokalemia	15.1%	28.6%	35.3%	22.0%	0%	9.5%	0.0%	2.2%
**Musculoskeletal and connective tissue disorders**								
Muscle spasms	9.4%	23.8%	52.9%	20.9%	0%	0%	0%	0%
Back pain	13.2%	33.3%	23.5%	19.8%	0.0%	0.0%	5.9%	1.1%
Myalgia	9.4%	19.0%	11.8%	12.1%	0%	0%	0%	0%
Pain in extremity	13.2%	4.8%	11.8%	11.0%	0%	0%	0%	0%
**Nervous system disorders**								
Headache	22.6%	9.5%	5.9%	16.5%	0%	0%	0%	0%
Dizziness	11.3%	4.8%	29.4%	13.2%	0%	0%	0%	0%
**Psychiatric disorders**								
Insomnia	15.1%	4.8%	5.9%	11.0%	0%	0%	0%	0%
**Respiratory, thoracic and mediastinal disorders**								

Cough	15.1%	23.8%	29.4%	19.8%	0%	0%	0%	0%
Dyspnoea	15.1%	4.8%	5.9%	11.0%	3.8%	0%	0%	2.2%
**Skin and subcutaneous tissue disorders**								
Rash	3.8%	23.8%	29.4%	13.2%	0%	0%	0%	0%
**Vascular disorders**								
Hypertension	13.2%	23.8%	11.8%	15.4%	9.4%	19.0%	11.8%	12.1%

N: number of patients; [a]Incidence: percentage of patients; [b]Includes White blood cell count decreased / Leukopenia; [c]Includes Lymphocyte count decreased / Natural killer cell count decreased / CD4 lymphocytes decreased / B-lymphocyte count decreased / Lymphopenia; [d]Includes Neutrophil count decreased / Neutropenia; [e]Includes Platelet count decreased / Thrombocytopenia; [f]Includes Red blood cell count decreased / Hemoglobin decreased / Hematocrit decreased / Mean cell volume decreased /Anemia. [g]One infusion-related reaction was related to comedication and was considered to be not related to felzartamab.

The most common grade 3 or higher treatment-emergent AE were hematologic and included lymphopenia (in 36 [39.6%] of 91 patients; whole study, including combinations with pomalidomide and lenalidomide), neutropenia (in 33 [36.3%] patients), leukopenia (in 27 [29.7%] patients), thrombocytopenia (in 13 [14.3%] patients) and anemia (in 10 [11.0%] patients) which were reported at higher incidences in patients treated with felzartamab plus an immunomodulatory drug than in patients treated with felzartamab with or without dexamethasone. The most common grade ≥3 non-hematologic TEAEs were hypertension (in 11 [12.1%] patients), pneumonia (in 9 [9.9%] patients), respiratory tract infection (in 4 [4.4%] patients), and diarrhea (in 4 [4.4%] patients).Infusion-related reactions were reported in 18 (19.8%) of 91 patients (in one case, infusion-related reaction was related to a concomitant medication / not felzartamab-related) ; infusion-related reactions graded ≥3 in one patient [1.1%]. Infusion-related reactions were less frequent (7%) among patients who received felzartamab in combination with dexamethasone (with or without combinations with lenalidomide or pomalidomide). This might be attributed to a preventive effect of dexamethasone.

11.2. Adverse drug reactions - specific considerations

11.2.1. Felzartamab

Opportunistic Infections and Serious Infections

During the first study period, the rate of infections was higher in felzartamab-treated patients compared to placebo (felzartamab n=10 patients [91%] versus placebo n=7 patients [64%]). Felzartamab-treated patients had a greater incidence of nasopharyngitis (n=9 patients [82%] versus placebo: n=3 patients [27%]). However,

serious adverse events related to infections were lower in the felzartamab group (felzartamab: 1 patient [9%] vs. placebo: 4 patients [36%]). One patient in the felzartamab group had cytomegalovirus (CMV) viremia, one patient had herpes simplex 2 viremia. There were no episodes of herpes simplex virus 1 viremia, varizella zoster viremia or polyomavirus viremia[49].

In the phase 1-2a trial of felzartamab performed in MM patients the rate of serious infection-related AE was considerably low [28]. In addition, large phase 3 trials evaluating daratumumab in MM rates of serious infections did not or only slightly increase under antibody treatment [47,48]. Currently, however, no systematic data are available for the use of CD38 mAb as a rejection treatment in organ transplant recipients on multi-compound baseline immunosuppression, and intensified immunosuppression, in addition to standard dual or triple baseline immunosuppression, can be expected to be associated with an increased infection risk. Hence, a careful patient follow-up will include a close monitoring for infectious complications (bacterial, viral and fungal infections).

Considering a potentially increased risk of viral infections, patients under felzartamab will be regularly monitored for cytomegalovirus (CMV), herpes simplex virus (HSV) and herpes zoster virus (HZV) viremia. Moreover, patients will be monitored for IgG levels and substituted with intravenous immunoglobulin in case of severe hypogammaglobulinemia (IgG below 400 mg/dL). Physicians should exercise caution when considering the use of felzartamab in patients with a history of recurring infection or with underlying conditions (eg, diabetes) which may predispose patients to infections (see also inclusion/exclusion criteria). Felzartamab should not be administered in patients with active infection. The effects of felzartamab on neutrophil counts, and possibly signs and symptoms of infection should be considered when evaluating a patient for a potential infection. Patients must be instructed to contact their physician immediately when any symptoms suggesting infection appear, in order to assure rapid evaluation and appropriate treatment. If a patient develops a serious infection, administration of felzartamab is to be interrupted until the infection is controlled. The clinician should consider the benefit-risk before resuming treatment with felzartamab.

Hematologic Abnormalities

Decreases in leukocyte counts (including neutrophil and lymphocyte counts) and platelet counts have not been observed following treatment with felzartamab in kidney transplant patients on triple immunosuppression during the first study period (grade III or higher)[49]. Decreases in leukocyte (neutrophil and lymphocyte) and platelet counts have been observed following treatment with felzartamab as monotherapy and in combination with dexamethasone and/or immunomodulatory agents [28]. For patients with concomitant medications associated with hematologic toxicity, the reduction or interruption of the suspected medication is recommended prior to modifying felzartamab. Dosing guidance for neutropenia and thrombocytopenia are described in Tables 13 and 14.

Table 13. Neutropenia risk mitigation

ANC (cells/mm^3)	Action
> 1000	Maintain dose (in case of mild neutropenia, reduction of the daily dose of MMF/EC-MPA)
500 – 1000	Interrupt felzartamab dosing.<br>When ANC increases to > 1000, resume treatment
< 500	Discontinue felzartamab; start filgrastim biosimilar.
ANC = absolute neutrophil count	

Table 14. Thrombocytopenia risk mitigation

Platelet count (cells/mm^3)	Action
> 100,000<br>50,000 – 100,000	Maintain dose.<br>Interrupt felzartamab dosing.<br>When platelet count increases to > 100,000, resume treatment
< 50,000	Discontinue felzartamab.

Elevated Liver Enzymes and Hepatic Events

Elevations in ALT and AST have been observed during treatment with the study medications. Dosing recommendations for events of elevated liver enzymes are described in Table 15.

Table 15. Elevated liver enzymes – risk mitigation

Lab Value	Action

> 1 to 3x ULN	Dose modify concomitant transplant immunosuppressive drugs if appropriate<br>For persistent increases in this range, interrupt felzartamab until ALT/AST have normalized<br>Restart with treatment as clinically appropriate
> 3 to 5x ULN<br>(confirmed by repeat testing)	Interrupt felzartamab dosing until < 3x ULN and follow recommendations above for >1 to 3x ULN<br>For persistent increases > 3x ULN, discontinue felzartamab
> 5x ULN	Discontinue felzartamab

Infusion-related reactions

During the first study period (felzartamab in ABMR), infusion-related reactions were observed in 8/11 felzartamab-treated patients (73%). As of August 5th 2024, nine patients have been included into the open-label extension phase. Infusion-related reactions were detected in 8/9 patients (89%) during the open label phase[49].

In the felzartamab MM trial[52], overall, infusion-related reactions to felzartamab were reported in 18 (20%) of 91 patients (grade ≥3: 1.1%). Importantly, all occurred during the first treatment cycle. As detailed above (see also IB and Table 5), a higher proportion of patients in the steroid-free felzartamab monotherapy groups (14 [40%] of 35 patients; ten [32%] of 31 in the felzartamab q2w group and four [100%] of four in the felzartamab q1w group) had infusion-related reactions than those treated in the felzartamab combination groups (four [7%] of 56; two [11%] of 18 in the felzartamab with dexamethasone group, one [5%] of 21 in the felzartamab with dexamethasone plus pomalidomide group, and one [6%] of 17 in the felzartamab with dexamethasone plus lenalidomide group). Notably, in the felzartamab MM trial, rates of infusion-related reactions were reported at lower frequencies than those reported for two other CD38, daratumumab and isatuximab. Moreover, lower rates of such events under combined regimens with dexamethasone may point to a beneficial effect of steroid-based immunosuppression in transplant patients. However, available results reinforce that during the first cycle of felzartamab adequate pre-treatment including steroids is necessary to minimize the risk of infusion-related reactions.

Healthcare professionals administering felzartamab should be trained in the appropriate administrative procedures, be able to recognize the symptoms associated with potential anaphylactic or hypersensitivity reactions, and have the appropriate

medication available for immediate use in case of anaphylaxis or hypersensitivity reaction during or after administration of felzartamab. Healthcare professionals should also instruct patients to seek medical attention if they experience symptoms of a hypersensitivity reaction outside of the clinic.

If a patient has symptoms of anaphylaxis or serious hypersensitivity, or requires an interruption of the study drug because of symptoms of anaphylaxis or hypersensitivity, administration of felzartamab must be discontinued permanently. The patient should be treated according to the standard of care for management of the hypersensitivity reaction. A blood sample for the presence of anti-felzartamab antibodies should be obtained. Felzartamab should not be administered to subjects who have had any previous allergic reactions to monoclonal antibodies.

11.2.2. Premedication

Premedication for patients included prior to August 2024

Diphenhydramin-hydrochlorid

The administered single dose of dibondrin is low (30 mg) and is far below the maximum permitted daily dose. For diphenhydramin, the following side effects have been described: Diphenhydramin can frequently cause tiredness (≥1/10). Occasionally (≥1/1.000, <1/100) circulatory problems, dizziness, drowsiness, headaches, concentration and coordination disorders, muscle weakness, visual disturbances, increase in intraocular pressure, thickening of the bronchial secretion, a feeling of tightness in the chest, gastrointestinal complaints, impaired bladder function, dryness of the mouth, nose and throat. Rare side effects (≥1/10.000, <1/1.000) are palpitations, allergic skin reactions, skin irritations and light sensitivity of the skin. Changes in the blood count (neutropenia, leukopenia, thrombocytopenia,
Hemolytic anemia) are very rare (<1/10.000).

Paracetamol

The single administration of paracetamol in the planned dosage (1000 mg) rarely leads to side effects. Increases in liver parameters and malaise are rarely observed (1: 1000-1: 10000). Very rare side effects are (below 1: 10000): changes in blood counts (thrombocytopenia, leukocytopenia, neutropenia, agranulocytosis), allergic reactions (skin reactions, bronchospasm, anaphylactic shock).

Prednisolon

Short-term administration is considered to be unproblematic and usually causes no or only minor side effects. With the planned two administrations of intravenous prednisolone as part of premedication (100 mg each, at an interval of 14 days), the typical side effects known for long-term therapy, such as increased susceptibility to infection, changes in blood cell counts, endocrine disease, sodium retention, weight gain, osteoporosis, muscle atrophy, skin changes, eye diseases, diseases of the gastrointestinal tract (e.g. gastric ulcer, increase in liver parameters) or impaired wound healing, are therefore *not* to be expected. Short-term intravenous administration can occasionally lead to an increase in blood glucose, increases in blood pressure or restlessness or sleep disorders. Allergic reactions are extremely rare.

Premedication for patients included after August 2024

Due to the high rate of infusion-related reactions as discussed above, premedication will be administered according to the following regimen:

24 hours prior to study drug

- Paracetamol 1000 mg orally (PO) every 8 hours (Q8H)
- Nondrowsy histamine 1 (H1) antagonist: desloratadine 5mg

Desloratadine

Short term administration of desloratadine (5 mg) is considered to be unproblematic and usually causes no or only minor side effects. The following side effects have been described for desloratadine: Common (1-10%) headache, dry mouth and fatigue. Very rare (less than 1:10000) are muscle pain, hypersensitivity reactions, palpitations, hallucinations, dizziness, insomnia, abdominal pain or an increase in liver enzymes

Premedication 30 min prior to the first two infusions of felzartamab will be identical to the first study period (see above). A single dose of oral Famotidine 20 mg (Ulcusan) will be included in addition.

Famotidine

Short term administration of Famotidine (20 mg) is considered to be unproblematic and usually causes no or only minor side effects. The following side effects have been

described for famotidine: Common (1-10%) headache, dizziness, constipation, diarrhea. Occasionally (0.1%-1%) fatigue, taste disturbances, dry mouth, nausea, gastrointestinal complaints, loss of appetite, skin rash, itching, hives. Rarely (1:1000 - 1:10000) flatulence. Very rare (less than 1:10000) changes in blood count, interstitial pneumonia, convulsions, sensory disturbances, drowsiness, hair loss, allergic skin reactions, joint pain, muscle cramps, chest tightness, increase in liver enzymes, impotence, reversible mental disorders including depression, hallucinations, sleep disorders.

Modification of infusion rate for the first infusion of felzartamab

For the first dose, start infusion slowly and increase as shown below over 4 hours:

- 25 mL/hour for 1 hour, then
- 50 mL/hour for 1 hour, then
- 75 mL/hour for 1 hour, then
- 100 mL/hour

11.3. Covid-19

In the context of the trial, the study team will adhere to all measures currently prescribed by the authorities with regard to the COVID-19 pandemic, and will ensure that no resources required to combat the pandemic are tied up, that sufficient personnel will be available, and that the participants are not exposed to any additional risk of infection through their participation in the study. As mentioned in Section 10.1, Covid-19 antigen or PCR tests will be carried out at every visit (or before administration of study medication / placebo). In case of a positive test result, the study medication will not be administered, and patient care will follow current recommendations and guidelines.

11.4. Pregnancies and Women of Child Bearing Potential

There are no adequate well-controlled studies in pregnant or lactating women. All subjects of childbearing potential being treated with felzartamab (and their partners) must be informed of this risk, and use highly effective birth control, as defined in the study protocol. Under no circumstances shall felzartamab injection be administered to women known to be pregnant or lactating. All pregnancies must be reported to Hi-Bio within 24 hours and in accordance with SAE reporting procedures.

a) Women of childbearing potential and men with partners that are WOCBP must be using a highly effective acceptable method of contraception to avoid pregnancy throughout the study and for 6 months after the last dose of investigation product, in such a manner that the risk of pregnancy is minimized.
   For this study purpose, only following highly effective birth control methods are allowed (based on the CTFG-Working group on Contraception):
   - combined (estrogen and progestogen containing) hormonal contraception associated with inhibition of ovulation
     a) oral
     b) intravaginal
     c) transdermal
   - progestogen-only hormonal contraception associated with inhibition of ovulation:
     a) oral
     b) injectable
     c) implantable
   - intrauterine device (IUD)
   - intrauterine hormone-releasing system (IUS)
   - bilateral tubal occlusion
   - sexual abstinence
b) WOCBP must have a negative serum or urine pregnancy test (minimum sensitivity 25 IU/L or equivalent units of HCG) within 24 hours prior to the start of investigational product.
c) Women must not be breastfeeding.
d) Patients must be willing to use condoms as adequate barrier to minimize the risk for STDs transmission to their sex partner
e) Men must be willing to refrain from sperm donation during study participation and for 6 months after dosing has been concluded.

All subjects of childbearing potential being treated with felzartamab (and their partners) must be informed of this risk, and use highly effective birth control, as defined above.

All pregnancies must be reported to Hi-Bio within 24 hours and in accordance with SAE reporting procedures.

12. SAFETY ASSESSMENT AND REPORTING

All patients will be evaluated for safety and adverse events will be documented according to the procedures of the investigational site. The investigator will determine the relationship of the events to the administration of the investigational drug felzartamab. All serious adverse events (SAEs) and adverse event of special interest (AESIs) must be reported as described in this section. All nonserious adverse events must be collected as well and documented according to the procedures of the investigational site.

12.1. Safety Assessment

Following predefined safety assessments will be performed:

WOCBP (Women Of Childbearing Potential)

If the patient is a WOCBP, pregnancy tests (blood or urine; minimum sensitivity 25 IU/L of HCG) must be performed within 72 hours before the first infusion of felzartamab. Women of childbearing potential and men with partners that are WOCBP must be using a highly effective acceptable method of contraception to avoid pregnancy (s. section 9.2.). If the patient becomes pregnant, she must be discontinued from felzartamab treatment.

Standard of care

Laboratory tests (safety lab), assessment of vital signs [heart rate, blood pressure, body temperature, (in case of clinical symptoms monitoring of SpO2 and respiratory rate before and in 30 min intervals during and after infusion of the antibody) will be performed as part of routine standard of care as directed by the investigator. Laboratory specimens should be obtained before felzartamab infusion. Additional tests may be ordered if deemed necessary for monitoring the patient’s safety.

Renal transplant function

Renal function will be evaluated at every study visit. Estimated renal function (eGFR) will be determined by the CKD-EPI-formula. Additional surrogate parameter of transplant function will be assessed via protein excretion at predefined time points (see Table 3).

IgG monitoring

Patients will be monitored for IgG levels in serial intervals (every 4 weeks under felzartamab, every 8 weeks thereafter). In the case of severe hypogammaglobulinemia (IgG <400 mg/dL), treatment with IVIG will be permitted.

Covid-19-Screening

Before study inclusion and at every trial visit (and before administration of study medication/placebo), study participants will undergo Covid-19 antigen or PCR testing to exclude a SARS Cov-2 infection.

Monitoring for viremia

Patients will undergo CMV, HSV and HZV PCR monitoring in serial intervals (every 4 weeks under felzartamab, every 8 weeks thereafter).

Acute Rejection Episode

At every study visit renal transplant function will be evaluated in regard for acute rejection episode. Any clinical suspected rejection will be confirmed or excluded by renal transplant biopsy histology. Rejection will be classified by the actual BANFF classification from 2019. Incidence of biopsy-proven acute rejection will be reported as SAE.

C0 levels of immunosuppressant

At every visit, trough levels of immunosuppressant (CNI) will be measured. Medication of immunosuppressant will be adapted according to the defined target levels of corresponding immunosuppressive drugs. Every change of medication will be documented in the source data. Change of more than 50% of daily dosing under DCV+SOF will be reported as AE.

12.2. Definition of adverse events

An AE is any untoward or unfavorable medical occurrence in a clinical research study participant, including any abnormal sign (e.g. abnormal physical exam or laboratory finding), symptom, or disease, temporally associated with the participants' involvement in the research, whether or not considered related to participation in the research.

Adverse events include:

- Exacerbation of a pre-existing disease.
- Increase in frequency or intensity of a pre-existing episodic disease or medical condition.
- Disease or medical condition detected or diagnosed after study drug administration even though it may have been present prior to the start of the study.
- Continuous persistent disease or symptoms present at baseline that worsen following the start of the study.
- Lack of efficacy in the acute treatment of a life-threatening disease.
- Events considered by the investigator to be related to study-mandated procedures.
- Abnormal assessments, e.g., ECG and physical examination findings, must be reported as AEs if they represent a clinically significant finding that was not present at baseline or worsened during the course of the study.
- Laboratory test abnormalities must be reported as AEs if they represent a clinically significant finding, symptomatic or not, which was not present at baseline or worsened during the course of the study or led to dose reduction, interruption or permanent discontinuation of study drug.

Adverse events do not include:

- Pre-planned interventions or occurrence of endpoints specified in the study protocol are not considered AE´s, if not defined otherwise (e.g. as a result of overdose)
- Medical or surgical procedure, e.g. surgery, endoscopy, tooth extraction, transfusion. However, the event leading to the procedure is an AE. If this event is serious, the procedure will be described in the SAE narrative.
- Pre-existing disease or medical condition that does not worsen.

- Situations in which an adverse change did not occur, e.g., hospitalizations for cosmetic elective surgery or for social and/or convenience reasons.
- Overdose of either study drug or concomitant medication without any signs or symptoms. However, overdose must be mentioned in the Study Drug Log and reported on an AE form.

12.2.1. Serious Adverse Events (SAEs)

A Serious Adverse Event (SAE) will be defined by the International Conference on Harmonization (ICH) guidelines and WHO GCP guidelines as any AE fulfilling at least one of the following criteria:

- Results in deaths.
- Life-threatening – defined as an event in which the subject was, in the judgment of the investigator, at risk of death at the time of the event; it does not refer to an event that hypothetically might have caused death had it been more severe.
- Requiring subject's hospitalization or prolongation of existing hospitalization – inpatient hospitalization refers to any inpatient admission
- Resulting in persistent or significant disability or incapacity (i.e., a substantial disruption of a person’s ability to conduct normal life functions).
- Congenital anomaly or birth defect.
- Is medically significant or requires intervention to prevent at least one of the outcomes listed above.

Life-threatening refers to an event in which the subject was at risk of death at the time of the event. It does not refer to an event that hypothetically might have caused death if it were more severe. Important medical events that may not immediately result in death, be life-threatening, or require hospitalization may be considered as SAEs when, based upon appropriate medical judgment, they may jeopardize the subject and may require medical or surgical intervention to prevent one of the outcomes listed in the definitions above.

Hospitalization – Prolongation of existing hospitalization

Hospitalization is defined as an overnight stay in a hospital unit and/or emergency room. An additional overnight stay defines a prolongation of existing hospitalization.

The following is not considered an SAE and will be reported as an AE only:

- Treatment on an emergency or outpatient basis for an event not fulfilling the definition of seriousness given above and not resulting in hospitalization.

The following reasons for hospitalizations are not considered AEs, and therefore not SAEs:

- Hospitalizations for cosmetic elective surgery, social and/or convenience reasons.
- Standard monitoring of a pre-existing disease or medical condition that did not worsen, e.g., hospitalization for coronary angiography in a subject with stable angina pectoris.
- Elective treatment of a pre-existing disease or medical condition that did not worsen, e.g., hospitalization for chemotherapy for cancer, elective hip replacement for arthritis.

12.2.2. SAEs related to study-mandated procedures

Such SAEs are defined as SAEs that appear to have a reasonable possibility of causal relationship (i.e., a relationship cannot be ruled out) to study-mandated procedures (excluding administration of study drug) such as discontinuation of subject's previous treatment during a washout period, or complication of a mandated invasive procedure (e.g., blood sampling, heart catheterization), or car accident on the way to the hospital for a study visit, etc.

12.2.3. Suspected unexpected serious adverse reactions (SUSARs)

SUSARs are all serious adverse reactions with suspect causal relationship to the study drug that is unexpected (not previously described in the Investigator's brochure) and serious.

12.2.4. Pregnancy

Pregnancy itself is not an AE/SAE; the outcome of a pregnancy can be a SAE. Any pregnancy that occurs during study participation will be reported to the investigator/sponsor. To ensure subject safety, each pregnancy will be reported to the investigator/sponsor immediately. The pregnancy will be followed up to determine outcome (including premature termination) and status of mother and child. Pregnancy complications and elective terminations for medical reasons will be reported as an AE

or SAE. Spontaneous abortions will be reported as an SAE. Any SAE occurring in association with a pregnancy brought to the investigator's attention after the subject has completed the study and considered by the investigator as possibly related to the investigational product, will be promptly reported to the principal investigator/sponsor. In addition, the investigator will attempt to collect pregnancy information on any female partners of male study subjects who become pregnant while the subject is enrolled in the study. Pregnancy information will be reported to the investigator/sponsor as described above.

12.2.5. Adverse events of special interest

Adverse events of special interest (AESI) in this clinical trial should be reported along with their respective symptoms (e.g. hives, chill, and fever for infusion-related reaction) in the same way as described for SAEs (see Section 10.6).

AESIs in this trial are:

- Infusion-related reactions to IMP ≥ grade 3
- Cytokine release syndrome
- Allergic reaction to IMP
- Infections ≥ grade 3
- Neutropenia ≥ grade 3 (< 1.0 G/L)
- Thrombocytopenia ≥ grade 3 (< 50 G/L)
- Major bleeds (defined as critical organ bleed or hemoglobin decrease of more than 20 g/L within 24 h)
- Hepatitis B reactivation

12.3. Severity of adverse events

The severity of clinical AEs will be graded on a three-point scale: mild, moderate, severe, and reported on specific AE pages of the CRF. If the severity of an AE worsens during study drug administration, only the worst intensity should be reported on the AE page. If the AE lessens in intensity, no change in the severity is required. If an AE occurs during a washout or placebo run-in phase and afterwards worsens during the treatment phase, a new AE page will be filled in with the intensity observed during study drug administration.

Mild: Event may be noticeable to subject; does not influence daily activities; the AE resolves spontaneously or may require minimal therapeutic intervention;
Moderate: Event may make subject uncomfortable; performance of daily activities may be influenced; intervention may be needed; the AE produces no sequelae.
Severe: Event may cause noticeable discomfort; usually interferes with daily activities; subject may not be able to continue in the study; the AE produces sequelae, which require prolonged therapeutic intervention.
A mild, moderate or severe AE may or may not be serious. These terms are used to describe the intensity of a specific event (as in mild, moderate, or severe myocardial infarction). However, a severe event may be of relatively minor medical significance (such as severe headache) and is not necessarily serious. For example, nausea lasting several hours may be rated as severe, but may not be clinically serious. Fever of 39°C that is not considered severe may become serious if it prolongs hospital discharge by a day. Seriousness rather than severity serves as a guide for defining regulatory reporting obligations.

12.4. Relationship to study drug

For all AEs, the investigator will assess the causal relationship between the study drug and the AE using his/her clinical expertise and judgment according to the following algorithm that best fits the circumstances of the AE:

12.4.1. Unrelated

- May or may not follow a reasonable temporal sequence from administration of the study product
- Is biologically implausible and does not follow known response pattern to the suspect study drug (if response pattern is previously known).
- Can be explained by the known characteristics of the subject's clinical state or other modes of therapy administered to the subject.
- Unlikely
  -May or may not follow a reasonable temporal sequence from administration of the study product
  -Is biologically not very plausible
  -May be explained by the known characteristics of the subject's clinical state or other modes of therapy administered to the subject.

12.4.2. Related

Possible related

- Follows a reasonable temporal sequence form administration of the study drug.
- May follow a known response pattern to the study drug (if response pattern is previously known).
- Could not be reasonably explained by the known characteristics of the subject's clinical state or other modes of therapy administered to the subject, if applicable.
- Probable
  -Follows a reasonable temporal sequence form administration of the study drug.
  -Follows a known response pattern to the study drug (if response pattern is previously known).
  -other causes for the event are unlikely

Definitely related

- Follows a reasonable temporal sequence form administration of the study drug.
- Follows a known response pattern to the study drug (if response pattern is previously known).
- No other reasonable cause is present.

12.5. Reporting procedures for any AEs

A special section is designated to adverse events in the case report form. The following details must thereby be entered:

- Type of adverse event
- Start (date and time)
- End (date and time)
- Severity (mild, moderate, severe)
- Serious (no / yes)
- Unexpected (no / yes)
- Outcome (resolved, ongoing, ongoing – improved, ongoing – worsening)
- Relation to study drug (unrelated, related)

Adverse events are to be documented in the case report form in accordance with the above mentioned criteria.

12.6. Reporting procedures for SAEs and AESIs

In the event of serious, the investigator has to use all supportive measures for best patient treatment. A written report is also to be prepared and made available to the clinical investigator immediately. SAEs and AESIs should be reported within 24 h. The following details should at least be available:

- Patient number; ID (study identification number)
- Patient: age, sex, ethical origin
- Name of investigator and investigating site
- Period of administration
- The suspected investigational medical product (IMP)
- The adverse event assessed as serious
- Concomitant disease and medication
- Short description of the event:
    - Description
    - Onset and if applicable, end
    - Therapeutic intervention
    - Causal relationship
    - Hospitalization of prolongation of hospitalization
    - Death, life-threatening, persistent or significant disability or incapacity

If applicable, the initial report should be followed by the Follow up report, indicating the outcome of the SAE/AESI.

All SAEs/AESIs will be recorded on a CIOMS Form and submitted to:

SAE/AESI Reporting	Contact Information
Hi-Bio Safety Vendor: UBC	**TBD**

12.7. Reporting procedures for SUSARs

It must be remembered that the regulatory authorities, and in case of SUSARs which could possibly concern the safety of the study participants, also the Institutional Review

Board / Independent Ethics Committee (IRB / IEC) are to be informed. Such reports shall be made by the study management and the following details should be at least available:

- Patient number; ID (study identification number)
- Patient: age, sex, ethical origin
- Name of investigator and investigating site
- Period of administration
- The suspected investigational medical product (IMP)
- The adverse event assessed as serious and unexpected, and for which there is a reasonable suspected causal relationship to the IMP
- Concomitant disease and medication
- Short description of the event:
    - Description
    - Onset and if applicable, end
    - Therapeutic intervention
    - Causal relationship
    - Hospitalization of prolongation of hospitalization
    - Death, life-threatening, persistent or significant disability or incapacity

Electronic reporting should be the expected method for reporting of SUSARs to the competent authority.

12.8. The Development Safety Update Report (DSUR)

The DSUR will be provided by the principal investigator at least once a year.
This report will also be presented annually to the Independent Ethics (IEC) and to the competent authorities by the sponsor.

13. STUDY TERMINATION, WITHDRAWAL, REPLACEMENT

13.1. Criteria for withdrawal

Subjects may prematurely discontinue from the study at any time. Premature discontinuation from the study is to be understood when the subject did not undergo EOS examination and / or all pivotal assessments during the study.

Subjects MUST discontinue investigational product (and non-investigational product at the discretion of the investigator) and study participation will be terminated for any of the following reasons:

a) Withdrawal of informed consent (subject's decision to withdraw for any reason)
b) Any clinical adverse event (AE), laboratory abnormality or intercurrent illness which, in the opinion of the investigator, indicates that continued participation in the study is not in the best interest of the subject
c) New onset of pregnancy
d) Instruct WOCBP to contact the investigator or study staff immediately if they suspect they might be pregnant (e.g. missed or late menstrual period) at any time during study participation. Institutional policy and local regulations should determine the frequency of on-study pregnancy tests for WOCBP enrolled in the study.
e) The investigator must immediately notify Hi-Bio if a study subject becomes pregnant. The mechanism for reporting pregnancy is described above.
f) Termination of the study by the sponsor.
g) Loss of ability to freely provide consent through imprisonment or involuntary incarceration for treatment of either a psychiatric or physical (eg, infectious disease) illness
h) Inability to comply with protocol
i) By discretion of the investigator
j) Evidence of confirmed hepatic decompensation (Child-Pugh Class B or C, Score > 6)
k) ALT ≥ 5 x baseline OR ≥ 10x ULN AND either total bilirubin > 2x ULN or INR > 2x ULN
l) Platelets count <25,000/mL
m) Any Grade 4 AE or clinical laboratory finding considered study drug related

The only exception to this requirement is when a subject withdraws consent for all study procedures or loses the ability to consent freely (i.e., is imprisoned or involuntarily incarcerated for the treatment of either a psychiatric or physical illness). If a subject was withdrawn before completing the study, the reason for withdrawal must be entered on the appropriate case report form (CRF) page. In all cases, the reason why subjects are withdrawn will be recorded in detail in the CRF and in the subject's medical records.

Should the study be discontinued prematurely, all study materials (complete, partially completed and empty CRFs) will be retained.

13.2. Follow-up of patients withdrawn from the study

In case of premature discontinuation after study drug intake, the investigations scheduled for the EOS visit will be performed 28 days after study drug discontinuation. The subjects will be advised that participation in these investigations is voluntary. Furthermore, they may request that from the time point of withdrawal no more data will be recorded and that all biological samples collected in the course of the study will be destroyed.

13.3. Premature termination of the study

The sponsor has the right to close this study at any time. The IEC and the competent regulatory authority must be informed within 15 days of early termination.
The trial or single dose steps will be terminated prematurely in the following cases:

- If the overall pattern of related SAEs or changes in safety lab results strongly supports a major safety signal, then the DSMB may consider stopping the study.
- If the number of dropouts is so high that proper completion of the trial cannot realistically be expected.

14. ETHICAL ISSUES

The study will be conducted in accordance with the principles of the Declaration of Helsinki 2008. Ethical committee approval will be obtained for all aspects of the study. All study participants will be asked to sign the informed consent to participate in the study (patient insurance included).

15. REGULATORY REQUIREMENTS

We will adhere to all the trial-related requirements, Good Clinical Practice (GCP) requirements (ICH GCP), Good Laboratory Practice (GLP) and the applicable regulatory requirements.

16. PERIODIC MONITORING

The designated monitor (Clinical Trials Coordination Centres – Vienna/Berlin/Grenoble) will contact and visit the investigator regularly and will be allowed to have access to all source documents needed to verify the entries in the CRFs and other protocol-related documents provided that subject confidentiality is maintained in agreement with local regulations. It will be the monitor's responsibility to inspect the CRFs at regular intervals according to the monitoring plan throughout the study, to verify the adherence to the protocol and the completeness, consistency and accuracy of the data being entered on them. The monitoring standards require full verification for the presence of informed consent, adherence to the inclusion/exclusion criteria, documentation of SAEs/AESIs and the recording of the main efficacy, safety, and tolerability endpoints. The monitor will be working according to SOPs and will provide a monitoring report after each visit. The investigator will resolve discrepancies of data. Source data will be checked by the monitor (which means 100% SDV).

17. AUDIT AND INSPECTIONS

Upon request, the investigator will make all study-related source data and records available to a qualified quality assurance auditor mandated by the sponsor or to competent authority inspectors. The main purposes of an audit or inspection are to confirm that the rights and welfare of the subjects have been adequately protected, and that all data relevant for assessment of safety and efficacy of the investigational product have appropriately been reported to the sponsor in accordance with applicable legislation.

18. INVESTIGATOR TRAINING

All investigators and their study personnel will receive training regarding the study procedures and GCP/regulations specific to the conduct of clinical trials. This training will be documented and will take place prior to enrollment and throughout the study as necessary.

19. RECORD RETENTION

Essential documents will be retained as required by the applicable regulatory requirements. An agreement with HI-Bio may be adhered to in addition.

20. STUDY REGISTRATION

The study is planned to be approved by the Austrian regulatory authority (Federal Office for Safety in Health Care, Austrian Agency for Health and Food Safety) and German regulatory authority (The Paul-Ehrlich-Institute, PEI). We plan to register the study to the European Clinical Trials Database (EUDRACT) and a public clinical trial database (http://clinicaltrial.gov). The study protocol will be submitted for publication to *Trials* (http://www.trialsjournal.com/).

21. MODIFICATIONS OF THE PROTOCOL

21.1. Protocol Amendments

Protocol amendments, except where necessary to eliminate an immediate hazard to patients, will be made with the prior approval of HI-Bio. Each applicable Regulatory Authority/IRB/EC/IEC will review and approve amendments prior to their implementation. Regulatory Authority/IRB/EC/IEC approval need not be obtained prior to removal of an immediate hazard to patients.

21.2. Protocol Violations and Deviations

Protocol waivers will not be permitted except where necessary to eliminate an immediate hazard to patients. The Principal Investigator or designee must document and explain in the subject’s source documentation any deviation from the approved protocol. A deviation from the protocol is an unintended and/or unanticipated departure from the procedures and/or processes approved by the Sponsor and the IEC and agreed to by the Principal Investigator. Protocol violations and deviations will be documented by the clinical monitor throughout the course of monitoring visits. Principal Investigator will be notified of violations and/or deviations in writing by the monitor. The IEC will be notified of all protocol violations and deviations according to IEC reporting requirements.

22. REFERENCES

1. Loupy, A, Lefaucheur, C: Antibody-Mediated Rejection of Solid-Organ Allografts. *N Engl J Med,* 379**:** 1150-1160, 2018

2. Budde, K, Dürr, M: Any Progress in the Treatment of Antibody-Mediated Rejection? *J Am Soc Nephrol,* 29**:** 350-352, 2018

3. Böhmig, GA, Eskandary, F, Doberer, K, Halloran, PF: The therapeutic challenge of late antibody-mediated kidney allograft rejection. *Transpl Int,* 32**:** 775-788, 2019

4. Schinstock, CA, Mannon, RB, Budde, K, Chong, AS, Haas, M, Knechtle, S, et al.: Recommended Treatment for Antibody-mediated Rejection After Kidney Transplantation: The 2019 Expert Consensus From the Transplantion Society Working Group. *Transplantation,* 104**:** 911-922, 2020

5. Theruvath, TP, Saidman, SL, Mauiyyedi, S, Delmonico, FL, Williams, WW, Tolkoff-Rubin, N, et al.: Control of antidonor antibody production with tacrolimus and mycophenolate mofetil in renal allograft recipients with chronic rejection. *Transplantation,* 72**:** 77-83, 2001

6. Lederer, SR, Friedrich, N, Banas, B, Welser, G, Albert, ED, Sitter, T: Effects of mycophenolate mofetil on donor-specific antibody formation in renal transplantation. *Clin Transplant,* 19**:** 168-174, 2005

7. Schwarz, C, Mayerhoffer, S, Berlakovich, GA, Steininger, R, Soliman, T, Watschinger, B, et al.: Long-term outcome of belatacept therapy in de novo kidney transplant recipients - a case-match analysis. *Transplant International,* 28**:** 820-827, 2015

8. Fehr, T, Rusi, B, Fischer, A, Hopfer, H, Wüthrich, RP, Gaspert, A: Rituximab and intravenous immunoglobulin treatment of chronic antibody-mediated kidney allograft rejection. *Transplantation,* 87**:** 1837-1841, 2009

9. Billing, H, Rieger, S, Süsal, C, Waldherr, R, Opelz, G, Wühl, E, et al.: IVIG and rituximab for treatment of chronic antibody-mediated rejection: a prospective study in paediatric renal transplantation with a 2-year follow-up. *Transpl Int,* 25**:** 1165-1173, 2012

10. Cooper, JE, Gralla, J, Klem, P, Chan, L, Wiseman, AC: High dose intravenous immunoglobulin therapy for donor-specific antibodies in kidney transplant recipients with acute and chronic graft dysfunction. *Transplantation,* 97**:** 1253-1259, 2014

11. Bachelet, T, Nodimar, C, Taupin, JL, Lepreux, S, Moreau, K, Morel, D, et al.: Intravenous immunoglobulins and rituximab therapy for severe transplant glomerulopathy in chronic antibody-mediated rejection: a pilot study. *Clin Transplant,* 29**:** 439-446, 2015

12. Walsh, RC, Alloway, RR, Girnita, AL, Woodle, ES: Proteasome inhibitor-based therapy for antibody-mediated rejection. *Kidney Int,* 81**:** 1067-1074, 2012

13. Eskandary, F, Jilma, B, Mühlbacher, J, Wahrmann, M, Regele, H, Kozakowski, N, et al.: Anti-C1s monoclonal antibody BIVV009 in late antibody-mediated kidney allograft rejection-results from a first-in-patient phase 1 trial. *Am J Transplant,* 18**:** 916-926, 2017

14. Eskandary, F, Regele, H, Baumann, L, Bong, G, Kozakowski, N, Wahrmann, M, et al.: A randomized trial of bortezomib in late antibody-mediated rejection (BORTEJECT). *J Am Soc Nephrol,* 29**:** 591-605, 2018

15. Moreso, F, Crespo, M, Ruiz, JC, Torres, A, Gutierrez-Dalmau, A, Osuna, A, et al.: Treatment of chronic antibody mediated rejection with intravenous immunoglobulins and rituximab: A multicenter, prospective, randomized, double-blind clinical trial. *Am J Transplant,* 18**:** 927-935, 2018

16. Kulkarni, S, Kirkiles-Smith, NC, Deng, YH, Formica, RN, Moeckel, G, Broecker, V, et al.: Eculizumab therapy for chronic antibody-mediated injury in kidney transplant recipients: a pilot randomized controlled trial. *Am J Transplant,* 17**:** 682-691, 2017

17. Jordan, SC, Ammerman, N, Choi, J, Kumar, S, Huang, E, Toyoda, M, et al.: Interleukin 6: An Important Mediator of Allograft Injury. *Transplantation*, 2020

18. Doberer, K, Duerr, M, Halloran, PF, Eskandary, F, Budde, K, H., R, et al.: A Randomized Trial of Anti-Interleukin-6 Antibody Clazakizumab in Late Antibody-Mediated Kidney Transplant Rejection. *J Am Soc Nephrol*, in press

19. Parkes, MD, Halloran, PF, Hidalgo, LG: Evidence for CD16a-mediated NK cell stimulation in antibody-mediated kidney transplant rejection. *Transplantation,* 101**:** e102-e111. doi: 110.1097/TP.0000000000001586., 2017

20. Yazdani, S, Callemeyn, J, Gazut, S, Lerut, E, de Loor, H, Wevers, M, et al.: Natural killer cell infiltration is discriminative for antibody-mediated rejection and predicts outcome after kidney transplantation. *Kidney International,* 95**:** 188-198, 2019

21. Horenstein, AL, Faini, AC, Morandi, F, Bracci, C, Lanza, F, Giuliani, N, et al.: The Circular Life of Human CD38: From Basic Science to Clinics and Back. *Molecules,* 25, 2020

22. van de Donk, NWCJ, Richardson, PG, Malavasi, F: CD38 antibodies in multiple myeloma: back to the future. *Blood,* 131**:** 13-29, 2018

23. de Weers, M, Tai, Y-T, van der Veer, MS, Bakker, JM, Vink, T, Jacobs, DCH, et al.: Daratumumab, a Novel Therapeutic Human CD38 Monoclonal Antibody, Induces Killing of Multiple Myeloma and Other Hematological Tumors. *The Journal of Immunology,* 186**:** 1840-1848, 2011

24. Overdijk, MB, Verploegen, S, Bogels, M, van Egmond, M, Lammerts van Bueren, JJ, Mutis, T, et al.: Antibody-mediated phagocytosis contributes to the anti-tumor activity of the therapeutic antibody daratumumab in lymphoma and multiple myeloma. *MAbs,* 7**:** 311-321, 2015

25. Overdijk, MB, Jansen, JH, Nederend, M, Lammerts van Bueren, JJ, Groen, RW, Parren, PW, et al.: The Therapeutic CD38 Monoclonal Antibody Daratumumab Induces Programmed Cell Death via Fcgamma Receptor-Mediated Cross-Linking. *J Immunol,* 197**:** 807-813, 2016

26. Doberer, K, Kläger, J, Gualdoni, GA, Mayer, KA, Eskandary, F, Farkash, EA, et al.: CD38 Antibody Daratumumab for the Treatment of Chronic Active Antibody-mediated Kidney Allograft Rejection. *Transplantation,* Publish Ahead of Print, 2020

27. Kwun, J, Matignon, M, Manook, M, Guendouz, S, Audard, V, Kheav, D, et al.: Daratumumab in Sensitized Kidney Transplantation: Potentials and Limitations of Experimental and Clinical Use. *Journal of the American Society of Nephrology,* 30**:** 1206-1219, 2019

28. Raab, MS, Engelhardt, M, Blank, A, Goldschmidt, H, Agis, H, Blau, IW, et al.: MOR202, a novel anti-CD38 monoclonal antibody, in patients with relapsed or refractory multiple myeloma: a first-in-human, multicentre, phase 1-2a trial. *Lancet Haematol,* 7**:** e381-e394, 2020

29. Teiluf, K, Seidl, C, Blechert, B, Gaertner, FC, Gilbertz, KP, Fernandez, V, et al.: alpha-Radioimmunotherapy with (2)(1)(3)Bi-anti-CD38 immunoconjugates is effective in a mouse model of human multiple myeloma. *Oncotarget,* 6**:** 4692-4703, 2015

30. Busch, L, Mougiakakos, D, Buttner-Herold, M, Muller, MJ, Volmer, DA, Bach, C, et al.: Lenalidomide enhances MOR202-dependent macrophage-mediated effector functions via the vitamin D pathway. *Leukemia,* 32**:** 2445-2458, 2018

31. Flamann, C, Busch, L, Mackensen, A, Bruns, H: Combination of lenalidomide and vitamin D enhances MOR202-mediated cytotoxicity of macrophages: It takes three to tango. *Oncotarget,* 10**:** 10-12, 2019

32. Irish, W, Nickerson, P, Astor, BC, Chong, E, Wiebe, C, Moreso, F, et al.: Change in Estimated GFR and Risk of Allograft Failure in Patients Diagnosed With Late Active Antibody-mediated Rejection Following Kidney Transplantation. *Transplantation,* Publish Ahead of Print, 2020
33. Loupy, A, Aubert, O, Orandi, BJ, Naesens, M, Bouatou, Y, Raynaud, M, et al.: Prediction system for risk of allograft loss in patients receiving kidney transplants: international derivation and validation study. *BMJ,* 366**:** l4923, 2019
34. Doberer, K, Schiemann, M, Strassl, R, Haupenthal, F, Dermuth, F, Gorzer, I, et al.: Torque teno virus for risk stratification of graft rejection and infection in kidney transplant recipients-A prospective observational trial. *Am J Transplant,* in press, 2020
35. Halloran, PF, Famulski, KS, Reeve, J: Molecular assessment of disease states in kidney transplant biopsy samples. *Nat Rev Nephrol,* 12**:** 534-548, 2016
36. Schwaiger, E, Wahrmann, M, Bond, G, Eskandary, F, Böhmig, GA: Complement component C3 activation: the leading cause of the prozone phenomenon affecting HLA antibody detection on single-antigen beads. *Transplantation,* 97**:** 1279-1285, 2014
37. Loupy, A, Haas, M, Roufosse, C, Naesens, M, Adam, B, Afrouzian, M, et al.: The Banff 2019 Kidney Meeting Report (I): Updates on and clarification of criteria for T cell– and antibody-mediated rejection. *American Journal of Transplantation***:** ajt.15898, 2020
38. Halloran, PF, Reeve, J, Akalin, E, Aubert, O, Böhmig, GA, Brennan, D, et al.: Real time central assessment of kidney transplant indication biopsies by microarrays: the INTERCOMEX study. *Am J Transplant,* 17**:** 2851-2862, 2017
39. Reeve, J, Böhmig, GA, Eskandary, F, Einecke, G, Lefaucheur, C, Loupy, A, et al.: Precision molecular phenotyping of kidney transplant biopsies using archetypal analysis. *JCI Insight,* 2**:** e94197, 2017
40. Mühlbacher, J, Doberer, K, Kozakowski, N, Regele, H, Camovic, S, Haindl, S, et al.: Non-invasive Chemokine Detection: Improved Prediction of Antibody-Mediated Rejection in Donor-Specific Antibody-Positive Renal Allograft Recipients. *Front Med (Lausanne),* 7**:** 114, 2020
41. Bloom, RD, Bromberg, JS, Poggio, ED, Bunnapradist, S, Langone, AJ, Sood, P, et al.: Cell-Free DNA and Active Rejection in Kidney Allografts. *J Am Soc Nephrol,* 28**:** 2221-2232, 2017

42. Geissler, EK: The ONE Study compares cell therapy products in organ transplantation: introduction to a review series on suppressive monocyte-derived cells. *Transplant Res,* 1**:** 11, 2012
43. Streitz, M, Miloud, T, Kapinsky, M, Reed, MR, Magari, R, Geissler, EK, et al.: Standardization of whole blood immune phenotype monitoring for clinical trials: panels and methods from the ONE study. *Transplant Res,* 2**:** 17, 2013
44. Banham, GD, Flint, SM, Torpey, N, Lyons, PA, Shanahan, DN, Gibson, A, et al.: Belimumab in kidney transplantation: an experimental medicine, randomised, placebo-controlled phase 2 trial. *Lancet,* 391**:** 2619-2630, 2018
45. Maggi, F, Pifferi, M, Fornai, C, Andreoli, E, Tempestini, E, Vatteroni, M, et al.: TT virus in the nasal secretions of children with acute respiratory diseases: relations to viremia and disease severity. *J Virol,* 77**:** 2418-2425, 2003
46. Schiemann, M, Puchhammer-Stockl, E, Eskandary, F, Kohlbeck, P, Rasoul-Rockenschaub, S, Heilos, A, et al.: Torque Teno virus load-inverse association with antibody-mediated rejection after kidney transplantation. *Transplantation,* 101**:** 360-367, 2017
47. Mateos, MV, Dimopoulos, MA, Cavo, M, Suzuki, K, Jakubowiak, A, Knop, S, et al.: Daratumumab plus Bortezomib, Melphalan, and Prednisone for Untreated Myeloma. *N Engl J Med,* 378**:** 518-528, 2018
48. Moreau, P, Attal, M, Hulin, C, Arnulf, B, Belhadj, K, Benboubker, L, et al.: Bortezomib, thalidomide, and dexamethasone with or without daratumumab before and after autologous stem-cell transplantation for newly diagnosed multiple myeloma (CASSIOPEIA): a randomised, open-label, phase 3 study. *Lancet,* 394**:** 29-38, 2019
49. Mayer KA, Schrezenmeier E, Diebold M, Halloran PF, Schatzl M, Schranz S, et al. A Randomized Phase 2 Trial of Felzartamab in Antibody-Mediated Rejection. N Engl J Med. 2024;391(2):122-32.
50. Oellerich M, Shipkova M, Asendorf T, Walson PD, Schauerte V, Mettenmeyer N, et al. Absolute quantification of donor-derived cell-free DNA as a marker of rejection and graft injury in kidney transplantation: Results from a prospective observational study. Am J Transplant. 2019;19(11):3087-99.
51. Oellerich M, Budde K, Osmanodja B, Bornemann-Kolatzki K, Beck J, Schütz E, et al. Donor-derived cell-free DNA as a diagnostic tool in transplantation. Front Genet. 2022;13:1031894.

52. Raab MS, Engelhardt M, Blank A, Goldschmidt H, Agis H, Blau IW, et al. MOR202, a novel anti-CD38 monoclonal antibody, in patients with relapsed or refractory multiple myeloma: a first-in-human, multicentre, phase 1-2a trial. Lancet Haematol. 2020;7(5):e381-e94.

Summary of changes

Protocol Version	Date
**Original Protocol (Version 2.0)**	30 April 2021
**Amendment 1 (Protocol Version 3.0)**	29 March 2022
**Amendment 2 (Protocol Version 4.0)**	08 December 2022
**Amendment 3 (Protocol Version 5.0)**	25 May 2023
**Amendment 4 (Protocol Version 6.0)**	14 October 2023
**Amendment 5 (Protocol Version 7.0)**	6 August 2024

The reasons for amendments are listed, starting with the first amendment:

**Amendment 1**	**Protocol Version 3.0 (29 March 2022)**<br>**Changes in the protocol:**
Increase in the number of trial sites: Inclusion of a third center: Grenoble Alpes University Hospital; France	*Modification of the investigator list.*<br><br>*Site mentioned in Section 4.*

**Amendment 2**	**Protocol Version 4.0 (08 December 2022)**<br>**Changes in the protocol:**
Increase of patient number to 22	Section 3.<br>*The objective of our planned randomized controlled pilot trial (inclusion of 2 transplant centers) will be to evaluate the safety, tolerability and efficacy of CD38 monoclonal antibody felzartamab in a prospective cohort of 22 kidney transplant recipients diagnosed with late active or chronic active ABMR.*<br><br>Section 4.2.<br>*Inclusion and exclusion criteria are listed in Table 2. We plan to include 22 kidney transplant recipients (considering an anticipated drop-out rate of 1-2 patients, this planned case number is expected to allow for at least 20 participants completing the trial).*

**Amendment 3**	**Protocol Version 5.0 (25 May 2023)**<br>**Changes in the protocol:**
(i) Transfer of exclusive rights to develop and commercialize felzartamab from Morphosys AG, Planegg, Germany, to Human Immunology Biosciences, Inc. (HI-Bio), South San Francisco, CA).	Section 2.1.<br>*Felzartamab (MOR202; also referred to as MOR03087; Human Immunology Biosciences, Inc. [HI-Bio], South San Francisco, CA, USA) is a recombinant fully human monoclonal CD38 antibody (IgG1λ) derived from a proprietary antibody phage library (produced in human PER.C6® cells by recombinant expression technology).*

	Section 4.<br>*The funder (HI-Bio, South San Francisco, CA, USA) has set funding conditions and will provide external funding.*<br><br>Section 5.0.<br>*Felzartamab (MOR202; MOR03087) will be supplied and provided by Hi-Bio.*<br><br>Section 10.4.<br>*Under no circumstances shall felzartamab injection be administered to women known to be pregnant or lactating. All pregnancies must be reported to Hi-Bio within 24 hours and in accordance with SAE reporting procedures.*<br><br>Section 11.6.<br>*Hi-Bio Safety Vendor mentioned in the Table*<br><br>Section 12.1.<br>*The investigator must immediately notify Hi-Bio if a study subject becomes pregnant. The mechanism for reporting pregnancy is described above.*<br><br>*Section 18.*<br>*Essential documents will be retained as required by the applicable regulatory requirements. An agreement with HI-Bio may be adhered to in addition.*<br><br>Section 20.1.<br>*Protocol amendments, except where necessary to eliminate an immediate hazard to patients, will be made with the prior approval of HI-Bio.*
(ii) Inclusion of a new investigator	*Modification of the investigator list: inclusion of Martina Schatzl (Vienna)*
(iii) Grenoble Alpes University not included as a third study site (IRB approval was not obtained before the end of patient recruitment)	*Modification of the investigator list.*<br><br>*Removal of the center from Section 4.*
(iv) Minor wording changes	Section 2.<br>*Mechanisms of action include complement-dependent cytotoxicity (CDC), antibody-dependent cellular cytotoxicity (ADCC)/phagocytosis (ADCP), and/or apoptotic signalling*<br><br>Table 2.<br>*Enumeration of eligibility criteria*

	Section 5.1.<br>*Dosing occurs every week in cycle 1 (C1) and every four weeks in cycle 2-6.*<br><br>Section 5.1.<br>*Felzartamab will be administered after dilution with 250 mL 0.9% sodium chloride solution (final concentration should be between 1 and 20 mg/mL).*<br><br>Section 5.2.<br>*Premedication will be administered 30 min before the infusion of felzartamab, and will consist of Diphenhydramine (30 mg), Paracetamol (1000 mg), and Prednisolone (100 mg), respectively (each in 100 mL Volume). In the placebo arm, patients will receive 3x100 mL NaCl 0.9%.*<br><br>Section 7.11.<br>*Upon screening, we will perform a Quantiferon test and assess HIV/hepatitis serology (1x15 mL).*<br><br>Section 10.4.<br>*There are no adequate well-controlled studies in pregnant or lactating women. All subjects of childbearing potential being treated with felzartamab (and their partners) must be informed of this risk, and use highly effective birth control, as defined in the study protocol.*<br><br>Section 19.<br>*The study is planned to be approved by the Austrian regulatory authority (Federal Office for Safety in Health Care, Austrian Agency for Health and Food Safety) and German regulatory authority (The Paul-Ehrlich-Institute, PEI).*

**Amendment 4**	**Protocol Version 6.0 (14 October 2023)**<br>**Changes in the protocol:**
(i) Options for treatment extension after finalization of the trial	*New section (section 10) describing options for treatment extension*
(ii) Modification of investigator list	*Inclusion of Dr. Eva Schrezenmeier (Berlin) as a new investigator*<br><br>*Removal of Dr. Michael Dürr as an investigator*

**Amendment 5**	**Protocol Version 7.0 (6 August 2024)**<br>**Changes in the protocol:**
(i) Modifications of the open-label felzartamab treatment extension	Section 10<br>*After the initial 6 months of felzartamab therapy, antibody administration will be continued in a tailored (“on-demand”) manner based on the course of donor-derived cell-free DNA, up to month 12. This approach aims to minimize the risk of recurrent rejection at 12 months.*<br><br>*Monitoring of Felzartamab pharmacokinetics and anti-drug antibody (ADA) formation will be performed during the study extension.*<br><br>*Following completion and publication of the first phase of the Phase 2 study (Mayer et al.,* New England Journal of Medicine*, 2024; Reference 49), the protocol now summarizes key efficacy results, including results on donor-derived cell-free DNA.*<br><br>Section 11<br>*Following completion and publication of the first phase of the Phase 2 study (Mayer et al.,* New England Journal of Medicine*, 2024; Reference 49), the protocol now summarizes key safety results.*<br><br>*Patients entering the study extension phase (Option B) from August 2024 onward: Adjustment of premedication and infusion rates for the first two infusions to reduce the intensity and frequency of infusion reactions.*
